# Additional Biomarkers beyond RAS That Impact the Efficacy of Cetuximab plus Chemotherapy in mCRC: A Retrospective Biomarker Analysis

**DOI:** 10.1155/2018/5072987

**Published:** 2018-09-16

**Authors:** Peng Zheng, Chunmin Liang, Li Ren, Dexiang Zhu, Qingyang Feng, Wenju Chang, Guodong He, Lechi Ye, Jingwen Chen, Qi Lin, Tuo Yi, Meiling Ji, Zhengchuan Niu, Mi Jian, Ye Wei, Jianmin Xu

**Affiliations:** ^1^Department of General Surgery, Zhongshan Hospital, Fudan University, Shanghai, China; ^2^Department of Anatomy, Histology & Embryology, Shanghai Medical College, Fudan University, Shanghai, China; ^3^Department of Oncological Surgery, The First Affiliated Hospital of Wenzhou Medical University, Wenzhou, China

## Abstract

**Purpose:**

We aimed to identify new predictive biomarkers for cetuximab in first-line treatment for patients with RAS wild-type metastatic colorectal cancer (mCRC).

**Methods:**

The study included patients with KRAS wild-type unresectable liver-limited mCRC treated with chemotherapy with or without cetuximab. Next-generation sequencing was done for single nucleotide polymorphism according to custom panel. Potential predictive biomarkers were identified and integrated into a predictive model within a training cohort. The model was validated in a validation cohort.

**Results:**

Thirty-one of 247(12.6%) patients harbored RAS mutations. In training cohort (N=93), six potential predictive genes, namely, ATP6V1B1, CUL9, ERBB2, LY6G6D, PTCH1, and RBMXL3, were identified. According to predictive model, patients were divided into responsive group (n=66) or refractory group (n=27). In responsive group, efficacy outcomes were significantly improved by addition of cetuximab to chemotherapy. In refractory group, no benefit was observed. Interaction test was significant across all endpoints. In validation cohort (N=123), similar results were also observed.

**Conclusions:**

In the first-line treatment of mCRC, the predictive model integrating six new predictive mutations divided patients well, indicating a promising approach to further refine patient selection for cetuximab on the basis of RAS mutations.

## 1. Introduction

Cetuximab plus chemotherapy regimens are typically used in the first-line treatment of RAS wild-type metastatic colorectal cancer (mCRC) [1–3]. Our previous trial [4] (NCT01564810) compared first-line chemotherapy plus cetuximab with chemotherapy alone in Chinese patients with initially unresectable liver-limited KRAS exon 2 wild-type mCRC and achieved the primary end point of the conversion rate to the radical resection of liver metastases (LM), whereas the objective response rate (ORR) was finitely improved by exclusion of patients with RAS mutations. Results of many other trials indicated the same dilemma [1, 2]. To further refine patient selection, other markers, including BRAF mutation [5], PIK3CA mutations, loss of PTEN [6, 7], and amplification of MET and ERBB2 [8, 9], were investigated. But none of markers above really affected clinical practice. Recently, increasing evidences indicated predictive value of primary tumor location, but the underlying biological mechanism was still largely unknown [10].

Even now, for a proportion of patients, the responsible genetic alteration of primary resistance remains unknown [11]. Remarkably, although the mechanisms of resistance were genetically heterogeneous, they were suggested to biochemically converge on the EGFR signaling pathway, but in a remote manner [12]. This knowledge has been translated into a more comprehensive search for predictive biomarkers of EGFR-directed monoclonal antibodies (MoAbs), and next-generation sequencing (NGS) has served as an approach for genome-wide exploration [13, 14].

The aim of this biomarker analysis of Chinese trial was to reassess the efficacy outcomes according to “new” RAS mutations and primary tumor location, identify additional predictive biomarkers by NGS, and further generate a predictive model for patients with wild-type RAS.

## 2. Methods

### 2.1. Study Design and Patients

We conducted a retrospective analysis of gene mutations in two cohorts of patients with sufficient formalin-fixed paraffin-embedded (FFPE) tissue samples. The training cohort was derived from Chinese trial (NCT01564810) [4]. The validation cohort was recruited with same criteria as Chinese trial, between January 2012 and December 2014.

The Chinese trial compared chemotherapy (mFOLFOX6 or FOLFIRI) plus cetuximab with chemotherapy alone as first-line treatment for patients with initially unresectable liver-limited KRAS exon 2 wild-type mCRC. The primary end point was the conversion rate to radical resection for liver metastases, which was assessed by multidisciplinary team (MDT). The trial was approved by the local ethic committees and all patients provided written and oral informed consent, including research on tumor tissue.

Another ten patients were selected for whole exome sequencing (WES). All these patients were diagnosed with colorectal liver metastases and underwent resection of primary and metastatic tumors.

### 2.2. Procedures

To further refine patient selection for cetuximab plus chemotherapy, we searched for new predictive biomarkers through genome-wide exploration using NGS. To preliminarily select genes to construct a custom panel for target capture sequencing, we performed WES for ten triplets, each comprising primary colorectal tumor and normal colorectal mucosa and matched liver metastases, on an Ion™ Proton (Life Technologies, Carlsbad, CA) platform (Supplementary materials). Normal colorectal mucosa was sequenced to exclude germ-line variants. Then we constructed a custom panel based on driver mutations identified using WES and Tumor Mutation Hotspots Panel version 2 (Life Technologies, Carlsbad, CA)[15] (Table S5). Subsequently, patients were sequenced for single nucleotide polymorphism (SNP) on Ion™ Torrent Personal Genome Machine (Life Technologies, Carlsbad, CA) (Supplementary Materials). A 5% cutoff value was employed to call mutations.

Patients were analyzed according to status of RAS mutations first. For patients with wild-type RAS, efficacy according to primary tumor location was analyzed. Then, new predictive biomarkers were identified and integrated into predictive model in training cohort, and the model was validated in validation cohort.

### 2.3. Construction of Customized Panel

To prepare for target capture sequencing, we constructed a customized panel based on WES data and Tumor Mutation Hotspots Panel version 2 (Life Technologies, Carlsbad, CA). We identified a new predictive biomarker for the efficacy of cetuximab on the liver metastases of CRC patients. Thus, biomarkers essential for cancer progression, particularly liver metastasis, were considered a priority. These biomarkers were more potentially correlated with the EGFR signal pathway, which was also essential in liver metastatic tumors. By assessing the WES data, 608 driver mutations in primary tumors and 684 driver mutations in metastatic tumors were identified (Table S3). On one hand, we searched all mutations in the GeneRIF database using key words “cancer/tumor/carcinoma” and “metasta-/invasion/invade/invasive/migrate” and identified 124 genes with universal mutations (Table S4). On the other hand, we focused on 230 mutations in 219 universal genes in primary tumors and corresponding liver metastases in WES data according to cancer evolution model [16]. Among these genes, members of the signal transduction pathway were initially selected into the customized panel. Most genes in the Tumor Mutation Hotspots Panel version 2 were included. In addition, 7 metastases-private mutant genes and 5 genes with high frequency mutations were also included in the panel. The mutation information for these genes was acquired from the Catalog of Somatic Mutations in Cancer (COSMIC) database, and we employed the most frequent mutations to build the panel.

### 2.4. Statistical Analysis

The statistical analysis plan was established before the genotyping results were available. Differences in the baseline characteristics were calculated using a Chi-square test or Fisher's exact test. Survival curves were generated using the Kaplan-Meier method and compared using a log-rank test. Hazard ratios (HRs) and 95% confidence intervals (95%CI) were calculated using the Cox proportional hazards model. Odds ratios (ORs) and 95%CI were calculated using a logistic regression model. All statistical analyses were conducted using the statistical software SPSS version 18.0 (SPSS Inc., Chicago, IL). A p value < 0.05 was considered statistically significant.

Efficacy outcomes according to RAS status were investigated first. Outcomes for patients with wild-type RAS were further analyzed in subgroups defined by primary tumor location and BRAF mutation. Subsequently, among patients with wild-type RAS in the training cohort, potential predictive genes were selected and integrated into a predictive model according to the following procedures.

In first step of selecting procedures, the interaction effect on ORR between each gene and treatment was analyzed. The significance level for denying an explanatory gene was 0.10 to include those borderline genes. Second, interaction tests were adjusted with propensity score. Multivariable logistic regression was used to generate a propensity score based on age, gender, ECOG PS, primary tumor location, number of liver metastases, and maximum size of liver metastases. The significance level for adjusted interaction tests was 0.05. Third, efficacy outcomes according to each selected gene were summarized to show the precise effect on treatment in patients with wild-type RAS.

In procedure of generating predictive model, all potential predictive genes selected, along with treatment, were integrated into a predictive model for objective response using logistic regression model. Because the interactions between these genes and the efficacy of chemotherapy or anti-EGFR therapy were complex and remained largely unknown, potential predictive genes with p > 0.05 were maintained in the predictive model, as denying any of these genes was difficult and unreasonable. The sensitivity and specificity of the model were estimated based on a receiver operating characteristic (ROC) curve. The estimated predictive score representing the predicted possibility of achieving objective response was calculated as the weighted sum of regression coefficients and variable values (see (1)). Each patient was assigned two predictive scores, one

(1) Predictive  score=LogitP=α+β1X1+β2X2+…+βmXm

for receiving cetuximab plus chemotherapy (PS_Cet_) and another for receiving chemotherapy alone (PS_CT_). To display predictive efficacy of the model, predictive scores were dichotomized with a cutoff value determined for the maximum Youden's index. Accordingly, patients were divided into responsive group (PS_Cet_ ≥ cutoff value) or refractory group (PS_Cet_ < cutoff value). Then, efficacy outcomes were analyzed according to model-defined groups. In addition, outcomes of early tumor shrink (ETS) were also examined in model-defined groups. Then the model was applied in the validation cohort.

## 3. Results

### 3.1. Patients and Samples

**Figure 1:**
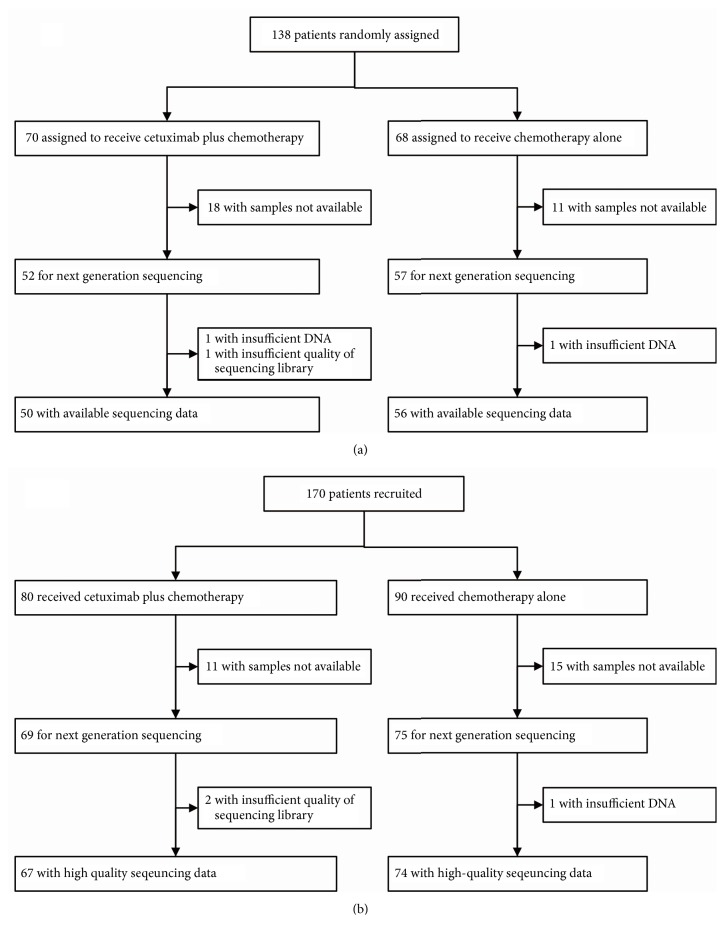
**Patient flow for the training cohort (a) and validation cohort (b)**. Chemotherapy included mFOLFOX6 and FOLFIRI.

A total of 247 patients were sequenced (Figure 1). Thirty-one (12.6%) patients harbored “new” RAS mutations (Table S6 and S7). With respect to BRAF, 24(9.7%) patients harbored a mutation. The detected BRAF mutations were exclusive of RAS mutations. Other mutations were also summarized.

### 3.2. Efficacy according to RAS/BRAF Status

For patients with wild-type RAS (n=216) receiving cetuximab plus chemotherapy, compared with those receiving chemotherapy alone, a significant improvement across all end points was observed (Table 1). In patients with RAS mutations (n=31), differences between arms were not significant. Efficacy outcomes of patients with wild-type RAS/BRAF were similar to those of patients with wild-type RAS (Table S8).

### 3.3. Efficacy according to Primary Tumor Location

Among patients with RAS wild-type left-sided tumors, the addition of cetuximab to chemotherapy significantly improved all efficacy outcomes, as expected based on results of RAS wild-type population, whereas limited benefit was observed upon the addition of cetuximab to chemotherapy in patients with RAS wild-type right-sided tumors (Table 1).

### 3.4. Selection of Potential Predictive Biomarkers

Only mutations with mutational frequencies >10% and <90% (n=54) were investigated for stability. In the first selection procedure, 8 mutations showed significant (p < 0.10) interaction effects on ORR (Table S9). In the second selection procedure, 6 genes remained significant after being adjusted by estimated propensity score based on age (>65 years versus ⩽65 years), gender (male versus female), ECOG PS (0 versus 1), primary tumor location (right-sided versus left-sided + rectum), number of liver metastases (⩽4 versus >4), and maximum size of liver metastases (*⩾*5cm versus <5cm) (Table S10). In the third selection procedure, all 6 genes showed their predictive value in efficacy analysis (Table S11).

### 3.5. Efficacy according to the Predictive Model

**Figure 2:**
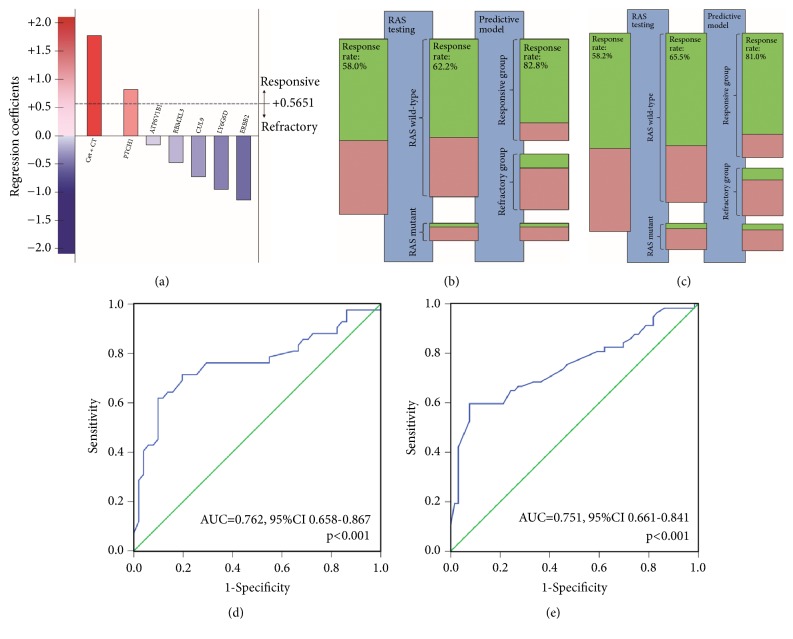
**The predictive model for objective response**. (a) The predictive model is calculated as the sum of treatment and gene mutation predictor values weighted by their regression coefficients. (b) ORR for patients treated with cetuximab plus chemotherapy in training cohort was improved by RAS testing and predictive model. The green bars represent responders (CR + PR); the red bars nonresponders (SD + PD). The size of the bars is in agreement with the corresponding numbers of patients. (c) ORR for patients treated with cetuximab plus chemotherapy in validation cohort was improved by RAS testing and predictive model. (d) ROC curve for the predictive model in training cohort (AUC=0.762, 95%CI 0.658-0.867, P<0.001); (e) ROC curve for the predictive model in validation cohort (AUC=0.751, 95%CI 0.661-0.841, p<0.001). ORR, objective response rate. ROC, receiver operating characteristics.

Using patients with wild-type RAS from the training cohort, a predictive model was generated for objective response. The predictive model was calculated as the sum of the predictor values weighted by regression coefficients and included the addition of cetuximab to chemotherapy (*β*=+1.771), mutations in 6 genes: ATP6V1B1 (*β*=-0.165) CUL9 (*β*=-0.726), ERBB2 (*β*=-1.140), LY6G6D (*β*=-0.994), PTCH1 (*β*=+0.821), and RBMXL3 (*β*=-0.477), and constant value (*α*=-0.255). With a cutoff value of 0.5651 determined for the maximum of Youden's index, patients were further divided into responsive group or refractory group (Figure 2 and Table 2). In responsive group (n=66), ORR was clearly and significantly improved in patients receiving cetuximab plus chemotherapy, compared with chemotherapy alone. In refractory group (n=27), the benefit of addition of cetuximab to chemotherapy on ORR was not apparent. The interaction test was significant. Though designed for ORR, the predictive model also showed predictive value on PFS and OS (Table 2 and Figure S2). Moreover, The HRs and ORs were more favorable towards the addition of cetuximab to chemotherapy in patients from responsive group compared with RAS wild patients.

In validation cohort, patients with wild-type RAS were also divided into a responsive group (n=83) and a refractory group (n=43). Efficacy outcomes keep the same pattern as in training cohort (Table 3 and Figure S3).

## 4. Discussion

In this study, we reported the construction and validation of a predictive model for the response to cetuximab plus chemotherapy in patients with liver-limited mCRC in a first-line treatment setting. The predictive model served as a more effective algorithm to further refine patient selection for cetuximab administration in our results.

Based on the understanding of the mechanisms of EGFR-directed MoAbs, we extracted our efforts to identify new predictive biomarkers. In contrast to previous studies focusing on single gene alterations related to the EGFR pathway [5–9], we used a multivariable approach, including genome-wide exploration using WES, to select predictors according to efficacy outcomes and to construct a multivariable model. The advantage of multivariable modeling is that it is unbiased by biological assumptions and thereby reflects the fact that interactions between distinct gene mutations and the efficacy of chemotherapy and anti-EGFR therapy are complex, interdependent, and largely unknown.

During multivariable modeling, we choose objective response as the main endpoint. Since many patients received resection of LM, PFS was not the best endpoint to choose. Neither was overall survival, because both liver surgery and complicated later line treatment impact overall survival. However, objective response is a robust measurement based on strict criteria and is independent of liver surgery. Moreover, tumor shrinkage and objective response are correlated with long-term outcome for cetuximab [17]. Therefore, objective response was relatively more accurate to indicate efficacy of cetuximab.

According to the model, approximately 30% of patients with wild-type RAS were reclassified into refractory group. The refractory group consisted of patients with a set of gene signatures who were less likely to benefit from anti-EGFR therapy. Administration of cetuximab to these patients needed more consideration and caution according to our results.

With respect to biological functions, most genes in model were related to EGFR pathway in a direct or remote way. ERBB2 (also HER2) and EGFR belong to the same family. The amplification of ERBB2 leads to primary resistance by bypassing EGFR [9]. A recent study indicated that ERBB2 activating mutations could lead to EGFR antibody resistance in colorectal patient-derived xenografts (PDXs) [14], providing experimental support for the clinical findings reported herein. The resistance mechanism of ERBB2 mutations might involve an alternative strategy for ERBB2 pathway activation complementary to ERBB2 amplification. Lymphocyte antigen 6 complex, locus G6D (LY6G6D) belongs to a cluster of leukocyte antigen-6 (LY6) genes. LY6G6D binds to growth-factor-receptor-bound protein2 (Grb2) and Grb7 and activates downstream signal pathways, including the RAS/MAPK pathway [18]. PTCH1 is core node in Hedgehog pathway. There is crosstalk between RAS and Hedgehog and new mechanism is emerging [19]. Moreover, resistance to anti-EGFR drugs in lung cancer is often related to the activation of Hedgehog signaling cascades [20]. CUL9 is the latest member of cullin family, which form E3 ubiquitin ligase to regulate a variety of cellular process by targeted polyubiquitination [21]. And, ubiquitination is essential posttranslational regulation of akt in EGFR pathway [22].

**Figure 3:**
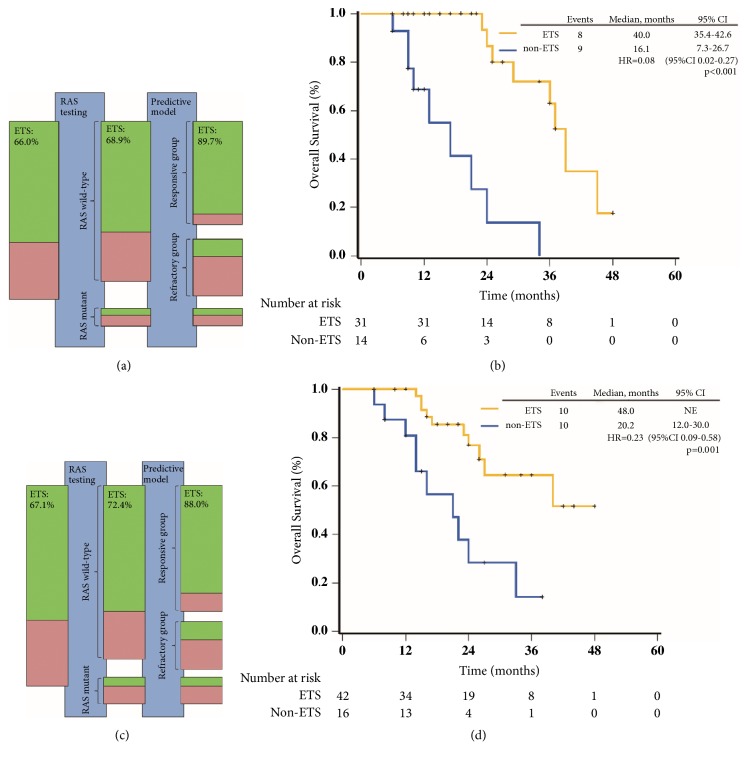
**Predictive model and ETS**. (a) The improvement of ETS rate of patients receiving cetuximab plus chemotherapy in training cohort was gained by RAS testing and predictive model. The green bars represent responders (CR + PR); the red bars nonresponders (SD + PD). The size of the bars is in agreement with the corresponding numbers of patients. (b) Kaplan-Meier curve for OS of patients receiving cetuximab plus chemotherapy in training cohort according to ETS. (c) The improvement of ETS rate of patients receiving cetuximab plus chemotherapy in validation cohort was gained by RAS testing and predictive model. (d) Kaplan-Meier curve for OS of patients receiving cetuximab plus chemotherapy in validation cohort according to ETS. ETS was defined as a ⩾20% reduction of the longest diameters of measurable liver metastases in eight weeks compared with baseline at the first evaluation. ETS, early tumor shrink. PFS, progression-free survival. OS, overall survival.

The predictive value of primary tumor location was widely reported recently. Our results also indicated limited benefit of cetuximab in patients with RAS wild-type right-sided tumors. However, predictive value of the model was likely to be independent of primary tumor location (Table S13). Efficacy of cetuximab plus chemotherapy was significantly improved in patients from responsive group with right-sided tumors. Further, this reminded us of other clinical markers. Previous analysis indicated that ETS was significantly associated with the long-term outcome in patients with wild-type KRAS treated with cetuximab plus chemotherapy [17]. Our results further verified the correlation between ETS and long-term outcomes in patients with wild-type RAS in the same setting (Figure 3). Based on the evidences, ETS was considered as a reasonable clinical predictor to provide early guidance for on-treatment decisions, including the continuation or discontinuation of cetuximab treatment [23]. In this study, the predictive model combined with RAS mutations improved the ORR of patients receiving cetuximab plus chemotherapy to more than 80%. Furthermore, analysis of ETS indicated that about 90% of patients from responsive group achieved ETS and vast majority of them achieved objective response simultaneously. Thereby, with application of ETS, 10% of patients from responsive group who benefit less from cetuximab plus chemotherapy were identified. A possible strategy for the administration of cetuximab arise that pretreatment selection using the predictive model followed by early on-treatment selection through ETS.

When generating the model, we only considered SNPs because of the limited sequencing method. Importantly, SNPs can be detected using other mature methods and widely accepted cutoff values, reflecting the broad applicability of these markers in clinical practice (e.g., RAS mutations). Importantly, other cohorts are also needed to verify the reliability and efficacy of the model. Moreover, with evolving omics and technologies, future studies will improve the model by adding or substituting genetic, epigenetic, or proteomic factors.

In summary, we generated a predictive model for patients with wild-type RAS. The benefit profile of addition of cetuximab was further improved by excluding patients from model-defined refractory group. Validation of this model in subsequent studies would be valuable to further refine patient selection for the administration of cetuximab.

## Figures and Tables

**Table 1:** Efficacy outcomes according to RAS status and primary tumor location.

	RAS assessble (n=247)		RAS wild-type (n=216)		RAS mutant (n=31)		RAS wild-type/ Left-sided (n=158)		RAS wild-type/ Right-sided (n=58)	
	Cetuximab plus chemotherapy (n=117)	Chemotherapy alone (n=130)	Cetuximab plus chemotherapy (n=103)	Chemotherapy alone (n=113)	Cetuximab plus chemotherapy (n=14)	Chemotherapy alone (n=17)	Cetuximab plus chemotherapy (n=74)	Chemotherapy alone (n=84)	Cetuximab plus chemotherapy (n=29)	Chemotherapy alone (n=29)
Overall response										
CR	2 (1.7%)	1 (0.8%)	2 (1.9%)	1 (0.9%)	0 (0)	0 (0)	2 (2.7%)	1 (1.2%)	0 (0)	0 (0)
PR	67 (57.3%)	38 (29.2%)	64 (62.1%)	32 (28.3%)	3 (21.4%)	6 (35.2%)	49 (66.2%)	24 (28.6%)	15 (58.6%)	8 (27.6%)
SD	30 (25.6%)	47 (36.1%)	24 (23.3%)	42 (37.1%)	6 (42.9%)	6 (35.2%)	16 (21.6%)	32 (38.1%)	8 (27.6%)	10 (34.5%)
PD	17 (14.5%)	40 (30.8%)	12 (11.7%)	35 (31.0%)	5 (35.7%)	4 (23.5%)	8 (10.8%)	26 (31.0%)	4 (13.7%)	9 (31.0%)
Not assessable*∗*	1 (0.9%)	4 (3.1%)	1 (1.0%)	3 (2.7%)	0 (0)	1 (5.9%)	1 (1.4%)	1 (1.2%)	0 (0)	2 (6.9%)

ORR, %	59.0	30.0	64.1	29.2	21.4	35.3	68.9	29.8	51.7	27.6
OR	3.35		4.32		0.50		5.23		2.81	
95% CI	1.98-5.67		2.44-7.66		0.10-2.52		2.65-10.32		0.94-8.39	
p (Chi-square or Fisher's)	<0.001		<0.001		0.456		<0.001		0.060	
p for interaction test			0.014				0.344			

Radical resection rate of LM, %	28.2	10.0	31.1	9.7	7.3	11.8	35.1	10.7	20.7	6.9
OR	3.54		4.18		0.58		4.51		3.52	
95% CI	176-7.12		1.98-8.84		0.05-7.12		1.95-10.46		0.65-19.17	
p (Chi-square or Fisher's)	<0.001		<0.001		0.835		<0.001		0.128	

PFS, months										
Median	9.8	5.7	10.2	5.5	6.0	8.9	11.3	5.6	8.1	5.1
95%CI	8.9-11.1	4.1-5.9	9.3-10.7	4.1-5.9	1.3-6.8	5.0-9.0	9.9-12.1	3.9-6.1	5.2-8.8	2.2-5.8
HR	0.61		0.52		1.63		0.46		0.67	
95%CI	0.46-0.80		0.39-0.70		0.75-3.54		0.32-0.65		0.39-1.17	
p (log-rank)	<0.001		<0.001		0.166		<0.001		0.127	
p for interaction test			0.003				0.183			

OS, months										
Median	30.0	22.8	36.1	21.7	16.8	25.6	48.0	22.5	23.6	16.8
95%CI	18.9-39.1	18.3-25.7	24.6-43.4	18.0-24.0	11.2-20.8	15.1-32.9	-	18.1-23.9	21.7-26.3	4.5-23.5
HR	0.55		0.46		1.94		0.27		0.66	
95%CI	0.38-0.80		0.31-0.70		0.75-5.02		0.14-0.50		0.35-1.26	
p (log-rank)	0.001		<0.001		0.146		<0.001		0.200	
p for interaction test			0.006				0.036			

*∗* 3 early deaths (less than 3 months) and 2 lost to follow-up before the first time evaluation by MDT.

CR, complete response; PR, partial response; SD, stable disease; PD, progressive disease; ORR, objective response rate; LM, liver metastases; PFS, progression free survival; OS, overall survival; HR, hazard ratio; OR, odds ratio;95% CI, 95% confidence interval. MDT, multidisciplinary team; NE, not evaluable.

**Table 2:** Efficacy outcomes according to RAS status and predictive model in training cohort.

	RAS assessable (n=106)		RAS wild-type (n=93)		RAS wild-type/ Model-defined responsive group (n=66)		RAS wild-type/ Model-defined refractory group (n=27)	
	Cetuximab plus chemotherapy (n=50)	Chemotherapy alone (n=56)	Cetuximab plus chemotherapy (n=45)	Chemotherapy alone (n=48)	Cetuximab plus chemotherapy (n=29)	Chemotherapy alone (n=37)	Cetuximab plus chemotherapy (n=16)	Chemotherapy alone (n=11)
Overall response								
CR	1 (2.0%)	0 (0%)	1 (2.2%)	0 (0%)	1 (3.4%)	0 (0%)	0 (0%)	0 (0%)
PR	28 (56.0%)	17 (30.4%)	27 (60.0%)	14 (29.2%)	23 (79.3%)	11 (29.7%)	4 (25.0%)	3 (27.3%)
SD	14 (28.0%)	21 (37.5%)	12 (26.7%)	19 (39.6%)	3 (10.3%)	14 (37.8%)	9 (56.3%)	5 (45.5%)
PD	7 (14.0%)	15 (26.8%)	5 (11.1%)	13 (27.1%)	2 (6.9%)	10 (27.0%)	3 (18.8%)	3 (27.3%)
Not assessable*∗*	0 (0%)	3 (5.4%)	0 (0%)	2 (4.2%)	0 (0%)	2 (5.4%)	0 (0%)	0 (0%)

ORR, %	58.0	30.4	62.2	29.2	82.8	29.7	25.0	27.3
OR	3.17		4.00		11.35		0.889	
95% CI	1.42-7.05		1.68-9.51		3.44-37.44		0.155-5.084	
p (Chi-square or Fisher's)	0.004		0.001		<0.001		1.000	
p for interaction test					0.006			

Radical resection rate of LM, %	28.0	8.9	28.8	8.3	37.9	8.1	12.5	9.1
OR	3.97		4.47		6.93		1.43	
95% CI	1.31-12.0		1.33-14.98		1.71-28.05		0.11-18.04	
p (Chi-square or Fisher's)	0.011		0.010		0.003		1.000	

PFS, months								
Median	9.5	5.6	9.8	5.3	11.8	4.8	7.8	8.2
95%CI	8.7-11.3	3.8-6.2	9.1-10.8	3.9-6.1	8.1-13.9	3.9-6.1	5.2-8.8	3.8-8.3
HR	0.60		0.52		0.25		1.25	
95%CI	0.39-0.91		0.33-0.81		0.13-0.48		0.52-2.99	
p (log-rank)	0.010		0.002		<0.001		0.588	
p for interaction test					0.001			

OS, months								
Median	30.0	22.1	35.1	21.7	39.6	20.0	25.6	33.8
95%CI	15.3-42.6	17.0-25.0	21.4-44.6	17.5-24.5	25.6-48.4	12.9-27.1	18.1-29.9	11.2-48.8
HR	0.54		0.44		0.19		2.30	
95%CI	0.30-0.97		0.23-0.83		0.08-0.47		0.61-8.77	
p (log-rank)	0.034		0.009		<0.001		0.200	
p for interaction test					0.002			

*∗* 2 early deaths (less than 3 months) and 1 lost to follow-up before the first time evaluation by MDT.

CR, complete response; PR, partial response; SD, stable disease; PD, progressive disease; ORR, objective response rate; LM, liver metastases; PFS, progression free survival; OS, overall survival; HR, hazard ratio; OR, odds ratio; 95% CI, 95% confidence interval. MDT, multidisciplinary team; NE, not evaluable.

**Table 3:** Efficacy outcomes according to RAS status and predictive model in validation cohort.

	RAS assessable (n=141)		RAS wild-type (n=123)		RAS wild-type/ Model-defined responsive group (n=83)		RAS wild-type/ Model-defined refractory group (n=40)	
	Cetuximab plus chemotherapy (n=67)	Chemotherapy alone (n=74)	Cetuximab plus chemotherapy (n=58)	Chemotherapy alone (n=65)	Cetuximab plus chemotherapy (n=42)	Chemotherapy alone (n=41)	Cetuximab plus chemotherapy (n=16)	Chemotherapy alone (n=24)
Overall response								
CR	1 (1.5%)	1 (1.4%)	1 (1.7%)	1 (1.5%)	1 (2.4%)	1 (2.4%)	0 (0%)	0 (0%)
PR	39 (58.2%)	21 (28.4%)	37 (63.8%)	18 (27.7%)	33 (78.6%)	11 (26.8%)	4 (25.0%)	7 (29.2%)
SD	16 (23.9%)	26 (35.1%)	12 (20.7%)	23 (35.4%)	6 (14.2%)	14 (34.1%)	6 (37.5%)	9 (37.5%)
PD	10 (14.9%)	25 (33.8%)	7 (12.1%)	22 (33.8%)	2 (4.8%)	14 (34.1%)	5 (31.3%)	8 (33.3%)
Not assessable*∗*	1 (1.5%)	1 (1.4%)	1 (1.7%)	1 (1.5%)	1 (2.4%)	1 (2.2%)	0 (0%)	0 (0%)

ORR, %	59.7	29.7	65.5	29.2	81.0	29.3	25.0	29.2
OR	3.50		4.60		7.45		0.59	
95% CI	1.74-7.04		2.15-9.84		2.88-19.27		0.05-5.61	
P (Chi-square or Fisher's)	<0.001		<0.001		<0.001		1.000	
p for interaction test					0.005			

Radical resection rate of LM, %	28.4	10.8	32.8	10.8	40.5	9.8	12.5	12.5
OR	3.27		4.04		6.29		1.00	
95% CI	1.32-8.08		1.55-10.51		1.89-20.92		0.15-6.77	
p(Chi-square or Fisher's)	0.008		0.003		0.001		1.000	

PFS, months								
Median	10.0	5.8	10.5	5.6	11.3	4.7	8.6	7.4
95%CI	7.5-10.5	3.7-6.3	8.2-11.8	3.7-6.3	9.0-13.0	2.1-5.9	6.7-9.3	4.6-7.5
HR	0.63		0.54		0.45		0.83	
95%CI	0.44-0.91		0.37-0.80		0.28-0.74		0.44-1.59	
p(log-rank)	0.004		<0.001		0.001		0.534	
p for interaction test					0.094			

OS, months								
Median	31.7	23.7	36.8	21.6	42.0	20.3	24.8	23.0
95%CI	21.2-44.8	15.7-28.3	23.4-48.6	15.6-28.4	NE-NE	11.4-28.6	20.6-27.4	14.3-29.7
HR	0.54		0.45		0.27		0.92	
95%CI	0.33-0.89		0.26-0.77		0.13-0.58		0.41-2.06	
p(log-rank)	0.011		0.003		<0.001		0.828	
p for interaction test					0.020			

*∗* 1 early death (less than 3 months) and 1 lost to follow-up before the first time evaluation by MDT.

CR, complete response; PR, partial response; SD, stable disease; PD, progressive disease; ORR, objective response rate; LM, liver metastases; PFS, progression free survival; OS, overall survival; HR, hazard ratio; OR, odds ratio; 95% CI, 95% confidence interval. MDT, multidisciplinary team; NE, not evaluable.

## Data Availability

The data used to support the findings of this study are available from the corresponding author upon request.

## Supplementary Materials

Supplementary Materials The supplementary file include four parts: supplementary methods, supplementary results, supplementary figures, and supplementary tables. Supplementary methods part mainly describes detailed procedure of next-generation sequencing. Supplementary results part summarizes the data of whole exome sequencing. Supplementary figures part demonstrates the survival curves according to RAS mutations and predictive model (Fig S1-S3) and data of whole exome sequencing (Fig S4 and Fig S5). Supplementary tables part consists of summary of sequencing data (Tables S1-S7), efficacy results of in different subgroups (Tables S8, S11, and S13), and construction of predictive model (Tables S9, S10, and S12).
