# Supplementary material for: Additional Biomarkers beyond RAS That Impact the Efficacy of Cetuximab plus Chemotherapy in mCRC: A Retrospective Biomarker Analysis
Source: J Oncol. 2018 Sep 16;2018:5072987. doi: 10.1155/2018/5072987 (PMC6165607; doi:10.1155/2018/5072987)
Supplement: Supplementary Materials — The supplementary file include four parts: supplementary methods, supplementary results, supplementary figures, and supplementary tables. Supplementary methods part mainly describes detailed procedure of next-generation sequencing. Supplementary results part summarizes the data of whole exome sequencing. Supplementary figures part demonstrates the survival curves according to RAS mutations and predictive model (Fig S1-S3) and data of whole exome sequencing (Fig S4 and Fig S5). Supplementary tables part consists of summary of sequencing data (Tables S1-S7), efficacy results of in different subgroups (Tables S8, S11, and S13), and construction of predictive model (Tables S9, S10, and S12). [file 5072987.f1.pdf]

Supplementary Materials

Supplementary Methods 2

Tissue collection 2

Whole exome sequencing 2

Primary processing of WES data 2

Target capture sequencing 3

Supplementary Results 4

Summary of WES 4

Supplementary Figures 5

Figure S1 Kaplan-Meier curves according to RAS status 5

Figure S2 Kaplan-Meier curves according to model defined groups in training cohort. 6

Figure S3 Kaplan-Meier curves according to model defined groups in validation cohort 7

Figure S4 Workflow (A) and variant analysis (B) of WES data. 8

Figure S5 Summary of driver mutations identified by WES. 8

Supplementary Tables 9

Table S1. Clinical and pathologic characteristics of patients using for whole exome sequencing 9

Table S2. Summary of priamry assessing of whole exome sequencing data 10

Table S5. List of genes analyzed using target capture sequencing 12

Table S6. Summary of RAS mutations 13

Table S7. Summary of detected mutations of RAS, BRAF, PI3KCA and genes included in the model 14

Table S8. Efficacy outcomes according to PI3KCA and BRAF status in RAS wild-type patients 23

Table S9. Summary of the first-step procedure of selecting potential predictive biomarker 25

Table S10. Summary of the second-step procedure of selecting potential predictive biomarker 28

Table S11. Efficacy outcomes according to mutation status in RAS wild-type patients. 29

Table S12. Predictor value and coefficients in the predictive model 30

Table S13. Predictive model and primary tumor location 31

Table S2. Driver somatic mutations detected by WES 33

Table S3. Summary of searching results for metastases related genes in GeneRIF database 186

Supplementary references 242

SUPPLEMENTARY METHODS

Tissue collection

Fresh tissue was collected from colectomy specimens and metastatic nodules in the liver during surgery. Subsequently, the samples were frozen in liquid nitrogen and stored at -80 degrees centigrade. Formalin-fixed paraffin-embedded (FFPE) tissue was obtained from the Department of Pathology of Zhongshan Hospital (Shanghai, China). For sequencing, 6 μm frozen sections from fresh tissue and 10 μm sections from FFPE tissue were stained with hematoxylin and eosin. An experienced pathologist reviewed each section and indicated the area of the tumor. Macro-dissection was performed using the H&E-stained slides to enrich the number of tumor cells in each sample.

Whole exome sequencing

DNA was extracted from fresh tumor samples using the MELT™ Total Nucleic Acid Isolation Kit (Life Technologies). Quantity and quality were assessed using Qubit 2.0 (Life Technologies). 50-100ng of DNA for each sample was used for exome capture and library preparation with Ion AmpliSeq™ Exome Kit 4xDuo (Life Technologies) following manufacturer's instructions. Then libraries were bar-coded with Ion Xpress™ Barcode Adapters Kit (Life Technologies). The concentration of each library was determined by qPCR with the Ion Library™ Quantization Kit (Life Technologies). According to the manufacturer's instructions, all libraries were diluted to 100pM working solutions and then pooled as needed to perform the template preparation with Ion PI™ Template OT2 200 kit v2 (Life Technologies) on Ion One Touch™ 2 System. Quality and quantity were determined with Qubit Ion Sphere™ Quality Control Kit (Life Technologies) for the obtained Ion sphere particles (ISPs). Whole exome sequencing was performed on Ion Proton™ platform, using the Ion PI™ Sequencing 200 kit v2 and Ion PI™ Chip kit v2. Sequencing data was analyzed with the Torrent Suite™ Software v4.0 (Life Technologies) using default parameters setting.

Primary processing of WES data

The primary whole exome sequencing data were analyzed for single nucleotide variants (SNVs) and insertions and deletions (InDels) following the procedures indicated below. The work flow is shown in **Figure S4** and results were summarized in **Table S2, S3 and Figure S5**.

(1) Variants with SNV quality (QUAL) <=20 were excluded. QUAL was calculated using Torrent Suite™ software.

(2) Variants of normal mucosa were considered background variants. Primary or metastatic tumor samples were filtered using background variants, and the variants were rejected as germ-line variants or sequencing artifacts when present in the corresponding normal samples.

(3) Primary or metastatic tumor-specific SNVs were analyzed using the SeattleSeq SNP Annotation (http://snp.gs.washington.edu/SeattleSeqAnnotation138/). Known germ-line mutations from the Exome Sequencing Project (ESP) (http://evs.gs.washington.edu/EVS/) and dbSNP databases (build 140) (http://www.ncbi.nlm.nih.gov/projects/SNP/) were also excluded. We selected non-silent mutations, including missense mutations and InDels.

(4) Non-silent mutations were predicted to affect gene function when any of the following criteria were fulfilled: a. functional impact score of SIFT[1] <=0.05 (http://sift.jcvi.org/); b. functional impact score of PolyPhen-2[2] >0.45 (http://genetics.bwh.harvard.edu/pph2/); c. functional impact label of Mutation Assessor[3] was "medium" or "high" (http://mutationassessor.org/); d. Condel[4] label was "deleterious" (http://bg.upf.edu/fannsdb/); e. functional impact score of FATHMM[5] <0 (http://fathmm.biocompute.org.uk/).

(5) The transFIC analysis was performed as previously described[6]. Mutations were considered cancer driver mutations when the outcome of the transFIC analysis was "high impact".

Target capture sequencing

DNA was extracted from FFPE tumor samples using the RecoverAll™ Total Nucleic Acid Isolation Kit (Life Technologies) according to the manufacturer's instructions. Quantity and quality were assessed using Qubit 2.0 (Life Technologies). 10ng of DNA for each sample was used for library construction and template preparation with same procedures described above in the "whole exome sequencing" section. Target capture sequencing was carried out with customized panel using the Ion PGM™ platform (Life Technologies) according to the manufacturer’s instructions. The panel consisted of two separate PCR primer pools and produced a total of 1712 amplicons covering recurrent mutations in 100 genes with 1500X sequence coverage on Ion™ 318 chip. Sequencing data was analyzed with Ion Reporter™ software v4.4 (Life Technologies) using default parameters setting.

SUPPLEMENTARY RESULTS

Summary of WES

We performed WES for 10 triplets, each comprising tissue from the primary colorectal tumor, normal colorectal mucosa and matched liver metastases (**Table S1**). Overall, we identified 608 potential somatic driver mutations in 511 genes in primary colorectal tumors and 694 potential somatic driver mutations in 638 genes in liver metastases (**Table S3**). The number of somatic mutations in 10 primary colorectal tumor ranged from 41 to 87, with a mean of 60 (**Figure S5**), which was not significantly different from that of the non-hyper-mutated CRCs reported in The Cancer Genome Atlas (TCGA)[8]. Among these mutations, 10 and 278 were documented in the Catalog of Somatic Mutations in Cancer (COSMIC) and dbSNP databases (build 140), respectively. When comparing mutations between tumors, 230 mutations in 219 genes were universal in the primary tumor and corresponding liver metastases. In addition, mutations observed in 10 patients were predominated by the C/G>T/A transition (**Figure S5**), consistent with the results of previous CRC genomics studies [8, 9].

SUPPLEMENTARY FIGURES

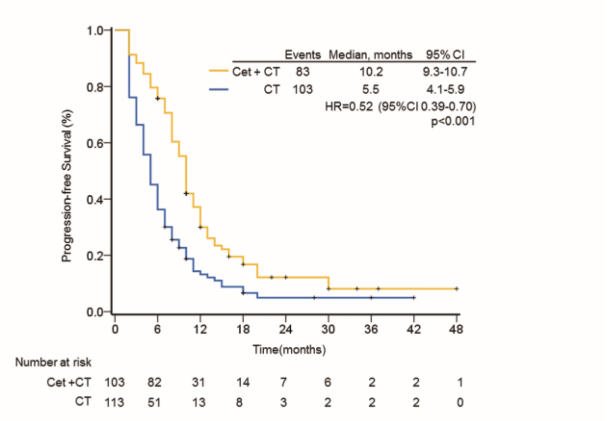

A

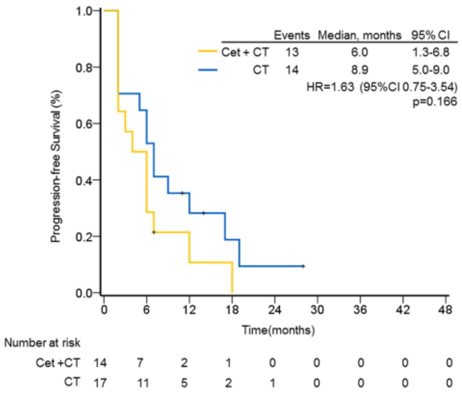

B

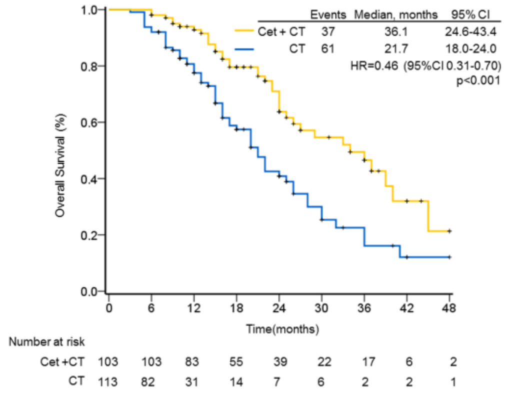

C

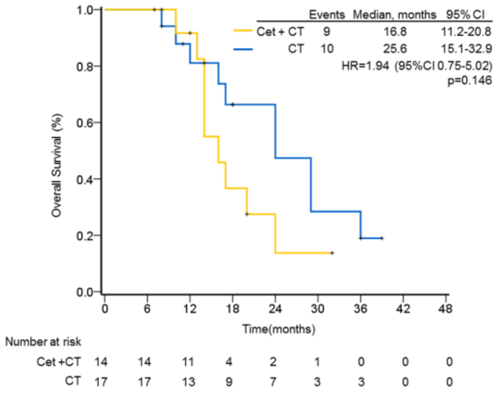

D

Figure S1 Kaplan-Meier curves according to RAS status. (A) PFS for patients with RAS mutations. (B) PFS for RAS assessable patients. (C) OS for patients with wild-type RAS. (D) OS for patients with RAS mutations.

Abbreviations: PFS, progression-free survival; OS, overall survival; cet, cetuximab; CT, chemotherapy; HR, hazard ratio.

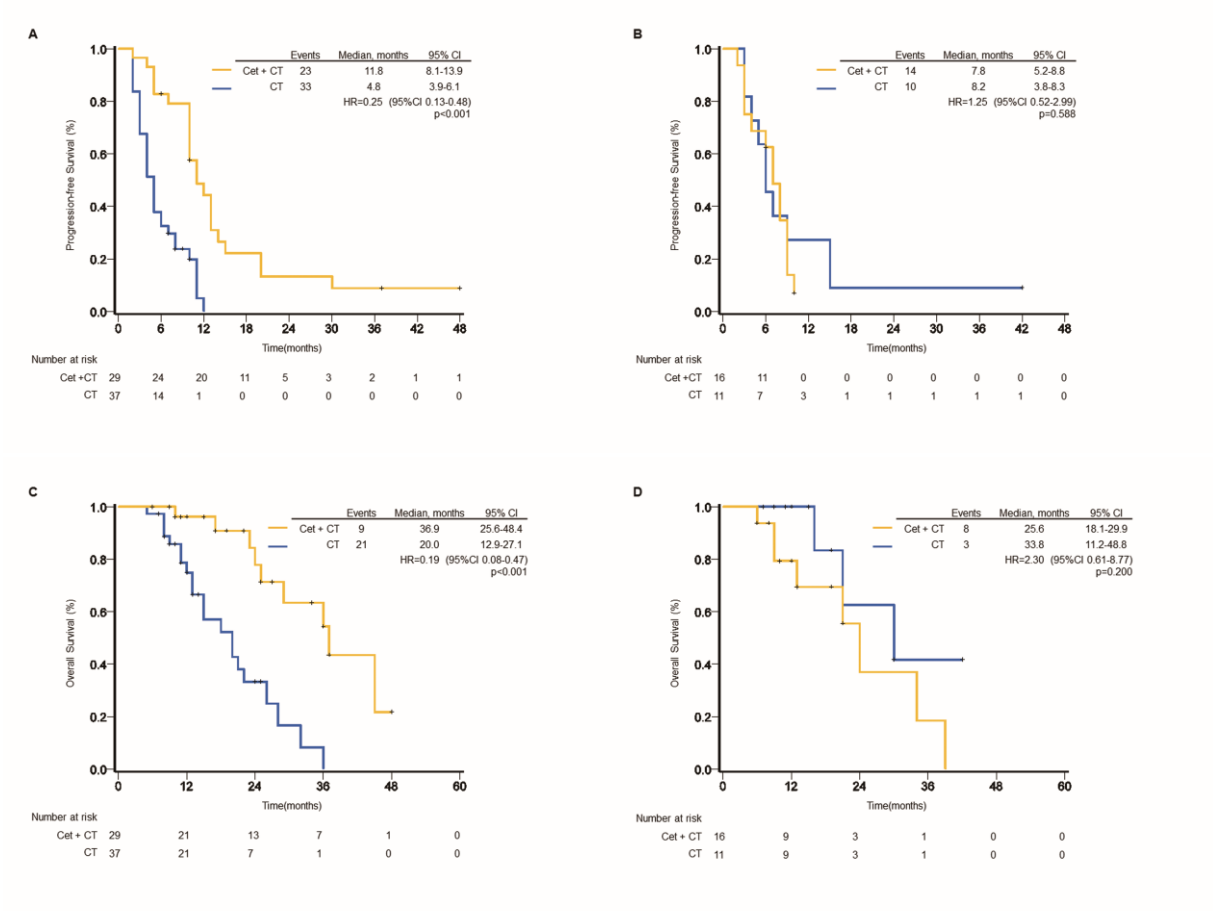

Figure S2 Kaplan-Meier curves according to model defined groups in training cohort.

(A) PFS for the responsive group. (B) PFS for the refractory group. (C) OS for the responsive group. (D) OS for the refractory group.

Abbreviations: PFS, progression-free survival; OS, overall survival; cet, cetuximab; CT, chemotherapy; HR, hazard ratio.

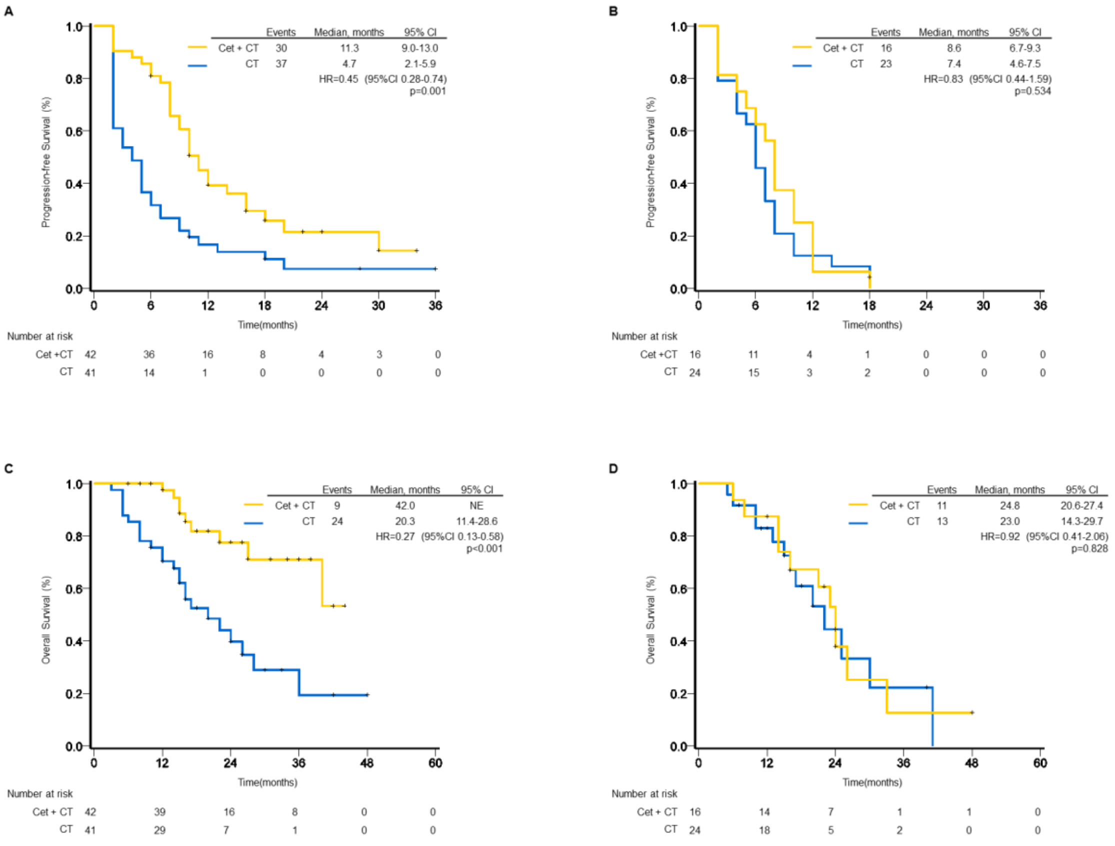

Figure S3 Kaplan-Meier curves according to model defined groups in validation cohort.

(A) PFS for the responsive group. (B) PFS for the refractory group.(C) OS for the responsive group.(D) OS for the refractory group.

Abbreviations: PFS, progression-free survival; OS, overall survival; cet, cetuximab; CT, chemotherapy; HR, hazard ratio.

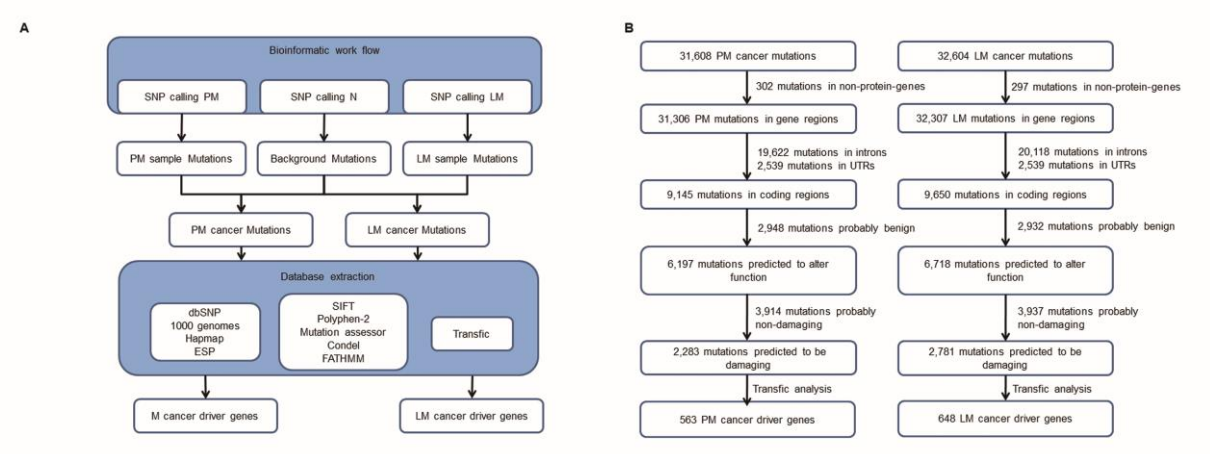

Figure S4 Workflow (A) and variant analysis (B) of WES data.

Abbreviations: PM, primary malignancy; LM, liver metastases

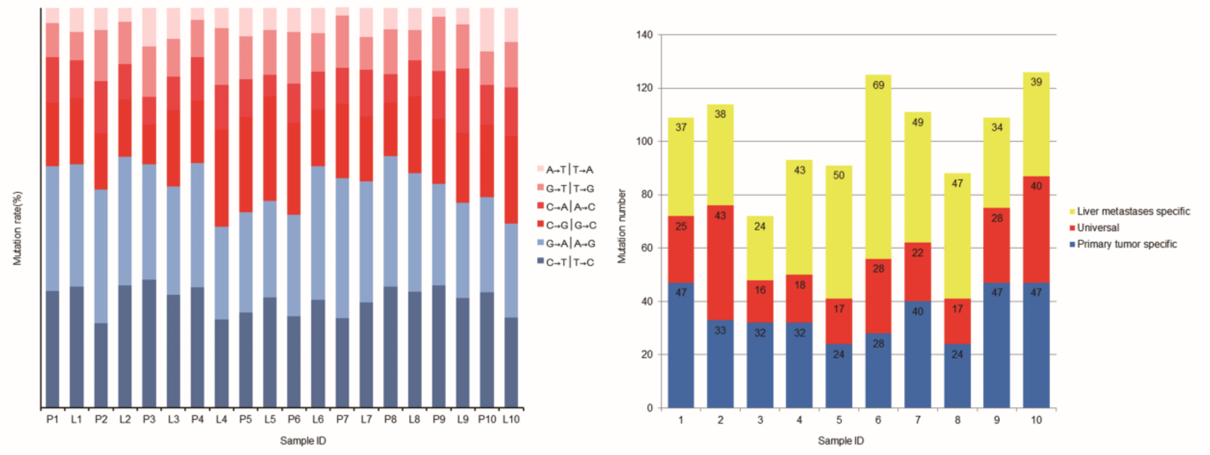

Figure S5 Summary of driver mutations identified by WES.

(A) Distribution of transitions and transversions. (B) Distribution of driver mutations in primary tumor and liver metastases.

Abbreviations: P, primary tumor; L, liver metastases.

SUPPLEMENTARY TABLES

Table S1. Clinical and pathologic characteristics of patients using for whole exome sequencing

Patient ID	Gender	Age, years	Primary tumor							Liver metastases		
			Location	Differentiation	T stage	N stage	Verineural invasion	Vascular invasion	Tumor deposits	Number	Maximum size, cm	Position
1	Male	48	Cecum	Grade III	4	2a	No	No	0	1	2.5	unilobar
2	Male	68	Sigmoid colon	Grade IV	4	1c	No	No	2	1	3	unilobar
3	Female	54	Hepatic flexure	Grade IV	4	1b	No	Yes	2	5	2.5	bilobar
4	Male	49	Rectum	Grade III	3	2b	No	No	5	2	3	unilobar
5	Male	69	Rectum	Grade III	4	1a	No	No	1	1	4.5	unilobar
6	Female	68	Ascending colon	Grade III	3	2b	Yes	Yes	0	4	5	bilobar
7	Male	63	Ascending colon	Grade II	3	0	No	No	0	1	2	unilobar
8	Female	56	Rectum	Grade III	4	1b	Yes	No	8	1	2	unilobar
9	Female	56	Transverse colon	Grade III	4	1b	No	Yes	1	2	1.5	bilobar
10	Female	61	Ascending colon	GradeIV	3	1b	No	No	0	1	1.5	unilobar

Table S2. Summary of priamry assessing of whole exome sequencing data

Patient ID	Sample type	Total number of SNVs	Average depth	> 20x cov.	Tumor-specific SNVs*	Non-silent SNVs **					Damaging SNVs***	Driver SNVs****
						Total	Missense	Frame-shift	Splice-site	Stop		
1	Primary tumor	49973	76	88.60%	4405	743	546	172	15	10	275	72
	Liver metastases	45818	67	84.90%	4256	759	530	211	12	6	250	62
	Normal mucosa	49738	71	88.20%	/	/	/	/	/	/	/	/
2	Primary tumor	47917	94	90.20%	4086	690	502	167	13	8	245	76
	Liver metastases	44228	82	86.80%	3668	713	513	179	10	11	262	81
	Normal mucosa	48866	89	89.80%	/	/	/	/	/	/	/	/
3	Primary tumor	51457	124	92.90%	3762	631	444	176	6	5	213	48
	Liver metastases	47771	67	83.70%	2900	487	316	159	8	4	173	40
	Normal mucosa	50923	121	92.00%	/	/	/	/	/	/	/	/
4	Primary tumor	49708	103	87.90%	4111	694	499	179	8	8	233	50
	Liver metastases	45583	70	91.10%	4159	715	505	181	17	12	250	61
	Normal mucosa	49588	105	80.70%	/	/	/	/	/	/	/	/
5	Primary tumor	49760	131	91.10%	3039	492	363	106	12	11	172	41
	Liver metastases	44183	73	80.70%	4251	937	457	447	16	17	261	67
	Normal mucosa	50453	133	92.50%	/	/	/	/	/	/	/	/
6	Primary tumor	46580	87	83.00%	4899	851	628	203	12	8	289	56
	Liver metastases	44549	99	84.50%	5354	960	753	176	16	16	369	97
	Normal mucosa	46271	107	84.00%	/	/	/	/	/	/	/	/

	Primary tumor	44512	86	80.60%	4749	845	590	229	15	11	267	62
7	Liver metastases	41831	74	79.90%	5105	906	675	195	20	16	312	71
	Normal mucosa	46457	82	82.40%	/	/	/	/	/	/	/	/
	Primary tumor	45208	76	81.10%	3796	639	396	217	19	7	195	41
8	Liver metastases	49155	85	88.50%	4316	810	562	225	17	6	282	64
	Normal mucosa	50877	78	89.50%	/	/	/	/	/	/	/	/
	Primary tumor	43981	71	77.80%	4284	820	644	143	15	18	337	75
9	Liver metastases	48210	87	85.30%	4236	740	550	160	14	16	287	62
	Normal mucosa	49746	81	86.50%	/	/	/	/	/	/	/	/
	Primary tumor	44710	88	84.70%	6827	1237	1025	176	24	12	486	87
10	Liver metastases	47004	81	81.10%	6458	1152	902	221	15	14	417	79
	Normal mucosa	45668	89	82.70%	/	/	/	/	/	/	/	/

* Variants present in normal samples were considered as germ-line events. Variants present in primary or metastatic tumor samples but not in corresponding normal sample were considered as primary or metastatic tumor-specific events.

** Synonymous variants and variants in intron and untranslated regions (UTR) were excluded. "Missense" included missense and missense-near-splice; "frame-shift" included frame-shift and frame-shift-near-splice; "splice-site" included splice-acceptor and splice-donor; "stop" included stop-lost, stop-lost-near-splice, stop-gained and stop-gained-near-splice.

*** predicted to possiably affect gene fuction by tools rank the functional impact of cancer somatic mutations (e.g. SIFT, Polyphen-2, Mutation assessor, Condel, FATHMM)

**** predicted to be cancer driver mutations by transFIC analysis

Table S5. List of genes analyzed using target capture sequencing

ABL1	CDKN2A	ERBB2	FGFR3	HRAS	LY6G6D	MMP2	PIK3CA	SERBP1	TBXAS1
ACTN4	COL1A1	ERBB4	FLT3	IDH1	MAGEC1	MPL	POMZP3	SMAD4	TCF3
AKT1	COL2A1	ESRRA	FN1	IDH2	MDC1	MUC16	POU5F1B	SMARCB1	TGFBR2
APC	CSF1R	EZH2	FSIP2	JAK2	MDN1	NCAM1	PTCH1	SMCR7	THBS1
ATAD3B	CTNNB1	FAM129A	GNA11	JAK3	MEGF6	NEK9	PTEN	SMO	TNXB
ATP6V1B1	CUL9	FBN3	GNAQ	KDR	MET	NOBOX	PTPN11	SRC	TP53
BRCA1	CXCR4	FBXW7	GNAS	KIAA0182	MICB	NOTCH1	PTPN23	SRPK3	TYK2
CARD10	DSE	FCGBP	HECA	KIT	MKI67	NPM1	RB1	STAB1	VHL
CD34	DST	FGFR1	HNF1A	LAMA4	MLH1	PDCD1LG2	RBMXL3	STK11	YSK4
CDH1	EGFR	FGFR2	HNRNPA0	LRRN4	MLL3	PDGFRA	RET	TAPBP	ZNF462

Table S6. Summary of RAS mutations

KRAS	3	59	175	G>A	A59T	COSM546
			176	C>A	A59E	COSM547
		61	182	A>G	Q61R	COSM552
			182	A>T	Q61L	COSM553
			183	A>C	Q61H	COSM554
			183	A>T	Q61H	COSM555
	4	117	351	A>C	K117N	COSM19940
			351	A>T	K117N	COSM28519
		146	436	G>A	A146T	COSM19404
			436	G>C	A146P	COSM19905
			437	C>T	A146V	COSM19900
NRAS	2	12	34	G>A	G12S	COSM563
			34	G>T	G12C	COSM562
			34	G>C	G12R	COSM561
			35	G>A	G12D	COSM564
			35	G>T	G12V	COSM566
			35	G>C	G12A	COSM565
		13	37	G>A	G13S	COSM571
			37	G>T	G13C	COSM570
			37	G>C	G13R	COSM569
			38	G>A	G13D	COSM573
			38	G>T	G13V	COSM574
			38	G>C	G13A	COSM575
	3	59	175	G>A	A59T	COSM578
		61	181	C>A	Q61K	COSM580
			181	C>G	Q61E	COSM581
			182	A>T	Q61L	COSM583
			182	A>G	Q61R	COSM584
			182	A>C	Q61P	COSM582
			183	A>T	Q61H	COSM585
			183	A>C	Q61H	COSM586
	4	146	436	G>A	A146T	COSM27174

Table S7. Summary of detected mutations of RAS, BRAF, PI3KCA and genes included in the model

Patient ID	Cohort	KRAS	NRAS	BRAF	ATP6V1B1		CUL9		ERBB2		LY6G6D		PTCH1		RBMXL3	
		Mutational exon	Mutational exon	Mutational exon	Amino acid change	Fraction of mutant alleles, %	Amino acid change	Fraction of mutant alleles, %	Amino acid change	Fraction of mutant alleles, %	Amino acid change	Fraction of mutant alleles, %	Amino acid change	Fraction of mutant alleles, %	Amino acid change	Fraction of mutant alleles, %
2	training	-	-	-	T30I	31.7	H1948P	40.4	I95V	44.3	-	-	-	-	-	-
3	training	-	-	Exon 15	-	-	H1948P	37.5	-	-	-	-	-	-	-	-
4	training	-	-	-	T30I	98.9	H1948P	98.5	-	-	-	-	-	-	-	-
5	training	-	-	-	-	-	H1948P	42.6	-	-	-	-	P1314L	96.9	D398N	52.5
6	training	-	-	-	T30I	30	H1948P	27.4	I95V	51.8	-	-	P1314L	95.6	D398N	61.4
7	training	-	-	-	T30I	100	H1948P	39	-	-	-	-	-	-	-	-
8	training	-	-	-	-	-	-	-	-	-	-	-	P1314L	52.6	D398N	57.7
9	training	-	-	-	-	-	H1948P	97.6	-	-	-	-	-	-	D398N	63.3
10	training	-	-	-	T30I	39.1	H1948P	30	-	-	-	-	P1314L	14.8	-	-
11	training	-	-	-	T30I	53.9	H1948P	20.7	-	-	-	-	P1314L	68.8	D398N	62.3
12	training	-	-	-	T30I	21.3	H1948P	26.9	I95V	21.3	R112C	20.2	P1314L	78.8	-	-
13	training	-	-	-	-	-	H1948P	47.9	-	-	-	-	P1314L	53.9	D398N	54.1
14	training	-	-	-	T30I	69.3	-	-	-	-	-	-	-	-	-	-
15	training	-	-	-	-	-	H1948P	66.7	-	-	-	-	-	-	-	-
16	training	Exon 4	-	-	T30I	100	H1948P	98.8	-	-	-	-	P1314L	98.2	-	-
18	training	Exon 3	-	-	-	-	-	-	-	-	-	-	-	-	D398N	61.5
19	training	-	-	-	-	-	H1948P	51.6	-	-	-	-	-	-	D398N	60.5
20	training	-	-	-	-	-	H1948P	100	-	-	-	-	P1314L	51.6	D398N	60.3
21	training	-	-	Exon 15	T30I	61.3	H1948P	53.7	I95V	35	-	-	P1314L	71.4	-	-
22	training	-	-	-	-	-	-	-	I95V	30	-	-	-	-	-	-
23	training	-	-	-	-	-	H1948P	43.4	-	-	-	-	P1314L	47.5	D398N	88.9
24	training	-	-	Exon 15	-	-	H1948P	100	-	-	-	-	P1314L	54.7	D398N	52
25	training	-	-	-	T30I	50.6	H1948P	7.6	-	-	R112C	39.1	P1314L	40.8	D398N	58.5

26	training	-	-	-	T30I	15.4	H1948P	47.8	-	-	-	-	P1314L	93.1	D398N	55.6
27	training	-	-	-	-	-	H1948P	52.5	I95V	46.6	-	-	P1314L	97.2	D398N	58.9
28	training	-	-	-	-	-	H1948P	100	-	-	-	-	P1314L	46.7	D398N	57.6
29	training	-	-	-	-	-	H1948P	84.2	-	-	R112C	41.8	P1314L	91.2	-	-
30	training	-	-	-	-	-	H1948P	44.5	-	-	-	-	-	-	D398N	51.6
31	training	-	-	-	T30I	45.1	-	-	-	-	R112C	100	-	-	D398N	47.8
32	training	-	-	-	-	-	H1948P	100	-	-	-	-	-	-	-	-
33	training	-	-	-	T30I	68.5	H1948P	50	-	-	-	-	P1314L	56.2	D398N	55.6
34	training	-	-	-	T30I	62.1	H1948P	53.8	-	-	-	-	P1314L	31	D398N	54.1
35	training	-	-	-	T30I	53.8	-	-	I95V	46.4	-	-	P1314L	40.3	-	-
36	training	-	-	-	-	-	-	-	-	-	-	-	-	-	-	-
37	training	-	-	-	-	-	-	-	I95V	45	-	-	-	-	D398N	58.3
38	training	-	-	-	-	-	-	-	-	-	-	-	P1314L	66.7	D398N	76.5
39	training	-	-	-	-	-	-	-	-	-	-	-	-	-	-	-
40	training	-	-	-	-	-	-	-	-	-	-	-	P1314L	81.9	D398N	9.1
41	training	-	-	Exon 15	T30I	53	-	-	-	-	R112C	52	P1314L	95.2	-	-
42	training	-	-	Exon 15	T30I	13	H1948P	41.7	-	-	-	-	P1314L	94.1	D398N	41.2
43	training	-	-	Exon 15	-	-	-	-	-	-	-	-	-	-	-	-
44	training	Exon 4	-	-	-	-	H1948P	41.3	I95V	69.9	-	-	-	-	-	-
45	training	-	-	-	T30I	64.6	H1948P	35.2	-	-	R112C	66.1	P1314L	79.2	-	-
46	training	-	-	-	T30I	73.5	H1948P	44.8	I95V	94.5	-	-	P1314L	95.3	D398N	66.8
47	training	-	-	-	-	-	H1948P	100	-	-	-	-	P1314L	97.2	D398N	55.3
48	training	-	-	-	-	-	-	-	-	-	R112C	41.1	-	-	D398N	50.6
49	training	-	-	-	T30I	47.7	-	-	-	-	R112C	45.8	P1314L	54.9	-	-
50	training	-	-	-	-	-	H1948P	50.4	I95V	44.3	R112C	41.7	P1314L	66.7	D398N	53.8
51	training	-	-	Exon 15	T30I	6.3	-	-	-	-	-	-	P1314L	54	D398N	64.2
52	training	-	-	-	T30I	59.5	H1948P	45.3	-	-	-	-	P1314L	44	D398N	60.3
53	training	Exon 4	-	-	T30I	10	-	-	I95V	40.5	-	-	P1314L	90.9	-	-
54	training	-	-	-	-	-	H1948P	54.8	-	-	-	-	P1314L	96.1	-	-
55	training	-	-	-	-	-	H1948P	43.1	-	-	-	-	-	-	D398N	60.8

56	training	-	-	-	T30I	52.5	H1948P	70.6	I95V	43.7	-	-	-	-	-	-
57	training	-	-	-	-	-	H1948P	100	-	-	-	-	P1314L	45.5	D398N	52.7
58	training	-	-	-	-	-	H1948P	47.6	-	-	-	-	P1314L	37.3	-	-
59	training	-	-	-	-	-	H1948P	44.7	-	-	-	-	P1314L	56.6	D398N	57.8
60	training	-	-	-	-	-	-	-	-	-	-	-	P1314L	97.6	-	-
61	training	-	-	-	T30I	100	H1948P	100	-	-	-	-	P1314L	96.4	D398N	61.8
62	training	-	-	-	-	-	-	-	-	-	-	-	P1314L	67.7	-	-
63	training	-	-	-	T30I	62.7	H1948P	55.5	-	-	R112C	50	P1314L	97.5	-	-
64	training	-	-	Exon 15	-	-	H1948P	98.3	I95V	35.3	-	-	-	-	D398N	58.8
65	training	-	Exon 2	-	T30I	67	H1948P	59.8	-	-	R112C	32.8	-	-	D398N	50.3
66	training	Exon 3	-	-	-	-	H1948P	48.7	-	-	R112C	52.1	P1314L	97.6	-	-
67	training	-	Exon 3	-	-	-	-	-	-	-	R112C	55.5	P1314L	93	-	-
68	training	-	-	-	-	-	H1948P	87.9	-	-	-	-	P1314L	69	-	-
69	training	-	-	Exon 15	-	-	-	-	-	-	-	-	-	-	-	-
70	training	-	-	-	T30I	13.1	-	-	-	-	-	-	P1314L	12.8	-	-
71	training	-	Exon 4	-	-	-	H1948P	36.4	-	-	-	-	-	-	-	-
72	training	Exon 3	-	-	-	-	H1948P	88.9	-	-	-	-	P1314L	100	-	-
73	training	-	-	-	-	-	H1948P	42.9	-	-	-	-	P1314L	97	D398N	55.3
74	training	-	Exon 3	-	-	-	H1948P	100	-	-	-	-	P1314L	100	D398N	37.5
75	training	-	-	-	T30I	66.9	H1948P	94	I95V	47.6	-	-	P1314L	97.2	-	-
76	training	-	-	-	-	-	-	-	-	-	-	-	-	-	-	-
77	training	-	-	-	-	-	-	-	I95V	48.1	R112C	31.3	-	-	D398N	52.1
78	training	-	-	Exon 15	-	-	-	-	-	-	-	-	-	-	-	-
79	training	-	-	-	-	-	H1948P	44.1	I95V	69.7	-	-	P1314L	87.2	-	-
80	training	-	-	-	-	-	H1948P	52	-	-	R112C	46.9	P1314L	33.3	-	-
81	training	-	-	-	-	-	-	-	I95V	85	-	-	P1314L	97.5	-	-
82	training	-	-	-	T30I	51	H1948P	56.6	-	-	R112C	55	P1314L	94.1	D398N	62.9
83	training	-	-	-	T30I	15.8	-	-	-	-	R112C	30.1	P1314L	92.6	D398N	57.4
84	training	-	-	Exon 15	-	-	H1948P	8.2	-	-	-	-	-	-	-	-
85	training	-	-	Exon 15	-	-	H1948P	72	-	-	-	-	P1314L	53.1	-	-

86	training	-	-	-	-	-	-	-	-	-	-	-	-	-	-	-
87	training	-	Exon 3	-	-	-	H1948P	100	I95V	48.3	R112C	87.9	-	-	D398N	56.7
88	training	-	-	-	-	-	H1948P	100	-	-	-	-	P1314L	52.8	-	-
89	training	-	Exon 2	-	T30I	69.9	H1948P	49.2	-	-	-	-	P1314L	97.5	D398N	60.4
90	training	-	-	Exon 15	T30I	47.9	H1948P	100	I95V	32.8	-	-	-	-	D398N	60.8
91	training	-	-	-	-	-	H1948P	60.3	-	-	-	-	P1314L	49.7	D398N	55.7
92	training	-	-	-	-	-	H1948P	41.3	-	-	-	-	P1314L	27.4	-	-
93	training	-	-	-	T30I	48.8	H1948P	48.3	-	-	-	-	P1314L	97.3	D398N	62.5
94	training	-	-	-	-	-	H1948P	34.1	-	-	R112C	44	P1314L	68.4	D398N	60.9
95	training	-	-	-	-	-	-	-	-	-	-	-	-	-	-	-
97	training	-	-	-	T30I	6.2	H1948P	58.2	I95V	54.7	R112C	43.1	P1314L	67	D398N	55.7
98	training	-	-	-	-	-	H1948P	50	-	-	-	-	-	-	-	-
99	training	-	-	-	-	-	-	-	-	-	R112C	42.5	-	-	D398N	43.3
100	training	-	-	-	T30I	50.2	-	-	I95V	41.3	-	-	P1314L	98.2	D398N	56.2
101	training	-	-	-	T30I	52.6	H1948P	45	-	-	R112C	95.1	P1314L	37.3	-	-
102	training	-	-	-	-	-	H1948P	100	-	-	-	-	-	-	D398N	70.9
103	training	-	-	-	T30I	50.6	H1948P	40.9	I95V	41.5	-	-	P1314L	97.8	-	-
104	training	Exon 3	-	-	T30I	36.7	H1948P	40.5	-	-	-	-	P1314L	97.8	D398N	60.9
105	training	-	-	Exon 15	T30I	53.5	H1948P	5.4	-	-	-	-	P1314L	51.2	D398N	59
106	training	-	-	-	T30I	8.3	-	-	-	-	R112C	33	-	-	D398N	59.6
107	training	-	-	-	-	-	-	-	-	-	-	-	-	-	D398N	61.2
108	training	-	-	-	T30I	54.4	-	-	I95V	40.8	-	-	-	-	D398N	55
109	training	-	-	-	-	-	H1948P	68.7	-	-	-	-	P1314L	78.6	D398N	33.3
401	vailidation	-	-	-	T30I	68.2	H1948P	25.1	-	-	R112C	24.3	P1314L	96.2	D398N	86.9
402	vailidation	-	-	Exon 15	T30I	43.1	H1948P	45.6	-	-	-	-	P1314L	64.8	D398N	90
403	vailidation	-	-	-	-	-	H1948P	56.7	-	-	-	-	P1314L	41.3	-	-
404	vailidation	-	-	-	T30I	38.8	H1948P	92.1	-	-	-	-	P1314L	33.3	D398N	68.9
405	vailidation	-	-	-	-	-	H1948P	39.1	-	-	R112C	53.2	P1314L	49.8	D398N	89.5
407	vailidation	-	-	-	-	-	H1948P	27.8	-	-	R112C	93.8	P1314L	61.9	D398N	79.6
408	vailidation	-	-	-	T30I	47.9	H1948P	59.2	-	-	-	-	P1314L	100	D398N	91.8

410	vailidation	-	-	-	-	-	H1948P	53.6	-	-	-	-	P1314L	59.7	D398N	90.7
411	vailidation	-	-	-	-	-	H1948P	56.1	-	-	R112C	49.1	P1314L	61.7	D398N	91.8
412	vailidation	-	-	-	T30I	62.1	H1948P	100	-	-	-	-	P1314L	96.8	D398N	81.2
413	vailidation	-	-	-	-	-	-	-	I95V	67.6	-	-	-	-	D398N	89.7
414	vailidation	-	-	Exon 15	T30I	34.3	H1948P	100	-	-	-	-	P1314L	45.9	D398N	77
415	vailidation	-	-	-	T30I	41.4	H1948P	63.6	-	-	-	-	P1314L	97.1	-	-
416	vailidation	Exon 3	-	-	-	-	-	-	-	-	R112C	48.9	P1314L	58.4	D398N	91.4
417	vailidation	Exon 3	-	-	-	-	-	-	-	-	-	-	-	-	-	-
418	vailidation	-	-	-	T30I	62	H1948P	46.3	-	-	-	-	-	-	D398N	93.1
419	vailidation	-	-	-	T30I	63.6	-	-	I95V	34.4	-	-	P1314L	66.1	D398N	65.2
420	vailidation	-	-	-	-	-	H1948P	58.8	I95V	98.2	-	-	P1314L	100	D398N	81.7
421	vailidation	-	-	-	T30I	50.3	-	-	-	-	R112C	41.4	P1314L	99.5	D398N	72.3
422	vailidation	-	-	-	T30I	42.4	H1948P	52.3	-	-	-	-	P1314L	100	D398N	89.7
423	vailidation	Exon 4	-	-	T30I	50	H1948P	41.5	-	-	-	-	-	-	D398N	74.5
424	vailidation	-	-	-	-	-	H1948P	98.2	-	-	-	-	P1314L	100	D398N	91.8
425	vailidation	-	-	-	T30I	61.3	-	-	-	-	-	-	-	-	D398N	89.8
426	vailidation	-	-	-	-	-	H1948P	60.5	-	-	-	-	P1314L	36.7	D398N	100
427	vailidation	-	-	-	-	-	H1948P	98.7	-	-	R112C	53.3	P1314L	96.7	D398N	89.1
428	vailidation	-	-	-	-	-	-	-	I95V	15.3	-	-	P1314L	91.5	D398N	80.9
429	vailidation	-	-	-	T30I	52.1	H1948P	43.3	I95V	48.7	R112C	7.8	P1314L	98.1	D398N	92.9
430	vailidation	-	-	Exon 15	-	-	-	-	-	-	-	-	-	-	-	-
431	vailidation	-	-	-	-	-	H1948P	100	I95V	71.8	R112C	20.9	P1314L	65	D398N	95.8
432	vailidation	Exon 4	-	-	-	-	H1948P	55.4	I95V	53.5	-	-	P1314L	98.3	D398N	86
433	vailidation	Exon 4	-	-	-	-	H1948P	47.5	-	-	R112C	50.1	P1314L	58.5	D398N	87.1
434	vailidation	-	-	-	-	-	H1948P	39.8	-	-	-	-	P1314L	38.6	D398N	8.9
435	vailidation	-	-	-	-	-	-	-	I95V	18.5	R112C	38	P1314L	98.2	D398N	12.7
436	vailidation	-	-	-	T30I	44	H1948P	38	-	-	-	-	P1314L	58.7	D398N	87.8
437	vailidation	-	-	-	-	-	-	-	-	-	R112C	32.4	P1314L	52.1	D398N	93.9
438	vailidation	-	-	-	-	-	-	-	I95V	95.1	R112C	53.2	P1314L	61.8	D398N	78.3
439	vailidation	-	Exon 3	-	T30I	100	H1948P	13.5	-	-	-	-	P1314L	47.5	D398N	95.6

440	vailidation	-	-	-	T30I	45.2	H1948P	59.8	-	-	-	-	-	-	D398N	87
441	vailidation	-	-	-	-	-	H1948P	35.2	I95V	44.6	-	-	P1314L	51.5	D398N	86.4
442	vailidation	-	-	-	T30I	58.4	H1948P	46	-	-	R112C	46.5	P1314L	41.5	D398N	93.9
443	vailidation	-	-	-	-	-	-	-	-	-	-	-	-	-	D398N	92.6
444	vailidation	-	-	-	T30I	49.6	H1948P	98.7	-	-	R112C	35.4	-	-	D398N	100
445	vailidation	-	-	-	-	-	H1948P	16.2	-	-	-	-	P1314L	70.3	D398N	100
446	vailidation	-	-	-	-	-	-	-	I95V	53.8	-	-	P1314L	42.5	D398N	18.2
447	vailidation	-	-	-	-	-	-	-	I95V	62.8	-	-	P1314L	95	D398N	89.2
448	vailidation	-	-	-	-	-	H1948P	57.5	-	-	-	-	P1314L	49.1	D398N	84.3
449	vailidation	-	-	-	-	-	H1948P	100	-	-	-	-	P1314L	41.6	D398N	89.1
450	vailidation	Exon 4	-	-	-	-	H1948P	94.3	-	-	R112C	7.1	-	-	-	-
451	vailidation	-	-	-	T30I	36.7	H1948P	46	I95V	5.3	-	-	P1314L	91.8	D398N	83.1
453	vailidation	-	-	-	T30I	46.9	H1948P	31.8	I95V	20.2	-	-	P1314L	14.2	D398N	84.9
454	vailidation	-	-	-	-	-	H1948P	47.3	-	-	-	-	-	-	D398N	88.5
455	vailidation	-	-	-	-	-	H1948P	40.8	I95V	100	-	-	P1314L	100	D398N	95.7
456	vailidation	-	-	-	-	-	H1948P	95.9	-	-	-	-	P1314L	68.4	D398N	69.4
457	vailidation	-	-	-	T30I	52.4	H1948P	46.2	-	-	-	-	P1314L	39.5	D398N	85.5
458	vailidation	-	-	-	-	-	-	-	-	-	-	-	-	-	D398N	82.1
459	vailidation	-	-	-	-	-	H1948P	95.7	-	-	-	-	P1314L	55.5	D398N	80.4
460	vailidation	-	-	Exon 15	-	-	-	-	-	-	-	-	P1314L	94.9	D398N	76.1
461	vailidation	-	-	-	-	-	H1948P	55.8	I95V	51.2	-	-	-	-	D398N	91.7
462	vailidation	-	-	-	T30I	50.8	H1948P	98.1	-	-	-	-	P1314L	59.4	D398N	89.8
463	vailidation	-	-	-	T30I	61	-	-	-	-	-	-	P1314L	95.2	D398N	81.8
464	vailidation	-	-	-	-	-	-	-	I95V	28	-	-	-	-	-	-
465	vailidation	-	-	-	-	-	-	-	-	-	-	-	-	-	-	-
466	vailidation	Exon 3	-	-	-	-	H1948P	52.9	I95V	11.9	-	-	-	-	D398N	87.5
467	vailidation	-	Exon 3	-	-	-	H1948P	100	-	-	R112C	37.9	P1314L	47.6	D398N	100
468	vailidation	Exon 4	-	-	-	-	-	-	-	-	-	-	-	-	-	-
469	vailidation	-	-	-	-	-	H1948P	4.5	-	-	-	-	P1314L	37.2	D398N	92.9
470	vailidation	-	-	-	-	-	-	-	-	-	-	-	-	-	-	-

471	vailidation	-	Exon 3	-	-	-	H1948P	98.9	-	-	-	-	P1314L	29.1	D398N	98.4
472	vailidation	-	-	-	-	-	H1948P	98	-	-	-	-	-	-	D398N	94.6
473	vailidation	-	-	-	T30I	64.9	H1948P	44.9	I95V	51.3	-	-	P1314L	97.6	D398N	96.7
474	vailidation	-	-	-	T30I	50	H1948P	47.6	-	-	-	-	-	-	D398N	83.8
475	vailidation	Exon 3	-	-	-	-	H1948P	64.1	I95V	13.1	-	-	P1314L	82.5	D398N	88.5
476	vailidation	-	-	Exon 15	T30I	51.5	H1948P	46.9	-	-	-	-	P1314L	100	D398N	88.9
477	vailidation	-	Exon 2	-	T30I	100	-	-	-	-	-	-	P1314L	95.2	D398N	91.9
478	vailidation	-	-	-	-	-	H1948P	51.7	-	-	-	-	-	-	D398N	95.3
479	vailidation	-	Exon 3	-	-	-	H1948P	100	-	-	-	-	P1314L	97	D398N	92.5
480	vailidation	-	-	-	T30I	12.1	H1948P	4.1	-	-	R112C	7.4	P1314L	33.3	D398N	98.9
481	vailidation	-	-	-	T30I	23.4	H1948P	51.6	-	-	-	-	P1314L	46	D398N	97.5
482	vailidation	-	-	-	T30I	13	H1948P	12	-	-	-	-	P1314L	83	D398N	84.1
483	vailidation	-	-	-	T30I	30.8	H1948P	6.8	-	-	R112C	55.5	P1314L	7.3	D398N	89
484	vailidation	-	-	-	T30I	57.7	H1948P	53	-	-	-	-	P1314L	70.5	D398N	93.5
485	vailidation	-	-	-	T30I	45.1	H1948P	60	-	-	-	-	P1314L	30.5	D398N	58.6
486	vailidation	-	-	Exon 15	T30I	42	H1948P	8.9	-	-	-	-	P1314L	70.6	-	-
487	vailidation	-	-	-	-	-	-	-	I95V	57.1	-	-	-	-	-	-
488	vailidation	-	-	-	-	-	H1948P	50.6	I95V	46.6	-	-	-	-	D398N	100
489	vailidation	-	-	-	T30I	89.5	H1948P	55.3	I95V	50.2	-	-	P1314L	95.8	D398N	96.5
490	vailidation	-	-	-	-	-	H1948P	45.5	-	-	-	-	-	-	-	-
491	vailidation	-	-	-	-	-	-	-	-	-	-	-	-	-	-	-
492	vailidation	-	-	-	-	-	H1948P	58	-	-	-	-	P1314L	13	-	-
493	vailidation	-	-	-	T30I	92.6	H1948P	50.2	-	-	-	-	P1314L	95.6	D398N	92.1
494	vailidation	-	-	-	T30I	63.3	H1948P	99.2	-	-	-	-	P1314L	97.6	D398N	93.2
495	vailidation	-	-	-	-	-	H1948P	38.5	-	-	-	-	P1314L	48.9	D398N	95
496	vailidation	-	-	-	-	-	H1948P	100	-	-	-	-	P1314L	97.1	D398N	93.1
497	vailidation	-	-	-	T30I	57.6	H1948P	48.6	-	-	-	-	P1314L	93.5	D398N	90.9
498	vailidation	Exon 3	-	-	-	-	H1948P	50.5	I95V	28.5	-	-	-	-	D398N	87.9
499	vailidation	-	-	-	-	-	H1948P	41.7	-	-	-	-	P1314L	50.4	D398N	95.8
500	vailidation	-	-	-	-	-	-	-	I95V	22.7	-	-	-	-	D398N	95.5

501	vailidation	-	-	-	-	-	H1948P	91.2	I95V	93.2	-	-	P1314L	51	D398N	91.3
502	vailidation	-	-	-	-	-	-	-	-	-	-	-	-	-	-	-
503	vailidation	-	-	-	-	-	-	-	-	-	-	-	-	-	-	-
504	vailidation	-	-	-	-	-	-	-	-	-	-	-	-	-	-	-
505	vailidation	-	-	Exon 15	-	-	-	-	I95V	52	R112C	43.6	P1314L	95.1	D398N	94.9
506	vailidation	-	-	-	-	-	H1948P	58.3	-	-	-	-	P1314L	34.1	D398N	99.2
507	vailidation	-	-	-	-	-	-	-	I95V	46.3	-	-	-	-	-	-
508	vailidation	Exon 3	-	-	-	-	H1948P	57.7	-	-	-	-	P1314L	97.5	D398N	97.5
509	vailidation	-	-	-	-	-	H1948P	98.6	I95V	100	-	-	P1314L	97.2	D398N	96.5
510	vailidation	-	-	-	T30I	38	H1948P	47.5	-	-	R112C	47.4	P1314L	58.1	D398N	95.1
511	vailidation	-	-	-	T30I	69.2	-	-	-	-	-	-	P1314L	40.9	D398N	100
512	vailidation	-	-	-	T30I	90.3	H1948P	46.6	I95V	59.6	-	-	P1314L	97.6	D398N	90.3
513	vailidation	-	-	-	-	-	H1948P	46.7	-	-	-	-	P1314L	47.3	D398N	98
514	vailidation	-	-	-	T30I	78.5	H1948P	38	-	-	-	-	P1314L	62.3	D398N	89.8
515	vailidation	-	-	-	T30I	47.9	-	-	-	-	-	-	-	-	D398N	86.8
516	vailidation	-	-	-	-	-	H1948P	53.5	-	-	-	-	P1314L	98.4	D398N	91.3
517	vailidation	-	-	-	T30I	8.7	H1948P	43.8	-	-	-	-	P1314L	54.9	D398N	93.6
518	vailidation	Exon 3	-	-	T30I	21.4	H1948P	7.9	-	-	R112C	9.3	P1314L	54.6	D398N	93.5
519	vailidation	-	-	-	-	-	H1948P	25.1	-	-	-	-	-	-	D398N	96
520	vailidation	-	-	-	T30I	71.3	H1948P	100	-	-	-	-	P1314L	55.9	D398N	90.3
521	vailidation	-	-	-	-	-	H1948P	42.4	I95V	100	-	-	P1314L	47.6	D398N	95.7
522	vailidation	-	-	-	-	-	H1948P	46.7	-	-	-	-	P1314L	5.9	D398N	95.2
523	vailidation	-	-	-	T30I	52.1	H1948P	93	-	-	R112C	51.4	P1314L	65.3	D398N	95.4
524	vailidation	-	-	-	-	-	-	-	-	-	-	-	-	-	-	-
525	vailidation	-	-	-	-	-	-	-	-	-	-	-	-	-	-	-
526	vailidation	-	-	-	-	-	-	-	-	-	-	-	-	-	-	-
527	vailidation	-	-	-	T30I	70.9	-	-	-	-	-	-	P1314L	45.1	D398N	94.1
528	vailidation	-	-	-	T30I	26.6	H1948P	36.3	I95V	59.3	-	-	P1314L	91.9	-	-
529	vailidation	-	-	-	-	-	H1948P	44.8	I95V	53.9	-	-	P1314L	97.5	D398N	87.7
530	vailidation	-	-	-	-	-	H1948P	100	-	-	-	-	P1314L	96.6	D398N	87.4

531	vailidation	-	-	-	T30I	41.1	-	-	-	-	R112C	47.1	P1314L	55.5	D398N	95.5
532	vailidation	Exon 4	-	Exon 15	-	-	-	-	-	-	-	-	P1314L	62.5	D398N	92
533	vailidation	-	-	-	-	-	H1948P	66.2	-	-	-	-	P1314L	97	D398N	81.9
534	vailidation	-	-	-	-	-	H1948P	50.6	-	-	R112C	10.2	-	-	D398N	16.9
535	vailidation	-	-	-	-	-	-	-	I95V	48.7	-	-	-	-	-	-
536	vailidation	-	-	-	-	-	-	-	-	-	-	-	-	-	D398N	86.9
537	vailidation	-	-	Exon 15	-	-	-	-	-	-	-	-	-	-	-	-
538	vailidation	-	-	-	-	-	H1948P	100	-	-	-	-	P1314L	36.3	D398N	85.7
539	vailidation	-	-	-	-	-	H1948P	73.4	-	-	-	-	P1314L	47.6	D398N	77.6
540	vailidation	-	-	-	T30I	63.6	H1948P	19	I95V	52.2	-	-	-	-	-	-
541	vailidation	-	-	Exon 15	T30I	15	H1948P	12.5	I95V	25	-	-	P1314L	90.5	-	-
542	vailidation	-	-	Exon 15	-	-	-	-	I95V	59	-	-	-	-	-	-
543	vailidation	-	-	-	-	-	-	-	-	-	R112C	49.1	-	-	D398N	93.1
544	vailidation	-	-	-	T30I	45.8	-	-	I95V	57.4	-	-	P1314L	92.9	D398N	90.5

Patient 96, 406 and 409 with insufficient quality of sequencing library and patients 1, 17 and 452 with insufficient amount of DNA extracted.

KRAS, NRAS and BRAF mutations were tested by ARMS. Several mutations shared the same PCR tube and are not distinguished respectively (Supplementary Table 5). Other mutations were tested by NGS.

Table S8. Efficacy outcomes according to PI3KCA and BRAF status in RAS wild-type patients

	RAS wild-type		RAS/BRAF wild-type		RAS wild-type/BRAF mutant		RAS/PI3KCA wild-type		RAS/BRAF/PI3KCA treble wild-type	
	Cetuximab plus chemotherapy (n=45)	Chemotherapy alone (n=48)	Cetuximab plus chemotherapy (n=38)	Chemotherapy alone (n=41)	Cetuximab plus chemotherapy (n=7)	Chemotherapy alone (n=7)	Cetuximab plus chemotherapy (n=38)	Chemotherapy alone (n=44)	Cetuximab plus chemotherapy (n=31)	Chemotherapy alone (n=37)
ORR, %	62.2	29.2	63.2	31.7	57.1	14.3	60.5	27.3	61.3	29.7
Odd ratios	4.00		3.69		8.00		4.09		3.74	
95%CI	1.68-9.51		1.46-9.37		0.60-107.0		1.62-10.35		1.36-10.27	
P value (chi-square)	*0.002*		*0.005*		*0.266*		*0.002*		*0.009*	
P value for interaction test	-		*0.582**				*0.723***		*0.807****	
Resection rate of LM, %	28.8	8.3	28.9	9.8	28.6	0.0	31.6	9.1	32.3	10.8
Odd ratios	4.47		3.69		8.00		4.62		3.93	
95%CI	1.33-14.98		1.46-9.37		0.60-106.9		1.34-15.86		1.09-14.16	
P value (chi-square)	*0.01*		*0.03*		*0.127*		*0.010*		*0.029*	
PFS, months										
Median	9.8	5.3	9.7	5.6	12.1	4.2	9.8	5.5	9.7	5.8
Hazard ratios	0.52		0.57		0.21		0.54		0.60	
95%CI	0.33-0.81		0.35-0.91		0.04-1.07		0.34-0.88		0.36-1.01	
P value (log-rank)	*0.002*		*0.011*		*0.025*		*0.007*		*0.036*	
P value for interaction test	-		*0.142**				*0.376***		*0.080****	
OS, months										
Median	35.1	21.7	37.1	21.8	24.5	20.0	36.8	21.8	38.1	22.0
Hazard ratios	0.44		0.40		0.78		0.42		0.37	

95%CI	0.23-0.83	0.20-0.83	0.15-4.00	0.21-0.84	0.17-0.83
P value (log-rank)	*0.009*	*0.010*	*0.700*	*0.011*	*0.011*
P value for interaction test	-	*0.440**		*0.728***	*0.656****

NE, not evaluable

* P values for the interaction between BRAF status and treatment

** P values for the interaction between PI3KCA status and treatment.

*** P values for the interaction between combination of BRAF and PI3CA (double wild-type vs. any mutant) and treatment (cetuximab + chemotherapy vs. chemotherapy)

Table S9. Summary of the first-step procedure of selecting potential predictive biomarker

Gene ID	Codon*	Mutation number	Mutation rate**, %	P value of interaction test for ORR
ABL1	578	13	12.3	NS
	All	34	32.1	NS
ACTN4	400	60	56.6	NS
	All	81	76.4	NS
AKT1	All	14	13.2	NS
ATAD3B	386	82	77.4	NS
	579	38	35.8	NS
	All	87	82.1	NS
ATP6V1B1	30	43	40.6	0.054
BRAC1	1183	61	57.5	NS
	871	61	57.5	NS
	All	76	71.7	NS
CD34	All	16	15.1	NS
CDH1	All	15	14.2	NS
CDKN2A	All	18	17	NS
COL1A1	All	13	12.3	NS
COL2A1	1405	67	63.2	NS
	All	76	71.7	NS
CSFR1	All	12	11.3	NS
CTBNN1	All	13	12.3	NS
CUL9	1948	72	67.9	0.024
	All	80	75.5	0.018
DST	All	10	9.4	NS
EGFR	468	82	77.4	NS
	All	82	77.4	NS
ERBB2	95	24	22.6	0.062
	1155	52	49.1	NS
	All	71	67	NS
ESRRA	All	20	18.9	NS
FAM129A	609	22	20.8	NS
	All	31	29.2	NS
FBXW7	ALL	12	11.3	NS
FCGBP	1340	46	43.4	NS
	15	20	18.9	NS
FGFR2	429	16	15.1	NS
	All	32	30.2	NS
FGFR3	All	18	17	NS
FLT3	All	13	12.3	NS
G6PD	All	60	57	NS
GNA11	All	68	64.2	NS
POU5F1B	214	17	16	NS

	All	16	15.1	NS
PTPN11	All	13	12.3	NS
KIT	All	33	31.1	NS
LY6G6D	112	23	21.7	0.014
LAMA4	1119	59	55.7	NS
	490	16	15.1	NS
MAGEC1	151	19	17.9	NS
	241	30	28.3	NS
	All	55	51.9	0.084
MDC1	1873	43	40.6	NS
	1558	59	55.7	NS
	1354	13	12.3	NS
	1349	16	15.1	NS
	1316	25	23.6	NS
	241	16	15.1	NS
	512	12	11.3	NS
MDN1	862	12	11.3	NS
	440	41	38.7	NS
	All	62	58.5	NS
MEGF6	1137	27	25.5	NS
	125	61	57.5	NS
	All	94	88.7	NS
MET	All	22	20.8	NS
MICB	80	44	41.5	0.08
	89	12	11.3	NS
	104	46	43.4	NS
MMP2	All	39	36.8	NS
MPL	All	18	17	NS
NOTCH1	All	17	16	NS
PDCD1LG2	All	18	17	NS
PDGFRA	All	21	19.8	NS
PTCH1	1314	67	63.2	0.017
	All	82	77.4	0.061
PTPN23	818	54	19.8	NS
	1121	21	19.8	NS
	All	77	72.6	0.091
RBMXL3	398	56	52.8	0.039
	1006	15	14.2	NS
	1049	13	12.3	NS
	All	85	80.2	0.042
RET	336	13	12.3	NS
	All	36	34	NS
SERBP1	All	11	10.4	NS
STK11	All	20	18.9	NS
TAPBP	84	16	15.1	NS

	70	33	31.1	NS
	All	82	77.4	NS
THBS1	All	15	14.2	NS
TP53	72	81	76.4	NS
	175	16	15.1	NS
TNXB	All	15	14.2	NS
TYK2	362	64	60.4	NS
	All	79	74.5	NS
ZNF462	1828	76	71.7	NS
	404	21	19.8	NS
	All	83	78.3	NS

NS, not significant (p > 0.10)

* "All" indicated that mutational status of a given gene was determined by all mutations sequenced; Numbers indicated that mutational status of a given gene was determined by specific codon.

**was caculated among all sequenced samples

Table S10. Summary of the second-step procedure of selecting potential predictive biomarker

Gene ID	Codon*	Mutation number	Mutation rate, %	Unadjusted pvalue for interaction test	Adjusted with propensity score
ATP6V1B1	30	43	40.6	0.054	0.033
CUL9	1948	80	75.5	0.008	0.005
ERBB2	95	24	22.6	0.062	0.048
LY6G6D	112	23	21.7	0.014	0.007
MAGEC1	All	55	51.9	0.077	0.07
PTCH1	1314	67	63.2	0.017	0.018
PTPN23	All	77	72.6	0.097	0.142
RBMXL3	398	56	52.8	0.039	0.045

* "All" indicated that mutational status of a given gene was determined by all mutations sequenced; Numbers indicated that mutational status of a given gene was determined by specific codon.

** Adjusted estimated propensity score based on age (>65 years vs. <=65 years), gender (male vs. female), ECOG PS (0 vs. 1), primary tumor location (right-sided vs. left-sided + rectum), number of liver metastases (<=4 vs. >4)and maximum size of liver metastases (>5cm vs. <5cm).

Table S11. Efficacy outcomes according to mutation status in RAS wild-type patients.

Gene ID	Codon*	Mutation status	Number	PFS Hazard ratios**	PFS 95% Confidence interval	PFS P value	OS Hazard ratios**	OS 95% Confidence interval	OS P value	ORR Odd ratios**	ORR 95% Confidence interval	ORR P value
ATP6V1B1	30	Wild-type	55	**0.50**	**0.27-0.90**	**0.022**	0.45	0.20-1.03	0.058	**8.57**	**2.55-28.85**	**0.001**
		Mutant	38	0.49	0.24-1.002	0.051	0.43	0.15-1.27	0.125	1.48	0.40-5.49	0.553
CUL9	1948	Wild-type	31	0.77	0.37-1.63	0.497	0.71	0.24-2.08	0.531	0.86	0.21-3.58	0.833
		Mutant	62	**0.41**	**0.23-0.72**	**0.002**	**0.31**	**0.13-0.75**	**0.009**	**11.0**	**3.26-37.14**	**<0.001**
ERBB2	95	Wild-type	72	**0.42**	**0.25-0.72**	**0.001**	**0.29**	**0.13-0.63**	**0.002**	**6.02**	**2.16-16.8**	**0.001**
		Mutant	21	1.11	0.41-2.98	0.843	1.69	0.40-7.19	0.476	1.5	0.20-11.5	0.697
LY6G6D	112	Wild-type	74	**0.34**	**0.20-0.58**	**<0.001**	**0.28**	**0.13-0.59**	**0.001**	**7.28**	**2.61-20.3**	**<0.001**
		Mutant	19	1.62	0.60-4.37	0.344	3.01	0.46-19.5	0.249	0.43	0.06-3.22	0.41
PTCH1	1314	Wild-type	34	0.86	0.39-1.90	0.705	0.67	0.23-1.98	0.472	1.09	0.28-4.32	0.901
		Mutant	59	**0.39**	**0.22-0.70**	**0.002**	**0.34**	**0.14-0.82**	**0.017**	**10.3**	**3.06-34.6**	**<0.001**
RBMXL3	398	Wild-type	43	0.6	0.21-1.73	0.342	1.30	0.21-8.11	0.777	0.83	0.15-4.64	0.835
		Mutant	50	**0.50**	**0.30-0.83**	**0.007**	**0.36**	**0.17-0.73**	**0.005**	**6.95**	**2.43-19.83**	**<0.001**

* "All" indicated that mutational status of a given gene was determined by all mutations sequenced; Numbers indicated that mutational status of a given gene was determined by specific codon.

** were calculated with cetuximab arm compared with chemotherapy arm

Table S12. Predictor value and coefficients in the predictive model

	Coefficients	Standard error	P value	Hazard ratios	Predictor value	
					0	1
ATP6V1B1 status	-0.165	0.519	0.750	1.180	Wild-type	Mutant
CUL9 status	-0.726	0.541	0.180	2.067	Wild-type	Mutant
ERBB2 status	-1.140	0.618	0.065	3.127	Wild-type	Mutant
LY6G6D status	-0.944	0.639	0.140	2.570	Wild-type	Mutant
RBMXL3 status	-0.477	0.587	0.417	1.611	Wild-type	Mutant
PTCH1 status	0.821	0.567	0.147	0.440	Wild-type	Mutant
Treatment	1.771	0.506	0.000	5.878	Chemotherapy alone	Cetuximab plus chemotherapy
Constant value	-0.255	0.634	0.688	0.775	-	-

Table S13. Predictive model and primary tumor location

Left-sided	Model-defined Responsive group (N=108)		Model-defined Refractory group (N=50)	
	Cetuximab plus chemotherapy (N=54)	Chemotherapy alone (N=54)	Cetuximab plus chemotherapy (N=20)	Chemotherapy alone (N=30)
ORR, %	85.2	29.6	25.0	30.0
Odd ratios	13.66		0.78	
95%CI	5.28-35.36		0.22-2.79	
P value (chi-square)	*<0.001*		*0.700*	
P value for interaction test		*<0.001*		
Resection rate of LM, %	42.6	9.3	15	13.3
Odd ratios	7.27		1.15	
95%CI	2.50-21.13		0.23-5.78	
P value (chi-square)	*<0.001*		*0.868*	
P value for interaction test		*0.062*		
PFS, months				
Median	12.7	4.8	8.8	7.9
Hazard ratios	0.35		0.90	
95%CI	0.22-0.54		0.49-1.64	
P value (log-rank)	*<0.001*		*0.706*	
P value for interaction test		*0.006*		
OS, months				
Median	48.0	21.7	26.6	23.8
Hazard ratios	0.12		0.91	
95%CI	0.05-0.31		0.37-2.22	
P value (log-rank)	*<0.001*		*0.986*	
P value for interaction test		*0.002*		

Right-sided	Model-defined Responsive group (N=41)		Model-defined Refractory group (N=17)	
	Cetuximab plus chemotherapy (N=17)	Chemotherapy alone (N=24)	Cetuximab plus chemotherapy (N=12)	Chemotherapy alone (N=5)
ORR, %	70.6	29.2	25.0	20.0
Odd ratios	5.83		1.33	
95%CI	1.49-22.82		0.10-17.10	
P value (chi-square)	*0.009*		*1.000*	
P value for interaction test		*0.318*		
Resection rate of LM, %	29.4	8.3	8.3	0
Odd ratios	4.58		0.99	
95%CI	0.77-27.30		-	
P value (chi-square)	*0.105*		*1.000*	
PFS, months				
Median	9	4.8	7.0	7.0

Hazard ratios	0.55		0.77	
95%CI	0.27-1.15		0.26-2.27	
P value (log-rank)	*0.071*		*0.599*	
P value for interaction test		*0.386*		
OS, months				
Median	25.8	15.5	20.2	34.3
Hazard ratios	0.41		2.48	
95%CI	0.18-0.95		0.53-11.35	
P value (log-rank)	*0.030*		*0.226*	
P value for interaction test		*0.030*		

Table S2. Driver somatic mutations detected by WES

Patient ID	Sample type	Sample type 2	Chromosomal location	Gene Symbol	Gene Description	Ensembl transcript identifier	Ensembl protein identifier	Protein position	Amino acid change	Codon change	Existing variation	SIFT	Polyphen-2	Mutation assessor	SIFT-TransFIC	SIFT-TransFIC Label	Polyphen-2 TransFIC	Polyphen-2 TransFIC Label	Mutation assessor TransFIC	Mutation assessor TransFIC Label
1	Liver metastases	Private in metastatic tumor	12:50453703	ACCN2	amiloride-sensitive cation channel 2, neuronal	ENST00000447966	ENSP00000400228	175	R/P	cGg/cCg	-	0.03	0.998	NA	0.647	medium_impact	1.705	high_impact	NA	NA
1	Primary tumor	Common in primary and metastatic tumors	3:64606911	ADAMTS9	ADAM metallopeptidase with thrombospondin type 1 motif, 9	ENST00000295903	ENSP00000295903	870	R/W	Cgg/Tgg	-	0.01	0.998	NA	1.199	medium_impact	1.687	high_impact	NA	NA
1	Liver metastases	Common in primary and metastatic tumors	3:64606911	ADAMTS9	ADAM metallopeptidase with thrombospondin type 1 motif, 9	ENST00000295903	ENSP00000295903	870	R/W	Cgg/Tgg	-	0.01	0.998	NA	1.199	medium_impact	1.687	high_impact	NA	NA
1	Primary tumor	Private in primary tumor	11:47712295	AGBL2	ATP/GTP binding protein-like 2	ENST00000532595	ENSP00000436063	266	I/V	Att/Gtt	-	0.03	0.997	NA	0.602	medium_impact	1.519	high_impact	NA	NA
1	Primary tumor	Private in primary tumor	X:55035653	ALAS2	aminolevulinate, delta-, synthase 2	ENST00000330807	ENSP00000332369	575	F/S	tTc/tCc	-	0	0.996	2.445	2.575	high_impact	1.643	high_impact	1.082	medium_impact

1	Primary tumor	Private in primary tumor	15:85405995	ALPK3	alpha-kinase 3	ENST00000258888	ENSP00000258888	1622	L/P	cTt/cCt	rs187316	0	0.99	1.5	2.008	high_impact	1.46	medium_impact	0.435	low_impact
1	Liver metastases	Private in metastatic tumor	12:123466614	ARL6IP4	ADP-ribosylation-like factor 6 interacting protein 4	ENST00000439686	ENSP00000396365	186	R/S	Cgc/Agc	-	0	0.991	NA	2.062	high_impact	1.355	medium_impact	NA	NA
1	Primary tumor	Private in primary tumor	7:102715804	ARMC10	armadillo repeat containing 10	ENST00000441711	ENSP00000413619	29	R/W	Cgg/Tgg	rs7798381	0.07	0.998	NA	0.371	medium_impact	1.705	high_impact	NA	NA
1	Primary tumor	Common in primary and metastatic tumors	2:96789798	ASTL	astacin-like metallo-endopeptidase (M12 family)	ENST00000342380	ENSP00000343674	363	P/S	Cct/Tct	-	0	0.808	0.895	2.174	high_impact	0.567	medium_impact	-0.137	low_impact
1	Liver metastases	Common in primary and metastatic tumors	2:96789798	ASTL	astacin-like metallo-endopeptidase (M12 family)	ENST00000342380	ENSP00000343674	363	P/S	Cct/Tct	-	0	0.808	0.895	2.174	high_impact	0.567	medium_impact	-0.137	low_impact
1	Liver metastases	Private in metastatic tumor	2:71163086	ATP6V1B1	ATPase, H+ transporting, lysosomal 56/58kDa, V1 subunit B1	ENST00000412314	ENSP00000388353	1	M/T	aTg/aCg	rs11681642	0	0.059	NA	2.106	high_impact	-0.017	low_impact	NA	NA
1	Primary tumor	Private in primary tumor	10:1191860	C10orf47	chromosome 10 open reading frame 47	ENST00000379200	ENSP00000368498	59	S/R	Agc/Cgc	-	0	0.97	NA	2.929	high_impact	0.929	medium_impact	NA	NA
1	Primary tumor	Private in primary tumor	11:61254511	C11orf66	chromosome 11 open reading frame 66	ENST00000535545	ENSP00000437511	76	G/D	gGc/gAc	-	0	0.995	NA	2.929	high_impact	1.384	medium_impact	NA	NA
1	Primary tumor	Common in primary and metastatic	1:25570081	C1orf63	chromosome 1 open reading frame 63	ENST00000417642	ENSP00000411631	240	E/G	gAa/gGa	rs1043879	0	0.907	NA	2.929	high_impact	0.63	medium_impact	NA	NA

		tumors																		
1	Liver metastases	Common in primary and metastatic tumors	1:25570081	C1orf63	chromosome 1 open reading frame 63	ENST0000417642	ENSP0000411631	240	E/G	gAa/gGa	rs1043879	0	0.907	NA	2.929	high_impact	0.63	medium_impact	NA	NA
1	Primary tumor	Common in primary and metastatic tumors	12:49729859	C1QL4	complement component 1, q subcomponent-like 4	ENST0000334221	ENSP0000335285	134	N/K	aaC/aaA	-	0	0.999	3.95	2.062	high_impact	1.894	high_impact	3.076	high_impact
1	Liver metastases	Common in primary and metastatic tumors	12:49729859	C1QL4	complement component 1, q subcomponent-like 4	ENST0000334221	ENSP0000335285	134	N/K	aaC/aaA	-	0	0.999	3.95	2.062	high_impact	1.894	high_impact	3.076	high_impact
1	Primary tumor	Common in primary and metastatic tumors	3:129140499	C3orf25	chromosome 3 open reading frame 25	ENST0000505956	ENSP0000420854	66	E/G	gAg/gGg	rs3774787	0	0.955	NA	2.075	high_impact	0.91	medium_impact	NA	NA
1	Liver metastases	Common in primary and metastatic tumors	3:129140499	C3orf25	chromosome 3 open reading frame 25	ENST0000505956	ENSP0000420854	66	E/G	gAg/gGg	rs3774787	0	0.955	NA	2.075	high_impact	0.91	medium_impact	NA	NA
1	Liver metastases	Private in metastatic tumor	19:15121684	CCDC105	coiled-coil domain containing 105	ENST0000292574	ENSP0000292574	16	V/G	gTt/gGt	-	0	0.788	0.975	2.929	high_impact	0.388	medium_impact	0.08	low_impact
1	Primary tumor	Common in primary and metastatic tumors	1:44461822	CCDC24	coiled-coil domain containing 24	ENST0000372318	ENSP0000361392	305	A/V	gCc/gTc	rs2301983	0	0.832	0.895	2.929	high_impact	0.46	medium_impact	-0.008	low_impact
1	Liver metas	Common in primary and	1:44461822	CCDC2	coiled-coil domain containing 24	ENST000003	ENSP0003613	305	A/V	gCc/gTc	rs2301983	0	0.832	0.895	2.929	high_impact	0.46	medium_impact	-0.008	low_impact

	tases	metastatic tumors		4		72318	92													
1	Primary tumor	Private in primary tumor	1:27709140	CD164L2	CD164 sialomucin-like 2	ENST00000374025	ENSP00000363137	36	F/V	Ttt/Gtt	-	0	0.636	NA	2.929	high_impact	0.2	medium_impact	NA	NA
1	Liver metastases	Private in metastatic tumor	1:117307204	CD2	CD2 molecule	ENST00000369478	ENSP00000358490	238	K/E	Aaa/Gaa	-	0.19	0.993	2.095	-0.043	low_impact	1.53	high_impact	1.48	medium_impact
1	Primary tumor	Private in primary tumor	16:67865940	CENPT	centromere protein T	ENST00000440851	ENSP00000400140	121	S/A	Tcc/Gcc	-	0	0.632	2.19	2.051	high_impact	0.342	medium_impact	1.297	medium_impact
1	Primary tumor	Private in primary tumor	15:93521491	CHD2	chromodomain helicase DNA binding protein 2	ENST00000394196	ENSP00000377747	869	A/T	Gct/Act	-	0	0.993	3.42	2.175	high_impact	1.389	medium_impact	2.458	medium_impact
1	Primary tumor	Common in primary and metastatic tumors	8:121228679	COL14A1	collagen, type XIV, alpha 1	ENST00000523142	ENSP00000429123	320	N/H	Aat/Cat	rs4870723	0.11	1	NA	0.137	medium_impact	1.723	high_impact	NA	NA
1	Liver metastases	Common in primary and metastatic tumors	8:121228679	COL14A1	collagen, type XIV, alpha 1	ENST00000523142	ENSP00000429123	320	N/H	Aat/Cat	rs4870723	0.11	1	NA	0.137	medium_impact	1.723	high_impact	NA	NA
1	Primary tumor	Common in primary and metastatic tumors	12:48398080	COL2A1	collagen, type II, alpha 1	ENST00000380518	ENSP00000369889	9	T/S	Acg/Tcg	rs3803183	0	0	0	2.263	high_impact	-2.068	low_impact	-1.063	low_impact
1	Liver metastases	Common in primary and metastatic	12:48398080	COL2A1	collagen, type II, alpha 1	ENST00000395281	ENSP00000378696	9	T/S	Acg/Tcg	rs3803183	0	0	NA	2.263	high_impact	-2.068	low_impact	NA	NA

		tumors																		
1	Primary tumor	Private in primary tumor	8:113651006	CSMD3	CUB and Sushi multiple domains 3	ENST00000343508	ENSP00000345799	1109	L/I	Ctc/Atc	-	0.34	0.998	NA	-0.559	low_impact	1.613	high_impact	NA	NA
1	Primary tumor	Private in primary tumor	12:111748244	CUX2	cut-like homeobox 2	ENST00000261726	ENSP00000261726	553	L/R	cTg/cGg	-	0	0.976	2.525	2.089	high_impact	1.16	medium_impact	1.885	medium_impact
1	Primary tumor	Private in primary tumor	14:24768248	DHRS1	dehydrogenase/reductase (SDR family) member 1	ENST00000396813	ENSP00000380027	22	G/D	gGc/gAc	-	0	0.998	2.165	1.974	medium_impact	1.666	high_impact	0.672	low_impact
1	Liver metastases	Private in metastatic tumor	10:12154945	DHTKD1	dehydrogenase E1 and transketolase domain containing 1	ENST00000263035	ENSP00000263035	734	M/K	aTg/aAg	-	0	0.98	3.8	1.729	medium_impact	1.559	high_impact	1.711	medium_impact
1	Primary tumor	Private in primary tumor	13:60453394	DIAPH3	diaphanous homolog 3 (Drosophila)	ENST00000267214	ENSP00000267214	596	T/I	aCc/aTc	-	0	0.061	NA	2.122	high_impact	-0.559	low_impact	NA	NA
1	Primary tumor	Private in primary tumor	3:57448553	DNAH12	dynein, axonemal, heavy chain 12	ENST00000351747	ENSP00000295937	777	R/H	cGt/cAt	rs62621232	0	1	3.545	2.056	high_impact	1.81	high_impact	2.425	medium_impact
1	Primary tumor	Private in primary tumor	19:10935877	DNM2	dynamin 2	ENST00000355667	ENSP00000347890	676	M/V	Atg/Gtg	-	0	0.151	NA	2.07	high_impact	-0.194	low_impact	NA	NA
1	Primary tumor	Private in primary tumor	3:137807259	DZIP1L	DAZ interacting protein 1-like	ENST00000327532	ENSP00000332148	321	R/W	Cgg/Tgg	rs2724693	0	0.94	0	2.069	high_impact	0.912	medium_impact	-1.026	low_impact
1	Primary tumor	Private in primary tumor	10:50678251	ERCC6	excision repair cross-complementing rodent repair deficiency, complementation group 6	ENST00000374129	ENSP00000363244	629	L/W	tTg/tGg	-	0	1	NA	2.021	high_impact	2.04	high_impact	NA	NA
1	Liver	Private in	16:8879	FA	family with sequence similarity 38, member A	ENST0	ENSP00	1225	P/	cCt/c	-	0	0.9	NA	2.128	high_im	1.643	high_impa	NA	NA

	metastases	metastatic tumor	2824	M38A		00004 51779	0004082 44		H	At			98			pact		ct		
1	Liver metastases	Private in metastatic tumor	16:88792822	FAM38A	family with sequence similarity 38, member A	ENST00000451779	ENSP00000408244	1226	V/M	Gtg/Atg	-	0.19	0.998	NA	-0.171	low_impact	1.643	high_impact	NA	NA
1	Liver metastases	Private in metastatic tumor	1:16103727	FBLIM1	filamin binding LIM protein 1	ENST00000332305	ENSP00000364920	221	A/D	gCc/gAc	-	0	0.081	NA	2.069	high_impact	-0.377	low_impact	NA	NA
1	Liver metastases	Private in metastatic tumor	1:11710638	FBXO2	F-box protein 2	ENST00000452872	ENSP00000414826	92	C/W	tgC/tgG	-	0	0.997	NA	2.085	high_impact	1.439	medium_impact	NA	NA
1	Primary tumor	Common in primary and metastatic tumors	4:153247366	FBXW7	F-box and WD repeat domain containing 7	ENST00000393956	ENSP00000377528	303	R/Q	cGa/cAa	COSM22974	0	1	NA	2.066	high_impact	1.96	high_impact	NA	NA
1	Liver metastases	Common in primary and metastatic tumors	4:153247366	FBXW7	F-box and WD repeat domain containing 7	ENST00000393956	ENSP00000377528	303	R/Q	cGa/cAa	COSM22974	0	1	NA	2.066	high_impact	1.96	high_impact	NA	NA
1	Primary tumor	Common in primary and metastatic tumors	9:133779519	FIBCD1	fibrinogen C domain containing 1	ENST00000372338	ENSP00000361413	440	W/R	Tgg/Cgg	-	0.01	1	3.22	1.019	medium_impact	1.957	high_impact	2.097	medium_impact
1	Liver metastases	Common in primary and metastatic tumors	9:133779519	FIBCD1	fibrinogen C domain containing 1	ENST00000448616	ENSP00000414501	440	W/R	Tgg/Cgg	-	0.01	1	3.22	1.019	medium_impact	1.957	high_impact	2.097	medium_impact
1	Primary	Common in primary and	2:186668645	FSIP2	fibrous sheath interacting protein 2	ENST0 00004	ENSP00 0004013	4871	L/R	cTt/cGt	-	0	0	0.805	2.929	high_impact	-1.667	low_impact	-0.106	low_impact

	tumor	metastatic tumors				24728	06													
1	Liver metastases	Common in primary and metastatic tumors	2:186668645	FSIP2	fibrous sheath interacting protein 2	ENST00000424728	ENSP00000401306	4871	L/R	cTt/cGt	-	0	0	0.805	2.929	high_impact	-1.667	low_impact	-0.106	low_impact
1	Primary tumor	Private in primary tumor	6:139488036	HECA	headcase homolog (Drosophila)	ENST00000367658	ENSP00000356630	296	E/V	gAg/gTg	-	0	0.955	0.805	2.064	high_impact	0.955	medium_impact	-0.196	low_impact
1	Liver metastases	Private in metastatic tumor	5:137089673	HNRNPA0	heterogeneous nuclear ribonucleoprotein A0	ENST00000314940	ENSP00000316042	28	E/V	gAg/gTg	-	0	0.382	2.265	2.056	high_impact	0.093	medium_impact	1.215	medium_impact
1	Primary tumor	Private in primary tumor	15:74467345	ISLR	immunoglobulin superfamily containing leucine-rich repeat	ENST00000249842	ENSP00000249842	49	P/L	cCg/cTg	-	0	0.416	2.02	2.062	high_impact	0.122	medium_impact	1.052	medium_impact
1	Liver metastases	Private in metastatic tumor	2:26951419	KCNK3	potassium channel, subfamily K, member 3	ENST00000538762	ENSP00000444002	267	R/C	Cgc/Tgc	-	0	1	NA	2.511	high_impact	2.082	high_impact	NA	NA
1	Liver metastases	Private in metastatic tumor	16:85667696	KIAA0182	KIAA0182	ENST00000253458	ENSP00000253458	62	A/T	Gcc/Acc	-	0	0	0.805	2.062	high_impact	-1.483	low_impact	-0.223	low_impact
1	Primary tumor	Private in primary tumor	14:104642127	KIF26A	kinesin family member 26A	ENST00000423312	ENSP00000388241	1001	R/H	cGc/cAc	-	0	0.353	0.6	2.07	high_impact	0.082	medium_impact	-0.442	low_impact
1	Primary tumor	Private in primary tumor	22:50987287	KLHDC7B	kelch domain containing 7B	ENST00000395676	ENSP00000379034	231	Q/R	cAg/cGg	rs5770886	0	0.033	1.95	2.062	high_impact	-0.621	low_impact	0.978	low_impact
1	Primary tumor	Private in primary tumor	12:10588530	KLRC2	killer cell lectin-like receptor subfamily C, member 2	ENST00000381901	ENSP00000371326	19	R/P	cGg/cCg	rs34195537	0	0.719	1.9	2.056	high_impact	0.449	medium_impact	0.985	low_impact

1	Liver metastases	Private in metastatic tumor	1:47013464	KNCN	kinocilin	ENST00000396314	ENSP00000379607	82	S/P	Tcc/Ccc	-	0	0.629	NA	2.929	high_impact	0.192	medium_impact	NA	NA
1	Primary tumor	Common in primary and metastatic tumors	12:25398281	KRAS	v-Ki-ras2 Kirsten rat sarcoma viral oncogene homolog	ENST00000256078	ENSP00000256078	13	G/D	gGc/gAc	rs112445441	0	0.259	3.375	2.172	high_impact	-0.181	low_impact	2.209	medium_impact
1	Liver metastases	Common in primary and metastatic tumors	12:25398281	KRAS	v-Ki-ras2 Kirsten rat sarcoma viral oncogene homolog	ENST00000256078	ENSP00000256078	13	G/D	gGc/gAc	rs112445441	0	0.259	3.375	2.172	high_impact	-0.181	low_impact	2.209	medium_impact
1	Liver metastases	Private in metastatic tumor	17:39505658	KRT33A	keratin 33A	ENST00000007735	ENSP00000007735	124	N/T	aAt/aCt	-	0.02	0.999	3.685	0.795	medium_impact	1.682	high_impact	2.312	medium_impact
1	Primary tumor	Private in primary tumor	21:15516948	LIPI	lipase, member I	ENST00000536861	ENSP00000440381	431	E/K	Gaa/Aaa	rs2822432	0	0.024	NA	2.044	high_impact	-0.649	low_impact	NA	NA
1	Liver metastases	Private in metastatic tumor	15:74219546	LOXL1	lysyl oxidase-like 1	ENST00000395162	ENSP00000378591	3	R/L	cGg/cTg	rs1048661	0	0.944	NA	2.235	high_impact	0.329	medium_impact	NA	NA
1	Primary tumor	Common in primary and metastatic tumors	12:40657700	LRRK2	leucine-rich repeat kinase 2	ENST00000298910	ENSP00000298910	551	N/K	aaC/aaG	rs7308720	0	0.998	1.905	2.644	high_impact	1.716	high_impact	1.535	medium_impact
1	Liver metastases	Common in primary and metastatic tumors	12:40657700	LRRK2	leucine-rich repeat kinase 2	ENST00000298910	ENSP00000298910	551	N/K	aaC/aaG	rs7308720	0	0.998	1.905	2.644	high_impact	1.716	high_impact	1.535	medium_impact
1	Liver	Private in	20:6033	LRR	leucine rich repeat neuronal 4	ENST0	ENSP00	148	L/	Ctc/T	rs611	0	0.9	3.685	2.062	high_im	1.031	medium_i	2.798	medium_imp

	metastases	metastatic tumor	004	N4		0000378858	000368135		F	tc	7050		67			pact		mpact		act
1	Primary tumor	Common in primary and metastatic tumors	X:140994407	MAGEC1	melanoma antigen family C, 1	ENST00000285879	ENSP00000285879	406	S/C	tCt/tGt	rs62611965	0	0	0	2.062	high_impact	-1.483	low_impact	-1.067	low_impact
1	Liver metastases	Common in primary and metastatic tumors	X:140994407	MAGEC1	melanoma antigen family C, 1	ENST00000285879	ENSP00000285879	406	S/C	tCt/tGt	rs62611965	0	0	0	2.062	high_impact	-1.483	low_impact	-1.067	low_impact
1	Liver metastases	Private in metastatic tumor	15:94841691	MCTP2	multiple C2 domains, transmembrane 2	ENST00000456504	ENSP00000388887	66	R/Q	cGg/cAg	rs61737195	0	0.849	NA	2.075	high_impact	0.59	medium_impact	NA	NA
1	Primary tumor	Private in primary tumor	1:156446909	MEF2D	myocyte enhancer factor 2D	ENST00000353795	ENSP00000344705	204	K/N	aaG/aaC	-	0.01	0.996	NA	1.127	medium_impact	1.585	high_impact	NA	NA
1	Primary tumor	Private in primary tumor	1:3411201	MEGF6	multiple EGF-like-domains 6	ENST00000356575	ENSP00000348982	1326	W/R	Tgg/Agg	-	0	0.999	3.005	2.075	high_impact	1.838	high_impact	1.983	medium_impact
1	Liver metastases	Private in metastatic tumor	22:40859263	MKL1	megakaryoblastic leukemia (translocation) 1	ENST00000422851	ENSP00000398478	17	R/Q	cGg/cAg	-	0	1	NA	2.175	high_impact	2.245	high_impact	NA	NA
1	Liver metastases	Private in metastatic tumor	17:36868963	MLLT6	myeloid/lymphoid or mixed-lineage leukemia (trithorax homolog, Drosophila)	ENST00000325718	ENSP00000316426	247	R/H	cGc/cAc	-	0	0	1.845	2.069	high_impact	-1.491	low_impact	0.815	low_impact
1	Primary tumor	Common in primary and metastatic tumors	2:242036797	MTERFD2	MTERF domain containing 2	ENST00000406593	ENSP00000384998	1	M/T	aTg/aCg	rs2286323	0	0.708	NA	2.929	high_impact	0.282	medium_impact	NA	NA

1	Liver metastases	Common in primary and metastatic tumors	2:242036797	MTERFD2	MTERF domain containing 2	ENST00000406593	ENSP00000384998	1	M/T	aTg/aCg	rs2286323	0	0.708	NA	2.929	high_impact	0.282	medium_impact	NA	NA
1	Primary tumor	Private in primary tumor	12:10588530	NA	NA	ENST00000539033	ENSP00000437563	19	R/P	cGg/cCg	rs34195537	0	0.988	NA	2.117	high_impact	1.27	medium_impact	NA	NA
1	Liver metastases	Private in metastatic tumor	17:15517237	NA	NA	ENST00000455584	ENSP00000402644	586	L/V	Ctg/Gtg	rs62070406	0	0	NA	2.064	high_impact	-1.516	low_impact	NA	NA
1	Primary tumor	Common in primary and metastatic tumors	1:201777250	NAV1	neuron navigator 1	ENST00000367295	ENSP00000356264	879	S/L	tCg/tTg	rs2820289	0.002	0.996	NA	0.83	medium_impact	1.5	high_impact	NA	NA
1	Liver metastases	Common in primary and metastatic tumors	1:201777250	NAV1	neuron navigator 1	ENST00000367295	ENSP00000356264	879	S/L	tCg/tTg	rs2820289	0.002	0.996	NA	0.83	medium_impact	1.5	high_impact	NA	NA
1	Liver metastases	Private in metastatic tumor	3:47038034	NBEAL2	neurobeachin-like 2	ENST00000450053	ENSP00000415034	809	F/V	Ttt/Gtt	-	0	0.92	2.16	2.117	high_impact	0.794	medium_impact	1.075	medium_impact
1	Primary tumor	Private in primary tumor	19:56569629	NLRP5	NLR family, pyrin domain containing 5	ENST00000390649	ENSP00000375063	1108	S/C	tCt/tGt	rs12462795	0.004	0.998	3.17	0.55	medium_impact	1.666	high_impact	1.881	medium_impact
1	Liver metastases	Private in metastatic tumor	12:57619160	NXPH4	neurexophilin 4	ENST00000349394	ENSP00000333593	186	L/R	cTt/cGt	-	0	0.788	0	2.064	high_impact	0.523	medium_impact	-1.036	low_impact
1	Liver metas	Private in metastatic	1:248112809	OR2L8	olfactory receptor, family 2, subfamily L, member 8	ENST000003	ENSP000003497	217	Y/C	tAt/tGt	rs71535243	0	1	3.76	1.67	medium_imp	1.724	high_impact	1.471	medium_impact

	tases	tumor				57191	19									act				
1	Primary tumor	Private in primary tumor	11:5758062	OR56B1	olfactory receptor, family 56, subfamily B, member 1	ENST00000317121	ENSP00000322939	106	C/R	Tgc/Cgc	rs7397032	0	1	4	1.67	medium_impact	1.724	high_impact	1.629	medium_impact
1	Primary tumor	Private in primary tumor	11:65562132	OVOL1	ovo-like 1(Drosophila)	ENST00000335987	ENSP00000337862	148	C/R	Tgc/Cgc	-	0	1	3.6	2.064	high_impact	2.005	high_impact	2.579	medium_impact
1	Primary tumor	Common in primary and metastatic tumors	9:5557708	PDCD1LG2	programmed cell death 1 ligand 2	ENST00000397745	ENSP00000380853	151	I/T	aTa/aCa	rs7854413	0	0.889	NA	2.064	high_impact	0.713	medium_impact	NA	NA
1	Liver metastases	Common in primary and metastatic tumors	9:5557708	PDCD1LG2	programmed cell death 1 ligand 2	ENST00000397745	ENSP00000380853	151	I/T	aTa/aCa	rs7854413	0	0.889	NA	2.064	high_impact	0.713	medium_impact	NA	NA
1	Primary tumor	Private in primary tumor	16:81213264	PKD1L2	polycystic kidney disease 1-like 2	ENST00000526632	ENSP00000436389	277	R/Q	cGa/cAa	-	0.62	0.996	NA	-0.778	low_impact	1.546	high_impact	NA	NA
1	Primary tumor	Private in primary tumor	14:19553556	POTEG	POTE ankyrin domain family, member G	ENST00000409832	ENSP00000386971	47	G/E	gGa/gAa	rs79489020	0	0.999	NA	2.062	high_impact	1.894	high_impact	NA	NA
1	Liver metastases	Private in metastatic tumor	16:31095647	PRSS53	protease, serine, 53	ENST00000280606	ENSP00000280606	479	G/R	Ggg/Cgg	-	0	0.996	4.165	2.061	high_impact	1.514	high_impact	2.539	medium_impact
1	Primary tumor	Private in primary tumor	16:31095649	PRSS53	protease, serine, 53	ENST00000280606	ENSP00000280606	478	S/C	tCt/tGt	-	0	0.996	4.225	2.061	high_impact	1.514	high_impact	2.591	medium_impact
1	Primary	Private in primary tumor	19:40902565	PRX	periaxin	ENST000003	ENSP000003260	565	P/R	cCa/cGa	-	0.	0.998	2.39	1.128	medium_imp	1.738	high_impact	1.457	medium_impact

	tumor					24001	18					01				act				
1	Primary tumor	Private in primary tumor	6:31106516	PSORS1C1	psoriasis susceptibility 1 candidate 1	ENST00000259881	ENSP00000259881	43	P/S	Cct/Tct	rs9501057	0	0.036	0	2.929	high_impact	-0.762	low_impact	-0.987	low_impact
1	Liver metastases	Private in metastatic tumor	3:47453783	PTPN23	protein tyrosine phosphatase, non-receptor type 23	ENST00000431726	ENSP00000387825	1271	G/C	Ggc/Tgc	-	0.01	1	NA	1.119	medium_impact	2.005	high_impact	NA	NA
1	Liver metastases	Private in metastatic tumor	8:94746399	RBM12B	RNA binding motif protein 12B	ENST00000517700	ENSP00000427729	627	F/Y	tTt/tAt	-	0	0.326	NA	2.056	high_impact	0.032	medium_impact	NA	NA
1	Liver metastases	Private in metastatic tumor	X:114426361	RBMXL3	RNA binding motif protein, X-linked-like 3	ENST00000424776	ENSP00000417451	786	S/L	tCg/tTg	-	0	0.403	0	2.056	high_impact	0.114	medium_impact	-1.129	low_impact
1	Primary tumor	Private in primary tumor	10:43598017	RET	ret proto-oncogene	ENST00000535749	ENSP00000445963	189	R/C	Cgc/Tgc	-	0.01	1	NA	1.236	medium_impact	1.904	high_impact	NA	NA
1	Primary tumor	Common in primary and metastatic tumors	22:32586875	RFPL2	ret finger protein-like 2	ENST00000400236	ENSP00000383095	251	R/C	Cgc/Tgc	rs136468	0.13	1	NA	0.035	medium_impact	1.946	high_impact	NA	NA
1	Liver metastases	Common in primary and metastatic tumors	22:32586875	RFPL2	ret finger protein-like 2	ENST00000248980	ENSP00000248980	280	R/C	Cgc/Tgc	rs136468	0.05	1	NA	0.459	medium_impact	1.946	high_impact	NA	NA
1	Primary	Private in primary tumor	3:16475541	RFTN1	raftlin, lipid raft linker 1	ENST00000004	ENSP000004039	14	E/G	gAg/gGg	-	0	0.724	NA	2.929	high_impact	0.301	medium_impact	NA	NA

	tumor					32519	26													
1	Liver metastases	Private in metastatic tumor	8:145556990	SCRT1	scratch homolog 1, zinc finger protein (Drosophila)	ENST00000332135	ENSP00000331692	302	K/E	Aag/Gag	-	0	0	1.7	2.066	high_impact	-1.495	low_impact	0.752	low_impact
1	Liver metastases	Private in metastatic tumor	19:15067421	SLC1A6	solute carrier family 1 (high affinity aspartate/glutamate transporter), member 6	ENST00000430939	ENSP00000409386	282	V/I	Gtc/Atc	-	0.04	0.699	NA	0.386	medium_impact	0.334	medium_impact	NA	NA
1	Primary tumor	Private in primary tumor	11:92930991	SLC36A4	solute carrier family 36 (proton/amino acid symporter), member 4	ENST00000326402	ENSP00000317382	3	A/V	gCg/gTg	-	0	0	0	2.124	high_impact	-1.345	low_impact	-1.086	low_impact
1	Primary tumor	Private in primary tumor	2:231036796	SP110	SP110 nuclear body protein	ENST00000358662	ENSP00000351488	601	P/S	Cct/Tct	-	0	0.731	2.485	2.119	high_impact	0.424	medium_impact	1.276	medium_impact
1	Primary tumor	Private in primary tumor	7:99917252	SPDYE3	speedy homolog E3 (Xenopus laevis)	ENST00000437326	ENSP00000401147	94	Y/D	Tac/Gac	-	0	0.996	NA	2.929	high_impact	1.44	medium_impact	NA	NA
1	Primary tumor	Private in primary tumor	X:153050572	SRPK3	SRSF protein kinase 3	ENST00000489426	ENSP00000420058	555	I/T	aTc/aCc	-	0	0.095	NA	2.032	high_impact	-0.323	low_impact	NA	NA
1	Primary tumor	Private in primary tumor	2:108875198	SULT1C3	sulfotransferase family, cytosolic, 1C, member 3	ENST00000376700	ENSP00000365890	179	G/R	Ggg/Agg	rs2219078	0	1	NA	1.877	medium_impact	2.028	high_impact	NA	NA
1	Liver metastases	Private in metastatic tumor	9:113173777	SVEP1	sushi, von Willebrand factor type A, EGF and pentraxin domain containing 1	ENST00000374469	ENSP00000363593	2049	G/C	Ggt/Tgt	-	0.11	1	NA	0.145	medium_impact	1.992	high_impact	NA	NA
1	Primary tumor	Common in primary and metastatic	3:100039736	TBC1D23	TBC1 domain family, member 23	ENST00000394144	ENSP00000377700	647	K/Q	Aaa/Caa	-	0.0	0.998	2.175	0.941	medium_impact	1.765	high_impact	1.235	medium_impact

		tumors										2								
1	Liver metastases	Common in primary and metastatic tumors	3:100039736	TBC1D23	TBC1 domain family, member 23	ENST00000475134	ENSP00000418059	510	K/Q	Aaa/Caa	-	0.02	0.998	NA	0.941	medium_impact	1.765	high_impact	NA	NA
1	Primary tumor	Private in primary tumor	11:124496941	TBRG1	transforming growth factor beta regulator 1	ENST00000375005	ENSP00000364144	94	P/S	Cct/Tct	-	0	1	NA	2.064	high_impact	2.005	high_impact	NA	NA
1	Primary tumor	Private in primary tumor	7:139717533	TBXAS1	thromboxane A synthase 1 (platelet)	ENST00000436047	ENSP00000392361	477	P/R	cCa/cGa	-	0	1	NA	1.928	medium_impact	1.904	high_impact	NA	NA
1	Primary tumor	Private in primary tumor	14:104436924	TDRD9	tudor domain containing 9	ENST00000339063	ENSP00000343545	271	R/H	cGc/cAc	-	0	1	NA	2.043	high_impact	1.941	high_impact	NA	NA
1	Liver metastases	Private in metastatic tumor	3:30715600	TGFBR2	transforming growth factor, beta receptor II (70/80kDa)	ENST00000439925	ENSP00000392572	250	G/R	Gga/Aga	-	0	1	NA	2.316	high_impact	1.566	high_impact	NA	NA
1	Liver metastases	Private in metastatic tumor	20:2384093	TGM6	transglutaminase 6	ENST00000381423	ENSP00000370831	347	P/L	cCc/cTc	-	0	0.297	NA	2.209	high_impact	-0.15	low_impact	NA	NA
1	Primary tumor	Common in primary and metastatic tumors	19:3728609	TJP3	tight junction protein 3 (zona occludens 3)	ENST00000382008	ENSP00000371438	19	R/H	cGc/cAc	rs2067019	0.17	0.997	0.675	-0.066	low_impact	1.625	high_impact	-0.359	low_impact
1	Liver metastases	Common in primary and metastatic tumors	19:3728609	TJP3	tight junction protein 3 (zona occludens 3)	ENST00000382008	ENSP00000371438	19	R/H	cGc/cAc	rs2067019	0.17	0.997	0.675	-0.066	low_impact	1.625	high_impact	-0.359	low_impact
1	Liver metas	Private in metastatic	17:7577539	TP53	tumor protein p53	ENST00005	ENSP000004251	116	R/W	Cgg/Tgg	COSM115	0	1	NA	2.265	high_impact	2.147	high_impact	NA	NA

	tases	tumor				09690	04				64									
1	Liver metastases	Private in metastatic tumor	X:111097265	TRPC5	transient receptor potential cation channel, subfamily C, member 5	ENST00000262839	ENSP00000262839	324	R/W	Cgg/Tgg	-	0	1	3.065	2.387	high_impact	1.724	high_impact	1.542	medium_impact
1	Primary tumor	Private in primary tumor	19:10463225	TYK2	tyrosine kinase 2	ENST00000529739	ENSP00000436155	91	Y/S	tAt/tCt	-	0.04	1	NA	0.684	medium_impact	1.702	high_impact	NA	NA
1	Primary tumor	Common in primary and metastatic tumors	3:49156473	USP19	ubiquitin specific peptidase 19	ENST00000398892	ENSP00000381867	36	D/H	Gat/Cat	rs11552724	0	0.99	NA	2.021	high_impact	1.308	medium_impact	NA	NA
1	Liver metastases	Common in primary and metastatic tumors	3:49156473	USP19	ubiquitin specific peptidase 19	ENST00000398898	ENSP00000381872	36	D/H	Gat/Cat	rs11552724	0	0.99	NA	2.021	high_impact	1.308	medium_impact	NA	NA
1	Liver metastases	Private in metastatic tumor	14:74824783	VRTN	vertebrae development homolog (pig)	ENST00000256362	ENSP00000256362	433	R/W	Cgg/Tgg	-	0	1	1.04	2.064	high_impact	2.005	high_impact	0.03	low_impact
1	Liver metastases	Private in metastatic tumor	11:61026509	VWCE	von Willebrand factor C and EGF domains	ENST00000535710	ENSP00000442570	301	G/R	Ggg/Cgg	-	0	0.995	NA	2.145	high_impact	1.439	medium_impact	NA	NA
1	Primary tumor	Private in primary tumor	2:160132089	WDSUB1	WD repeat, sterile alpha motif and U-box domain containing 1	ENST00000359774	ENSP00000352820	215	K/T	aAa/aCa	rs16843852	0.01	0.994	2.44	1.154	medium_impact	1.595	high_impact	1.601	medium_impact
1	Primary tumor	Private in primary tumor	15:41105043	ZFYVE19	zinc finger, FYVE domain containing 19	ENST00000336455	ENSP00000337824	315	R/W	Cgg/Tgg	-	0.04	0.997	NA	0.554	medium_impact	1.672	high_impact	NA	NA

1	Primary tumor	Common in primary and metastatic tumors	18:74625778	ZNF236	zinc finger protein 236	ENST00000543926	ENSP00000444524	993	K/N	aaG/aaT	-	0	0.998	NA	2.066	high_impact	1.786	high_impact	NA	NA
1	Liver metastases	Common in primary and metastatic tumors	18:74625778	ZNF236	zinc finger protein 236	ENST00000253159	ENSP00000253159	993	K/N	aaG/aaT	-	0	0.998	2.21	2.066	high_impact	1.786	high_impact	1.299	medium_impact
1	Liver metastases	Private in metastatic tumor	15:56974464	ZNF280D	zinc finger protein 280D	ENST00000260435	ENSP00000260435	167	K/R	aAa/aGa	-	0.04	0.999	NA	0.574	medium_impact	2.005	high_impact	NA	NA
1	Primary tumor	Private in primary tumor	19:58438584	ZNF418	zinc finger protein 418	ENST00000396147	ENSP00000379451	322	S/C	tCt/tGt	-	0.01	0.999	0.6	1.164	medium_impact	1.992	high_impact	-0.429	low_impact
1	Primary tumor	Private in primary tumor	5:121488203	ZNF474	zinc finger protein 474	ENST00000296600	ENSP00000296600	173	R/H	cGc/cAc	rs2560306	0	1	2.825	2.069	high_impact	1.946	high_impact	1.793	medium_impact
1	Liver metastases	Private in metastatic tumor	19:20807298	ZNF626	zinc finger protein 626	ENST00000453075	ENSP00000390883	386	F/S	tTt/tCt	-	0	0.999	NA	2.056	high_impact	1.933	high_impact	NA	NA
1	Liver metastases	Private in metastatic tumor	19:24115442	ZNF726	zinc finger protein 726	ENST00000322487	ENSP00000317125	175	C/Y	tGt/tAt	-	0.13	0.998	[sent]	0.092	medium_impact	1.818	high_impact	NA	NA
1	Primary tumor	Private in primary tumor	15:43653780	ZSCAN29	zinc finger and SCAN domain containing 29	ENST00000396976	ENSP00000380174	684	G/R	Ggg/Agg	-	0	0.998	2.83	2.066	high_impact	1.786	high_impact	1.965	medium_impact
2	Prima	Private in	20:3652	ADA	ADAM metallopeptidase domain 33	ENST0	ENSP00	530	W/	tGg/t	-	0	1	NA	2.174	high_im	1.85	high_impa	NA	NA

	ry tumor	primary tumor	789	M33		0000379861	000369190		S	Cg						pact		ct		
2	Primary tumor	Common in primary and metastatic tumors	15:86822926	AGBL1	ATP/GTP binding protein-like 1	ENST0000441037	ENSP00000413001	694	Y/C	tAt/tGt	rs117184817	0	1	NA	2.067	high_impact	1.777	high_impact	NA	NA
2	Liver metastases	Common in primary and metastatic tumors	15:86822926	AGBL1	ATP/GTP binding protein-like 1	ENST0000389298	ENSP00000373949	396	Y/C	tAt/tGt	rs117184817	0	0.995	NA	2.067	high_impact	1.399	medium_impact	NA	NA
2	Liver metastases	Private in metastatic tumor	11:67256797	AIP	aryl hydrocarbon receptor interacting protein	ENST0000279146	ENSP00000279146	113	D/E	gaC/gaG	-	0.15	0.998	1.565	-0.242	low_impact	1.734	high_impact	0.611	low_impact
2	Liver metastases	Private in metastatic tumor	1:104166547	AMY2A	amylase, alpha 2A (pancreatic)	ENST0000393932	ENSP00000377509	387	I/M	atT/atG	-	0	0.091	2.465	2.225	high_impact	-0.357	low_impact	1.046	medium_impact
2	Liver metastases	Private in metastatic tumor	1:1431060	ATAD3B	ATPase family, AAA domain containing 3B	ENST0000308647	ENSP00000311766	604	Y/H	Tac/Cac	rs9792997	0	0	0	2.101	high_impact	-1.431	low_impact	-1.067	low_impact
2	Primary tumor	Common in primary and metastatic tumors	1:193149774	B3GALT2	UDP-Gal:betaGlcNAc beta 1,3-galactosyltransferase, polypeptide 2	ENST0000367434	ENSP00000356404	307	R/C	Cgt/Tgt	-	0.04	0.998	2.715	0.745	medium_impact	2.106	high_impact	1.502	medium_impact
2	Liver metastases	Common in primary and metastatic tumors	1:193149774	B3GALT2	UDP-Gal:betaGlcNAc beta 1,3-galactosyltransferase, polypeptide 2	ENST0000367434	ENSP00000356404	307	R/C	Cgt/Tgt	-	0.04	0.998	2.715	0.745	medium_impact	2.106	high_impact	1.502	medium_impact
2	Primary	Private in primary tumor	17:1174461	BHLHA9	basic helix-loop-helix family, member a9	ENST000003	ENSP000003752	202	G/R	Ggg/Cgg	-	0	0.326	0.55	2.056	high_impact	0.014	medium_impact	-0.51	low_impact

	tumor					91429	48													
2	Liver metastases	Private in metastatic tumor	11:33566521	C11orf41	chromosome 11 open reading frame 41	ENST00000526400	ENSP00000433481	95	K/N	aaG/aaT	-	0	0.011	NA	2.929	high_impact	-1.065	low_impact	NA	NA
2	Primary tumor	Private in primary tumor	17:36830562	C17orf96	chromosome 17 open reading frame 96	ENST00000325814	ENSP00000317905	63	L/V	Ctg/Gtg	rs79676758	0	0.901	0.695	2.929	high_impact	0.612	medium_impact	-0.227	low_impact
2	Liver metastases	Private in metastatic tumor	17:77044085	C1QTNF1	C1q and tumor necrosis factor related protein 1	ENST00000392444	ENSP00000376239	254	E/G	gAa/gGa	-	0.32	0.997	1.45	-0.399	low_impact	1.625	high_impact	0.454	low_impact
2	Liver metastases	Private in metastatic tumor	7:89938680	C7orf63	chromosome 7 open reading frame 63	ENST00000412839	ENSP00000415818	114	T/M	aCg/aTg	rs1134956	0	0.99	NA	2.117	high_impact	1.314	medium_impact	NA	NA
2	Primary tumor	Common in primary and metastatic tumors	9:97535322	C9orf3	chromosome 9 open reading frame 3	ENST00000424143	ENSP00000402171	102	R/M	aGg/aTg	-	0	0	NA	2.306	high_impact	-1.494	low_impact	NA	NA
2	Liver metastases	Common in primary and metastatic tumors	9:97535322	C9orf3	chromosome 9 open reading frame 3	ENST00000428313	ENSP00000401854	61	R/M	aGg/aTg	-	0	1	NA	2.306	high_impact	1.991	high_impact	NA	NA
2	Primary tumor	Common in primary and metastatic tumors	17:65052304	CACNG1	calcium channel, voltage-dependent, gamma subunit 1	ENST00000226021	ENSP00000226021	196	G/S	Ggt/Agt	rs1799938	0.02	0.999	2.665	0.881	medium_impact	1.834	high_impact	2.232	medium_impact
2	Liver metastases	Common in primary and metastatic tumors	17:65052304	CACNG1	calcium channel, voltage-dependent, gamma subunit 1	ENST00000226021	ENSP00000226021	196	G/S	Ggt/Agt	rs1799938	0.02	0.999	2.665	0.881	medium_impact	1.834	high_impact	2.232	medium_impact

2	Primary tumor	Private in primary tumor	1:1848231	CALML6	calmodulin-like 6	ENST0000378604	ENSP0000367867	81	M/I	atG/atA	-	0	0.123	NA	2.075	high_impact	-0.3	low_impact	NA	NA
2	Primary tumor	Common in primary and metastatic tumors	22:37906262	CARD10	caspase recruitment domain family, member 10	ENST0000251973	ENSP0000251973	289	R/Q	cGg/cAg	rs9610775	0.29	0.992	1.87	-0.325	low_impact	1.585	high_impact	1.054	medium_impact
2	Liver metastases	Common in primary and metastatic tumors	22:37906262	CARD10	caspase recruitment domain family, member 10	ENST0000251973	ENSP0000251973	289	R/Q	cGg/cAg	rs9610775	0.29	0.992	1.87	-0.325	low_impact	1.585	high_impact	1.054	medium_impact
2	Primary tumor	Common in primary and metastatic tumors	17:78178893	CARD14	caspase recruitment domain family, member 14	ENST0000344227	ENSP0000344549	820	R/W	Cgg/Tgg	rs11652075	0	0.175	1.04	2.054	high_impact	-0.316	low_impact	-0.033	low_impact
2	Liver metastases	Common in primary and metastatic tumors	17:78178893	CARD14	caspase recruitment domain family, member 14	ENST0000344227	ENSP0000344549	820	R/W	Cgg/Tgg	rs11652075	0	0.175	1.04	2.054	high_impact	-0.316	low_impact	-0.033	low_impact
2	Primary tumor	Private in primary tumor	10:70507311	CCAR1	cell division cycle and apoptosis regulator 1	ENST0000540807	ENSP0000445607	77	P/S	Cct/Tct	-	0	0	NA	2.084	high_impact	-1.484	low_impact	NA	NA
2	Primary tumor	Private in primary tumor	19:15121684	CCDC105	coiled-coil domain containing 105	ENST0000292574	ENSP0000292574	16	V/G	gTt/gGt	-	0	0.788	0.975	2.929	high_impact	0.388	medium_impact	0.08	low_impact
2	Primary tumor	Common in primary and metastatic tumors	1:158151897	CD1D	CD1d molecule	ENST0000368171	ENSP0000357153	135	A/E	gCa/gAa	-	0	0.992	3.105	1.972	medium_impact	1.558	high_impact	2.175	medium_impact
2	Liver	Common in	1:15815	CD1	CD1d molecule	ENST0	ENSP00	135	A/	gCa/	-	0	0.9	3.105	1.972	mediu	1.558	high_impa	2.175	medium_imp

	metastases	primary and metastatic tumors	1897	D		0000368171	000357153		E	gAa			92			m_impact		ct		act
2	Primary tumor	Private in primary tumor	22:46777744	CELSR1	cadherin, EGF LAG seven-pass G-type receptor 1 (flamingo homolog, Drosophila)	ENST00000262738	ENSP00000262738	2363	R/W	Cgg/Tgg	-	0.01	1	2.63	1.205	medium_impact	1.915	high_impact	1.756	medium_impact
2	Liver metastases	Private in metastatic tumor	16:55862844	CES1	carboxylesterase 1	ENST00000422046	ENSP00000390492	31	G/A	gGc/gCc	-	0	1	NA	1.953	medium_impact	1.574	high_impact	NA	NA
2	Primary tumor	Private in primary tumor	3:126261207	CHST13	carbohydrate (chondroitin 4) sulfotransferase 13	ENST00000383575	ENSP00000373069	211	R/W	Cgg/Tgg	rs1056523	0	0	NA	2.103	high_impact	-1.305	low_impact	NA	NA
2	Primary tumor	Private in primary tumor	11:46832681	CKAP5	cytoskeleton associated protein 5	ENST00000312055	ENSP00000310227	169	L/S	tTg/tCg	-	0	0.998	NA	2.117	high_impact	1.705	high_impact	NA	NA
2	Primary tumor	Private in primary tumor	5:79029726	CMYA5	cardiomyopathy associated 5	ENST00000446378	ENSP00000394770	1713	I/N	aTt/aAt	rs16877141	0	0.364	0.69	2.02	high_impact	0.111	medium_impact	-0.403	low_impact
2	Primary tumor	Private in primary tumor	10:105824333	COL17A1	collagen, type XVII, alpha 1	ENST00000393211	ENSP00000376905	210	T/M	aCg/aTg	rs805708	0.25	0.997	NA	-0.316	low_impact	1.512	high_impact	NA	NA
2	Primary tumor	Private in primary tumor	X:107404863	COL4A6	collagen, type IV, alpha 6	ENST00000394872	ENSP00000378340	1441	P/H	cCt/cAt	-	0.14	1	NA	0.046	medium_impact	1.682	high_impact	NA	NA
2	Liver metastases	Private in metastatic tumor	10:76994883	COMTD1	catechol-O-methyltransferase domain containing 1	ENST00000536650	ENSP00000444168	128	G/W	Ggg/Tgg	-	0	1	NA	2.52	high_impact	2.775	high_impact	NA	NA

2	Primary tumor	Common in primary and metastatic tumors	11:68560822	CPT1A	carnitine palmitoyltransferase 1A (liver)	ENST00000539743	ENSP00000446108	310	R/W	Cgg/Tgg	-	0	0.999	NA	1.809	medium_impact	2.053	high_impact	NA	NA
2	Liver metastases	Common in primary and metastatic tumors	11:68560822	CPT1A	carnitine palmitoyltransferase 1A (liver)	ENST00000540367	ENSP00000439084	310	R/W	Cgg/Tgg	-	0	0.997	NA	1.809	medium_impact	1.793	high_impact	NA	NA
2	Primary tumor	Private in primary tumor	6:43188273	CUL9	cullin 9	ENST00000372647	ENSP00000361730	2092	C/S	tGc/tCc	-	0	0.999	NA	1.981	medium_impact	1.955	high_impact	NA	NA
2	Primary tumor	Private in primary tumor	16:70398463	DDX19A	DEAD (Asp-Glu-Ala-As) box polypeptide 19A	ENST00000302227	ENSP00000306209	27	A/G	gCc/gGc	-	0.61	1	NA	-0.836	low_impact	1.941	high_impact	NA	NA
2	Primary tumor	Private in primary tumor	15:40662323	DISP2	dispatched homolog 2 (Drosophila)	ENST00000267889	ENSP00000267889	1337	R/Q	cGg/cAg	-	0	1	1.04	2.064	high_impact	1.91	high_impact	0.049	low_impact
2	Primary tumor	Common in primary and metastatic tumors	4:3475345	DOK7	docking protein 7	ENST00000507039	ENSP00000423614	105	R/C	Cgc/Tgc	rs115883468	0	1	NA	2.158	high_impact	1.993	high_impact	NA	NA
2	Liver metastases	Common in primary and metastatic tumors	4:3475345	DOK7	docking protein 7	ENST00000507039	ENSP00000423614	105	R/C	Cgc/Tgc	rs115883468	0	1	NA	2.158	high_impact	1.993	high_impact	NA	NA
2	Primary tumor	Common in primary and metastatic tumors	18:32443980	DTNA	dystrobrevin, alpha	ENST00000399113	ENSP00000382064	539	R/Q	cGg/cAg	-	0.01	1	2.36	1.155	medium_impact	1.838	high_impact	1.332	medium_impact

2	Liver metastases	Common in primary and metastatic tumors	18:32443980	DTNA	dystrobrevin, alpha	ENST00000399113	ENSP00000382064	539	R/Q	cGg/cAg	-	0.001	1	2.36	1.155	medium_impact	1.838	high_impact	1.332	medium_impact
2	Primary tumor	Private in primary tumor	17:42929181	EFTUD2	elongation factor Tu GTP binding domain containing 2	ENST00000426333	ENSP00000392094	907	V/A	gTg/gCg	-	0	0.999	2.7	1.937	medium_impact	2	high_impact	1.157	medium_impact
2	Primary tumor	Private in primary tumor	11:62369955	EML3	echinoderm microtubule associated protein like 3	ENST00000278845	ENSP00000278845	896	D/Y	Gac/Tac	-	0	0	NA	2.062	high_impact	-1.483	low_impact	NA	NA
2	Primary tumor	Private in primary tumor	2:55126807	EML6	echinoderm microtubule associated protein like 6	ENST00000356458	ENSP00000348842	1004	L/F	ttG/ttT	-	0	1	3.345	2.062	high_impact	1.894	high_impact	2.442	medium_impact
2	Primary tumor	Common in primary and metastatic tumors	17:37880261	ERBB2	v-erb-b2 erythroblastic leukemia viral oncogene homolog 2, neuro/glioblastoma derived oncogene homolog (avian)	ENST00000406381	ENSP00000385185	739	D/Y	Gac/Tac	COSM13170	0	1	NA	2.062	high_impact	1.615	high_impact	NA	NA
2	Liver metastases	Common in primary and metastatic tumors	17:37880261	ERBB2	v-erb-b2 erythroblastic leukemia viral oncogene homolog 2, neuro/glioblastoma derived oncogene homolog (avian)	ENST00000540147	ENSP00000443562	739	D/Y	Gac/Tac	COSM13170	0	1	NA	2.062	high_impact	1.615	high_impact	NA	NA
2	Liver metastases	Private in metastatic tumor	17:74019674	EVPL	envoplakin	ENST00000301607	ENSP00000301607	87	L/P	cTg/cCg	-	0	0.988	1.75	2.126	high_impact	1.127	medium_impact	0.691	low_impact
2	Liver metastases	Private in metastatic tumor	7:4802075	FOXK1	forkhead box K1	ENST00000446823	ENSP00000394442	565	G/R	Ggg/Agg	-	0	0.998	NA	1.754	medium_impact	1.735	high_impact	NA	NA
2	Primary tumor	Common in primary and metastatic	2:49196021	FSHR	follicle stimulating hormone receptor	ENST00000304421	ENSP00000306780	198	D/Y	Gat/Tat	-	0	1	NA	2.128	high_impact	1.897	high_impact	NA	NA

		tumors																		
2	Liver metastases	Common in primary and metastatic tumors	2:49196021	FSHR	follicle stimulating hormone receptor	ENST00000304421	ENSP00000306780	198	D/Y	Gat/Tat	-	0	1	NA	2.128	high_impact	1.897	high_impact	NA	NA
2	Primary tumor	Common in primary and metastatic tumors	17:78085871	GAA	glucosidase, alpha	ENST00000302262	ENSP00000305692	576	G/S	Ggc/Agc	rs1800307	0	1	3.255	2.035	high_impact	1.634	high_impact	2.079	medium_impact
2	Liver metastases	Common in primary and metastatic tumors	17:78085871	GAA	glucosidase, alpha	ENST00000390015	ENSP00000374665	576	G/S	Ggc/Agc	rs1800307	0	1	3.255	2.035	high_impact	1.634	high_impact	2.079	medium_impact
2	Primary tumor	Common in primary and metastatic tumors	14:24707598	GMPR2	guanosine monophosphate reductase 2	ENST00000348719	ENSP00000334409	282	V/M	Gtg/Atg	-	0	0.997	NA	1.972	medium_impact	1.568	high_impact	NA	NA
2	Liver metastases	Common in primary and metastatic tumors	14:24707598	GMPR2	guanosine monophosphate reductase 2	ENST00000348719	ENSP00000334409	282	V/M	Gtg/Atg	-	0	0.997	NA	1.972	medium_impact	1.568	high_impact	NA	NA
2	Primary tumor	Common in primary and metastatic tumors	12:131456051	GPR133	G protein-coupled receptor 133	ENST00000261654	ENSP00000261654	79	K/R	aAg/aGg	-	0	0.967	1.955	2.048	high_impact	1.083	medium_impact	1.158	medium_impact
2	Liver metastases	Common in primary and metastatic tumors	12:131456051	GPR133	G protein-coupled receptor 133	ENST00000261654	ENSP00000261654	79	K/R	aAg/aGg	-	0	0.967	1.955	2.048	high_impact	1.083	medium_impact	1.158	medium_impact
2	Liver	Private in	10:4699	GP	G protein regulated inducer of neurite	ENST0	ENSP00	348	V/	Gtg/	rs492	0	0.8	2.36	2.929	high_im	0.429	medium_i	1.596	medium_imp

	metastases	metastatic tumor	9922	RIN2	outgrowth 2	0000374317	000363436		L	Ttg	6046		14			pact		mpact		act
2	Liver metastases	Private in metastatic tumor	1:55134585	HEATR8	HEAT repeat containing 8	ENST00000438846	ENSP00000404780	455	K/R	aAg/aGg	rs2304314	0	0.889	NA	2.117	high_impact	0.706	medium_impact	NA	NA
2	Primary tumor	Common in primary and metastatic tumors	6:139488030	HECA	headcase homolog (Drosophila)	ENST00000367658	ENSP00000356630	294	L/R	cTc/cGc	-	0	0.975	0.805	2.064	high_impact	1.106	medium_impact	-0.196	low_impact
2	Liver metastases	Common in primary and metastatic tumors	6:139488030	HECA	headcase homolog (Drosophila)	ENST00000367658	ENSP00000356630	294	L/R	cTc/cGc	-	0	0.975	0.805	2.064	high_impact	1.106	medium_impact	-0.196	low_impact
2	Primary tumor	Private in primary tumor	6:34211288	HMGA1	high mobility group AT-hook 1	ENST00000447654	ENSP00000399888	88	K/R	aAa/aGa	-	0	0	0.55	2.039	high_impact	-1.648	low_impact	-0.936	low_impact
2	Primary tumor	Common in primary and metastatic tumors	10:124895955	HMX3	H6 family homeobox 3	ENST00000357878	ENSP00000350549	130	P/L	cCg/cTg	-	0	0.072	0.55	2.056	high_impact	-0.446	low_impact	-0.51	low_impact
2	Liver metastases	Common in primary and metastatic tumors	10:124895955	HMX3	H6 family homeobox 3	ENST00000357878	ENSP00000350549	130	P/L	cCg/cTg	-	0	0.072	0.55	2.056	high_impact	-0.446	low_impact	-0.51	low_impact
2	Primary tumor	Common in primary and metastatic tumors	1:245018856	HNRNPU	heterogeneous nuclear ribonucleoprotein U (scaffold attachment factor A)	ENST00000444376	ENSP00000393151	722	G/V	gGt/gTt	-	0	0.995	NA	2.056	high_impact	1.508	high_impact	NA	NA
2	Liver metas	Common in primary and	1:245018856	HNRN	heterogeneous nuclear ribonucleoprotein U (scaffold attachment factor A)	ENST000004	ENSP000004164	666	G/V	gGt/gTt	-	0	0.995	NA	2.056	high_impact	1.508	high_impact	NA	NA

	tases	metastatic tumors		PU		27948	55													
2	Liver metastases	Private in metastatic tumor	3:186395286	HRG	histidine-rich glycoprotein	ENST00000232003	ENSP00000232003	398	G/R	Gga/Cga	-	0	0.896	2.25	2.142	high_impact	0.825	medium_impact	1.556	medium_impact
2	Liver metastases	Private in metastatic tumor	4:142651060	IL15	interleukin 15	ENST00000320650	ENSP00000323505	101	E/K	Gag/Aag	-	0	1	2.645	1.781	medium_impact	1.971	high_impact	1.021	medium_impact
2	Liver metastases	Private in metastatic tumor	1:62367114	INADL	InaD-like (Drosophila)	ENST00000255202	ENSP00000255202	1122	G/R	Gga/Aga	-	0	1	NA	2.062	high_impact	1.894	high_impact	NA	NA
2	Primary tumor	Common in primary and metastatic tumors	2:121107302	INHBB	inhibin, beta B	ENST00000295228	ENSP00000295228	359	R/H	cGc/cAc	-	0	1	1.665	1.75	medium_impact	1.731	high_impact	0.717	low_impact
2	Liver metastases	Common in primary and metastatic tumors	2:121107302	INHBB	inhibin, beta B	ENST00000295228	ENSP00000295228	359	R/H	cGc/cAc	-	0	1	1.665	1.75	medium_impact	1.731	high_impact	0.717	low_impact
2	Primary tumor	Private in primary tumor	7:4830142	KIAA0415	KIAA0415	ENST00000348624	ENSP00000297562	620	V/M	Gtg/Atg	-	0.01	0.996	NA	1.182	medium_impact	1.537	high_impact	NA	NA
2	Liver metastases	Private in metastatic tumor	17:39739541	KRT14	keratin 14	ENST00000167586	ENSP00000167586	407	R/Q	cGg/cAg	-	0.01	1	2.705	1.124	medium_impact	1.891	high_impact	1.767	medium_impact
2	Primary tumor	Private in primary tumor	17:39505658	KRT33A	keratin 33A	ENST00000007735	ENSP00000007735	124	N/T	aAt/aCt	-	0.0	0.999	3.685	0.795	medium_impact	1.682	high_impact	2.312	medium_impact

												2								
2	Primary tumor	Common in primary and metastatic tumors	17:39216085	KRTAP2-3	keratin associated protein 2-3	ENST00000391418	ENSP00000375237	73	C/Y	tGc/tAc	rs113397060	0	0.997	2.095	2.929	high_impact	1.512	high_impact	1.306	medium_impact
2	Liver metastases	Common in primary and metastatic tumors	17:39216085	KRTAP2-3	keratin associated protein 2-3	ENST00000391418	ENSP00000375237	73	C/Y	tGc/tAc	rs113397060	0	0.997	2.095	2.929	high_impact	1.512	high_impact	1.306	medium_impact
2	Primary tumor	Common in primary and metastatic tumors	4:1825472	LETM1	leucine zipper-EF-hand containing transmembrane protein 1	ENST00000302787	ENSP00000305653	411	P/S	Ccc/Tcc	-	0.14	1	2.01	0.049	medium_impact	1.838	high_impact	0.979	low_impact
2	Liver metastases	Common in primary and metastatic tumors	4:1825472	LETM1	leucine zipper-EF-hand containing transmembrane protein 1	ENST00000302787	ENSP00000305653	411	P/S	Ccc/Tcc	-	0.14	1	2.01	0.049	medium_impact	1.838	high_impact	0.979	low_impact
2	Liver metastases	Private in metastatic tumor	5:176764500	LMAN2	lectin, mannose-binding 2	ENST00000502560	ENSP00000425229	173	M/T	aTg/aCg	-	0	0.994	NA	2.056	high_impact	1.448	medium_impact	NA	NA
2	Primary tumor	Private in primary tumor	20:6033004	LRRN4	leucine rich repeat neuronal 4	ENST00000378858	ENSP00000368135	148	L/F	Ctc/Ttc	rs6117050	0	0.967	3.685	2.062	high_impact	1.031	medium_impact	2.798	medium_impact
2	Liver metastases	Private in metastatic tumor	12:57883279	MARS	methionyl-tRNA synthetase	ENST00000537638	ENSP00000446168	118	R/W	Cgg/Tgg	-	0.02	0.995	NA	0.694	medium_impact	1.89	high_impact	NA	NA
2	Liver metastases	Private in metastatic tumor	1:3418368	MEGF6	multiple EGF-like-domains 6	ENST00000294599	ENSP00000294599	664	C/S	tGt/tCt	-	0	0.416	NA	2.075	high_impact	0.092	medium_impact	NA	NA

2	Primary tumor	Private in primary tumor	17:34094795	MMP28	matrix metallopeptidase 28	ENST00000338839	ENSP0000340652	381	D/Y	Gat/Tat	-	0	0.698	NA	2.174	high_impact	0.426	medium_impact	NA	NA
2	Primary tumor	Common in primary and metastatic tumors	18:11893557	MPPE1	metallophosphoesterase 1	ENST00000309976	ENSP0000311200	100	R/S	agA/agC	-	0	1	3.145	2.451	high_impact	2.178	high_impact	3.098	high_impact
2	Liver metastases	Common in primary and metastatic tumors	18:11893557	MPPE1	metallophosphoesterase 1	ENST00000317251	ENSP0000312935	3	R/S	agA/agC	-	0	1	NA	2.451	high_impact	2.178	high_impact	NA	NA
2	Liver metastases	Private in metastatic tumor	6:30593485	MRPS18B	mitochondrial ribosomal protein S18B	ENST00000259873	ENSP0000259873	230	P/A	Ccc/Gcc	rs34315095	0	0.907	1.95	2.159	high_impact	0.707	medium_impact	0.59	low_impact
2	Liver metastases	Private in metastatic tumor	8:11172476	MTMR9	myotubularin related protein 9	ENST00000526292	ENSP0000433239	254	S/F	tCc/tTc	-	0	0.788	NA	2.398	high_impact	0.779	medium_impact	NA	NA
2	Primary tumor	Common in primary and metastatic tumors	19:9003645	MUC16	mucin 16, cell surface associated	ENST00000542240	ENSP0000444391	172	T/I	aCt/aTt	rs11085777	0	0.76	NA	2.929	high_impact	0.348	medium_impact	NA	NA
2	Liver metastases	Common in primary and metastatic tumors	19:9003645	MUC16	mucin 16, cell surface associated	ENST00000542240	ENSP0000444391	172	T/I	aCt/aTt	rs11085777	0	0.76	NA	2.929	high_impact	0.348	medium_impact	NA	NA
2	Primary tumor	Common in primary and metastatic tumors	17:4451453	MYBBP1A	MYB binding protein (P160) 1a	ENST00000254718	ENSP0000254718	570	R/H	cGc/cAc	rs56716962	0.002	0.999	1.935	0.782	medium_impact	1.877	high_impact	0.961	low_impact
2	Liver	Common in	17:4451	MY	MYB binding protein (P160) 1a	ENST0	ENSP00	570	R/	cGc/	rs567	0	0.9	1.935	0.782	mediu	1.877	high_impa	0.961	low_impact

	metastases	primary and metastatic tumors	453	BBP1A		0000254718	000254718		H	cAc	16962	.02	99			m_impact		ct		
2	Primary tumor	Common in primary and metastatic tumors	14:23894201	MYH7	myosin, heavy chain 7, cardiac muscle, beta	ENST00000544444	ENSP00000446086	819	R/Q	cGg/cAg	-	0	0.594	NA	2.018	high_impact	0.219	medium_impact	NA	NA
2	Liver metastases	Common in primary and metastatic tumors	14:23894201	MYH7	myosin, heavy chain 7, cardiac muscle, beta	ENST00000361594	ENSP00000355048	819	R/Q	cGg/cAg	-	0	0.431	3.01	2.018	high_impact	0.065	medium_impact	0.795	low_impact
2	Primary tumor	Common in primary and metastatic tumors	5:138730037	NA	NA	ENST00000434752	ENSP00000416033	245	Q/R	cAg/cGg	rs11748963	0	0.889	NA	2.929	high_impact	0.58	medium_impact	NA	NA
2	Liver metastases	Common in primary and metastatic tumors	5:138730037	NA	NA	ENST00000434752	ENSP00000416033	245	Q/R	cAg/cGg	rs11748963	0	0.889	NA	2.929	high_impact	0.58	medium_impact	NA	NA
2	Liver metastases	Private in metastatic tumor	9:34724832	NA	NA	ENST00000535536	ENSP00000441758	218	P/L	cCg/cTg	-	0	0.975	NA	2.929	high_impact	0.976	medium_impact	NA	NA
2	Primary tumor	Common in primary and metastatic tumors	1:145368518	NBPF10	neuroblastoma breakpoint family, member 10	ENST00000369338	ENSP00000358344	615	S/L	tCa/tTa	rs61813437	0	0.984	NA	2.929	high_impact	1.09	medium_impact	NA	NA
2	Liver metastases	Common in primary and metastatic tumors	1:145368518	NBPF10	neuroblastoma breakpoint family, member 10	ENST00000369338	ENSP00000358344	615	S/L	tCa/tTa	rs61813437	0	0.984	NA	2.929	high_impact	1.09	medium_impact	NA	NA

2	Primary tumor	Private in primary tumor	14:75558149	NEK9	NIMA (never in mitosis gene a)- related kinase 9	ENST00000238616	ENSP0000238616	756	G/R	Ggg/Agg	-	0	0.94	0	2.128	high_impact	0.898	medium_impact	-1.125	low_impact
2	Primary tumor	Private in primary tumor	10:104161600	NFKB2	nuclear factor of kappa light polypeptide gene enhancer in B-cells 2 (p49/p100)	ENST00000428099	ENSP0000410256	798	L/V	Ctg/Gtg	-	0	0.997	NA	2.033	high_impact	1.523	high_impact	NA	NA
2	Liver metastases	Private in metastatic tumor	11:279551	NLRP6	NLR family, pyrin domain containing 6	ENST00000312165	ENSP0000309767	85	L/P	cTc/cCc	-	0	0.998	2.775	2.011	high_impact	1.666	high_impact	1.516	medium_impact
2	Primary tumor	Private in primary tumor	16:136855	NPRL3	nitrogen permease regulator-like 3 (S. cerevisiae)	ENST00000399953	ENSP0000382834	519	F/Y	tTc/tAc	-	0.01	0.996	NA	1.128	medium_impact	1.566	high_impact	NA	NA
2	Liver metastases	Private in metastatic tumor	15:28269996	OCA2	oculocutaneous albinism II	ENST00000445578	ENSP0000414425	190	C/G	Tgt/Ggt	-	0	0.988	NA	2.037	high_impact	0.744	medium_impact	NA	NA
2	Primary tumor	Common in primary and metastatic tumors	5:167674845	ODZ2	odz, odd Oz/ten-m homolog 2 (Drosophila)	ENST00000518659	ENSP0000429430	2301	R/C	Cgc/Tgc	-	0.18	0.999	1.67	-0.042	low_impact	1.968	high_impact	0.495	low_impact
2	Liver metastases	Common in primary and metastatic tumors	5:167674845	ODZ2	odz, odd Oz/ten-m homolog 2 (Drosophila)	ENST00000545108	ENSP0000438635	2300	R/C	Cgc/Tgc	-	0.18	0.997	NA	-0.042	low_impact	1.709	high_impact	NA	NA
2	Liver metastases	Private in metastatic tumor	11:132307162	OPCML	opioid binding protein/cell adhesion molecule-like	ENST00000416724	ENSP0000390643	173	N/K	aaC/aaA	-	0	1	NA	2.001	high_impact	1.564	high_impact	NA	NA
2	Primary tumor	Common in primary and metastatic	11:6942726	OR2D3	olfactory receptor, family 2, subfamily D, member 3	ENST00000317834	ENSP0000320560	165	W/S	tGg/tCg	rs10839659	0	1	3.88	1.67	medium_impact	1.724	high_impact	1.55	medium_impact

		tumors																		
2	Liver metastases	Common in primary and metastatic tumors	11:6942726	OR2D3	olfactory receptor, family 2, subfamily D, member 3	ENST00000317834	ENSP00000320560	165	W/S	tGg/tCg	rs10839659	0	1	3.88	1.67	medium_impact	1.724	high_impact	1.55	medium_impact
2	Liver metastases	Private in metastatic tumor	16:88932012	PABPN1L	poly(A) binding protein, nuclear 1-like (cytoplasmic)	ENST00000427766	ENSP00000390913	140	E/K	Gag/Aag	-	0	0.762	NA	2.056	high_impact	0.496	medium_impact	NA	NA
2	Liver metastases	Private in metastatic tumor	14:24568323	PCK2	phosphoenolpyruvate carboxykinase 2 (mitochondrial)	ENST00000545054	ENSP00000441826	110	R/W	Cgg/Tgg	rs75497728	0	0.993	NA	1.517	medium_impact	1.529	high_impact	NA	NA
2	Primary tumor	Common in primary and metastatic tumors	8:144991022	PLEC	plectin	ENST00000398774	ENSP00000381756	4291	D/Y	Gac/Tac	-	0.01	1	NA	1.137	medium_impact	1.545	high_impact	NA	NA
2	Liver metastases	Common in primary and metastatic tumors	8:144991022	PLEC	plectin	ENST00000354589	ENSP00000346602	4323	D/Y	Gac/Tac	-	0.01	1	NA	1.137	medium_impact	1.545	high_impact	NA	NA
2	Primary tumor	Private in primary tumor	8:128428284	POU5F1B	POU class 5 homeobox 1B	ENST00000391675	ENSP00000375557	58	W/S	tGg/tCg	-	0	0.002	1.795	2.056	high_impact	-1.366	low_impact	0.719	low_impact
2	Liver metastases	Private in metastatic tumor	22:22042368	PPIL2	peptidylprolyl isomerase (cyclophilin)-like 2	ENST00000446951	ENSP00000405214	112	G/R	Ggg/Cgg	-	0	0.999	NA	1.916	medium_impact	1.788	high_impact	NA	NA
2	Liver metastases	Private in metastatic tumor	16:31095649	PRSS53	protease, serine, 53	ENST00000280606	ENSP00000280606	478	S/C	tCt/tGt	-	0	0.996	4.225	2.061	high_impact	1.514	high_impact	2.591	medium_impact
2	Liver metas	Private in metastatic	6:31106516	PSOR	psoriasis susceptibility 1 candidate 1	ENST000002	ENSP000002598	43	P/S	Cct/Tct	rs9501057	0	0.036	0	2.929	high_impact	-0.762	low_impact	-0.987	low_impact

	tases	tumor		S1C1		59881	81													
2	Primary tumor	Common in primary and metastatic tumors	6:31105988	PSORS1C2	psoriasis susceptibility 1 candidate 2	ENST00000259845	ENSP00000259845	51	G/S	Ggt/Agt	rs2233951	0	0.179	1.04	2.064	high_impact	-0.181	low_impact	0.049	low_impact
2	Liver metastases	Common in primary and metastatic tumors	6:31105988	PSORS1C2	psoriasis susceptibility 1 candidate 2	ENST00000259845	ENSP00000259845	51	G/S	Ggt/Agt	rs2233951	0	0.179	1.04	2.064	high_impact	-0.181	low_impact	0.049	low_impact
2	Primary tumor	Private in primary tumor	3:47453783	PTPN23	protein tyrosine phosphatase, non-receptor type 23	ENST00000265562	ENSP00000265562	1397	G/C	Ggc/Tgc	-	0.01	1	4.285	1.119	medium_impact	2.005	high_impact	3.326	high_impact
2	Primary tumor	Common in primary and metastatic tumors	15:93616975	RGMA	RGM domain family, member A	ENST00000542321	ENSP00000440025	4	L/P	cTg/cCg	rs4598860	0	0	NA	2.929	high_impact	-1.667	low_impact	NA	NA
2	Liver metastases	Common in primary and metastatic tumors	15:93616975	RGMA	RGM domain family, member A	ENST00000542321	ENSP00000440025	4	L/P	cTg/cCg	rs4598860	0	0	NA	2.929	high_impact	-1.667	low_impact	NA	NA
2	Primary tumor	Common in primary and metastatic tumors	6:117650532	ROS1	c-ros oncogene 1 , receptor tyrosine kinase	ENST00000368508	ENSP00000357494	1776	D/H	Gat/Cat	rs12664076	0.07	0.997	NA	0.379	medium_impact	1.632	high_impact	NA	NA
2	Liver metastases	Common in primary and metastatic tumors	6:117650532	ROS1	c-ros oncogene 1 , receptor tyrosine kinase	ENST00000403284	ENSP00000384647	83	D/H	Gat/Cat	rs12664076	0.02	1	NA	0.936	medium_impact	1.904	high_impact	NA	NA

2	Liver metastases	Private in metastatic tumor	1:26142180	SEPN1	selenoprotein N, 1	ENST00000374315	ENSP00000363434	548	R/W	Cgg/Tgg	-	0	0.988	NA	2.064	high_impact	1.291	medium_impact	NA	NA
2	Primary tumor	Private in primary tumor	11:406059	SIGIRR	single immunoglobulin and toll-interleukin 1 receptor (TIR) domain	ENST00000431843	ENSP00000403104	357	G/A	gGt/gCt	-	0	0.998	1.905	2.048	high_impact	1.783	high_impact	1.104	medium_impact
2	Primary tumor	Private in primary tumor	15:48527152	SLC12A1	solute carrier family 12 (sodium/potassium/chloride transporters), member 1	ENST00000380993	ENSP00000370381	389	P/L	cCa/cTa	-	0	1	4.14	2.219	high_impact	1.659	high_impact	3.154	high_impact
2	Liver metastases	Private in metastatic tumor	X:73751224	SLC16A2	solute carrier family 16, member 2 (monocarboxylic acid transporter 8)	ENST00000276033	ENSP00000276033	560	P/A	Ccc/Gcc	-	0.36	0.999	NA	-0.477	low_impact	1.825	high_impact	NA	NA
2	Primary tumor	Private in primary tumor	22:31486802	SMTN	smoothelin	ENST00000333137	ENSP00000329532	292	G/R	Gga/Cga	-	0	0.002	NA	2.062	high_impact	-1.314	low_impact	NA	NA
2	Primary tumor	Common in primary and metastatic tumors	12:57499285	STAT6	signal transducer and activator of transcription 6, interleukin-4 induced	ENST00000300134	ENSP00000300134	260	R/W	Cgg/Tgg	-	0.04	0.998	0.895	0.581	medium_impact	1.688	high_impact	-0.261	low_impact
2	Liver metastases	Common in primary and metastatic tumors	12:57499285	STAT6	signal transducer and activator of transcription 6, interleukin-4 induced	ENST00000535201	ENSP00000441002	150	R/W	Cgg/Tgg	-	0	0.998	NA	2.119	high_impact	1.688	high_impact	NA	NA
2	Primary tumor	Common in primary and metastatic tumors	6:33281576	TAPBP	TAP binding protein (tapasin)	ENST00000434618	ENSP00000395701	35	G/R	Gga/Aga	rs117394742	0	0.126	NA	2.062	high_impact	-0.268	low_impact	NA	NA
2	Liver metas	Common in primary and	6:33281576	TAPBP	TAP binding protein (tapasin)	ENST000004	ENSP000004048	35	G/R	Gga/Aga	rs11739474	0	0.519	NA	2.062	high_impact	0.224	medium_impact	NA	NA

	tases	metastatic tumors				26633	33				2									
2	Prima ry tumor	Common in primary and metastatic tumors	11:6740 2362	TBX 10	T-box 10	ENST0 00003 35385	ENSP00 0003351 91	101	K/ T	aAg/ aCg	rs375 8938	0	0.9 51	1.605	2.008	high_im pact	0.995	medium_i mpact	0.646	low_impact
2	Liver metas tases	Common in primary and metastatic tumors	11:6740 2362	TBX 10	T-box 10	ENST0 00003 35385	ENSP00 0003351 91	101	K/ T	aAg/ aCg	rs375 8938	0	0.9 51	1.605	2.008	high_im pact	0.995	medium_i mpact	0.646	low_impact
2	Prima ry tumor	Private in primary tumor	18:4455 5312	TCE B3C	transcription elongation factor B polypeptide 3C (elongin A3)	ENST0 00003 30682	ENSP00 0003282 32	301	S/ C	tCc/t Gc	rs765 39063	0	0.9 4	1.1	2.174	high_im pact	1.052	medium_i mpact	-0.075	low_impact
2	Liver metas tases	Private in metastatic tumor	17:6722 521	TEK T1	tektin 1	ENST0 00003 38694	ENSP00 0003413 46	116	L/ Q	cTg/c Ag	-	0	1	2.955	2.929	high_im pact	1.787	high_impa ct	2.247	medium_imp act
2	Prima ry tumor	Private in primary tumor	9:35852 947	TM EM8 B	transmembrane protein 8B	ENST0 00003 77988	ENSP00 0003672 27	315	R/ C	Cgt/T gt	-	0 . 0 1	0.9 99	2.665	1.137	mediu m_imp act	1.894	high_impa ct	1.728	medium_imp act
2	Prima ry tumor	Common in primary and metastatic tumors	17:7577 141	TP5 3	tumor protein p53	ENST0 00002 69305	ENSP00 0002693 05	266	G/ E	gGa/ gAa	COS M108 67	0	1	3.3	2.265	high_im pact	2.147	high_impa ct	4.679	high_impact
2	Liver metas tases	Common in primary and metastatic tumors	17:7577 141	TP5 3	tumor protein p53	ENST0 00005 09690	ENSP00 0004251 04	134	G/ E	gGa/ gAa	COS M108 67	0	1	NA	2.265	high_im pact	2.147	high_impa ct	NA	NA
2	Prima ry	Common in primary and	11:5730 715	TRI M22	tripartite motif containing 22	ENST0 00005	ENSP00 0004443	256	V/ D	gTt/g At	-	0	0.9 19	NA	2.344	high_im pact	0.789	medium_i mpact	NA	NA

	tumor	metastatic tumors				45338	78													
2	Liver metastases	Common in primary and metastatic tumors	11:5730715	TRIM22	tripartite motif containing 22	ENST00000455293	ENSP00000412845	367	V/D	gTt/gAt	-	0	0.621	NA	2.344	high_impact	0.324	medium_impact	NA	NA
2	Primary tumor	Common in primary and metastatic tumors	7:98550866	TRRAP	transformation/transcription domain-associated protein	ENST00000355540	ENSP00000347733	1822	V/G	gTg/gGg	-	0	0.999	NA	1.837	medium_impact	1.904	high_impact	NA	NA
2	Liver metastases	Common in primary and metastatic tumors	7:98550866	TRRAP	transformation/transcription domain-associated protein	ENST00000456197	ENSP00000394645	1562	V/G	gTg/gGg	-	0	0.996	NA	1.837	medium_impact	1.563	high_impact	NA	NA
2	Primary tumor	Common in primary and metastatic tumors	6:99956560	USP45	ubiquitin specific peptidase 45	ENST00000500704	ENSP00000424372	67	K/E	Aaa/Gaa	rs7744845	0.466	0.999	NA	-0.64	low_impact	1.858	high_impact	NA	NA
2	Liver metastases	Common in primary and metastatic tumors	6:99956560	USP45	ubiquitin specific peptidase 45	ENST00000500704	ENSP00000424372	67	K/E	Aaa/Gaa	rs7744845	0.466	0.999	NA	-0.64	low_impact	1.858	high_impact	NA	NA
2	Primary tumor	Common in primary and metastatic tumors	1:1372400	VWA1	von Willebrand factor A domain containing 1	ENST00000476993	ENSP00000417185	56	F/C	tTt/tGt	-	0	1	3.715	2.062	high_impact	1.894	high_impact	2.83	medium_impact
2	Liver metastases	Common in primary and metastatic tumors	1:1372400	VWA1	von Willebrand factor A domain containing 1	ENST00000476993	ENSP00000417185	56	F/C	tTt/tGt	-	0	1	3.715	2.062	high_impact	1.894	high_impact	2.83	medium_impact

2	Primary tumor	Common in primary and metastatic tumors	7:73275565	WBSCR28	Williams-Beuren syndrome chromosome region 28	ENST0000320531	ENSP0000316775	14	I/N	aTc/aAc	rs11770052	0	0.018	0.805	2.929	high_impact	-0.94	low_impact	-0.106	low_impact
2	Liver metastases	Common in primary and metastatic tumors	7:73275565	WBSCR28	Williams-Beuren syndrome chromosome region 28	ENST0000320531	ENSP0000316775	14	I/N	aTc/aAc	rs11770052	0	0.018	0.805	2.929	high_impact	-0.94	low_impact	-0.106	low_impact
2	Primary tumor	Common in primary and metastatic tumors	15:53901720	WDR72	WD repeat domain 72	ENST0000396328	ENSP0000379619	981	Q/R	cAg/cGg	-	0	0.953	1.955	2.062	high_impact	0.941	medium_impact	0.984	low_impact
2	Liver metastases	Common in primary and metastatic tumors	15:53901720	WDR72	WD repeat domain 72	ENST0000360509	ENSP0000353699	981	Q/R	cAg/cGg	-	0	0.953	1.955	2.062	high_impact	0.941	medium_impact	0.984	low_impact
2	Liver metastases	Private in metastatic tumor	2:160132089	WDSUB1	WD repeat, sterile alpha motif and U-box domain containing 1	ENST0000409124	ENSP0000386891	215	K/T	aAa/aCa	rs16843852	0.02	0.994	NA	0.863	medium_impact	1.595	high_impact	NA	NA
2	Liver metastases	Private in metastatic tumor	9:131515573	ZER1	zer-1 homolog (C. elegans)	ENST0000291900	ENSP0000291900	206	L/V	Ctc/Gtc	-	0	0.958	1.82	2.11	high_impact	1.091	medium_impact	0.917	low_impact
2	Liver metastases	Private in metastatic tumor	19:21216348	ZNF430	zinc finger protein 430	ENST0000261560	ENSP0000261560	61	R/S	agA/agT	-	0	0.46	3.31	2.084	high_impact	0.214	medium_impact	2.083	medium_impact
2	Liver metastases	Private in metastatic tumor	7:149462789	ZNF467	zinc finger protein 467	ENST0000302017	ENSP0000304769	268	G/S	Ggc/Agc	-	0.01	0.994	1.785	1.14	medium_impact	1.55	high_impact	0.772	low_impact

2	Liver metastases	Private in metastatic tumor	7:99161576	ZNF655	zinc finger protein 655	ENST00000320583	ENSP00000322363	147	E/V	gAa/gTa	-	0	0.955	NA	2.064	high_impact	0.955	medium_impact	NA	NA
3	Primary tumor	Private in primary tumor	12:125603262	AACS	acetoacetyl-CoA synthetase	ENST00000261686	ENSP00000261686	358	P/S	Ccc/Tcc	-	0	0.998	NA	2.04	high_impact	1.922	high_impact	NA	NA
3	Liver metastases	Private in metastatic tumor	5:64595955	ADAMTS6	ADAM metallopeptidase with thrombospondin type 1 motif, 6	ENST00000464680	ENSP00000423551	409	F/L	ttT/ttG	-	0	0.998	NA	2.174	high_impact	1.687	high_impact	NA	NA
3	Liver metastases	Private in metastatic tumor	20:853752	ANGPT4	angiopoietin 4	ENST00000381922	ENSP00000371347	455	D/Y	Gac/Tac	-	0.04	1	2.105	0.423	medium_impact	1.767	high_impact	0.964	low_impact
3	Primary tumor	Private in primary tumor	19:33137427	ANKRD27	ankyrin repeat domain 27 (VPS9 domain)	ENST00000306065	ENSP00000304292	103	E/V	gAa/gTa	-	0	0.992	2.045	2.112	high_impact	1.448	medium_impact	1.158	medium_impact
3	Primary tumor	Private in primary tumor	19:1469239	APC2	adenomatosis polyposis coli 2	ENST00000535453	ENSP00000442954	1980	S/Y	tCc/tAc	-	0.03	0.995	NA	0.657	medium_impact	1.674	high_impact	NA	NA
3	Liver metastases	Private in metastatic tumor	X:138878526	ATP11C	ATPase, class VI, type 11C	ENST00000359686	ENSP00000352715	374	S/F	tCc/tTc	-	0	0.996	NA	2.189	high_impact	1.465	medium_impact	NA	NA
3	Primary tumor	Private in primary tumor	2:71163086	ATP6V1B1	ATPase, H+ transporting, lysosomal 56/58kDa, V1 subunit B1	ENST00000432367	ENSP00000405114	1	M/T	aTg/aCg	rs11681642	0	0.254	NA	2.106	high_impact	0.435	medium_impact	NA	NA
3	Primary tumor	Private in primary tumor	X:77268383	ATP7A	ATPase, Cu++ transporting, alpha polypeptide	ENST00000341514	ENSP00000345728	727	G/V	gGa/gTa	-	0	0.954	1.515	2.025	high_impact	0.637	medium_impact	-0.238	low_impact
3	Prima	Private in	14:9964	BCL	B-cell CLL/lymphoma 11B (zinc finger protein)	ENST0	ENSP00	133	P/	Ccg/	-	0	0.9	NA	2.776	high_im	0.8	medium_i	NA	NA

	ry tumor	primary tumor	2194	11B		0000443726	000387419		T	Acg			2			pact		mpact		
3	Primary tumor	Private in primary tumor	16:66503684	BEAN1	brain expressed, associated with NEDD4, 1	ENST0000536005	ENSP0000442793	69	R/W	Cgg/Tgg	-	0	0.975	NA	2.062	high_impact	1.101	medium_impact	NA	NA
3	Primary tumor	Private in primary tumor	17:41222964	BRCA1	breast cancer 1, early onset	ENST0000352993	ENSP0000312236	514	G/A	gGc/gCc	rs80357414	0.15	0.999	NA	-0.358	low_impact	2.005	high_impact	NA	NA
3	Primary tumor	Common in primary and metastatic tumors	11:82625814	C11orf82	chromosome 11 open reading frame 82	ENST0000528759	ENSP0000431234	12	V/I	Gta/Ata	rs61902276	0	0.003	NA	2.929	high_impact	-1.392	low_impact	NA	NA
3	Liver metastases	Common in primary and metastatic tumors	11:82625814	C11orf82	chromosome 11 open reading frame 82	ENST0000528759	ENSP0000431234	12	V/I	Gta/Ata	rs61902276	0	0.003	NA	2.929	high_impact	-1.392	low_impact	NA	NA
3	Liver metastases	Private in metastatic tumor	4:140201266	C4orf49	chromosome 4 open reading frame 49	ENST0000398955	ENSP0000381928	16	R/T	aGg/aCg	-	0	0.955	1.905	2.929	high_impact	0.824	medium_impact	1.098	medium_impact
3	Liver metastases	Private in metastatic tumor	17:48694902	CACNA1G	calcium channel, voltage-dependent, T type, alpha 1G subunit	ENST0000503607	ENSP0000426558	1668	R/C	Cgc/Tgc	-	0	1	NA	2.574	high_impact	1.829	high_impact	NA	NA
3	Primary tumor	Common in primary and metastatic tumors	16:1245967	CACNA1H	calcium channel, voltage-dependent, T type, alpha 1H subunit	ENST0000348261	ENSP0000334198	196	S/L	tCg/tTg	-	0.02	0.954	2.525	0.983	medium_impact	0.92	medium_impact	3.134	high_impact
3	Liver metastases	Common in primary and metastatic	16:1245967	CACNA1H	calcium channel, voltage-dependent, T type, alpha 1H subunit	ENST0000358590	ENSP0000351401	196	S/L	tCg/tTg	-	0	0.993	NA	2.574	high_impact	1.371	medium_impact	NA	NA

		tumors																		
3	Liver metastases	Private in metastatic tumor	16:88958689	CBFA2T3	core-binding factor, runt domain, alpha subunit 2	ENST00000327483	ENSP00000332122	109	I/S	aTt/aGt	-	0	0.106	NA	2.066	high_impact	-0.301	low_impact	NA	NA
3	Primary tumor	Private in primary tumor	21:30440008	CCT8	chaperonin containing TCP1, subunit 8 (theta)	ENST00000540844	ENSP00000442730	11	K/Q	Aaa/Caa	-	0.002	0.999	NA	0.826	medium_impact	1.968	high_impact	NA	NA
3	Liver metastases	Private in metastatic tumor	15:49048668	CEP152	centrosomal protein 152kDa	ENST00000380950	ENSP00000370337	926	E/V	gAa/gTa	rs117557829	0.002	0.998	NA	0.859	medium_impact	1.829	high_impact	NA	NA
3	Liver metastases	Private in metastatic tumor	11:117299270	DSCAML1	Down syndrome cell adhesion molecule like 1	ENST00000446508	ENSP00000394795	1746	P/H	cCc/cAc	-	0	0.003	NA	2.052	high_impact	-1.182	low_impact	NA	NA
3	Primary tumor	Private in primary tumor	2:216272908	FN1	fibronectin 1	ENST00000345488	ENSP00000273049	814	P/H	cCt/cAt	-	0	1	NA	1.931	medium_impact	1.608	high_impact	NA	NA
3	Liver metastases	Private in metastatic tumor	8:11606529	GATA4	GATA binding protein 4	ENST00000526716	ENSP00000435347	34	A/T	Gcc/Acc	-	0.004	0.99	NA	0.816	medium_impact	1.615	high_impact	NA	NA
3	Liver metastases	Private in metastatic tumor	22:25024072	GGT1	gamma-glutamyltransferase 1	ENST00000404920	ENSP00000385001	110	C/F	tGc/tTc	-	0	0.845	NA	2.489	high_impact	1.486	medium_impact	NA	NA
3	Liver metastases	Private in metastatic tumor	20:57430029	GNAS	GNAS complex locus	ENST00000306120	ENSP00000302237	507	P/T	Cca/Aca	-	0	0.003	NA	2.562	high_impact	-1.297	low_impact	NA	NA
3	Liver metas	Private in metastatic	20:57430029	GNAS	GNAS complex locus	ENST000003	ENSP000003601	570	A/D	gCc/gAc	-	0	0.074	1.1	2.562	high_impact	-0.443	low_impact	-0.535	low_impact

	tases	tumor				71100	41													
3	Primary tumor	Private in primary tumor	6:32713619	HLA-DQA2	major histocompatibility complex, class II, DQ alpha 2	ENST00000374940	ENSP00000364076	128	P/H	cCc/cAc	-	0	1	2.605	1.629	medium_impact	2.165	high_impact	0.579	low_impact
3	Liver metastases	Private in metastatic tumor	6:32729595	HLA-DQB2	major histocompatibility complex, class II, DQ beta 2	ENST00000427449	ENSP00000415997	68	D/V	gAc/gTc	-	0	0.989	NA	2.062	high_impact	1.305	medium_impact	NA	NA
3	Primary tumor	Private in primary tumor	13:110437122	IRS2	insulin receptor substrate 2	ENST00000375856	ENSP00000365016	427	H/D	Cac/Gac	-	0	0.794	1.7	2.101	high_impact	0.808	medium_impact	0.826	low_impact
3	Liver metastases	Private in metastatic tumor	1:110765601	KCNC4	potassium voltage-gated channel, Shaw-related subfamily, member 4	ENST00000413138	ENSP00000388029	232	S/P	Tct/Cct	-	0	0.979	NA	2.511	high_impact	1.289	medium_impact	NA	NA
3	Primary tumor	Private in primary tumor	22:37449227	KCTD17	potassium channel tetramerisation domain containing 17	ENST00000431531	ENSP00000402434	61	D/Y	Gac/Tac	-	0	0.941	NA	2.06	high_impact	1.004	medium_impact	NA	NA
3	Primary tumor	Common in primary and metastatic tumors	1:33231380	KIAA1522	KIAA1522	ENST00000294521	ENSP00000294521	4	F/V	Ttc/Gtc	rs6694085	0	0.12	NA	2.929	high_impact	-0.438	low_impact	NA	NA
3	Liver metastases	Common in primary and metastatic tumors	1:33231380	KIAA1522	KIAA1522	ENST00000373480	ENSP00000362579	4	F/V	Ttc/Gtc	rs6694085	0	0.403	1.445	2.929	high_impact	-0.038	low_impact	0.594	low_impact
3	Liver metastases	Private in metastatic tumor	12:10588530	KLRC2	killer cell lectin-like receptor subfamily C, member 2	ENST00000381901	ENSP00000371326	19	R/P	cGg/cCg	rs34195537	0	0.719	1.9	2.056	high_impact	0.449	medium_impact	0.985	low_impact
3	Primary tumor	Common in primary and metastatic	12:25398284	KRAS	v-Ki-ras2 Kirsten rat sarcoma viral oncogene homolog	ENST00000256078	ENSP00000256078	12	G/D	gGt/gAt	COSM521	0	0.877	3.245	2.172	high_impact	0.599	medium_impact	2.064	medium_impact

		tumors																		
3	Liver metastases	Common in primary and metastatic tumors	12:25398284	KRAS	v-Ki-ras2 Kirsten rat sarcoma viral oncogene homolog	ENST00000256078	ENSP00000256078	12	G/D	gGt/gAt	COSM521	0	0.877	3.245	2.172	high_impact	0.599	medium_impact	2.064	medium_impact
3	Primary tumor	Common in primary and metastatic tumors	9:139649612	LCN8	lipocalin 8	ENST00000371688	ENSP00000360753	141	G/V	gGg/gTg	rs2282258	0	0.998	NA	2.117	high_impact	1.705	high_impact	NA	NA
3	Liver metastases	Common in primary and metastatic tumors	9:139649612	LCN8	lipocalin 8	ENST00000371688	ENSP00000360753	141	G/V	gGg/gTg	rs2282258	0	0.998	NA	2.117	high_impact	1.705	high_impact	NA	NA
3	Liver metastases	Private in metastatic tumor	10:85981890	LRIT2	leucine-rich repeat, immunoglobulin-like and transmembrane domains 2	ENST00000372113	ENSP00000361185	480	G/V	gGc/gTc	-	0	1	0	2.062	high_impact	1.894	high_impact	-1.067	low_impact
3	Primary tumor	Private in primary tumor	6:31683157	LY6G6D	lymphocyte antigen 6 complex, locus G6D	ENST00000375824	ENSP00000364984	9	L/V	Ttg/Gtg	rs3749952	0	0.782	NA	2.929	high_impact	0.379	medium_impact	NA	NA
3	Liver metastases	Private in metastatic tumor	1:85491872	MCOLN3	mucolipin 3	ENST00000370587	ENSP00000359619	310	G/R	Gga/Aga	-	0	1	NA	2.128	high_impact	1.81	high_impact	NA	NA
3	Primary tumor	Common in primary and metastatic tumors	7:151945007	MLL3	myeloid/lymphoid or mixed-lineage leukemia 3	ENST00000355193	ENSP00000347325	838	G/S	Ggt/Agt	rs2479172	NA	0.999	NA	NA	NA	1.617	high_impact	NA	NA
3	Liver metastases	Common in primary and metastatic tumors	7:151945007	MLL3	myeloid/lymphoid or mixed-lineage leukemia 3	ENST00000355193	ENSP00000347325	838	G/S	Ggt/Agt	rs2479172	NA	0.999	NA	NA	NA	1.617	high_impact	NA	NA

3	Primary tumor	Private in primary tumor	10:102743721	MRPL43	mitochondrial ribosomal protein L43	ENST0000370242	ENSP00000359262	192	Q/H	caG/caC	-	0	0.179	NA	2.562	high_impact	-0.36	low_impact	NA	NA
3	Primary tumor	Private in primary tumor	10:102743733	MRPL43	mitochondrial ribosomal protein L43	ENST0000370242	ENSP00000359262	188	Q/H	caG/caC	-	0	0.179	NA	2.562	high_impact	-0.36	low_impact	NA	NA
3	Primary tumor	Common in primary and metastatic tumors	11:59863104	MS4A2	membrane-spanning 4-domains, subfamily A, member 2 (Fc fragment of IgE, high affinity I, receptor for	ENST0000278888	ENSP00000278888	237	E/G	gAa/gGa	rs569108	0	0.443	1.04	1.881	medium_impact	-0.303	low_impact	0.012	low_impact
3	Liver metastases	Common in primary and metastatic tumors	11:59863104	MS4A2	membrane-spanning 4-domains, subfamily A, member 2 (Fc fragment of IgE, high affinity I, receptor for	ENST0000278888	ENSP00000278888	237	E/G	gAa/gGa	rs569108	0	0.443	1.04	1.881	medium_impact	-0.303	low_impact	0.012	low_impact
3	Primary tumor	Private in primary tumor	16:70714717	MTSS1L	metastasis suppressor 1-like	ENST0000254951	ENSP00000254951	62	D/V	gAc/gTc	-	0.13	1	NA	0.104	medium_impact	1.836	high_impact	NA	NA
3	Liver metastases	Private in metastatic tumor	1:26140655	NA	NA	ENST0000527604	ENSP00000432311	37	P/T	Ccc/Acc	-	0	0	NA	2.929	high_impact	-1.667	low_impact	NA	NA
3	Liver metastases	Private in metastatic tumor	12:10588530	NA	NA	ENST0000539033	ENSP00000437563	19	R/P	cGg/cCg	rs34195537	0	0.988	NA	2.117	high_impact	1.27	medium_impact	NA	NA
3	Primary tumor	Common in primary and metastatic tumors	2:133540605	NCKAP5	NCK-associated protein 5	ENST0000317721	ENSP00000380603	1260	P/Q	cCa/cAa	rs13016342	0	0.862	NA	2.062	high_impact	0.653	medium_impact	NA	NA
3	Liver metas	Common in primary and	2:133540605	NCKAP	NCK-associated protein 5	ENST000003	ENSP000003806	1260	P/Q	cCa/cAa	rs13016342	0	0.862	NA	2.062	high_impact	0.653	medium_impact	NA	NA

	tases	metastatic tumors		5		17721	03													
3	Primary tumor	Private in primary tumor	19:36338987	NPHS1	nephrosis 1, congenital, Finnish type (nephrin)	ENST00000378910	ENSP00000368190	466	L/V	Ttg/Gtg	-	0.96	0.923	0.535	-2.302	low_impact	1.541	high_impact	-0.652	low_impact
3	Primary tumor	Private in primary tumor	20:3870079	PANK2	pantothenate kinase 2	ENST00000316562	ENSP00000313377	111	L/Q	cTg/cAg	rs71647828	0	0.89	0	2.015	high_impact	0.794	medium_impact	-1.065	low_impact
3	Primary tumor	Private in primary tumor	17:8167118	PFAS	phosphoribosylformylglycinamidine synthase	ENST00000314666	ENSP00000313490	552	L/P	cTg/cCg	-	0.01	1	3.465	1.056	medium_impact	2.102	high_impact	1.763	medium_impact
3	Liver metastases	Private in metastatic tumor	9:131482719	PKN3	protein kinase N3	ENST00000291906	ENSP00000291906	835	F/S	tTc/tCc	-	0	0.018	3.615	2.572	high_impact	-0.568	low_impact	2.381	medium_impact
3	Primary tumor	Common in primary and metastatic tumors	7:76240786	POMZP3	POM121 and ZP3 fusion	ENST00000310842	ENSP00000309233	187	L/P	cTg/cCg	rs71819724	0	0	0	2.064	high_impact	-1.516	low_impact	-1.036	low_impact
3	Liver metastases	Common in primary and metastatic tumors	7:76240786	POMZP3	POM121 and ZP3 fusion	ENST00000441393	ENSP00000416963	124	L/P	cTg/cCg	rs71819724	0	NA	NA	2.064	high_impact	NA	NA	NA	NA
3	Liver metastases	Private in metastatic tumor	19:52715982	PPP2R1A	protein phosphatase 2, regulatory subunit A, alpha	ENST00000391791	ENSP00000375668	103	R/W	Cgg/Tgg	COSM51210	0	1	NA	2.748	high_impact	1.817	high_impact	NA	NA
3	Primary tumor	Common in primary and metastatic	9:98209594	PTCH1	patched 1	ENST00000375284	ENSP00000364433	107	P/L	cCc/cTc	rs357564	0	0.952	NA	2.256	high_impact	1.218	medium_impact	NA	NA

		tumors																		
3	Liver metastases	Common in primary and metastatic tumors	9:98209594	PTCH1	patched 1	ENST00000375284	ENSP00000364433	107	P/L	cCc/cTc	rs357564	0	0.952	NA	2.256	high_impact	1.218	medium_impact	NA	NA
3	Liver metastases	Private in metastatic tumor	5:63802483	RGS7BP	regulator of G-protein signaling 7 binding protein	ENST00000334025	ENSP00000334851	11	R/H	cGc/cAc	-	0	0.993	0.895	2.062	high_impact	1.417	medium_impact	-0.128	low_impact
3	Liver metastases	Private in metastatic tumor	3:78649426	ROBO1	roundabout, axon guidance receptor, homolog 1 (Drosophila)	ENST00000495273	ENSP00000420637	1548	P/L	cCa/cTa	-	0	0.444	NA	2.172	high_impact	0.032	medium_impact	NA	NA
3	Liver metastases	Private in metastatic tumor	19:50139934	RRAS	related RAS viral (r-ras) oncogene homolog	ENST00000246792	ENSP00000246792	132	R/H	cGc/cAc	-	0.06	1	1.07	0.358	medium_impact	2.094	high_impact	-0.403	low_impact
3	Primary tumor	Private in primary tumor	20:62329767	RTEL1	regulator of telomere elongation helicase 1	ENST00000370006	ENSP00000359023	252	A/P	Gcc/Ccc	-	0	0.952	1.245	2.343	high_impact	1.05	medium_impact	-0.181	low_impact
3	Primary tumor	Private in primary tumor	10:102743724	SEMA4G	sema domain, immunoglobulin domain (Ig), transmembrane domain (TM) and short cytoplasmic domain, (semaphorin) 4G	ENST00000370250	ENSP00000359270	785	P/A	Ccc/Gcc	-	0	0.999	1.04	1.949	medium_impact	1.856	high_impact	-0.081	low_impact
3	Primary tumor	Private in primary tumor	10:102743721	SEMA4G	sema domain, immunoglobulin domain (Ig), transmembrane domain (TM) and short cytoplasmic domain, (semaphorin) 4G	ENST00000210633	ENSP00000210633	789	L/V	Ctg/Gtg	-	0	0.997	NA	1.949	medium_impact	1.593	high_impact	NA	NA
3	Primary tumor	Private in primary tumor	10:102743733	SEMA4G	sema domain, immunoglobulin domain (Ig), transmembrane domain (TM) and short cytoplasmic domain, (semaphorin) 4G	ENST00000521006	ENSP00000430881	788	L/V	Ctg/Gtg	-	0.03	0.997	NA	0.665	medium_impact	1.593	high_impact	NA	NA
3	Liver metas	Private in metastatic	1:26140655	SEPN1	selenoprotein N, 1	ENST000003	ENSP000003634	496	P/T	Ccc/Acc	-	0.	0.998	NA	0.675	medium_imp	1.738	high_impact	NA	NA

	tases	tumor				74315	34					03				act				
3	Primary tumor	Private in primary tumor	1:67895901	SERBP1	SERPINE1 mRNA binding protein 1	ENST00000370994	ENSP00000360033	28	F/S	tTc/tCc	-	0	0.893	NA	2.339	high_impact	0.987	medium_impact	NA	NA
3	Primary tumor	Private in primary tumor	1:156783743	SH2D2A	SH2 domain containing 2A	ENST00000368198	ENSP00000357181	137	G/V	gGc/gTc	-	0.08	1	NA	0.212	medium_impact	1.784	high_impact	NA	NA
3	Liver metastases	Private in metastatic tumor	11:406059	SIGIRR	single immunoglobulin and toll-interleukin 1 receptor (TIR) domain	ENST00000528845	ENSP00000434164	77	G/A	gGt/gCt	-	0	0.99	NA	2.048	high_impact	1.383	medium_impact	NA	NA
3	Primary tumor	Private in primary tumor	16:56936319	SLC12A3	solute carrier family 12 (sodium/chloride transporters), member 3	ENST00000438926	ENSP00000402152	927	R/C	Cgt/Tgt	rs12708965	0.03	0.997	NA	0.866	medium_impact	1.545	high_impact	NA	NA
3	Liver metastases	Private in metastatic tumor	16:31497186	SLC5A2	solute carrier family 5 (sodium/glucose cotransporter), member 2	ENST00000419665	ENSP00000410601	147	L/P	cTt/cCt	-	0	1	NA	2.418	high_impact	1.782	high_impact	NA	NA
3	Primary tumor	Private in primary tumor	5:149582191	SLC6A7	solute carrier family 6 (neurotransmitter transporter, L-proline), member 7	ENST00000524041	ENSP00000428200	338	G/S	Ggc/Agc	rs117381766	0	0.994	NA	1.937	medium_impact	1.555	high_impact	NA	NA
3	Primary tumor	Private in primary tumor	17:27963318	SSH2	slingshot homolog 2 (Drosophila)	ENST00000269033	ENSP00000269033	617	T/S	Aca/Tca	-	0	0.921	1.995	2.16	high_impact	0.851	medium_impact	1.001	medium_impact
3	Primary tumor	Private in primary tumor	17:27958468	SSH2	slingshot homolog 2 (Drosophila)	ENST00000540801	ENSP00000444743	1248	I/M	atA/atG	-	0	0.002	NA	2.16	high_impact	-1.247	low_impact	NA	NA
3	Primary	Private in primary tumor	3:52550165	STAB1	stabilin 1	ENST000003	ENSP000003129	1352	G/V	gGc/gTc	-	0.	0.998	1.445	1.298	medium_imp	1.55	high_impact	0.327	low_impact

	tumor					21725	46					0 1				act				
3	Primary tumor	Private in primary tumor	22:19754319	TBX1	T-box 1	ENST00000332710	ENSP0000331791	473	A/T	Gcc/Acc	-	0	0.464	NA	2.008	high_impact	0.201	medium_impact	NA	NA
3	Primary tumor	Private in primary tumor	2:85826257	TMEM150A	transmembrane protein 150A	ENST00000409668	ENSP0000387292	253	G/E	gGg/gAg	-	0	0.839	1.04	2.929	high_impact	0.473	medium_impact	0.151	low_impact
3	Primary tumor	Common in primary and metastatic tumors	5:34850389	TTC23L	tetratricopeptide repeat domain 23-like	ENST00000502674	ENSP0000425242	119	A/T	Gct/Act	rs77793555	0	0.998	NA	2.117	high_impact	1.705	high_impact	NA	NA
3	Liver metastases	Common in primary and metastatic tumors	5:34850389	TTC23L	tetratricopeptide repeat domain 23-like	ENST00000502674	ENSP0000425242	119	A/T	Gct/Act	rs77793555	0	0.998	NA	2.117	high_impact	1.705	high_impact	NA	NA
3	Liver metastases	Private in metastatic tumor	2:130951949	TUBA3E	tubulin, alpha 3e	ENST00000312988	ENSP0000318197	156	R/W	Cgg/Tgg	rs62165074	0	1	4.245	1.937	medium_impact	2	high_impact	2.469	medium_impact
3	Primary tumor	Private in primary tumor	16:1364057	UBE2I	ubiquitin-conjugating enzyme E2I (UBC9 homolog, yeast)	ENST00000397514	ENSP0000380649	9	L/H	cTc/cAc	-	0	0.999	4.59	2.239	high_impact	2.399	high_impact	7.795	high_impact
3	Primary tumor	Private in primary tumor	9:109687431	ZNF462	zinc finger protein 462	ENST00000277225	ENSP0000277225	413	A/E	gCa/gAa	-	0	0	0.805	2.084	high_impact	-1.484	low_impact	-0.239	low_impact
3	Primary tumor	Private in primary tumor	16:88500641	ZNF469	zinc finger protein 469	ENST00000437464	ENSP0000402343	2227	D/Y	Gat/Tat	-	0	0.86	0.55	2.064	high_impact	0.716	medium_impact	-0.458	low_impact
3	Primary	Common in primary and	19:52942534	ZNF534	zinc finger protein 534	ENST000003 00003	ENSP000003275	620	K/N	aaA/aaT	-	0 .	0.995	2.05	-0.43	low_impact	1.527	high_impact	0.971	low_impact

	tumor	metastatic tumors				32323	38					38								
3	Liver metastases	Common in primary and metastatic tumors	19:52942534	ZNF534	zinc finger protein 534	ENST00000332323	ENSP00000327538	620	K/N	aaA/aaT	-	0.38	0.995	2.05	-0.43	low_impact	1.527	high_impact	0.971	low_impact
3	Primary tumor	Private in primary tumor	19:53075596	ZNF701	zinc finger protein 701	ENST00000540331	ENSP00000444339	34	H/Q	caC/caG	-	0	0	NA	2.084	high_impact	-1.484	low_impact	NA	NA
3	Primary tumor	Private in primary tumor	20:57828953	ZNF831	zinc finger protein 831	ENST00000371030	ENSP00000360069	1397	D/Y	Gat/Tat	-	0	0.47	1.24	2.084	high_impact	0.224	medium_impact	0.164	low_impact
4	Primary tumor	Private in primary tumor	7:44146224	AEBP1	AE binding protein 1	ENST00000223357	ENSP00000223357	111	E/D	gaG/gaC	-	0	0	0.895	2.042	high_impact	-1.287	low_impact	-0.263	low_impact
4	Primary tumor	Private in primary tumor	7:100151813	AGFG2	ArfGAP with FG repeats 2	ENST00000300176	ENSP00000300176	228	D/V	gAc/gTc	-	0	0.996	2.215	2.067	high_impact	1.582	high_impact	1.027	medium_impact
4	Primary tumor	Private in primary tumor	15:86128960	AKAP13	A kinase (PRKA) anchor protein 13	ENST00000458540	ENSP00000394403	1355	E/G	gAa/gGa	-	0	0.993	NA	1.859	medium_impact	1.734	high_impact	NA	NA
4	Primary tumor	Common in primary and metastatic tumors	9:38588602	ANKRD18A	ankyrin repeat domain 18A	ENST00000399703	ENSP00000382610	688	Y/C	tAt/tGt	rs2799163	0.2	0.997	1.04	-0.146	low_impact	1.625	high_impact	0.024	low_impact
4	Liver metastases	Common in primary and metastatic tumors	9:38588602	ANKRD18A	ankyrin repeat domain 18A	ENST00000399703	ENSP00000382610	688	Y/C	tAt/tGt	rs2799163	0.2	0.997	1.04	-0.146	low_impact	1.625	high_impact	0.024	low_impact
4	Prima	Private in	12:5853	AN	anoctamin 2	ENST0	ENSP00	407	C/	tGt/t	-	0	0.9	NA	2.124	high_im	1.808	high_impa	NA	NA

	ry tumor	primary tumor	445	O2		0000356134	000348453		S	Ct			99			pact		ct		
4	Liver metastases	Private in metastatic tumor	19:36362903	APLP1	amyloid beta (A4) precursor-like protein 1	ENST00000221891	ENSP00000221891	272	P/H	cCc/cAc	-	0	0.948	0.55	2.085	high_impact	0.787	medium_impact	-0.551	low_impact
4	Liver metastases	Private in metastatic tumor	1:32205185	BAI2	brain-specific angiogenesis inhibitor 2	ENST00000398547	ENSP00000381555	673	F/S	tTc/tCc	-	0.02	1	NA	0.923	medium_impact	1.955	high_impact	NA	NA
4	Liver metastases	Private in metastatic tumor	20:60988580	C20orf151	chromosome 20 open reading frame 151	ENST00000252998	ENSP00000252998	518	G/R	Ggg/Cgg	-	0	0.995	1.65	2.929	high_impact	1.384	medium_impact	0.819	low_impact
4	Primary tumor	Private in primary tumor	7:89938680	C7orf63	chromosome 7 open reading frame 63	ENST00000449577	ENSP00000391571	422	T/M	aCg/aTg	rs1134956	0	1	NA	2.117	high_impact	1.873	high_impact	NA	NA
4	Liver metastases	Private in metastatic tumor	8:27905120	C8orf80	chromosome 8 open reading frame 80	ENST00000341513	ENSP00000345031	406	P/S	Cca/Tca	-	0.09	0.995	1.04	0.141	medium_impact	1.601	high_impact	-0.253	low_impact
4	Liver metastases	Private in metastatic tumor	9:139740080	C9orf172	chromosome 9 open reading frame 172	ENST00000436881	ENSP00000412388	405	G/V	gGc/gTc	-	0	0.952	0.695	2.929	high_impact	0.807	medium_impact	-0.227	low_impact
4	Primary tumor	Common in primary and metastatic tumors	11:67287539	CABP2	calcium binding protein 2	ENST00000353903	ENSP00000312037	101	R/W	Cgg/Tgg	-	0	1	NA	2.075	high_impact	1.838	high_impact	NA	NA
4	Liver metastases	Common in primary and metastatic tumors	11:67287539	CABP2	calcium binding protein 2	ENST00000353903	ENSP00000312037	101	R/W	Cgg/Tgg	-	0	1	NA	2.075	high_impact	1.838	high_impact	NA	NA

4	Liver metastases	Private in metastatic tumor	12:1989005	CACNA2D4	calcium channel, voltage-dependent, alpha 2/delta subunit 4	ENST00000444595	ENSP00000403371	510	V/M	Gtg/Atg	-	0.003	0.996	NA	0.683	medium_impact	1.594	high_impact	NA	NA
4	Liver metastases	Private in metastatic tumor	2:27447926	CAD	carbamoyl-phosphate synthetase 2, aspartate transcarbamylase, and dihydroorotase	ENST00000403525	ENSP00000384510	479	Q/E	Cag/Gag	-	0	0.954	NA	2.137	high_impact	0.476	medium_impact	NA	NA
4	Primary tumor	Private in primary tumor	10:5541014	CALML5	calmodulin-like 5	ENST00000380332	ENSP00000369689	130	Q/E	Cag/Gag	-	0	0.127	0.895	2.075	high_impact	-0.291	low_impact	-0.148	low_impact
4	Liver metastases	Private in metastatic tumor	5:179149927	CANX	calnexin	ENST00000452673	ENSP00000391646	435	F/L	ttT/ttG	-	0	0.999	3.235	2.124	high_impact	1.9	high_impact	1.845	medium_impact
4	Primary tumor	Private in primary tumor	16:88958689	CBFA2T3	core-binding factor, runt domain, alpha subunit 2	ENST00000327483	ENSP00000332122	109	I/S	aTt/aGt	-	0	0.106	NA	2.066	high_impact	-0.301	low_impact	NA	NA
4	Liver metastases	Private in metastatic tumor	11:93088655	CCDC67	coiled-coil domain containing 67	ENST00000527307	ENSP00000433002	50	R/W	Cgg/Tgg	-	0	0.998	NA	2.355	high_impact	2.187	high_impact	NA	NA
4	Liver metastases	Private in metastatic tumor	7:75443027	CCL24	chemokine (C-C motif) ligand 24	ENST00000222902	ENSP00000222902	3	G/R	Ggc/Cgc	-	0	0.94	2.2	2.023	high_impact	0.854	medium_impact	1.161	medium_impact
4	Liver metastases	Private in metastatic tumor	1:100933556	CDC14A	CDC14 cell division cycle 14 homolog A (S. cerevisiae)	ENST00000544534	ENSP00000442543	295	K/E	Aaa/Gaa	-	0	0.996	NA	2.16	high_impact	1.592	high_impact	NA	NA
4	Liver metastases	Private in metastatic tumor	19:16636226	CHERP	calcium homeostasis endoplasmic reticulum protein	ENST00000546361	ENSP00000439856	8	R/P	cGg/cCg	-	0	0.999	NA	2.056	high_impact	1.904	high_impact	NA	NA
4	Primary	Private in primary tumor	16:75563330	CHST5	carbohydrate (N-acetylglucosamine 6-O) sulfotransferase 5	ENST00003	ENSP000003387	318	T/M	aCg/aTg	rs3826107	0.	0.997	2.61	0.651	medium_imp	1.709	high_impact	1.517	medium_impact

	tumor					36257	83					03				act					
4	Liver metastases	Private in metastatic tumor	17:40841570	CNTNAP1	contactin associated protein 1	ENST00000264638	ENSP00000264638	554	R/C	Cgc/Tgc	-	0.04	1	3.325	0.513	medium_impact	1.784	high_impact	2.545	medium_impact
4	Liver metastases	Private in metastatic tumor	1:36564546	COL8A2	collagen, type VIII, alpha 2	ENST00000481785	ENSP00000436433	181	G/R	Ggg/Cgg	-	0	0	NA	2.126	high_impact	-1.576	low_impact	NA	NA
4	Primary tumor	Private in primary tumor	1:36564545	COL8A2	collagen, type VIII, alpha 2	ENST00000303143	ENSP00000305913	246	G/V	gGg/gTg	-	0	0	4.05	2.126	high_impact	-1.576	low_impact	3.139	high_impact
4	Primary tumor	Private in primary tumor	6:116757558	DSE	dermatan sulfate epimerase	ENST00000359564	ENSP00000352567	643	G/R	Ggg/Cgg	-	0.22	0.995	1.78	-0.226	low_impact	1.52	high_impact	0.279	low_impact
4	Primary tumor	Common in primary and metastatic tumors	6:56437837	DST	dystonin	ENST00000370769	ENSP00000359805	4212	R/H	cGt/cAt	-	NA	0.999	NA	NA	NA	1.936	high_impact	NA	NA
4	Liver metastases	Common in primary and metastatic tumors	6:56437837	DST	dystonin	ENST00000421834	ENSP00000400883	2124	R/H	cGt/cAt	-	0.78	0.999	NA	-1.119	low_impact	1.936	high_impact	NA	NA
4	Liver metastases	Private in metastatic tumor	3:56330129	ERC2	ELKS/RAB6-interacting/CAST family member 2	ENST00000460849	ENSP00000417445	331	R/Q	cGg/cAg	-	0	0.981	1.735	2.051	high_impact	1.194	medium_impact	0.796	low_impact
4	Liver metastases	Private in metastatic tumor	11:64083293	ESRRA	estrogen-related receptor alpha	ENST00000545035	ENSP00000444710	157	R/L	cGg/cTg	-	0.2	1	NA	-0.564	low_impact	1.644	high_impact	NA	NA

												5								
4	Liver metastases	Private in metastatic tumor	11:64083320	ESRRA	estrogen-related receptor alpha	ENST0000545035	ENSP00000444710	166	L/P	cTa/cCa	-	0	NA	NA	2.036	high_impact	NA	NA	NA	NA
4	Liver metastases	Private in metastatic tumor	6:617756	EXOC2	exocyst complex component 2	ENST0000230449	ENSP00000230449	206	K/Q	Aaa/Caa	-	0	0.999	2.44	2.124	high_impact	1.872	high_impact	1.38	medium_impact
4	Primary tumor	Common in primary and metastatic tumors	11:108409784	EXPH5	exophilin 5	ENST0000428840	ENSP00000391966	61	E/V	gAg/gTg	rs2640785	0	0.952	NA	2.013	high_impact	0.95	medium_impact	NA	NA
4	Liver metastases	Common in primary and metastatic tumors	11:108409784	EXPH5	exophilin 5	ENST0000525344	ENSP00000432546	130	E/V	gAg/gTg	rs2640785	0	0.952	NA	2.013	high_impact	0.95	medium_impact	NA	NA
4	Liver metastases	Private in metastatic tumor	11:61643380	FADS3	fatty acid desaturase 3	ENST0000525588	ENSP00000432206	382	K/M	aAg/aTg	-	0	1	NA	1.848	medium_impact	1.852	high_impact	NA	NA
4	Primary tumor	Private in primary tumor	11:58892351	FAM111B	family with sequence similarity 111, member B	ENST0000411426	ENSP00000393855	231	G/R	Gga/Aga	-	0	0.995	NA	2.034	high_impact	1.425	medium_impact	NA	NA
4	Primary tumor	Private in primary tumor	1:184868431	FAM129A	family with sequence similarity 129, member A	ENST0000367511	ENSP00000356481	23	A/T	Gct/Act	-	0.2	0.998	2.045	-0.171	low_impact	1.738	high_impact	1.097	medium_impact
4	Primary tumor	Private in primary tumor	6:71162209	FAM135A	family with sequence similarity 135, member A	ENST0000194672	ENSP00000194672	31	R/H	cGt/cAt	-	0	0.997	NA	2.929	high_impact	1.512	high_impact	NA	NA
4	Liver metastases	Private in metastatic tumor	5:175535644	FAM153B	family with sequence similarity 153, member B	ENST0000508296	ENSP00000424849	223	Q/P	cAg/cCg	-	0	0.287	NA	2.929	high_impact	-0.167	low_impact	NA	NA

4	Primary tumor	Private in primary tumor	19:8191415	FBN3	fibrillin 3	ENST00000270509	ENSP00000270509	831	R/W	Cgg/Tgg	-	0	0.999	2.045	2.145	high_impact	1.824	high_impact	0.813	low_impact
4	Liver metastases	Private in metastatic tumor	1:27939784	FGR	Gardner-Rasheed feline sarcoma viral (v-fgr) oncogene homolog	ENST00000374004	ENSP00000363116	443	S/P	Tcc/Ccc	-	0	0.999	4	1.992	medium_impact	1.866	high_impact	2.922	medium_impact
4	Liver metastases	Private in metastatic tumor	1:152278814	FLG	filaggrin	ENST00000368796	ENSP00000357786	112	G/S	Ggc/Agc	rs2184952	0.62	0.998	NA	-0.982	low_impact	1.515	high_impact	NA	NA
4	Liver metastases	Private in metastatic tumor	12:50039615	FMNL3	formin-like 3	ENST00000352151	ENSP00000344311	972	A/P	Gct/Cct	-	0	0.438	NA	2.122	high_impact	0.028	medium_impact	NA	NA
4	Liver metastases	Private in metastatic tumor	11:62402393	GANAB	glucosidase, alpha	ENST00000525994	ENSP00000434805	40	R/W	Cgg/Tgg	rs2276296	0	0.982	NA	2.215	high_impact	2.57	high_impact	NA	NA
4	Primary tumor	Private in primary tumor	1:155783537	GON4L	gon-4-like (C. elegans)	ENST00000539959	ENSP00000444059	447	G/A	gGg/gCg	-	0.08	0.998	NA	0.28	medium_impact	1.829	high_impact	NA	NA
4	Liver metastases	Private in metastatic tumor	10:101163286	GOT1	glutamic-oxaloacetic transaminase 1, soluble (aspartate aminotransferase 1)	ENST00000535447	ENSP00000440686	253	P/R	cCc/cGc	-	0	1	NA	2.132	high_impact	2.19	high_impact	NA	NA
4	Primary tumor	Common in primary and metastatic tumors	6:31630315	GPANK1	G patch domain and ankyrin repeats 1	ENST00000375893	ENSP00000365057	267	G/R	Ggc/Cgc	-	0	0.999	3.805	2.084	high_impact	1.992	high_impact	2.542	medium_impact
4	Liver metastases	Common in primary and metastatic	6:31630315	GPANK1	G patch domain and ankyrin repeats 1	ENST00000375893	ENSP00000365057	267	G/R	Ggc/Cgc	-	0	0.999	3.805	2.084	high_impact	1.992	high_impact	2.542	medium_impact

		tumors																		
4	Liver metastases	Private in metastatic tumor	X:153220581	HCFC1	host cell factor C1 (VP16-accessory protein)	ENST00000369984	ENSP00000359001	1090	T/S	aCc/aGc	-	0	0.808	NA	2.033	high_impact	0.467	medium_impact	NA	NA
4	Primary tumor	Private in primary tumor	5:137089679	HNRNPA0	heterogeneous nuclear ribonucleoprotein A0	ENST00000314940	ENSP00000316042	26	H/L	cAc/cTc	-	0	0.829	0.515	2.056	high_impact	0.597	medium_impact	-0.596	low_impact
4	Primary tumor	Private in primary tumor	5:137089673	HNRNPA0	heterogeneous nuclear ribonucleoprotein A0	ENST00000314940	ENSP00000316042	28	E/V	gAg/gTg	-	0	0.382	2.265	2.056	high_impact	0.093	medium_impact	1.215	medium_impact
4	Primary tumor	Common in primary and metastatic tumors	22:38823596	KCNJ4	potassium inwardly-rectifying channel, subfamily J, member 4	ENST00000303592	ENSP00000306497	181	R/Q	cGg/cAg	-	0	0.999	3.095	1.995	medium_impact	1.753	high_impact	1.843	medium_impact
4	Liver metastases	Common in primary and metastatic tumors	22:38823596	KCNJ4	potassium inwardly-rectifying channel, subfamily J, member 4	ENST00000303592	ENSP00000306497	181	R/Q	cGg/cAg	-	0	0.999	3.095	1.995	medium_impact	1.753	high_impact	1.843	medium_impact
4	Primary tumor	Private in primary tumor	21:45999653	KRTAP10-5	keratin associated protein 10-5	ENST00000400372	ENSP00000383223	268	R/P	cGc/cCc	rs464391	0	0.998	2.6	2.929	high_impact	1.613	high_impact	1.859	medium_impact
4	Liver metastases	Private in metastatic tumor	21:46086731	KRTAP12-2	keratin associated protein 12-2	ENST00000539483	ENSP00000439531	25	C/R	Tgc/Cgc	-	0	0	NA	2.929	high_impact	-1.667	low_impact	NA	NA
4	Primary tumor	Private in primary tumor	15:74219546	LOXL1	lysyl oxidase-like 1	ENST00000395162	ENSP00000378591	3	R/L	cGg/cTg	rs1048661	0	0.944	NA	2.235	high_impact	0.329	medium_impact	NA	NA
4	Primary tumor	Private in primary tumor	1:82447538	LPHN2	latrophilin 2	ENST00000335786	ENSP00000337306	1050	T/A	Acc/Gcc	-	0.0	0.993	3.385	1.32	medium_impact	2.033	high_impact	4.354	high_impact

												1								
4	Primary tumor	Private in primary tumor	12:85432040	LRRIQ1	leucine-rich repeats and IQ motif containing 1	ENST0000393217	ENSP0000376910	29	I/N	aTt/aAt	rs7312075	0	0.363	NA	2.062	high_impact	0.068	medium_impact	NA	NA
4	Primary tumor	Private in primary tumor	14:24785656	LTB4R	leukotriene B4 receptor	ENST0000396789	ENSP0000380008	267	R/C	Cgc/Tgc	-	0.01	1	2.835	1.711	medium_impact	2.18	high_impact	1.768	medium_impact
4	Primary tumor	Private in primary tumor	15:41804049	LTK	leukocyte receptor tyrosine kinase	ENST0000355166	ENSP0000347293	208	G/D	gGc/gAc	-	0	0.998	NA	2.223	high_impact	1.733	high_impact	NA	NA
4	Liver metastases	Private in metastatic tumor	4:156289917	MAP9	microtubule-associated protein 9	ENST0000515654	ENSP0000427402	177	R/W	Cgg/Tgg	rs3733391	0	0.998	NA	2.929	high_impact	1.613	high_impact	NA	NA
4	Liver metastases	Private in metastatic tumor	3:50654997	MAPKAPK3	mitogen-activated protein kinase-activated protein kinase 3	ENST0000430409	ENSP0000410970	1	M/V	Atg/Gtg	-	0	0.007	NA	2.322	high_impact	-1.069	low_impact	NA	NA
4	Primary tumor	Private in primary tumor	6:30672332	MDC1	mediator of DNA-damage checkpoint 1	ENST0000376406	ENSP0000365588	1543	T/R	aCa/aGa	-	0.02	0.974	2.175	0.916	medium_impact	1.087	medium_impact	3.656	high_impact
4	Primary tumor	Private in primary tumor	6:30672335	MDC1	mediator of DNA-damage checkpoint 1	ENST0000420817	ENSP0000411775	190	P/A	Cca/Gca	-	0	0	NA	2.082	high_impact	-1.654	low_impact	NA	NA
4	Liver metastases	Private in metastatic tumor	6:30672257	MDC1	mediator of DNA-damage checkpoint 1	ENST0000422104	ENSP0000390375	1134	P/H	cCt/cAt	-	0	0.999	NA	2.082	high_impact	1.942	high_impact	NA	NA
4	Liver metastases	Private in metastatic tumor	6:30672257	MDC1	mediator of DNA-damage checkpoint 1	ENST0000420817	ENSP0000411775	216	L/M	Ctg/Atg	-	0	0	NA	2.082	high_impact	-1.654	low_impact	NA	NA

4	Primary tumor	Private in primary tumor	6:30672335	MDC1	mediator of DNA-damage checkpoint 1	ENST00000376405	ENSP00000365587	1278	S/C	tCc/tGc	-	0	0.948	NA	2.082	high_impact	0.9	medium_impact	NA	NA
4	Primary tumor	Common in primary and metastatic tumors	17:27438469	MYO18A	myosin XVIIIA	ENST00000527372	ENSP00000437073	958	A/V	gCc/gTc	rs8076604	0.05	0.997	NA	0.444	medium_impact	1.502	high_impact	NA	NA
4	Liver metastases	Common in primary and metastatic tumors	17:27438469	MYO18A	myosin XVIIIA	ENST00000354682	ENSP00000346713	627	A/V	gCc/gTc	rs8076604	0	0.681	NA	2.001	high_impact	0.265	medium_impact	NA	NA
4	Primary tumor	Private in primary tumor	16:3614345	NLRC3	NLR family, CARD domain containing 3	ENST00000359128	ENSP00000352039	198	F/S	tTc/tCc	-	0	0.839	NA	2.064	high_impact	0.607	medium_impact	NA	NA
4	Liver metastases	Private in metastatic tumor	16:136855	NPRL3	nitrogen permease regulator-like 3 (S. cerevisiae)	ENST00000399953	ENSP00000382834	519	F/Y	tTc/tAc	-	0.01	0.996	NA	1.128	medium_impact	1.566	high_impact	NA	NA
4	Primary tumor	Common in primary and metastatic tumors	16:56871605	NUP93	nucleoporin 93kDa	ENST00000308159	ENSP00000310668	662	R/K	aGg/aAg	-	0	0.997	2.69	2.062	high_impact	1.625	high_impact	1.755	medium_impact
4	Liver metastases	Common in primary and metastatic tumors	16:56871605	NUP93	nucleoporin 93kDa	ENST00000308159	ENSP00000310668	662	R/K	aGg/aAg	-	0	0.997	2.69	2.062	high_impact	1.625	high_impact	1.755	medium_impact
4	Primary tumor	Private in primary tumor	1:248844959	OR14I1	olfactory receptor, family 14, subfamily I, member 1	ENST00000342623	ENSP00000339726	216	Y/C	tAt/tGt	rs55871516	0	1	4.43	1.67	medium_impact	1.724	high_impact	1.913	medium_impact
4	Prima	Common in	21:4780	PC	pericentrin	ENST0	ENSP00	1163	R/	Cgc/	rs727	0	1	1.935	2.438	high_im	2.056	high_impa	0.906	low_impact

	ry tumor	primary and metastatic tumors	8679	NT		0000359568	000352572		C	Tgc	9204					pact		ct		
4	Liver metastases	Common in primary and metastatic tumors	21:47808679	PCNT	pericentrin	ENST00000359568	ENSP00000352572	1163	R/C	Cgc/Tgc	rs7279204	0	1	1.935	2.438	high_impact	2.056	high_impact	0.906	low_impact
4	Primary tumor	Private in primary tumor	1:144880814	PDE4DIP	phosphodiesterase 4D interacting protein	ENST00000530740	ENSP00000435654	1408	L/F	Ctc/Ttc	rs1747960	0.001	0.996	NA	1.068	medium_impact	1.521	high_impact	NA	NA
4	Primary tumor	Private in primary tumor	12:76425160	PHLDA1	pleckstrin homology-like domain, family A, member 1	ENST00000266671	ENSP00000266671	121	P/R	cCg/cGg	-	0	0.975	1.04	2.062	high_impact	1.101	medium_impact	0.024	low_impact
4	Liver metastases	Private in metastatic tumor	17:37224211	PLXDC1	plexin domain containing 1	ENST00000444911	ENSP00000409687	422	R/H	cGt/cAt	rs75117355	0.37	0.997	NA	-0.516	low_impact	1.637	high_impact	NA	NA
4	Liver metastases	Private in metastatic tumor	8:128428245	POU5F1B	POU class 5 homeobox 1B	ENST00000391675	ENSP00000375557	45	P/R	cCa/cGa	-	0	0.106	1.78	2.056	high_impact	-0.34	low_impact	0.705	low_impact
4	Primary tumor	Private in primary tumor	X:114426145	RBMXL3	RNA binding motif protein, X-linked-like 3	ENST00000424776	ENSP00000417451	714	H/R	cAc/cGc	-	0	0	0	2.056	high_impact	-1.483	low_impact	-1.129	low_impact
4	Liver metastases	Private in metastatic tumor	1:24859618	RCAN3	RCAN family member 3	ENST00000538532	ENSP00000445401	81	P/T	Ccc/Acc	-	0	1	NA	1.631	medium_impact	1.515	high_impact	NA	NA
4	Primary tumor	Common in primary and metastatic	5:96513471	RIOK2	RIO kinase 2 (yeast)	ENST00000508447	ENSP00000420932	96	S/C	tCt/tGt	rs2544773	0	0.998	NA	2.008	high_impact	1.861	high_impact	NA	NA

		tumors																		
4	Liver metastases	Common in primary and metastatic tumors	5:96513471	RIOK2	RIO kinase 2 (yeast)	ENST00000508447	ENSP00000420932	96	S/C	tCt/tGt	rs2544773	0	0.998	NA	2.008	high_impact	1.861	high_impact	NA	NA
4	Primary tumor	Private in primary tumor	16:30776791	RNF40	ring finger protein 40	ENST00000324685	ENSP00000325677	322	G/W	Ggg/Tgg	-	0.01	0.988	2.36	1.53	medium_impact	1.598	high_impact	1.361	medium_impact
4	Liver metastases	Private in metastatic tumor	19:38996563	RYR1	ryanodine receptor 1 (skeletal)	ENST00000359596	ENSP00000352608	2840	R/W	Cgg/Tgg	CM064237	NA	0.998	2.52	NA	NA	1.557	high_impact	0.531	low_impact
4	Liver metastases	Private in metastatic tumor	14:21991967	SALL2	sal-like 2 (Drosophila)	ENST00000546363	ENSP00000440054	491	Q/L	cAg/cTg	-	0	0.782	NA	2.089	high_impact	0.542	medium_impact	NA	NA
4	Primary tumor	Private in primary tumor	19:50154971	SCAF1	SR-related CTD-associated factor 1	ENST00000360565	ENSP00000353769	442	E/G	gAg/gGg	-	0	0	0.55	2.01	high_impact	-1.543	low_impact	-0.459	low_impact
4	Primary tumor	Common in primary and metastatic tumors	1:169673838	SELL	selectin L	ENST00000236147	ENSP00000236147	226	P/S	Cct/Tct	rs2229569	0.05	0.999	NA	0.608	medium_impact	1.81	high_impact	NA	NA
4	Liver metastases	Common in primary and metastatic tumors	1:169673838	SELL	selectin L	ENST00000236147	ENSP00000236147	226	P/S	Cct/Tct	rs2229569	0.05	0.999	NA	0.608	medium_impact	1.81	high_impact	NA	NA
4	Liver metastases	Private in metastatic tumor	6:100838568	SIM1	single-minded homolog 1 (Drosophila)	ENST00000262901	ENSP00000262901	657	R/Q	cGg/cAg	-	0	0.966	0.695	2.056	high_impact	1.038	medium_impact	-0.367	low_impact
4	Liver	Private in	22:3148	SM	smoothelin	ENST0	ENSP00	292	G/	Gga/	-	0	0.0	NA	2.062	high_im	-1.089	low_impa	NA	NA

	metastases	metastatic tumor	6802	TN		0000329852	000329393		R	Cga			05			pact		ct		
4	Liver metastases	Private in metastatic tumor	1:118514634	SPAG17	sperm associated antigen 17	ENST00000437255	ENSP00000402749	540	S/T	Tct/Act	-	0	0.993	NA	2.929	high_impact	1.299	medium_impact	NA	NA
4	Liver metastases	Private in metastatic tumor	2:220337041	SPEG	SPEG complex locus	ENST00000265327	ENSP00000265327	1310	D/N	Gac/Aac	rs116911250	0.002	0.997	NA	0.831	medium_impact	1.627	high_impact	NA	NA
4	Primary tumor	Private in primary tumor	9:131388058	SPTAN1	spectrin, alpha, non-erythrocytic 1 (alpha-fodrin)	ENST00000372719	ENSP00000361804	2002	D/V	gAc/gTc	-	0	0.981	NA	2.111	high_impact	1.154	medium_impact	NA	NA
4	Primary tumor	Common in primary and metastatic tumors	6:10931678	SYCP2L	synaptonemal complex protein 2-like	ENST00000283141	ENSP00000283141	547	P/A	Cca/Gca	-	0	0.07	2.095	2.929	high_impact	-0.587	low_impact	1.306	medium_impact
4	Liver metastases	Common in primary and metastatic tumors	6:10931678	SYCP2L	synaptonemal complex protein 2-like	ENST00000283141	ENSP00000283141	547	P/A	Cca/Gca	-	0	0.07	2.095	2.929	high_impact	-0.587	low_impact	1.306	medium_impact
4	Liver metastases	Private in metastatic tumor	11:119290839	THY1	Thy-1 cell surface antigen	ENST00000527590	ENSP00000433689	107	E/K	Gag/Aag	-	0.007	0.998	NA	0.611	medium_impact	2.016	high_impact	NA	NA
4	Primary tumor	Private in primary tumor	10:98144400	TLL2	tolloid-like 2	ENST00000357947	ENSP00000350630	713	E/G	gAg/gGg	-	0	0.999	2.4	2.174	high_impact	1.85	high_impact	1.288	medium_impact
4	Liver metastases	Private in metastatic tumor	17:7578406	TP53	tumor protein p53	ENST00000396473	ENSP00000379735	164	R/H	cGc/cAc	rs28934578	0	1	NA	2.265	high_impact	2.147	high_impact	NA	NA

4	Liver metastases	Private in metastatic tumor	11:118889604	TRAPPC4	trafficking protein particle complex 4	ENST00000533058	ENSP00000432920	33	S/R	agT/agA	-	0	0.838	NA	2.062	high_impact	0.607	medium_impact	NA	NA
4	Primary tumor	Common in primary and metastatic tumors	1:154525245	UBE2Q1	ubiquitin-conjugating enzyme E2Q family member 1	ENST00000292211	ENSP00000292211	261	R/W	Agg/Tgg	-	0	1	2.675	2.114	high_impact	2.205	high_impact	1.553	medium_impact
4	Liver metastases	Common in primary and metastatic tumors	1:154525245	UBE2Q1	ubiquitin-conjugating enzyme E2Q family member 1	ENST00000292211	ENSP00000292211	261	R/W	Agg/Tgg	-	0	1	2.675	2.114	high_impact	2.205	high_impact	1.553	medium_impact
4	Primary tumor	Common in primary and metastatic tumors	2:135744416	YSK4	YSK4 Sps1/Ste20-related kinase homolog (S. cerevisiae)	ENST00000392915	ENSP00000376647	693	E/Q	Gag/Cag	rs1112542	0	0.812	NA	2.032	high_impact	0.576	medium_impact	NA	NA
4	Liver metastases	Common in primary and metastatic tumors	2:135744416	YSK4	YSK4 Sps1/Ste20-related kinase homolog (S. cerevisiae)	ENST00000437365	ENSP00000392827	66	E/Q	Gag/Cag	rs1112542	0	0.788	NA	2.032	high_impact	0.54	medium_impact	NA	NA
4	Liver metastases	Private in metastatic tumor	20:39832235	ZHX3	zinc fingers and homeoboxes 3	ENST00000421422	ENSP00000405421	150	S/F	tCc/tTc	-	0	0.165	NA	2.051	high_impact	-0.199	low_impact	NA	NA
4	Liver metastases	Private in metastatic tumor	19:21606429	ZNF493	zinc finger protein 493	ENST00000355504	ENSP00000347691	195	C/F	tGt/tTt	rs4621113	0	0.985	3.495	2.084	high_impact	1.307	medium_impact	2.255	medium_impact
4	Primary tumor	Common in primary and metastatic tumors	19:52919972	ZNF528	zinc finger protein 528	ENST00000360465	ENSP00000353652	623	H/Y	Cat/Tat	rs77428121	0	0.947	3.715	2.084	high_impact	0.98	medium_impact	2.459	medium_impact
4	Liver	Common in	19:5291	ZNF	zinc finger protein 528	ENST0	ENSP00	623	H/	Cat/T	rs774	0	0.9	3.715	2.084	high_im	0.98	medium_i	2.459	medium_imp

	metastases	primary and metastatic tumors	9972	528		0000360465	000353652		Y	at	28121		47			pact		mpact		act
5	Liver metastases	Private in metastatic tumor	7:150553910	ABP1	amiloride binding protein 1 (amine oxidase (copper-containing))	ENST00000416793	ENSP0000411613	118	P/A	Ccc/Gcc	-	0	0.93	NA	2.219	high_impact	0.837	medium_impact	NA	NA
5	Liver metastases	Private in metastatic tumor	7:44146224	AEBP1	AE binding protein 1	ENST00000223357	ENSP0000223357	111	E/D	gaG/gaC	-	0	0	0.895	2.042	high_impact	-1.287	low_impact	-0.263	low_impact
5	Liver metastases	Private in metastatic tumor	11:94603952	AMOTL1	angiomotin like 1	ENST00000317837	ENSP0000323474	542	L/I	Ctc/Atc	-	0	0	NA	2.02	high_impact	-1.457	low_impact	NA	NA
5	Liver metastases	Private in metastatic tumor	2:97505297	ANKRD23	ankyrin repeat domain 23	ENST00000418232	ENSP0000398987	303	T/P	Acc/Ccc	rs74826568	0	0.698	0.695	2.062	high_impact	0.41	medium_impact	-0.338	low_impact
5	Liver metastases	Private in metastatic tumor	12:5853445	ANO2	anoctamin 2	ENST00000541277	ENSP0000437754	411	C/S	tGt/tCt	-	0	0.999	NA	2.124	high_impact	1.808	high_impact	NA	NA
5	Primary tumor	Private in primary tumor	11:430396	ANO9	anoctamin 9	ENST00000332826	ENSP0000332788	183	F/L	Ttt/Ctt	-	0	0.496	2.67	2.056	high_impact	0.249	medium_impact	1.553	medium_impact
5	Primary tumor	Private in primary tumor	22:29727891	AP1B1	adaptor-related protein complex 1, beta 1 subunit	ENST00000405198	ENSP0000384194	775	P/R	cCc/cGc	-	0	1	3.405	2.035	high_impact	1.851	high_impact	1.873	medium_impact
5	Liver metastases	Private in metastatic tumor	X:1755398	ASMT	acetylserotonin O-methyltransferase	ENST00000432523	ENSP0000392053	36	W/C	tgG/tgT	-	0	0.998	NA	2.52	high_impact	2.564	high_impact	NA	NA
5	Liver metastases	Private in metastatic tumor	4:47574207	ATP10D	ATPase, class V, type 10D	ENST00000273859	ENSP0000273859	1067	A/E	gCa/gAa	-	0	0.967	4.76	2.189	high_impact	0.963	medium_impact	3.289	high_impact

5	Liver metastases	Private in metastatic tumor	17:41222964	BRCA1	breast cancer 1, early onset	ENST00000478531	ENSP00000420412	552	G/A	gGc/gCc	rs80357414	0.007	0.997	NA	-0.013	low_impact	1.713	high_impact	NA	NA
5	Primary tumor	Private in primary tumor	17:79203065	C17orf56	chromosome 17 open reading frame 56	ENST00000300714	ENSP00000300714	414	P/H	cCt/cAt	-	0	0.86	NA	2.929	high_impact	0.514	medium_impact	NA	NA
5	Liver metastases	Private in metastatic tumor	20:62715548	C20orf201	chromosome 20 open reading frame 201	ENST00000308906	ENSP00000310801	9	G/W	Ggg/Tgg	rs4431000	0	0.985	NA	2.929	high_impact	1.106	medium_impact	NA	NA
5	Primary tumor	Common in primary and metastatic tumors	1:181680146	CACNA1E	calcium channel, voltage-dependent, R type, alpha 1E subunit	ENST00000360108	ENSP00000353222	371	R/Q	cGg/cAg	-	0	0.994	NA	2.318	high_impact	1.396	medium_impact	NA	NA
5	Liver metastases	Common in primary and metastatic tumors	1:181680146	CACNA1E	calcium channel, voltage-dependent, R type, alpha 1E subunit	ENST00000367573	ENSP00000356545	371	R/Q	cGg/cAg	-	0	0.983	2.565	2.318	high_impact	1.14	medium_impact	2.1	medium_impact
5	Primary tumor	Common in primary and metastatic tumors	10:18690862	CACNB2	calcium channel, voltage-dependent, beta 2 subunit	ENST00000377319	ENSP00000366536	20	D/N	Gac/Aac	-	0	0.992	NA	2.318	high_impact	1.326	medium_impact	NA	NA
5	Liver metastases	Common in primary and metastatic tumors	10:18690862	CACNB2	calcium channel, voltage-dependent, beta 2 subunit	ENST00000377329	ENSP00000366546	21	D/N	Gac/Aac	-	0	0.992	NA	2.318	high_impact	1.326	medium_impact	NA	NA
5	Liver metastases	Private in metastatic tumor	12:54107541	CALCOCO1	calcium binding and coiled-coil domain 1	ENST00000413257	ENSP00000399426	517	P/T	Ccg/Acg	-	0.001	0.999	NA	1.242	medium_impact	1.843	high_impact	NA	NA

5	Liver metastases	Private in metastatic tumor	5:79031558	CMYA5	cardiomyopathy associated 5	ENST0000446378	ENSP00000394770	2324	G/S	Ggt/Agt	rs62621912	0	0.995	2.175	2.02	high_impact	1.555	high_impact	1.124	medium_impact
5	Liver metastases	Private in metastatic tumor	17:48275136	COL1A1	collagen, type I, alpha 1	ENST0000225964	ENSP00000225964	218	G/A	gGt/gCt	-	0	0	4.085	2.263	high_impact	-2.068	low_impact	2.504	medium_impact
5	Primary tumor	Private in primary tumor	2:189916099	COL5A2	collagen, type V, alpha 2	ENST0000452536	ENSP00000398603	600	G/R	Ggc/Cgc	-	NA	0.998	NA	NA	NA	1.611	high_impact	NA	NA
5	Primary tumor	Common in primary and metastatic tumors	1:225525089	DNAH14	dynein, axonemal, heavy chain 14	ENST0000430092	ENSP00000414402	3308	W/L	tGg/tTg	-	0	0.978	NA	2.056	high_impact	1.062	medium_impact	NA	NA
5	Liver metastases	Common in primary and metastatic tumors	1:225525089	DNAH14	dynein, axonemal, heavy chain 14	ENST0000439375	ENSP00000392061	3308	W/L	tGg/tTg	-	0	0.978	NA	2.056	high_impact	1.062	medium_impact	NA	NA
5	Primary tumor	Private in primary tumor	1:38184055	EPHA10	EPH receptor A10	ENST0000330210	ENSP00000330379	501	V/M	Gtg/Atg	rs78757428	0	0.94	NA	2.386	high_impact	0.705	medium_impact	NA	NA
5	Liver metastases	Private in metastatic tumor	15:41476236	EXD1	exonuclease 3'-5' domain containing 1	ENST0000314992	ENSP00000321029	480	S/C	Agt/Tgt	-	0	0.844	2.015	2.092	high_impact	0.781	medium_impact	1.037	medium_impact
5	Liver metastases	Private in metastatic tumor	1:184868431	FAM129A	family with sequence similarity 129, member A	ENST0000367511	ENSP00000356481	23	A/T	Gct/Act	-	0.2	0.998	2.045	-0.171	low_impact	1.738	high_impact	1.097	medium_impact
5	Liver metastases	Private in metastatic tumor	4:47408705	GABRB1	gamma-aminobutyric acid (GABA) A receptor, beta 1	ENST0000295454	ENSP00000295454	281	T/M	aCg/aTg	-	0.03	0.999	2.51	0.72	medium_impact	1.696	high_impact	1.24	medium_impact

5	Primary tumor	Private in primary tumor	19:48205418	GLTSCR1	glioma tumor suppressor candidate region gene 1	ENST00000396720	ENSP00000379946	1477	S/A	Tcc/Gcc	-	0	0.901	0.975	2.062	high_impact	0.745	medium_impact	-0.044	low_impact
5	Liver metastases	Private in metastatic tumor	8:37690604	GPR124	G protein-coupled receptor 124	ENST00000416514	ENSP00000405145	385	G/R	Ggg/Cgg	-	0	0.99	NA	2.048	high_impact	1.383	medium_impact	NA	NA
5	Liver metastases	Private in metastatic tumor	1:226253443	H3F3A	H3 histone, family 3A	ENST00000366814	ENSP00000355779	72	V/A	gTg/gCg	-	0	0.266	NA	2.064	high_impact	0	medium_impact	NA	NA
5	Primary tumor	Private in primary tumor	15:73614899	HCN4	hyperpolarization activated cyclic nucleotide-gated potassium channel 4	ENST00000261917	ENSP00000261917	1179	P/S	Cct/Tct	-	0	0.239	1.04	2.072	high_impact	-0.076	low_impact	0.03	low_impact
5	Liver metastases	Private in metastatic tumor	19:40886465	HIPK4	homeodomain interacting protein kinase 4	ENST00000452139	ENSP00000397736	443	Y/S	tAc/tCc	-	0	0.955	NA	2.032	high_impact	0.962	medium_impact	NA	NA
5	Liver metastases	Private in metastatic tumor	6:34211288	HMGA1	high mobility group AT-hook 1	ENST00000347617	ENSP00000288245	77	K/R	aAa/aGa	-	0	0	NA	2.039	high_impact	-1.648	low_impact	NA	NA
5	Liver metastases	Private in metastatic tumor	16:71127814	HYDIN	hydrocephalus inducing homolog (mouse)	ENST00000448089	ENSP00000398544	451	R/P	cGa/cCa	rs7200485	0.210	0.998	NA	-0.189	low_impact	1.613	high_impact	NA	NA
5	Liver metastases	Private in metastatic tumor	7:50367288	IKZF1	IKAROS family zinc finger 1 (Ikaros)	ENST00000331340	ENSP00000331614	32	P/L	cCg/cTg	-	0.030	0.998	[sent]	0.545	medium_impact	1.791	high_impact	NA	NA
5	Liver metastases	Private in metastatic tumor	3:12957102	IQSEC1	IQ motif and Sec7 domain 1	ENST00000450726	ENSP00000397811	733	K/E	Aaa/Gaa	-	0.02	0.993	NA	0.639	medium_impact	1.544	high_impact	NA	NA

5	Liver metastases	Private in metastatic tumor	X:153282413	IRAK1	interleukin-1 receptor-associated kinase 1	ENST00000443220	ENSP00000403730	110	G/R	Gga/Cga	-	0.36	0.999	NA	-0.459	low_impact	1.87	high_impact	NA	NA
5	Liver metastases	Private in metastatic tumor	10:102822575	KAZALD1	Kazal-type serine peptidase inhibitor domain 1	ENST00000370199	ENSP00000359218	76	C/G	Tgc/Ggc	rs11547671	0	0.999	NA	2.277	high_impact	1.841	high_impact	NA	NA
5	Primary tumor	Private in primary tumor	17:38953472	KRT28	keratin 28	ENST00000306658	ENSP00000305263	251	P/R	cCg/cGg	-	0	1	2.945	2.018	high_impact	1.682	high_impact	1.625	medium_impact
5	Liver metastases	Private in metastatic tumor	6:30673103	MDC1	mediator of DNA-damage checkpoint 1	ENST00000376406	ENSP00000365588	1286	P/R	cCc/cGc	-	0.02	0.988	2.135	0.916	medium_impact	1.292	medium_impact	3.569	high_impact
5	Primary tumor	Common in primary and metastatic tumors	4:187455522	MTNR1A	melatonin receptor 1A	ENST00000307161	ENSP00000302811	125	R/H	cGc/cAc	-	0	1	4.55	1.948	medium_impact	1.856	high_impact	2.856	medium_impact
5	Liver metastases	Common in primary and metastatic tumors	4:187455522	MTNR1A	melatonin receptor 1A	ENST00000307161	ENSP00000302811	125	R/H	cGc/cAc	-	0	1	4.55	1.948	medium_impact	1.856	high_impact	2.856	medium_impact
5	Primary tumor	Common in primary and metastatic tumors	17:48594772	MYCBPAP	MYCBP associated protein	ENST00000323776	ENSP00000323184	151	P/L	cCt/cTt	-	0.02	0.999	NA	0.856	medium_impact	1.894	high_impact	NA	NA
5	Liver metastases	Common in primary and metastatic tumors	17:48594772	MYCBPAP	MYCBP associated protein	ENST00000436259	ENSP00000397209	114	P/L	cCt/cTt	-	0.02	0.995	2.515	0.856	medium_impact	1.5	high_impact	1.571	medium_impact

5	Primary tumor	Common in primary and metastatic tumors	1:1007222	NA	NA	ENST0000453464	ENSP0000410533	242	P/H	cCc/cAc	rs71628928	0	0	NA	2.069	high_impact	-1.491	low_impact	NA	NA
5	Liver metastases	Common in primary and metastatic tumors	1:1007222	NA	NA	ENST0000453464	ENSP0000410533	242	P/H	cCc/cAc	rs71628928	0	0	NA	2.069	high_impact	-1.491	low_impact	NA	NA
5	Liver metastases	Private in metastatic tumor	20:62193062	NA	NA	ENST0000427522	ENSP0000393257	1674	R/H	cGt/cAt	-	0	0.998	NA	1.92	medium_impact	1.597	high_impact	NA	NA
5	Primary tumor	Private in primary tumor	17:15517237	NA	NA	ENST0000455584	ENSP0000402644	586	L/V	Ctg/Gtg	rs62070406	0	0	NA	2.064	high_impact	-1.516	low_impact	NA	NA
5	Liver metastases	Private in metastatic tumor	11:113146050	NCAM1	neural cell adhesion molecule 1	ENST0000433634	ENSP0000390982	295	E/K	Gaa/Aaa	-	0	0.967	NA	2.062	high_impact	1.031	medium_impact	NA	NA
5	Liver metastases	Private in metastatic tumor	11:5758062	OR56B1	olfactory receptor, family 56, subfamily B, member 1	ENST0000317121	ENSP0000322939	106	C/R	Tgc/Cgc	rs7397032	0	1	4	1.67	medium_impact	1.724	high_impact	1.629	medium_impact
5	Primary tumor	Common in primary and metastatic tumors	10:102777868	PDZD7	PDZ domain containing 7	ENST0000370215	ENSP0000359234	504	D/N	Gac/Aac	-	0	0.238	0.975	2.062	high_impact	-0.079	low_impact	-0.044	low_impact
5	Liver metastases	Common in primary and metastatic tumors	10:102777868	PDZD7	PDZ domain containing 7	ENST0000370215	ENSP0000359234	504	D/N	Gac/Aac	-	0	0.238	0.975	2.062	high_impact	-0.079	low_impact	-0.044	low_impact
5	Liver metas	Private in metastatic	16:2159349	PKD1	polycystic kidney disease 1 (autosomal dominant)	ENST000003	ENSP0003719	219	F/S	tTc/tCc	-	0.	0.996	NA	0.245	medium_imp	1.531	high_impact	NA	NA

	tases	tumor				82481	21					08				act				
5	Liver metastases	Private in metastatic tumor	19:4513269	PLIN4	perilipin 4	ENST00000301286	ENSP00000301286	221	K/E	Aaa/Gaa	-	0.33	0.997	2.48	-0.534	low_impact	1.512	high_impact	1.727	medium_impact
5	Primary tumor	Common in primary and metastatic tumors	2:44571067	PREPL	prolyl endopeptidase-like	ENST00000438314	ENSP00000395034	145	L/I	Tta/Ata	-	0	0.743	NA	2.111	high_impact	0.4	medium_impact	NA	NA
5	Liver metastases	Common in primary and metastatic tumors	2:44571067	PREPL	prolyl endopeptidase-like	ENST00000438314	ENSP00000395034	145	L/I	Tta/Ata	-	0	0.743	NA	2.111	high_impact	0.4	medium_impact	NA	NA
5	Liver metastases	Private in metastatic tumor	12:70988289	PTPRB	protein tyrosine phosphatase, receptor type, B	ENST00000451516	ENSP00000393028	274	A/T	Gct/Act	-	0.04	0.998	2.135	0.598	medium_impact	1.799	high_impact	1.065	medium_impact
5	Primary tumor	Private in primary tumor	X:114426361	RBMXL3	RNA binding motif protein, X-linked-like 3	ENST00000424776	ENSP00000417451	786	S/L	tCg/tTg	-	0	0.403	0	2.056	high_impact	0.114	medium_impact	-1.129	low_impact
5	Primary tumor	Private in primary tumor	7:150069226	REPIN1	replication initiator 1	ENST00000488943	ENSP00000419872	359	R/H	cGc/cAc	-	0	0.996	NA	2.056	high_impact	1.584	high_impact	NA	NA
5	Primary tumor	Common in primary and metastatic tumors	16:111621	RHBDF1	rhomboid 5 homolog 1 (Drosophila)	ENST00000454039	ENSP00000392133	428	A/T	Gcg/Acg	-	0.01	0.998	NA	1.156	medium_impact	1.686	high_impact	NA	NA
5	Liver metas	Common in primary and	16:111621	RHBDF	rhomboid 5 homolog 1 (Drosophila)	ENST000004	ENSP000003921	428	A/T	Gcg/Acg	-	0.	0.998	NA	1.156	medium_imp	1.686	high_impact	NA	NA

	tases	metastatic tumors		1		54039	33					0 1				act				
5	Primary tumor	Common in primary and metastatic tumors	1:156347834	RHBG	Rh family, B glycoprotein (gene/pseudogene)	ENST00000368246	ENSP00000357229	143	V/D	gTc/gAc	rs11586833	0	0.391	NA	2.268	high_impact	0.029	medium_impact	NA	NA
5	Liver metastases	Common in primary and metastatic tumors	1:156347834	RHBG	Rh family, B glycoprotein (gene/pseudogene)	ENST00000368246	ENSP00000357229	143	V/D	gTc/gAc	rs11586833	0	0.391	NA	2.268	high_impact	0.029	medium_impact	NA	NA
5	Primary tumor	Common in primary and metastatic tumors	17:78319717	RNF213	ring finger protein 213	ENST00000336301	ENSP00000338218	601	R/W	Cgg/Tgg	-	0	1	NA	2.011	high_impact	1.832	high_impact	NA	NA
5	Liver metastases	Common in primary and metastatic tumors	17:78319717	RNF213	ring finger protein 213	ENST00000336301	ENSP00000338218	601	R/W	Cgg/Tgg	-	0	1	NA	2.011	high_impact	1.832	high_impact	NA	NA
5	Liver metastases	Private in metastatic tumor	14:91444710	RPS6KA5	ribosomal protein S6 kinase, 90kDa, polypeptide 5	ENST00000536315	ENSP00000442803	33	P/S	Cca/Tca	-	0	1	NA	2.204	high_impact	1.921	high_impact	NA	NA
5	Primary tumor	Private in primary tumor	7:92764032	SAMD9L	sterile alpha motif domain containing 9-like	ENST00000411955	ENSP00000405760	418	I/T	aTt/aCt	-	0	0.66	2.045	2.062	high_impact	0.367	medium_impact	1.078	medium_impact
5	Liver metastases	Private in metastatic tumor	1:67895901	SERBP1	SERPINE1 mRNA binding protein 1	ENST00000370990	ENSP00000360029	28	F/S	tTc/tCc	-	0	0.904	NA	2.339	high_impact	1.02	medium_impact	NA	NA
5	Liver metastases	Private in metastatic tumor	6:100896395	SIM1	single-minded homolog 1 (Drosophila)	ENST00000262901	ENSP00000262901	235	R/C	Cgc/Tgc	-	0	1	3.775	2.056	high_impact	1.933	high_impact	2.675	medium_impact

5	Primary tumor	Private in primary tumor	4:985442	SLC26A1	solute carrier family 26 (sulfate transporter), member 1	ENST00000398520	ENSP00000381532	17	V/G	gTc/gGc	-	0	0.433	NA	2.356	high_impact	0.215	medium_impact	NA	NA
5	Primary tumor	Common in primary and metastatic tumors	17:18220352	SMCR8	Smith-Magenis syndrome chromosome region, candidate 8	ENST00000406438	ENSP00000385025	417	S/T	Tct/Act	-	0	0.953	1.905	2.929	high_impact	0.813	medium_impact	1.098	medium_impact
5	Liver metastases	Common in primary and metastatic tumors	17:18220352	SMCR8	Smith-Magenis syndrome chromosome region, candidate 8	ENST00000406438	ENSP00000385025	417	S/T	Tct/Act	-	0	0.953	1.905	2.929	high_impact	0.813	medium_impact	1.098	medium_impact
5	Liver metastases	Private in metastatic tumor	3:140785199	SPSB4	splA/ryanodine receptor domain and SOCS box containing 4	ENST00000310546	ENSP00000311609	85	D/N	Gac/Aac	-	0.01	0.998	2.885	1.137	medium_impact	1.724	high_impact	1.959	medium_impact
5	Liver metastases	Private in metastatic tumor	17:36720413	SRCIN1	SRC kinase signaling inhibitor 1	ENST00000398579	ENSP00000381584	15	R/H	cGc/cAc	-	0	1	NA	2.128	high_impact	1.903	high_impact	NA	NA
5	Liver metastases	Private in metastatic tumor	X:153050572	SRPK3	SRSF protein kinase 3	ENST00000458681	ENSP00000406166	88	I/T	aTc/aCc	-	0	0.762	NA	2.032	high_impact	0.504	medium_impact	NA	NA
5	Liver metastases	Private in metastatic tumor	7:99786076	STAG3	stromal antigen 3	ENST00000317296	ENSP00000319318	145	G/V	gGc/gTc	-	0	0.781	2.76	2.117	high_impact	0.511	medium_impact	1.68	medium_impact
5	Primary tumor	Common in primary and metastatic tumors	1:6638781	TAS1R1	taste receptor, type 1, member 1	ENST00000437392	ENSP00000390751	223	R/C	Cgc/Tgc	-	0.01	1	NA	1.061	medium_impact	1.662	high_impact	NA	NA
5	Liver metas	Common in primary and	1:6638781	TAS1R1	taste receptor, type 1, member 1	ENST000003	ENSP000003125	301	R/C	Cgc/Tgc	-	0	1	NA	1.987	medium_imp	1.662	high_impact	NA	NA

	tases	metastatic tumors				51136	58									act				
5	Liver metastases	Private in metastatic tumor	11:67171803	TBC1D10C	TBC1 domain family, member 10C	ENST00000542590	ENSP00000443654	44	G/W	Ggg/Tgg	-	0.04	0.995	NA	0.635	medium_impact	1.537	high_impact	NA	NA
5	Liver metastases	Private in metastatic tumor	7:139717533	TBXAS1	thromboxane A synthase 1 (platelet)	ENST00000458722	ENSP00000411274	522	P/R	cCa/cGa	-	0	1	NA	1.928	medium_impact	1.904	high_impact	NA	NA
5	Liver metastases	Private in metastatic tumor	19:1611816	TCF3	transcription factor 3 (E2A immunoglobulin enhancer binding factors E12/E47)	ENST00000395423	ENSP00000378813	623	K/E	Aaa/Gaa	-	0	0.984	NA	3.104	high_impact	1.229	medium_impact	NA	NA
5	Primary tumor	Private in primary tumor	20:61492664	TCFL5	transcription factor-like 5 (basic helix-loop-helix)	ENST00000335351	ENSP00000334294	120	C/Y	tGc/tAc	-	0	0.887	0.895	2.056	high_impact	0.714	medium_impact	-0.17	low_impact
5	Liver metastases	Private in metastatic tumor	17:56699054	TEX14	testis expressed 14	ENST00000240361	ENSP00000240361	171	R/W	Cgg/Tgg	-	0	0.982	0.975	2.032	high_impact	1.19	medium_impact	-0.073	low_impact
5	Primary tumor	Common in primary and metastatic tumors	6:155451352	TIAM2	T-cell lymphoma invasion and metastasis 2	ENST00000456144	ENSP00000407746	332	R/H	cGt/cAt	rs931312	0.1	0.999	2.19	0.212	medium_impact	1.943	high_impact	1.324	medium_impact
5	Liver metastases	Common in primary and metastatic tumors	6:155451352	TIAM2	T-cell lymphoma invasion and metastasis 2	ENST00000528928	ENSP00000431402	578	R/H	cGt/cAt	rs931312	0	0.999	NA	1.937	medium_impact	1.943	high_impact	NA	NA
5	Liver metastases	Private in metastatic tumor	3:136573306	TMEM22	transmembrane protein 22	ENST00000393079	ENSP00000376794	2	D/H	Gat/Cat	-	0	0.997	NA	2.929	high_impact	1.512	high_impact	NA	NA
5	Prima	Private in	6:32064	TNX	tenascin XB	ENST0	ENSP00	420	V/	Gtg/	-	0	0.9	NA	2.031	high_im	0.986	medium_i	NA	NA

	ry tumor	primary tumor	372	B		0000375244	000364393		M	Atg			7			pact		mpact		
5	Liver metastases	Private in metastatic tumor	2:1481231	TPO	thyroid peroxidase	ENST0000329066	ENSP0000329869	398	S/T	aGc/aCc	rs2175977	0.002	0.994	1.935	0.746	medium_impact	1.632	high_impact	1.852	medium_impact
5	Primary tumor	Private in primary tumor	1:117659352	TRIM45	tripartite motif containing 45	ENST0000369461	ENSP0000358473	356	R/Q	cGg/cAg	rs3738413	0.003	0.996	NA	0.675	medium_impact	1.6	high_impact	NA	NA
5	Liver metastases	Private in metastatic tumor	17:73874071	TRIM47	tripartite motif containing 47	ENST0000254816	ENSP0000254816	187	R/W	Cgg/Tgg	rs4600514	0	0.998	2.24	2.11	high_impact	1.876	high_impact	1.38	medium_impact
5	Liver metastases	Private in metastatic tumor	9:135796804	TSC1	tuberous sclerosis 1	ENST0000545250	ENSP0000444017	177	R/Q	cGa/cAa	-	0	0.998	NA	1.654	medium_impact	1.677	high_impact	NA	NA
5	Liver metastases	Private in metastatic tumor	20:51871153	TSHZ2	teashirt zinc finger homeobox 2	ENST0000329613	ENSP0000333114	383	G/R	Ggg/Cgg	-	0	1	NA	2.051	high_impact	1.936	high_impact	NA	NA
5	Primary tumor	Common in primary and metastatic tumors	8:98288929	TSPYL5	TSPY-like 5	ENST0000322128	ENSP0000322802	382	L/I	Ctt/Att	-	0	0.959	2.83	2.929	high_impact	0.848	medium_impact	2.111	medium_impact
5	Liver metastases	Common in primary and metastatic tumors	8:98288929	TSPYL5	TSPY-like 5	ENST0000322128	ENSP0000322802	382	L/I	Ctt/Att	-	0	0.959	2.83	2.929	high_impact	0.848	medium_impact	2.111	medium_impact
5	Liver metastases	Private in metastatic tumor	6:167754289	TTLL2	tubulin tyrosine ligase-like family, member 2	ENST0000540954	ENSP0000440649	228	S/G	Agc/Ggc	-	0.0	0.999	NA	1.068	medium_impact	1.714	high_impact	NA	NA

												1								
5	Primary tumor	Private in primary tumor	11:77920601	USP35	ubiquitin specific peptidase 35	ENST00000441408	ENSP00000400825	153	E/G	gAa/gGa	-	0	0	NA	2.021	high_impact	-1.429	low_impact	NA	NA
5	Primary tumor	Private in primary tumor	4:144116976	USP38	ubiquitin specific peptidase 38	ENST00000510377	ENSP00000427647	309	V/G	gTt/gGt	-	0	0.996	NA	2.021	high_impact	1.527	high_impact	NA	NA
5	Primary tumor	Private in primary tumor	7:12400783	VWDE	von Willebrand factor D and EGF domains	ENST00000275358	ENSP00000275358	1050	C/Y	tGt/tAt	rs35171886	0	0.996	2.34	2.075	high_impact	1.503	high_impact	1.312	medium_impact
5	Primary tumor	Private in primary tumor	9:131515573	ZER1	zer-1 homolog (C. elegans)	ENST00000291900	ENSP00000291900	206	L/V	Ctc/Gtc	-	0	0.958	1.82	2.11	high_impact	1.091	medium_impact	0.917	low_impact
5	Primary tumor	Private in primary tumor	14:23994199	ZFHX2	zinc finger homeobox 2	ENST00000419474	ENSP00000413418	1651	R/H	cGc/cAc	-	0	0	3.755	2.056	high_impact	-1.54	low_impact	2.655	medium_impact
5	Primary tumor	Private in primary tumor	10:75188033	ZMYND17	zinc finger, MYND-type containing 17	ENST00000299432	ENSP00000299432	4	R/W	Cgg/Tgg	-	0	0	1.59	2.044	high_impact	-1.412	low_impact	0.439	low_impact
5	Primary tumor	Private in primary tumor	6:27420450	ZNF184	zinc finger protein 184	ENST00000341087	ENSP00000341552	296	H/Q	caT/caG	-	0	0.88	NA	2.084	high_impact	0.756	medium_impact	NA	NA
5	Primary tumor	Common in primary and metastatic tumors	19:9271985	ZNF317	zinc finger protein 317	ENST00000360385	ENSP00000353554	523	E/G	gAg/gGg	-	0	0.804	NA	2.084	high_impact	0.609	medium_impact	NA	NA
5	Liver metastases	Common in primary and metastatic tumors	19:9271985	ZNF317	zinc finger protein 317	ENST00000360385	ENSP00000353554	523	E/G	gAg/gGg	-	0	0.804	NA	2.084	high_impact	0.609	medium_impact	NA	NA

5	Primary tumor	Common in primary and metastatic tumors	9:109688001	ZNF462	zinc finger protein 462	ENST00000457913	ENSP00000414570	603	T/N	aCc/aAc	-	0	0.822	NA	2.084	high_impact	0.639	medium_impact	NA	NA
5	Liver metastases	Common in primary and metastatic tumors	9:109688001	ZNF462	zinc finger protein 462	ENST00000457913	ENSP00000414570	603	T/N	aCc/aAc	-	0	0.822	NA	2.084	high_impact	0.639	medium_impact	NA	NA
5	Liver metastases	Private in metastatic tumor	9:109687431	ZNF462	zinc finger protein 462	ENST00000277225	ENSP00000277225	413	A/E	gCa/gAa	-	0	0	0.805	2.084	high_impact	-1.484	low_impact	-0.239	low_impact
5	Primary tumor	Private in primary tumor	19:58118381	ZNF530	zinc finger protein 530	ENST00000332854	ENSP00000332861	496	C/W	tgT/tgG	-	0	0.998	4.165	2.084	high_impact	1.818	high_impact	2.876	medium_impact
5	Liver metastases	Private in metastatic tumor	19:58370895	ZNF587	zinc finger protein 587	ENST00000423137	ENSP00000393865	371	R/L	cGt/cTt	-	0	0.987	NA	2.084	high_impact	1.344	medium_impact	NA	NA
6	Liver metastases	Private in metastatic tumor	3:151535235	AADAC	arylacetamide deacetylase (esterase)	ENST00000232892	ENSP00000232892	74	T/A	Acc/Gcc	-	0.53	0.644	2.47	-0.516	low_impact	0.184	medium_impact	4.09	high_impact
6	Primary tumor	Common in primary and metastatic tumors	7:20762646	ABCB5	ATP-binding cassette, sub-family B (MDR/TAP), member 5	ENST00000258738	ENSP00000258738	365	G/V	gGt/gTt	rs62453384	0	0.611	3.195	2.027	high_impact	0.006	medium_impact	1.262	medium_impact
6	Liver metastases	Common in primary and metastatic tumors	7:20762646	ABCB5	ATP-binding cassette, sub-family B (MDR/TAP), member 5	ENST00000258738	ENSP00000258738	365	G/V	gGt/gTt	rs62453384	0	0.611	3.195	2.027	high_impact	0.006	medium_impact	1.262	medium_impact
6	Liver	Private in	16:7735	ADA	ADAM metallopeptidase with thrombospondin	ENST0	ENSP00	626	L/I	Tta/A	rs116	0	0.0	1.015	2.174	high_im	-1.012	low_impa	-0.023	low_impact

	metastases	metastatic tumor	9919	MTS18	type 1 motif, 18	0000282849	000282849			ta	40912		05			pact		ct		
6	Primary tumor	Common in primary and metastatic tumors	14:105417766	AHNAK2	AHNAK nucleoprotein 2	ENST00000333244	ENSP00000353114	1341	S/F	tCt/tTt	rs2396457	0.003	0.996	3.275	0.69	medium_impact	1.554	high_impact	2.368	medium_impact
6	Liver metastases	Common in primary and metastatic tumors	14:105417766	AHNAK2	AHNAK nucleoprotein 2	ENST00000333244	ENSP00000353114	1341	S/F	tCt/tTt	rs2396457	0.003	0.996	3.275	0.69	medium_impact	1.554	high_impact	2.368	medium_impact
6	Liver metastases	Private in metastatic tumor	19:16308875	AP1M1	adaptor-related protein complex 1, mu 1 subunit	ENST00000429941	ENSP00000411498	13	G/A	gGc/gCc	-	0	0.962	NA	2.062	high_impact	0.995	medium_impact	NA	NA
6	Liver metastases	Private in metastatic tumor	19:1469239	APC2	adenomatosis polyposis coli 2	ENST00000233607	ENSP00000233607	1980	S/Y	tCc/tAc	-	0.003	0.995	1.845	0.657	medium_impact	1.674	high_impact	0.808	low_impact
6	Liver metastases	Private in metastatic tumor	4:77025779	ART3	ADP-ribosyltransferase 3	ENST00000511188	ENSP00000422249	86	P/S	Cct/Tct	-	0	0	NA	2.102	high_impact	-1.239	low_impact	NA	NA
6	Primary tumor	Common in primary and metastatic tumors	1:1431165	ATAD3B	ATPase family, AAA domain containing 3B	ENST00000378737	ENSP00000368011	473	P/S	Ccg/Tcg	rs9792879	0	0.794	NA	2.101	high_impact	0.52	medium_impact	NA	NA
6	Liver metastases	Common in primary and metastatic tumors	1:1431165	ATAD3B	ATPase family, AAA domain containing 3B	ENST00000308647	ENSP00000311766	639	P/S	Ccg/Tcg	rs9792879	0	0.438	0	2.101	high_impact	0.142	medium_impact	-1.067	low_impact
6	Liver metas	Private in metastatic	X:107393517	ATG4A	ATG4 autophagy related 4 homolog A (S. cerevisiae)	ENST000003 00003	ENSP000003783	290	S/L	tCa/tTa	-	0	0.005	NA	2.07	high_impact	-0.998	low_impact	NA	NA

	tases	tumor				94892	54													
6	Liver metastases	Private in metastatic tumor	7:140453136	BRAF	v-raf murine sarcoma viral oncogene homolog B1	ENST00000496384	ENSP00000419060	208	V/E	gTg/gAg	rs113488022	0	0.998	NA	1.786	medium_impact	1.951	high_impact	NA	NA
6	Liver metastases	Private in metastatic tumor	19:15353743	BRD4	bromodomain containing 4	ENST00000263377	ENSP00000263377	1046	P/H	cCc/cAc	-	0	0	1.355	2.064	high_impact	-1.491	low_impact	0.343	low_impact
6	Primary tumor	Common in primary and metastatic tumors	14:50092471	C14orf104	chromosome 14 open reading frame 104	ENST00000298292	ENSP00000298292	768	D/G	gAt/gGt	rs9989177	0	0.664	1.39	2.062	high_impact	0.372	medium_impact	0.391	low_impact
6	Liver metastases	Common in primary and metastatic tumors	14:50092471	C14orf104	chromosome 14 open reading frame 104	ENST00000406043	ENSP00000384862	720	D/G	gAt/gGt	rs9989177	0	0.438	NA	2.062	high_impact	0.144	medium_impact	NA	NA
6	Primary tumor	Private in primary tumor	19:2280885	C19orf35	chromosome 19 open reading frame 35	ENST00000342063	ENSP00000345102	16	P/S	Ccc/Tcc	rs55662626	0	0.794	1.79	2.929	high_impact	0.397	medium_impact	0.972	low_impact
6	Primary tumor	Private in primary tumor	19:6712372	C3	complement component 3	ENST00000428815	ENSP00000406291	389	V/L	Gtg/Ttg	-	0	0	NA	2.194	high_impact	-1.461	low_impact	NA	NA
6	Liver metastases	Private in metastatic tumor	6:32261329	C6orf10	chromosome 6 open reading frame 10	ENST00000442822	ENSP00000411164	365	G/D	gGt/gAt	-	0.08	0.998	NA	0.435	medium_impact	1.613	high_impact	NA	NA
6	Primary tumor	Private in primary tumor	6:71289189	C6orf57	chromosome 6 open reading frame 57	ENST00000370474	ENSP00000359505	46	Q/R	cAg/cGg	rs1048886	0	0.909	2.215	2.929	high_impact	0.636	medium_impact	1.437	medium_impact
6	Liver metas	Private in metastatic	8:86389503	CA2	carbonic anhydrase II	ENST000002	ENSP000002853	221	Q/R	cAg/cGg	-	0	1	4.69	1.922	medium_imp	1.673	high_impact	2.971	medium_impact

	tases	tumor				85379	79									act				
6	Liver metastases	Private in metastatic tumor	15:40914177	CASC5	cancer susceptibility candidate 5	ENST0000399668	ENSP0000382576	572	M/T	aTg/aCg	rs11858113	0	0	NA	2.062	high_impact	-1.483	low_impact	NA	NA
6	Liver metastases	Private in metastatic tumor	2:109408208	CCDC138	coiled-coil domain containing 138	ENST0000456512	ENSP0000392385	13	R/K	aGa/aAa	rs6740879	0	0.162	NA	2.117	high_impact	-0.194	low_impact	NA	NA
6	Primary tumor	Common in primary and metastatic tumors	1:208061142	CD34	CD34 molecule	ENST0000367037	ENSP0000356004	337	A/S	Gcc/Tcc	rs28362497	0	0.997	NA	2.992	high_impact	1.773	high_impact	NA	NA
6	Liver metastases	Common in primary and metastatic tumors	1:208061142	CD34	CD34 molecule	ENST0000537704	ENSP0000442874	232	A/S	Gcc/Tcc	rs28362497	0	0.997	NA	2.992	high_impact	1.773	high_impact	NA	NA
6	Liver metastases	Private in metastatic tumor	10:11356161	CELF2	CUGBP, Elav-like family member 2	ENST0000399850	ENSP0000382743	315	G/W	Ggg/Tgg	-	0.02	0.997	NA	0.843	medium_impact	1.634	high_impact	NA	NA
6	Primary tumor	Common in primary and metastatic tumors	4:47939595	CNGA1	cyclic nucleotide gated channel alpha 1	ENST0000402813	ENSP0000384264	375	Y/H	Tat/Cat	-	0.01	0.998	3.035	1.052	medium_impact	1.837	high_impact	1.535	medium_impact
6	Liver metastases	Common in primary and metastatic tumors	4:47939595	CNGA1	cyclic nucleotide gated channel alpha 1	ENST0000358519	ENSP0000351320	306	Y/H	Tat/Cat	-	0.03	1	3.685	0.532	medium_impact	2.027	high_impact	2.121	medium_impact
6	Liver metastases	Private in metastatic tumor	10:105824333	COL17A1	collagen, type XVII, alpha 1	ENST0000393211	ENSP0000376905	210	T/M	aCg/aTg	rs805708	0.2	0.997	NA	-0.316	low_impact	1.512	high_impact	NA	NA

												5								
6	Liver metastases	Private in metastatic tumor	4:1388459	CRIPAK	cysteine-rich PAK1 inhibitor	ENST00000324803	ENSP00000323978	54	R/C	Cgc/Tgc	-	NA	0.998	0	NA	NA	1.724	high_impact	-1.067	low_impact
6	Liver metastases	Private in metastatic tumor	8:2909992	CSMD1	CUB and Sushi multiple domains 1	ENST00000335551	ENSP00000334828	1969	T/M	aCg/aTg	rs6558702	0.14	0.998	NA	0.085	medium_impact	1.613	high_impact	NA	NA
6	Liver metastases	Private in metastatic tumor	10:17130299	CUBN	cubilin (intrinsic factor-cobalamin receptor)	ENST00000377833	ENSP00000367064	604	P/L	cCg/cTg	-	0	1	3.645	2.214	high_impact	1.866	high_impact	2.931	medium_impact
6	Liver metastases	Private in metastatic tumor	8:42231715	DKK4	dickkopf homolog 4 (Xenopus laevis)	ENST00000220812	ENSP00000220812	193	R/H	cGt/cAt	-	0	0.999	2.465	2.064	high_impact	1.91	high_impact	1.535	medium_impact
6	Primary tumor	Common in primary and metastatic tumors	X:32380996	DMD	dystrophin	ENST00000378682	ENSP00000367953	404	R/H	cGc/cAc	rs1801187	0.05	0.978	NA	0.637	medium_impact	1.757	high_impact	NA	NA
6	Liver metastases	Common in primary and metastatic tumors	X:32380996	DMD	dystrophin	ENST00000357033	ENSP00000354923	1745	R/H	cGc/cAc	rs1801187	0.03	0.997	2.25	0.875	medium_impact	2.38	high_impact	2.319	medium_impact
6	Primary tumor	Private in primary tumor	7:21751375	DNAH11	dynein, axonemal, heavy chain 11	ENST00000328843	ENSP00000330671	2301	M/L	Atg/Ctg	-	0	0.084	[sent]	2.056	high_impact	-0.424	low_impact	NA	NA
6	Primary tumor	Private in primary tumor	7:21893993	DNAH11	dynein, axonemal, heavy chain 11	ENST00000421290	ENSP00000403651	103	V/L	Gtg/Ttg	rs4722064	0.04	0.998	NA	0.531	medium_impact	1.643	high_impact	NA	NA
6	Liver	Private in	2:19663	DN	dynein, axonemal, heavy chain 7	ENST0	ENSP00	265	N/	aAc/	-	0	0.9	NA	2.055	high_im	1.543	high_impa	NA	NA

	metastases	metastatic tumor	6472	AH7		0000409063	000386912		S	aGc			97			pact		ct		
6	Liver metastases	Private in metastatic tumor	11:117308650	DSCAML1	Down syndrome cell adhesion molecule like 1	ENST0000446508	ENSP0000394795	1232	R/C	Cgc/Tgc	-	0	1	NA	2.052	high_impact	1.877	high_impact	NA	NA
6	Primary tumor	Common in primary and metastatic tumors	6:56426282	DST	dystonin	ENST0000244364	ENSP0000244364	2089	L/F	Ctc/Ttc	-	0.02	0.998	NA	0.931	medium_impact	1.77	high_impact	NA	NA
6	Liver metastases	Common in primary and metastatic tumors	6:56426282	DST	dystonin	ENST0000244364	ENSP0000244364	2089	L/F	Ctc/Ttc	-	0.02	0.998	NA	0.931	medium_impact	1.77	high_impact	NA	NA
6	Liver metastases	Private in metastatic tumor	11:58940192	DTX4	deltex homolog 4 (Drosophila)	ENST0000227451	ENSP0000227451	42	G/R	Ggc/Cgc	-	0.1	1	2.045	0.155	medium_impact	1.946	high_impact	1.015	medium_impact
6	Primary tumor	Common in primary and metastatic tumors	2:63182752	EHBP1	EH domain binding protein 1	ENST0000422032	ENSP0000388442	2	N/S	aAt/aGt	-	0	0.022	NA	2.062	high_impact	-0.723	low_impact	NA	NA
6	Liver metastases	Common in primary and metastatic tumors	2:63182752	EHBP1	EH domain binding protein 1	ENST0000422032	ENSP0000388442	2	N/S	aAt/aGt	-	0	0.022	NA	2.062	high_impact	-0.723	low_impact	NA	NA
6	Liver metastases	Private in metastatic tumor	11:65349756	EHBP1L1	EH domain binding protein 1-like 1	ENST0000309295	ENSP0000312671	538	V/G	gTg/gGg	rs6591182	0	0.975	1.1	2.064	high_impact	1.106	medium_impact	0.112	low_impact
6	Liver metastases	Private in metastatic tumor	1:79357360	ELTD1	EGF, latrophilin and seven transmembrane domain containing 1	ENST0000370742	ENSP0000359778	620	A/G	gCc/gGc	rs2275902	0.0	0.999	2.84	1.205	medium_impact	1.915	high_impact	1.985	medium_impact

												1								
6	Liver metastases	Private in metastatic tumor	11:62369955	EML3	echinoderm microtubule associated protein like 3	ENST00000439994	ENSP00000409611	136	D/Y	Gac/Tac	-	0	0	NA	2.062	high_impact	-1.483	low_impact	NA	NA
6	Liver metastases	Private in metastatic tumor	3:40456245	ENTPD3	ectonucleoside triphosphate diphosphohydrolase 3	ENST00000445129	ENSP00000404671	171	F/V	Ttt/Gtt	-	0	0.408	NA	2.044	high_impact	0.145	medium_impact	NA	NA
6	Primary tumor	Private in primary tumor	5:96219561	ERAP2	endoplasmic reticulum aminopeptidase 2	ENST00000379904	ENSP00000369235	214	P/L	cCg/cTg	rs3733905	0	1	NA	2.306	high_impact	1.991	high_impact	NA	NA
6	Primary tumor	Private in primary tumor	5:137849395	ETF1	eukaryotic translation termination factor 1	ENST00000512198	ENSP00000422272	88	G/V	gGc/gTc	-	0	0	NA	2.613	high_impact	-1.048	low_impact	NA	NA
6	Liver metastases	Private in metastatic tumor	15:76578762	ETFA	electron-transfer-flavoprotein, alpha polypeptide	ENST00000433983	ENSP00000399273	122	T/I	aCa/aTa	rs1801591	0	1	NA	1.982	medium_impact	1.795	high_impact	NA	NA
6	Primary tumor	Private in primary tumor	11:61643380	FADS3	fatty acid desaturase 3	ENST00000278829	ENSP00000278829	410	K/M	aAg/aTg	-	0	1	3.38	1.848	medium_impact	1.852	high_impact	1.572	medium_impact
6	Primary tumor	Private in primary tumor	X:37026735	FAM47C	family with sequence similarity 47, member C	ENST00000358047	ENSP00000367913	84	R/S	agA/agT	-	0.42	0.998	1.7	-0.749	low_impact	1.613	high_impact	0.874	low_impact
6	Primary tumor	Private in primary tumor	2:186678577	FSIP2	fibrous sheath interacting protein 2	ENST00000343098	ENSP00000344403	6800	K/N	aaA/aaT	-	0	0.759	NA	2.929	high_impact	0.347	medium_impact	NA	NA
6	Primary tumor	Private in primary tumor	19:1008226	GRIN3B	glutamate receptor, ionotropic, N-methyl-D-aspartate 3B	ENST00000234389	ENSP00000234389	801	D/A	gAc/gCc	-	0.01	0.998	2.485	1.063	medium_impact	1.814	high_impact	1.327	medium_impact

6	Liver metastases	Private in metastatic tumor	22:46725986	GTSE1	G-2 and S-phase expressed 1	ENST00000361934	ENSP00000354634	687	A/T	Gct/Act	-	0.117	0.995	NA	-0.09	low_impact	1.51	high_impact	NA	NA
6	Liver metastases	Private in metastatic tumor	6:32609800	HLA-DQA1	major histocompatibility complex, class II, DQ alpha 1	ENST00000486548	ENSP00000437183	101	P/H	cCc/cAc	-	0	0.998	NA	2.062	high_impact	1.724	high_impact	NA	NA
6	Liver metastases	Private in metastatic tumor	10:92508705	HTR7	5-hydroxytryptamine (serotonin) receptor 7 (adenylate cyclase-coupled)	ENST00000371721	ENSP00000360786	396	R/C	Cgc/Tgc	-	0.01	0.997	NA	1.079	medium_impact	1.506	high_impact	NA	NA
6	Primary tumor	Common in primary and metastatic tumors	11:125769354	HYLS1	hydrolethalus syndrome 1	ENST00000425380	ENSP00000414884	31	C/R	Tgt/Cgt	rs667782	0	0.594	1.61	2.929	high_impact	0.155	medium_impact	0.775	low_impact
6	Liver metastases	Common in primary and metastatic tumors	11:125769354	HYLS1	hydrolethalus syndrome 1	ENST00000356438	ENSP00000348815	31	C/R	Tgt/Cgt	rs667782	0	0.594	1.61	2.929	high_impact	0.155	medium_impact	0.775	low_impact
6	Liver metastases	Private in metastatic tumor	19:39734325	IL28B	interleukin 28B (interferon, lambda 3)	ENST00000413851	ENSP00000409000	180	R/C	Cgc/Tgc	rs77379751	0	1	2.785	2.087	high_impact	1.83	high_impact	1.612	medium_impact
6	Primary tumor	Private in primary tumor	5:61923141	IPO11	importin 11	ENST00000409296	ENSP00000386992	1015	F/S	tTc/tCc	-	0	0.759	NA	2.035	high_impact	0.493	medium_impact	NA	NA
6	Liver metastases	Private in metastatic tumor	15:74468406	ISLR	immunoglobulin superfamily containing leucine-rich repeat	ENST00000395118	ENSP00000378550	403	G/R	Ggg/Agg	-	0	0.94	0.695	2.062	high_impact	0.878	medium_impact	-0.338	low_impact
6	Primary	Private in primary tumor	14:75936027	JDP2	Jun dimerization protein 2	ENST000002	ENSP000002675	125	L/R	cTg/cGg	-	0	0.998	NA	2.077	high_impact	1.857	high_impact	NA	NA

	tumor					67569	69													
6	Liver metastases	Private in metastatic tumor	7:150648020	KCNH2	potassium voltage-gated channel, subfamily H (eag-related), member 2	ENST00000430723	ENSP00000387657	712	D/N	Gac/Aac	-	0	0.997	NA	2.309	high_impact	2.069	high_impact	NA	NA
6	Primary tumor	Private in primary tumor	6:43039077	KLC4	kinesin light chain 4	ENST00000259708	ENSP00000259708	425	T/N	aCc/aAc	-	0.004	0.998	NA	0.531	medium_impact	1.643	high_impact	NA	NA
6	Liver metastases	Private in metastatic tumor	12:53242440	KRT78	keratin 78	ENST00000304620	ENSP00000306261	92	L/P	cTg/cCg	rs2013335	0	0.996	3.45	2.018	high_impact	1.348	medium_impact	2.093	medium_impact
6	Liver metastases	Private in metastatic tumor	21:45999653	KRTAP10-5	keratin associated protein 10-5	ENST00000400372	ENSP00000383223	268	R/P	cGc/cCc	rs464391	0	0.998	2.6	2.929	high_impact	1.613	high_impact	1.859	medium_impact
6	Primary tumor	Common in primary and metastatic tumors	6:112430669	LAMA4	laminin, alpha 4	ENST00000230538	ENSP00000230538	1815	V/I	Gta/Ata	rs3734292	0	0.965	1.94	2.031	high_impact	0.948	medium_impact	0.93	low_impact
6	Liver metastases	Common in primary and metastatic tumors	6:112430669	LAMA4	laminin, alpha 4	ENST00000389463	ENSP00000374114	1808	V/I	Gta/Ata	rs3734292	0	0.984	NA	2.031	high_impact	1.139	medium_impact	NA	NA
6	Liver metastases	Private in metastatic tumor	9:124975995	LHX6	LIM homeobox 6	ENST00000373755	ENSP00000362860	286	S/W	tCg/tGg	-	0	0.97	0.695	2.056	high_impact	1.07	medium_impact	-0.367	low_impact
6	Liver metastases	Private in metastatic tumor	19:2290325	LINGO3	leucine rich repeat and Ig domain containing 3	ENST00000404279	ENSP00000384979	484	N/I	aAc/aTc	-	0	1	4.505	2.062	high_impact	1.894	high_impact	3.659	high_impact
6	Liver metas	Private in metastatic	7:100183548	LRCH4	leucine-rich repeats and calponin homology (CH) domain containing 4	ENST000003	ENSP000003096	59	R/P	cGg/cCg	-	0	0.999	1.965	2.089	high_impact	1.974	high_impact	1.225	medium_impact

	tases	tumor				10300	89													
6	Liver metastases	Private in metastatic tumor	3:169514585	LRRC34	leucine rich repeat containing 34	ENST00000528597	ENSP00000436883	35	L/I	Tta/Ata	rs10936600	0	0.958	NA	2.064	high_impact	0.973	medium_impact	NA	NA
6	Primary tumor	Common in primary and metastatic tumors	6:90408742	MDN1	MDN1, midasin homolog (yeast)	ENST00000428876	ENSP00000413970	3004	E/K	Gaa/Aaa	rs12530146	0	0.961	0.805	2.124	high_impact	0.979	medium_impact	-0.377	low_impact
6	Liver metastases	Common in primary and metastatic tumors	6:90408742	MDN1	MDN1, midasin homolog (yeast)	ENST00000369393	ENSP00000358400	3004	E/K	Gaa/Aaa	rs12530146	0	0.961	0.805	2.124	high_impact	0.979	medium_impact	-0.377	low_impact
6	Primary tumor	Common in primary and metastatic tumors	1:3416449	MEGF6	multiple EGF-like-domains 6	ENST00000485002	ENSP00000419033	756	R/L	cGg/cTg	rs7553399	0.3	0.998	NA	-0.337	low_impact	1.671	high_impact	NA	NA
6	Liver metastases	Common in primary and metastatic tumors	1:3416449	MEGF6	multiple EGF-like-domains 6	ENST00000485002	ENSP00000419033	756	R/L	cGg/cTg	rs7553399	0.3	0.998	NA	-0.337	low_impact	1.671	high_impact	NA	NA
6	Primary tumor	Private in primary tumor	22:37873005	MFNG	MFNG O-fucosylpeptide 3-beta-N-acetylglucosaminyltransferase	ENST00000416983	ENSP00000413855	178	F/L	ttC/ttG	-	0	0.94	NA	2.051	high_impact	0.904	medium_impact	NA	NA
6	Primary tumor	Common in primary and metastatic tumors	6:31477681	MICB	MHC class I polypeptide-related sequence B	ENST00000252229	ENSP00000252229	383	T/A	Acc/Gcc	rs1065076	0	0	NA	2.062	high_impact	-1.483	low_impact	NA	NA
6	Liver metastases	Common in primary and metastatic	6:31477681	MICB	MHC class I polypeptide-related sequence B	ENST00000399150	ENSP00000382103	340	T/A	Acc/Gcc	rs1065076	0	0	NA	2.062	high_impact	-1.483	low_impact	NA	NA

		tumors																		
6	Primary tumor	Common in primary and metastatic tumors	8:2054058	MYOM2	myomesin (M-protein) 2, 165kDa	ENST0000523438	ENSP00000428396	346	D/N	Gat/Aat	-	0.009	0.999	NA	0.132	medium_impact	1.545	high_impact	NA	NA
6	Liver metastases	Common in primary and metastatic tumors	8:2054058	MYOM2	myomesin (M-protein) 2, 165kDa	ENST0000523438	ENSP00000428396	346	D/N	Gat/Aat	-	0.009	0.999	NA	0.132	medium_impact	1.545	high_impact	NA	NA
6	Primary tumor	Common in primary and metastatic tumors	3:40231834	MYRIP	myosin VIIA and Rab interacting protein	ENST0000458292	ENSP00000413392	515	E/D	gaG/gaC	-	0	0.816	NA	2.197	high_impact	0.479	medium_impact	NA	NA
6	Liver metastases	Common in primary and metastatic tumors	3:40231834	MYRIP	myosin VIIA and Rab interacting protein	ENST0000458292	ENSP00000413392	515	E/D	gaG/gaC	-	0	0.816	NA	2.197	high_impact	0.479	medium_impact	NA	NA
6	Primary tumor	Common in primary and metastatic tumors	13:24895437	NA	NA	ENST0000422229	ENSP00000396192	44	R/W	Cgg/Tgg	rs3751356	0.002	0.998	NA	1.244	medium_impact	1.613	high_impact	NA	NA
6	Liver metastases	Common in primary and metastatic tumors	13:24895437	NA	NA	ENST0000422229	ENSP00000396192	44	R/W	Cgg/Tgg	rs3751356	0.002	0.998	NA	1.244	medium_impact	1.613	high_impact	NA	NA
6	Primary tumor	Private in primary tumor	16:19548116	NA	NA	ENST0000396208	ENSP00000379511	375	M/I	atG/atA	rs7190666	0	0.984	1.845	2.062	high_impact	1.212	medium_impact	0.868	low_impact
6	Liver metas	Private in metastatic	3:175345143	NAALAD	N-acetylated alpha-linked acidic dipeptidase-like 2	ENST000004	ENSP000004047	622	P/R	cCc/cGc	rs9866564	0	0.999	1.04	2.12	high_impact	1.868	high_impact	-0.028	low_impact

	tases	tumor		L2		54872	05													
6	Liver metas tases	Private in metastatic tumor	2:15247 6028	NEB	nebulin	ENST0 00004 27231	ENSP00 0004165 78	3603	W/ C	tgG/t gC	rs101 72023	0	0.9 93	NA	2.154	high_im pact	1.055	medium_i mpact	NA	NA
6	Prima ry tumor	Common in primary and metastatic tumors	10:2115 7621	NEB L	nebulette	ENST0 00003 77119	ENSP00 0003663 23	219	A/ D	gCc/ gAc	rs229 6610	0	0.9 96	NA	2.154	high_im pact	1.196	medium_i mpact	NA	NA
6	Liver metas tases	Common in primary and metastatic tumors	10:2115 7621	NEB L	nebulette	ENST0 00003 77119	ENSP00 0003663 23	219	A/ D	gCc/ gAc	rs229 6610	0	0.9 96	NA	2.154	high_im pact	1.196	medium_i mpact	NA	NA
6	Prima ry tumor	Common in primary and metastatic tumors	22:2988 5473	NEF H	neurofilament, heavy polypeptide	ENST0 00003 10624	ENSP00 0003119 97	615	P/ L	cCg/ cTg	rs576 3269	0	0.9 46	2.215	2.064	high_im pact	0.907	medium_i mpact	1.275	medium_imp act
6	Liver metas tases	Common in primary and metastatic tumors	22:2988 5473	NEF H	neurofilament, heavy polypeptide	ENST0 00003 10624	ENSP00 0003119 97	615	P/ L	cCg/ cTg	rs576 3269	0	0.9 46	2.215	2.064	high_im pact	0.907	medium_i mpact	1.275	medium_imp act
6	Prima ry tumor	Common in primary and metastatic tumors	1:78392 446	NEX N	nexilin (F actin binding protein)	ENST0 00003 30010	ENSP00 0003273 63	181	G/ R	Gga/ Aga	rs116 6698	0 . 1	1	NA	0.096	mediu m_imp act	1.833	high_impa ct	NA	NA
6	Liver metas tases	Common in primary and metastatic tumors	1:78392 446	NEX N	nexilin (F actin binding protein)	ENST0 00003 34785	ENSP00 0003339 38	245	G/ R	Gga/ Aga	rs116 6698	0 . 0 4	1	2.63	0.501	mediu m_imp act	1.833	high_impa ct	1.747	medium_imp act
6	Liver metas	Private in metastatic	1:41236 345	NFY C	nuclear transcription factor Y, gamma	ENST0 00003	ENSP00 0003126	408	G/ R	Ggg/ Cgg	-	0	0.8 96	[sent]	2.008	high_im pact	0.788	medium_i mpact	NA	NA

	tases	tumor				08733	17													
6	Primary tumor	Common in primary and metastatic tumors	7:144095600	NOBOX	NOBOX oogenesis homeobox	ENST00000483238	ENSP00000419565	485	F/L	Ttc/Ctc	rs2699503	0	0.632	NA	2.051	high_impact	0.342	medium_impact	NA	NA
6	Liver metastases	Common in primary and metastatic tumors	7:144095600	NOBOX	NOBOX oogenesis homeobox	ENST00000223140	ENSP00000223140	400	F/L	Ttc/Ctc	rs2699503	0	0.607	NA	2.051	high_impact	0.316	medium_impact	NA	NA
6	Liver metastases	Private in metastatic tumor	12:133633170	NOC4L	nucleolar complex associated 4 homolog (S. cerevisiae)	ENST00000330579	ENSP00000328854	252	F/S	tTc/tCc	-	0	0.999	3.065	2.062	high_impact	1.894	high_impact	2.148	medium_impact
6	Liver metastases	Private in metastatic tumor	1:6614535	NOL9	nucleolar protein 9	ENST00000377705	ENSP00000366934	10	R/W	Cgg/Tgg	rs4908923	0	0.99	0.805	2.009	high_impact	1.463	medium_impact	-0.243	low_impact
6	Liver metastases	Private in metastatic tumor	19:41192855	NUMBL	numb homolog (Drosophila)-like	ENST00000252891	ENSP00000252891	24	G/R	Ggg/Cgg	-	0	0	0	2.062	high_impact	-1.483	low_impact	-1.067	low_impact
6	Primary tumor	Private in primary tumor	19:464217	ODF3L2	outer dense fiber of sperm tails 3-like 2	ENST00000315489	ENSP00000318029	166	M/T	aTg/aCg	-	0	0.021	1.04	2.929	high_impact	-0.901	low_impact	0.151	low_impact
6	Liver metastases	Private in metastatic tumor	14:20295779	OR4N2	olfactory receptor, family 4, subfamily N, member 2	ENST00000315947	ENSP00000319601	58	P/A	Ccc/Gcc	rs11621884	0.01	0.999	3.43	0.874	medium_impact	1.724	high_impact	1.253	medium_impact
6	Primary tumor	Private in primary tumor	11:55563051	OR5D14	olfactory receptor, family 5, subfamily D, member 14	ENST00000335605	ENSP00000334456	7	N/T	aAt/aCt	-	0	0.998	3.1	1.67	medium_impact	1.564	high_impact	1.035	medium_impact
6	Primary	Common in primary and	10:56423968	PCDH1	protocadherin-related 15	ENST000003	ENSP000003630	19	S/A	Tct/Gct	rs11004439	0	0.084	NA	2.075	high_impact	-0.402	low_impact	NA	NA

	tumor	metastatic tumors		5		73956	67													
6	Liver metastases	Common in primary and metastatic tumors	10:56423968	PCDH15	protocadherin-related 15	ENST0000373956	ENSP0000363067	19	S/A	Tct/Gct	rs11004439	0	0.084	NA	2.075	high_impact	-0.402	low_impact	NA	NA
6	Liver metastases	Private in metastatic tumor	5:140581220	PCDHB11	protocadherin beta 11	ENST0000536699	ENSP0000440344	260	R/S	Cgc/Agc	rs61742028	0	0.995	NA	2.075	high_impact	1.449	medium_impact	NA	NA
6	Liver metastases	Private in metastatic tumor	5:149513452	PDGFRB	platelet-derived growth factor receptor, beta polypeptide	ENST0000261799	ENSP0000261799	251	R/C	Cgc/Tgc	-	0.06	1	2.19	0.486	medium_impact	1.669	high_impact	1.247	medium_impact
6	Liver metastases	Private in metastatic tumor	20:1961128	PDYN	prodynorphin	ENST0000540134	ENSP0000442259	202	D/E	gaC/gaG	-	1	0.993	NA	-2.947	low_impact	1.503	high_impact	NA	NA
6	Liver metastases	Private in metastatic tumor	3:178928035	PIK3CA	phosphoinositide-3-kinase, catalytic, alpha polypeptide	ENST0000263967	ENSP0000263967	438	S/Y	tCt/tAt	-	0.01	0.371	2.08	1.097	medium_impact	-0.344	low_impact	3.754	high_impact
6	Primary tumor	Private in primary tumor	22:38539220	PLA2G6	phospholipase A2, group VI (cytosolic, calcium-independent)	ENST0000427114	ENSP0000407743	35	S/C	tCc/tGc	-	0	0	NA	2.095	high_impact	-1.439	low_impact	NA	NA
6	Liver metastases	Private in metastatic tumor	3:146167089	PLSCR2	phospholipid scramblase 2	ENST0000535500	ENSP0000437661	275	R/S	agA/agT	rs118036548	0	0.932	NA	2.441	high_impact	1.003	medium_impact	NA	NA
6	Primary tumor	Private in primary tumor	10:47087609	PPYR1	pancreatic polypeptide receptor 1	ENST0000395716	ENSP0000379066	276	V/M	Gtg/Atg	rs79871698	0.01	0.992	3.135	1.06	medium_impact	1.293	medium_impact	3.091	high_impact

6	Liver metastases	Private in metastatic tumor	7:127991153	PRRT4	proline-rich transmembrane protein 4	ENST00000535159	ENSP00000445239	819	C/W	tgT/tgG	-	0	0.927	NA	2.929	high_impact	0.696	medium_impact	NA	NA
6	Primary tumor	Private in primary tumor	17:5286462	RABEP1	rabaptin, RAB GTPase binding effector protein 1	ENST00000537505	ENSP00000445408	802	R/W	Cgg/Tgg	-	0	1	NA	2.1	high_impact	1.867	high_impact	NA	NA
6	Liver metastases	Private in metastatic tumor	12:113565946	RASAL1	RAS protein activator like 1 (GAP1 like)	ENST00000261729	ENSP00000261729	54	G/R	Ggg/Cgg	-	0	0.999	2.085	1.926	medium_impact	1.731	high_impact	1.211	medium_impact
6	Liver metastases	Private in metastatic tumor	8:55539057	RP1	retinitis pigmentosa 1 (autosomal dominant)	ENST00000220676	ENSP00000220676	872	R/H	cGt/cAt	rs444772	0	0.001	0	2.1	high_impact	-1.444	low_impact	-1.144	low_impact
6	Liver metastases	Private in metastatic tumor	1:237947731	RYR2	ryanodine receptor 2 (cardiac)	ENST00000542537	ENSP00000443798	4224	T/M	aCg/aTg	-	NA	0.993	NA	NA	NA	1.599	high_impact	NA	NA
6	Liver metastases	Private in metastatic tumor	5:76171281	S100Z	S100 calcium binding protein Z	ENST00000317593	ENSP00000320430	33	E/K	Gaa/Aaa	-	0	0.997	[sent]	2.075	high_impact	1.573	high_impact	NA	NA
6	Liver metastases	Private in metastatic tumor	3:38607917	SCN5A	sodium channel, voltage-gated, type V, alpha subunit	ENST00000449557	ENSP00000413996	1221	D/N	Gac/Aac	CM030274	0	0.997	NA	2.121	high_impact	1.603	high_impact	NA	NA
6	Liver metastases	Private in metastatic tumor	16:29908190	SEZ6L2	seizure related 6 homolog (mouse)-like 2	ENST00000350527	ENSP00000310206	85	T/S	aCc/aGc	-	0	0	NA	2.929	high_impact	-1.667	low_impact	NA	NA
6	Primary tumor	Private in primary tumor	3:157823711	SHOX2	short stature homeobox 2	ENST00000483851	ENSP00000419362	35	R/S	Cgc/Agc	-	0	0.972	NA	2.056	high_impact	1.088	medium_impact	NA	NA
6	Liver metastases	Private in metastatic tumor	14:72055479	SIPA1L1	signal-induced proliferation-associated 1 like 1	ENST00000381232	ENSP00000370630	297	R/H	cGt/cAt	-	0.0	0.999	NA	1.174	medium_impact	1.963	high_impact	NA	NA

												1									
6	Liver metastases	Private in metastatic tumor	X:153716859	SLC10A3	solute carrier family 10 (sodium/bile acid cotransporter family), member 3	ENST00000393586	ENSP00000377211	196	G/R	Ggg/Agg	-	0	1	NA	2.036	high_impact	1.593	high_impact	NA	NA
6	Primary tumor	Private in primary tumor	9:130160341	SLC2A8	solute carrier family 2 (facilitated glucose transporter), member 8	ENST00000373360	ENSP00000362458	126	G/A	gGc/gCc	-	0	0.971	NA	2.047	high_impact	1.458	medium_impact	NA	NA
6	Liver metastases	Private in metastatic tumor	9:15423064	SNAPC3	small nuclear RNA activating complex, polypeptide 3, 50kDa	ENST00000421710	ENSP00000391832	63	L/M	Ctg/Atg	-	0.004	0.996	NA	0.574	medium_impact	1.653	high_impact	NA	NA
6	Liver metastases	Private in metastatic tumor	20:1277022	SNPH	syntaphilin	ENST00000381873	ENSP00000371297	3	M/V	Atg/Gtg	-	0	0.025	1.245	2.496	high_impact	-0.513	low_impact	0.065	low_impact
6	Liver metastases	Private in metastatic tumor	16:1034740	SOX8	SRY (sex determining region Y)-box 8	ENST00000293894	ENSP00000293894	232	K/T	aAg/aCg	-	0	0.967	3.35	2.008	high_impact	1.1	medium_impact	2.619	medium_impact
6	Primary tumor	Common in primary and metastatic tumors	1:118565953	SPAG17	sperm associated antigen 17	ENST00000336338	ENSP00000337804	1348	P/L	cCa/cTa	rs10923472	0	0.946	2.045	2.929	high_impact	0.776	medium_impact	1.251	medium_impact
6	Liver metastases	Common in primary and metastatic tumors	1:118565953	SPAG17	sperm associated antigen 17	ENST00000336338	ENSP00000337804	1348	P/L	cCa/cTa	rs10923472	0	0.946	2.045	2.929	high_impact	0.776	medium_impact	1.251	medium_impact
6	Primary tumor	Private in primary tumor	19:56029820	SSC5D	scavenger receptor cysteine rich domain containing (5 domains)	ENST00000389623	ENSP00000374274	1393	P/S	Ccc/Tcc	-	0	0	0.205	2.091	high_impact	-1.824	low_impact	-0.7	low_impact
6	Liver metas	Private in metastatic	20:60551315	TAF4	TAF4 RNA polymerase II, TATA box binding protein (TBP)-associated factor, 135kDa	ENST000004	ENSP000003990	920	T/M	aCg/aTg	-	0.	1	NA	1.262	medium_imp	1.81	high_impact	NA	NA

	tases	tumor				36129	91					0 1				act				
6	Primary tumor	Private in primary tumor	6:33281590	TAPBP	TAP binding protein (tapasin)	ENST00000489157	ENSP00000419659	30	V/A	gTg/gCg	-	0	0.025	NA	2.062	high_impact	-0.69	low_impact	NA	NA
6	Liver metastases	Private in metastatic tumor	3:30732969	TGFBR2	transforming growth factor, beta receptor II (70/80kDa)	ENST00000439925	ENSP00000392572	358	R/G	Cgt/Ggt	rs104893810	0	1	NA	2.316	high_impact	1.566	high_impact	NA	NA
6	Liver metastases	Private in metastatic tumor	3:52256029	TLR9	toll-like receptor 9	ENST00000360658	ENSP00000353874	768	A/E	gCg/gAg	-	0	0.93	2.32	2.759	high_impact	0.933	medium_impact	1.091	medium_impact
6	Liver metastases	Private in metastatic tumor	3:133098945	TMEM108	transmembrane protein 108	ENST00000510183	ENSP00000421486	130	E/D	gaG/gaC	-	0	0.907	NA	2.064	high_impact	0.762	medium_impact	NA	NA
6	Primary tumor	Common in primary and metastatic tumors	19:18729234	TMEM59L	transmembrane protein 59-like	ENST00000262817	ENSP00000262817	278	V/M	Gtg/Atg	-	0	0.953	1.665	2.929	high_impact	0.813	medium_impact	0.835	low_impact
6	Liver metastases	Common in primary and metastatic tumors	19:18729234	TMEM59L	transmembrane protein 59-like	ENST00000262817	ENSP00000262817	278	V/M	Gtg/Atg	-	0	0.953	1.665	2.929	high_impact	0.813	medium_impact	0.835	low_impact
6	Liver metastases	Private in metastatic tumor	6:32064323	TNXB	tenascin XB	ENST00000375244	ENSP00000364393	436	P/R	cCa/cGa	-	0	0.975	NA	2.031	high_impact	1.03	medium_impact	NA	NA
6	Liver metastases	Private in metastatic tumor	15:63362098	TPM1	tropomyosin 1 (alpha)	ENST00000334895	ENSP00000334624	232	R/C	Cgc/Tgc	-	0	0.072	NA	2.111	high_impact	-0.463	low_impact	NA	NA
6	Primary	Private in primary tumor	9:77376647	TRPM6	transient receptor potential cation channel, subfamily M, member 6	ENST000003	ENSP000003660	1584	K/E	Aag/Gag	rs2274924	0	0.07	NA	2.145	high_impact	-0.478	low_impact	NA	NA

	tumor					76864	60													
6	Liver metastases	Private in metastatic tumor	11:75526481	UVRAG	UV radiation resistance associated gene	ENST0000356136	ENSP0000348455	10	P/H	cCc/cAc	rs7118567	0	0	0.345	2.062	high_impact	-1.483	low_impact	-0.705	low_impact
6	Primary tumor	Common in primary and metastatic tumors	19:38377773	WDR87	WD repeat domain 87	ENST0000447313	ENSP0000405012	2180	R/W	Cgg/Tgg	rs34785154	NA	0.995	NA	NA	NA	1.5	high_impact	NA	NA
6	Liver metastases	Common in primary and metastatic tumors	19:38377773	WDR87	WD repeat domain 87	ENST0000303868	ENSP0000368025	2141	R/W	Cgg/Tgg	rs34785154	NA	0.995	0.345	NA	NA	1.5	high_impact	-0.705	low_impact
6	Primary tumor	Private in primary tumor	2:135738850	YSK4	YSK4 Sps1/Ste20-related kinase homolog (S. cerevisiae)	ENST0000375845	ENSP0000365005	1154	R/H	cGt/cAt	rs56349597	0.01	0.999	1.815	1.111	medium_impact	1.894	high_impact	0.791	low_impact
6	Primary tumor	Private in primary tumor	13:20425539	ZMYM5	zinc finger, MYM-type 5	ENST0000502168	ENSP0000445779	171	G/E	gGa/gAa	rs116615412	0	0.96	NA	2.069	high_impact	1.018	medium_impact	NA	NA
6	Liver metastases	Private in metastatic tumor	19:14829319	ZNF333	zinc finger protein 333	ENST0000292530	ENSP0000292530	394	F/I	Ttt/Att	-	0	0.978	2.22	2.084	high_impact	1.209	medium_impact	1.073	medium_impact
6	Primary tumor	Private in primary tumor	19:21606429	ZNF493	zinc finger protein 493	ENST0000355504	ENSP0000347691	195	C/F	tGt/tTt	rs4621113	0	0.985	3.495	2.084	high_impact	1.307	medium_impact	2.255	medium_impact
6	Primary tumor	Common in primary and metastatic tumors	19:38055612	ZNF571	zinc finger protein 571	ENST0000451802	ENSP0000392638	573	L/H	cTt/cAt	rs4802029	0	1	4.02	2.056	high_impact	1.933	high_impact	2.917	medium_impact
6	Liver	Common in	19:3805	ZNF	zinc finger protein 571	ENST0	ENSP00	573	L/	cTt/c	rs480	0	1	4.02	2.056	high_im	1.933	high_impa	2.917	medium_imp

	metastases	primary and metastatic tumors	5612	571		0000328550	000333660		H	At	2029					pact		ct		act
6	Primary tumor	Common in primary and metastatic tumors	19:23928102	ZNF681	zinc finger protein 681	ENST00000528059	ENSP00000433806	15	D/H	Gac/Cac	rs7248674	0.03	0.994	NA	0.719	medium_impact	1.54	high_impact	NA	NA
6	Liver metastases	Common in primary and metastatic tumors	19:23928102	ZNF681	zinc finger protein 681	ENST00000402377	ENSP00000384000	84	D/H	Gac/Cac	rs7248674	0.02	0.994	2.08	0.884	medium_impact	1.54	high_impact	0.943	low_impact
7	Liver metastases	Private in metastatic tumor	2:215818761	ABCA12	ATP-binding cassette, sub-family A (ABC1), member 12	ENST00000389661	ENSP00000374312	1837	G/D	gGt/gAt	-	0	0.999	NA	2.101	high_impact	1.834	high_impact	NA	NA
7	Primary tumor	Private in primary tumor	1:6445639	ACOT7	acyl-CoA thioesterase 7	ENST00000377855	ENSP00000367086	30	G/W	Ggg/Tgg	-	0	0.717	1.39	3.041	high_impact	1.335	medium_impact	0.619	low_impact
7	Liver metastases	Private in metastatic tumor	19:39219738	ACTN4	actinin, alpha 4	ENST00000424234	ENSP00000411187	451	D/N	Gac/Aac	-	0.56	0.983	NA	-0.62	low_impact	1.577	high_impact	NA	NA
7	Liver metastases	Private in metastatic tumor	15:100636566	ADAMTS17	ADAM metallopeptidase with thrombospondin type 1 motif, 17	ENST00000378898	ENSP00000368176	468	G/E	gGg/gAg	-	0	0.998	NA	2.174	high_impact	1.687	high_impact	NA	NA
7	Liver metastases	Private in metastatic tumor	12:43828141	ADAMTS20	ADAM metallopeptidase with thrombospondin type 1 motif, 20	ENST00000395541	ENSP00000378911	30	K/M	aAg/aTg	rs7302446	0.02	0.996	NA	1.117	medium_impact	1.767	high_impact	NA	NA
7	Liver metas	Private in metastatic	21:46603386	ADARB1	adenosine deaminase, RNA-specific, B1	ENST000000003	ENSP000003539	453	D/N	Gat/Aat	-	0	1	2.885	1.872	medium_imp	1.508	high_impact	1.301	medium_impact

	tases	tumor				60697	20									act				
7	Liver metastases	Private in metastatic tumor	1:167849829	ADCY10	adenylate cyclase 10 (soluble)	ENST00000545172	ENSP00000441992	194	V/I	Gtc/Atc	-	0.38	0.994	NA	-0.226	low_impact	1.648	high_impact	NA	NA
7	Primary tumor	Private in primary tumor	7:150817078	AGAP3	ArfGAP with GTPase domain, ankyrin repeat and PH domain 3	ENST00000467724	ENSP00000419801	98	T/M	aCg/aTg	-	0.14	0.999	NA	-0.103	low_impact	1.929	high_impact	NA	NA
7	Liver metastases	Private in metastatic tumor	10:37505192	ANKRD30A	ankyrin repeat domain 30A	ENST00000361713	ENSP00000354432	929	R/C	Cgt/Tgt	rs1200875	0	0.901	-0.345	2.066	high_impact	0.785	medium_impact	-1.444	low_impact
7	Primary tumor	Private in primary tumor	8:131073043	ASAP1	ArfGAP with SH3 domain, ankyrin repeat and PH domain 1	ENST00000524124	ENSP00000429391	813	K/Q	Aaa/Caa	-	0.22	0.998	NA	-0.157	low_impact	1.669	high_impact	NA	NA
7	Primary tumor	Common in primary and metastatic tumors	1:176918403	ASTN1	astrotactin 1	ENST00000541808	ENSP00000443205	658	G/S	Ggc/Agc	-	0	0.995	NA	2.062	high_impact	1.5	high_impact	NA	NA
7	Liver metastases	Common in primary and metastatic tumors	1:176918403	ASTN1	astrotactin 1	ENST00000424564	ENSP00000395041	658	G/S	Ggc/Agc	-	0	0.995	NA	2.062	high_impact	1.5	high_impact	NA	NA
7	Liver metastases	Private in metastatic tumor	18:77013534	ATP9B	ATPase, class II, type 9B	ENST00000307671	ENSP00000304500	421	P/S	Ccc/Tcc	-	0	1	4.095	1.961	medium_impact	1.538	high_impact	2.944	medium_impact
7	Primary tumor	Private in primary tumor	5:70806110	BDP1	B double prime 1, subunit of RNA polymerase III transcription initiation factor IIIB	ENST00000358731	ENSP00000351575	1064	L/S	tTg/tCg	-	0	0.889	0.895	2.064	high_impact	0.783	medium_impact	-0.115	low_impact

7	Primary tumor	Private in primary tumor	7:140453136	BRAF	v-raf murine sarcoma viral oncogene homolog B1	ENST00000496384	ENSP00000419060	208	V/E	gTg/gAg	rs113488022	0	0.998	NA	1.786	medium_impact	1.951	high_impact	NA	NA
7	Liver metastases	Private in metastatic tumor	16:50388339	BRD7	bromodomain containing 7	ENST00000394688	ENSP00000378180	148	Q/R	cAg/cGg	-	0.01	0.997	1.755	1.301	medium_impact	1.566	high_impact	0.784	low_impact
7	Primary tumor	Private in primary tumor	10:45473043	C10orf10	chromosome 10 open reading frame 10	ENST00000298295	ENSP00000298295	146	G/R	Ggg/Cgg	-	0	0.979	0.55	2.929	high_impact	1.021	medium_impact	-0.385	low_impact
7	Primary tumor	Private in primary tumor	10:45473043	C10orf10	chromosome 10 open reading frame 10	ENST00000432283	ENSP00000392803	98	W/C	tgG/tgC	-	0	0	NA	2.929	high_impact	-1.667	low_impact	NA	NA
7	Primary tumor	Common in primary and metastatic tumors	20:31889141	C20orf114	chromosome 20 open reading frame 114	ENST00000375378	ENSP00000364527	115	V/M	Gtg/Atg	rs6141383	0	0.711	NA	2.143	high_impact	0.436	medium_impact	NA	NA
7	Liver metastases	Common in primary and metastatic tumors	20:31889141	C20orf114	chromosome 20 open reading frame 114	ENST00000375378	ENSP00000364527	115	V/M	Gtg/Atg	rs6141383	0	0.711	NA	2.143	high_impact	0.436	medium_impact	NA	NA
7	Primary tumor	Private in primary tumor	20:62715548	C20orf201	chromosome 20 open reading frame 201	ENST00000308906	ENSP00000310801	9	G/W	Ggg/Tgg	rs4431000	0	0.985	NA	2.929	high_impact	1.106	medium_impact	NA	NA
7	Liver metastases	Private in metastatic tumor	6:151779494	C6orf211	chromosome 6 open reading frame 211	ENST00000367294	ENSP00000356263	60	S/F	tCt/tTt	-	0.01	0.997	2.46	1.137	medium_impact	1.625	high_impact	1.513	medium_impact
7	Primary	Private in primary tumor	9:35674347	CA9	carbonic anhydrase IX	ENST00005	ENSP0004385	131	R/W	Agg/Tgg	rs77984049	0	0.998	NA	1.922	medium_imp	1.524	high_impact	NA	NA

	tumor					44074	41									act				
7	Liver metastases	Private in metastatic tumor	10:70507311	CCAR1	cell division cycle and apoptosis regulator 1	ENST00000540807	ENSP00000445607	77	P/S	Cct/Tct	-	0	0	NA	2.084	high_impact	-1.484	low_impact	NA	NA
7	Liver metastases	Private in metastatic tumor	11:64120581	CCDC88B	coiled-coil domain containing 88B	ENST00000359902	ENSP00000352974	338	R/C	Cgc/Tgc	-	0.11	0.999	NA	0.238	medium_impact	1.787	high_impact	NA	NA
7	Liver metastases	Private in metastatic tumor	1:27709140	CD164L2	CD164 sialomucin-like 2	ENST00000374025	ENSP00000363137	36	F/V	Ttt/Gtt	-	0	0.636	NA	2.929	high_impact	0.2	medium_impact	NA	NA
7	Liver metastases	Private in metastatic tumor	1:111862963	CHIA	chitinase, acidic	ENST00000489524	ENSP00000433309	275	G/R	Ggc/Cgc	-	0	1	NA	1.856	medium_impact	2.246	high_impact	NA	NA
7	Liver metastases	Private in metastatic tumor	16:75563330	CHST5	carbohydrate (N-acetylglucosamine 6-O) sulfotransferase 5	ENST00000336257	ENSP00000338783	318	T/M	aCg/aTg	rs3826107	0.03	0.997	2.61	0.651	medium_impact	1.709	high_impact	1.517	medium_impact
7	Primary tumor	Common in primary and metastatic tumors	12:10167986	CLEC12B	C-type lectin domain family 12, member B	ENST00000338896	ENSP00000344563	182	I/T	aTa/aCa	-	0	0.982	2.86	2.056	high_impact	1.182	medium_impact	1.95	medium_impact
7	Liver metastases	Common in primary and metastatic tumors	12:10167986	CLEC12B	C-type lectin domain family 12, member B	ENST00000338896	ENSP00000344563	182	I/T	aTa/aCa	-	0	0.982	2.86	2.056	high_impact	1.182	medium_impact	1.95	medium_impact
7	Liver metastases	Private in metastatic tumor	4:141317068	CLGN	calmegin	ENST00000414773	ENSP00000392782	352	R/W	Cgg/Tgg	rs12513290	0	0.904	-0.305	2.124	high_impact	0.74	medium_impact	-1.392	low_impact
7	Liver	Private in	9:17340	CNT	centlein, centrosomal protein	ENST0	ENSP00	562	R/	Cgc/	rs380	0	0.9	1.995	0.266	mediu	2.179	high_impa	1.449	medium_imp

	metastases	metastatic tumor	864	LN		0000262360	000262360		C	Tgc	8782	.14	9			m_impact		ct		act
7	Primary tumor	Private in primary tumor	1:197396689	CRB1	crumbs homolog 1 (Drosophila)	ENST00000367401	ENSP00000356371	394	T/M	aCg/aTg	rs28939720	0	0.997	NA	2.075	high_impact	1.573	high_impact	NA	NA
7	Primary tumor	Private in primary tumor	8:2909992	CSMD1	CUB and Sushi multiple domains 1	ENST00000335551	ENSP00000334828	1969	T/M	aCg/aTg	rs6558702	0.14	0.998	NA	0.085	medium_impact	1.613	high_impact	NA	NA
7	Liver metastases	Private in metastatic tumor	15:75967926	CSPG4	chondroitin sulfate proteoglycan 4	ENST00000308508	ENSP00000312506	2312	N/H	Aac/Cac	-	0	0.998	2.31	2.128	high_impact	1.735	high_impact	1.274	medium_impact
7	Liver metastases	Private in metastatic tumor	6:43188273	CUL9	cullin 9	ENST00000372647	ENSP00000361730	2092	C/S	tGc/tCc	-	0	0.999	NA	1.981	medium_impact	1.955	high_impact	NA	NA
7	Liver metastases	Private in metastatic tumor	2:136872509	CXCR4	chemokine (C-X-C motif) receptor 4	ENST00000537957	ENSP00000440311	200	S/Y	tCc/tAc	-	0	0.887	NA	2.126	high_impact	0.046	medium_impact	NA	NA
7	Primary tumor	Private in primary tumor	19:49878196	DKKL1	dickkopf-like 1	ENST00000221498	ENSP00000221498	214	E/K	Gaa/Aaa	rs2288481	0	0.907	0.975	2.119	high_impact	0.734	medium_impact	-0.184	low_impact
7	Liver metastases	Private in metastatic tumor	7:21893993	DNAH11	dynein, axonemal, heavy chain 11	ENST00000421290	ENSP00000403651	103	V/L	Gtg/Ttg	rs4722064	0.04	0.998	NA	0.531	medium_impact	1.643	high_impact	NA	NA
7	Liver metastases	Private in metastatic tumor	6:38951998	DNAH8	dynein, axonemal, heavy chain 8	ENST00000441566	ENSP00000402294	4070	T/M	aCg/aTg	rs1537232	0	1	NA	2.056	high_impact	1.81	high_impact	NA	NA
7	Prima	Common in	4:34949	DO	docking protein 7	ENST0	ENSP00	415	P/	Cct/T	rs168	0	0.9	NA	0.453	mediu	1.819	high_impa	NA	NA

	ry tumor	primary and metastatic tumors	56	K7		0000389653	000374304		S	ct	44464	.06	98			m_impact		ct		
7	Liver metastases	Common in primary and metastatic tumors	4:3494956	DOK7	docking protein 7	ENST00000389653	ENSP00000374304	415	P/S	Cct/Tct	rs16844464	0.006	0.998	NA	0.453	medium_impact	1.819	high_impact	NA	NA
7	Liver metastases	Private in metastatic tumor	2:37341947	EIF2AK2	eukaryotic translation initiation factor 2-alpha kinase 2	ENST00000233057	ENSP00000233057	435	L/P	cTt/cCt	-	0	1	3.615	2.315	high_impact	1.766	high_impact	2.851	medium_impact
7	Primary tumor	Common in primary and metastatic tumors	1:21177771	EIF4G3	eukaryotic translation initiation factor 4 gamma, 3	ENST00000400415	ENSP00000383266	1391	R/Q	cGa/cAa	-	0.25	0.995	NA	-0.289	low_impact	1.629	high_impact	NA	NA
7	Liver metastases	Common in primary and metastatic tumors	1:21177771	EIF4G3	eukaryotic translation initiation factor 4 gamma, 3	ENST00000374935	ENSP00000364071	915	R/Q	cGa/cAa	-	0.4	0.997	NA	-0.624	low_impact	1.763	high_impact	NA	NA
7	Liver metastases	Private in metastatic tumor	2:55119614	EML6	echinoderm microtubule associated protein like 6	ENST00000356458	ENSP00000348842	855	G/R	Gga/Aga	-	0	0.997	3.18	2.062	high_impact	1.625	high_impact	2.269	medium_impact
7	Primary tumor	Common in primary and metastatic tumors	11:64083331	ESRRA	estrogen-related receptor alpha	ENST00000405666	ENSP00000384851	389	R/C	Cgc/Tgc	rs80310817	0	1	3.79	2.036	high_impact	1.644	high_impact	3.336	high_impact
7	Liver metastases	Common in primary and metastatic tumors	11:64083331	ESRRA	estrogen-related receptor alpha	ENST00000545035	ENSP00000444710	170	R/C	Cgc/Tgc	rs80310817	0	0.996	NA	2.036	high_impact	1.345	medium_impact	NA	NA
7	Prima	Common in	11:6408	ESR	estrogen-related receptor alpha	ENST0	ENSP00	387	L/	Ctc/T	rs792	0	0.9	NA	2.036	high_im	1.644	high_impa	NA	NA

	ry tumor	primary and metastatic tumors	3328	RA		0000406310	000385971		F	tc	04587		99			pact		ct		
7	Liver metastases	Common in primary and metastatic tumors	11:64083328	ESRRA	estrogen-related receptor alpha	ENST00000545035	ENSP00000444710	169	L/F	Ctc/Ttc	rs79204587	0	0.996	NA	2.036	high_impact	1.345	medium_impact	NA	NA
7	Primary tumor	Private in primary tumor	11:64083320	ESRRA	estrogen-related receptor alpha	ENST00000545035	ENSP00000444710	166	L/P	cTa/cCa	-	0	NA	NA	2.036	high_impact	NA	NA	NA	NA
7	Primary tumor	Private in primary tumor	6:170627629	FAM120B	family with sequence similarity 120B	ENST00000537664	ENSP00000440125	407	E/V	gAa/gTa	-	0.11	0.996	NA	0.09	medium_impact	1.579	high_impact	NA	NA
7	Primary tumor	Private in primary tumor	5:175535644	FAM153B	family with sequence similarity 153, member B	ENST00000253490	ENSP00000253490	300	Q/P	cAg/cCg	-	0	0.287	0.55	2.929	high_impact	-0.167	low_impact	-0.385	low_impact
7	Liver metastases	Private in metastatic tumor	2:242346973	FARP2	FERM, RhoGEF and pleckstrin domain protein 2	ENST00000545004	ENSP00000443876	118	R/H	cGc/cAc	-	0.02	0.998	NA	0.749	medium_impact	1.57	high_impact	NA	NA
7	Primary tumor	Common in primary and metastatic tumors	19:8183871	FBN3	fibrillin 3	ENST00000270509	ENSP00000270509	1083	R/W	Cgg/Tgg	rs35579498	0	0.993	2.095	2.145	high_impact	1.358	medium_impact	0.859	low_impact
7	Liver metastases	Common in primary and metastatic tumors	19:8183871	FBN3	fibrillin 3	ENST00000270509	ENSP00000270509	1083	R/W	Cgg/Tgg	rs35579498	0	0.993	2.095	2.145	high_impact	1.358	medium_impact	0.859	low_impact
7	Liver	Private in	19:4039	FC	Fc fragment of IgG binding protein	ENST0	ENSP00	2670	G/	Ggg/	-	0	0	3.345	2.062	high_im	-1.483	low_impa	2.442	medium_imp

	metastases	metastatic tumor	2496	GBP		0000221347	000221347		R	Agg						pact		ct		act
7	Primary tumor	Private in primary tumor	19:40398273	FCGBP	Fc fragment of IgG binding protein	ENST0000221347	ENSP00000221347	2232	W/R	Tgg/Cgg	-	0.08	0.86	4.05	0.276	medium_impact	0.649	medium_impact	3.181	high_impact
7	Primary tumor	Private in primary tumor	11:111724133	FDXACB1	ferredoxin-fold anticodon binding domain containing 1	ENST0000428306	ENSP00000387627	522	V/I	Gtc/Atc	rs10502151	0.19	0.984	NA	-0.272	low_impact	1.826	high_impact	NA	NA
7	Liver metastases	Private in metastatic tumor	2:216272908	FN1	fibronectin 1	ENST0000421182	ENSP00000394423	814	P/H	cCt/cAt	-	0	1	NA	1.931	medium_impact	1.608	high_impact	NA	NA
7	Primary tumor	Private in primary tumor	7:4802075	FOXK1	forkhead box K1	ENST0000450194	ENSP00000389711	484	G/R	Ggg/Agg	-	0	0.995	NA	1.754	medium_impact	1.507	high_impact	NA	NA
7	Primary tumor	Private in primary tumor	X:153760261	G6PD	glucose-6-phosphate dehydrogenase	ENST0000393564	ENSP00000377194	501	F/S	tTc/tCc	CM973154	0	1	2.13	2.055	high_impact	1.774	high_impact	0.403	low_impact
7	Liver metastases	Private in metastatic tumor	19:38876352	GGN	gametogenetin	ENST0000334928	ENSP00000334940	517	A/V	gCg/gTg	rs56854837	0	0	0	2.071	high_impact	-1.447	low_impact	-1.116	low_impact
7	Primary tumor	Private in primary tumor	19:1005428	GRIN3B	glutamate receptor, ionotropic, N-methyl-D-aspartate 3B	ENST0000234389	ENSP00000234389	643	R/P	cGc/cCc	-	0	1	3.085	2.378	high_impact	2.004	high_impact	2.048	medium_impact
7	Primary tumor	Private in primary tumor	12:96384250	HAL	histidine ammonia-lyase	ENST0000541929	ENSP00000446364	51	P/R	cCa/cGa	rs121434329	0	0.999	NA	1.88	medium_impact	1.742	high_impact	NA	NA
7	Liver metas	Private in metastatic	4:89385130	HERC5	hect domain and RLD 5	ENST000002	ENSP000002643	302	C/S	tGt/tCt	rs78613619	0	0.834	2.86	2.114	high_impact	0.785	medium_impact	1.744	medium_impact

	tases	tumor				64350	50													
7	Liver metastases	Private in metastatic tumor	19:37854040	HKR1	HKR1, GLI-Kruppel zinc finger family member	ENST00000414402	ENSP00000410650	227	R/H	cGt/cAt	rs2921563	0	0.998	NA	2.084	high_impact	1.818	high_impact	NA	NA
7	Liver metastases	Private in metastatic tumor	6:31239060	HLA-C	major histocompatibility complex, class I, C	ENST00000376237	ENSP00000365412	131	V/A	gTa/gCa	rs2308574	0	0	NA	2.062	high_impact	-1.483	low_impact	NA	NA
7	Primary tumor	Private in primary tumor	16:71127814	HYDIN	hydrocephalus inducing homolog (mouse)	ENST00000448089	ENSP00000398544	451	R/P	cGa/cCa	rs7200485	0.21	0.998	NA	-0.189	low_impact	1.613	high_impact	NA	NA
7	Primary tumor	Private in primary tumor	2:86400824	IMMT	inner membrane protein, mitochondrial	ENST00000409258	ENSP00000387237	124	P/S	Cct/Tct	rs1050301	0.08	0.996	NA	0.257	medium_impact	1.566	high_impact	NA	NA
7	Primary tumor	Private in primary tumor	9:95396712	IPPK	inositol 1,3,4,5,6-pentakisphosphate 2-kinase	ENST00000287996	ENSP00000287996	376	L/F	Ctt/Ttt	rs2277170	0.03	0.992	1.995	0.671	medium_impact	1.509	high_impact	0.92	low_impact
7	Liver metastases	Private in metastatic tumor	1:226923800	ITPKB	inositol 1,4,5-trisphosphate 3-kinase B	ENST00000272117	ENSP00000272117	454	G/W	Ggg/Tgg	-	0	0.988	0.805	1.699	medium_impact	1.702	high_impact	0.375	low_impact
7	Primary tumor	Common in primary and metastatic tumors	16:85695081	KIAA0182	KIAA0182	ENST00000253458	ENSP00000253458	657	R/Q	cGa/cAa	-	0.4	0.998	1.04	-0.538	low_impact	1.724	high_impact	0.024	low_impact
7	Liver metastases	Common in primary and metastatic tumors	16:85695081	KIAA0182	KIAA0182	ENST00000393243	ENSP00000376934	584	R/Q	cGa/cAa	-	0.4	0.998	NA	-0.538	low_impact	1.724	high_impact	NA	NA

7	Liver metastases	Private in metastatic tumor	19:51535366	KLK12	kallikrein-related peptidase 12	ENST0000531374	ENSP00000436121	75	H/D	Cac/Gac	-	0	0.275	NA	2.111	high_impact	-0.096	low_impact	NA	NA
7	Liver metastases	Private in metastatic tumor	17:39032654	KRT20	keratin 20	ENST0000167588	ENSP00000167588	412	V/F	Gtc/Ttc	-	0	0	3.285	2.111	high_impact	-1.545	low_impact	2.4	medium_impact
7	Liver metastases	Private in metastatic tumor	17:38922891	KRT26	keratin 26	ENST0000335552	ENSP00000334798	428	T/I	aCa/aTa	-	0	0.984	2.755	2.018	high_impact	1.012	medium_impact	1.448	medium_impact
7	Primary tumor	Common in primary and metastatic tumors	19:55179364	LILRB4	leukocyte immunoglobulin-like receptor, subfamily B (with TM and ITIM domains), member 4	ENST0000391736	ENSP00000375616	414	Q/R	cAg/cGg	rs1048801	0	0.016	2.845	2.01	high_impact	-0.63	low_impact	1.88	medium_impact
7	Liver metastases	Common in primary and metastatic tumors	19:55179364	LILRB4	leukocyte immunoglobulin-like receptor, subfamily B (with TM and ITIM domains), member 4	ENST0000391736	ENSP00000375616	414	Q/R	cAg/cGg	rs1048801	0	0.016	2.845	2.01	high_impact	-0.63	low_impact	1.88	medium_impact
7	Primary tumor	Private in primary tumor	7:20199472	MACC1	metastasis associated in colon cancer 1	ENST0000400331	ENSP00000383185	171	L/H	cTt/cAt	-	0.01	0.999	2.095	1.177	medium_impact	1.867	high_impact	0.957	low_impact
7	Primary tumor	Private in primary tumor	6:136681876	MAP7	microtubule-associated protein 7	ENST0000438100	ENSP00000400790	573	H/Y	Cat/Tat	-	0.07	0.999	NA	0.266	medium_impact	1.682	high_impact	NA	NA
7	Liver metastases	Private in metastatic tumor	1:11090916	MASP2	mannan-binding lectin serine peptidase 2	ENST0000400897	ENSP00000383690	371	D/Y	Gat/Tat	rs12711521	0.01	0.998	3.02	1.171	medium_impact	1.659	high_impact	2.162	medium_impact
7	Prima	Common in	5:15434	MR	mitochondrial ribosomal protein L22	ENST0	ENSP00	169	R/	Cga/	-	0	0.9	NA	2.159	high_im	0.759	medium_i	NA	NA

	ry tumor	primary and metastatic tumors	6263	PL22		0000439747	000411177		G	Gga			23			pact		mpact		
7	Liver metastases	Common in primary and metastatic tumors	5:154346263	MRPL22	mitochondrial ribosomal protein L22	ENST00000439747	ENSP00000411177	169	R/G	Cga/Gga	-	0	0.923	NA	2.159	high_impact	0.759	medium_impact	NA	NA
7	Primary tumor	Common in primary and metastatic tumors	14:75562077	NEK9	NIMA (never in mitosis gene a)- related kinase 9	ENST00000238616	ENSP00000238616	744	T/I	aCt/aTt	-	0	0.766	0.695	2.128	high_impact	0.519	medium_impact	-0.403	low_impact
7	Liver metastases	Common in primary and metastatic tumors	14:75562077	NEK9	NIMA (never in mitosis gene a)- related kinase 9	ENST00000238616	ENSP00000238616	744	T/I	aCt/aTt	-	0	0.766	0.695	2.128	high_impact	0.519	medium_impact	-0.403	low_impact
7	Liver metastases	Private in metastatic tumor	4:103867931	NHEDC1	Na+/H+ exchanger domain containing 1	ENST00000514340	ENSP00000426056	76	G/D	gGt/gAt	-	0	0.999	NA	2.383	high_impact	1.933	high_impact	NA	NA
7	Liver metastases	Private in metastatic tumor	19:56569629	NLRP5	NLR family, pyrin domain containing 5	ENST00000390649	ENSP00000375063	1108	S/C	tCt/tGt	rs12462795	0.04	0.998	3.17	0.55	medium_impact	1.666	high_impact	1.881	medium_impact
7	Liver metastases	Private in metastatic tumor	4:56502307	NMU	neuromedin U	ENST00000505262	ENSP00000424246	18	A/E	gCg/gAg	rs3828555	0	0.438	NA	2.026	high_impact	0.096	medium_impact	NA	NA
7	Primary tumor	Private in primary tumor	12:57619160	NXPH4	neurexophilin 4	ENST00000349394	ENSP00000333593	186	L/R	cTt/cGt	-	0	0.788	0	2.064	high_impact	0.523	medium_impact	-1.036	low_impact
7	Primary	Private in primary tumor	5:140593754	PCDH	protocadherin beta 13	ENST000003	ENSP000003454	20	L/P	cTt/cCt	-	0	0.918	3.38	2.075	high_impact	0.756	medium_impact	2.362	medium_impact

	tumor			B13		41948	91													
7	Primary tumor	Private in primary tumor	5:32090332	PDZD2	PDZ domain containing 2	ENST00000382161	ENSP00000371596	2061	E/K	Gag/Aag	-	0	0	NA	2.062	high_impact	-1.483	low_impact	NA	NA
7	Liver metastases	Private in metastatic tumor	12:123519086	PITPNM2	phosphatidylinositol transfer protein, membrane-associated 2	ENST00000542749	ENSP00000437611	18	R/C	Cgc/Tgc	-	0	0.999	NA	2.143	high_impact	1.883	high_impact	NA	NA
7	Primary tumor	Private in primary tumor	19:4513269	PLIN4	perilipin 4	ENST00000301286	ENSP00000301286	221	K/E	Aaa/Gaa	-	0.33	0.997	2.48	-0.534	low_impact	1.512	high_impact	1.727	medium_impact
7	Liver metastases	Private in metastatic tumor	14:39650823	PNN	pinin, desmosome associated protein	ENST00000216832	ENSP00000216832	637	G/V	gGc/gTc	-	0	0	2.605	2.018	high_impact	-1.636	low_impact	1.309	medium_impact
7	Liver metastases	Private in metastatic tumor	21:14982776	POTED	POTE ankyrin domain family, member D	ENST00000299443	ENSP00000299443	76	G/D	gGc/gAc	-	0	0.082	NA	2.062	high_impact	-0.385	low_impact	NA	NA
7	Liver metastases	Private in metastatic tumor	8:128428284	POU5F1B	POU class 5 homeobox 1B	ENST00000391675	ENSP00000375557	58	W/S	tGg/tCg	-	0	0.002	1.795	2.056	high_impact	-1.366	low_impact	0.719	low_impact
7	Primary tumor	Private in primary tumor	3:47452382	PTPN23	protein tyrosine phosphatase, non-receptor type 23	ENST00000265562	ENSP00000265562	1032	G/R	Ggg/Cgg	-	0.15	0.995	0.695	-0.042	low_impact	1.602	high_impact	-0.377	low_impact
7	Primary tumor	Private in primary tumor	19:5214592	PTPRS	protein tyrosine phosphatase, receptor type, S	ENST00000536396	ENSP00000441585	1087	R/W	Cgg/Tgg	-	0	0.997	NA	2.084	high_impact	1.697	high_impact	NA	NA
7	Liver metastases	Private in metastatic tumor	17:77099335	RBFOX3	RNA binding protein, fox-1 homolog (C. elegans) 3	ENST00000338834	ENSP00000344726	138	G/V	gGt/gTt	-	0	1	NA	2.056	high_impact	1.904	high_impact	NA	NA

7	Primary tumor	Private in primary tumor	1:24859664	RCAN3	RCAN family member 3	ENST00000374395	ENSP00000363516	154	P/Q	cCg/cAg	-	0	0.999	3.425	1.631	medium_impact	1.515	high_impact	2.367	medium_impact
7	Primary tumor	Private in primary tumor	11:73103450	RELT	RELT tumor necrosis factor receptor	ENST00000064780	ENSP00000064780	188	R/W	Cgg/Tgg	-	0	0.999	0.55	1.949	medium_impact	1.856	high_impact	-0.49	low_impact
7	Liver metastases	Private in metastatic tumor	10:43595983	RET	ret proto-oncogene	ENST00000535749	ENSP00000445963	50	L/F	ttG/ttT	-	0.01	0.998	NA	1.236	medium_impact	1.733	high_impact	NA	NA
7	Primary tumor	Private in primary tumor	13:25433204	RNF17	ring finger protein 17	ENST00000418120	ENSP00000388892	550	P/T	Cct/Act	-	1	0.998	NA	-3.754	low_impact	1.581	high_impact	NA	NA
7	Primary tumor	Common in primary and metastatic tumors	X:38147170	RPGR	retinitis pigmentosa GTPase regulator	ENST00000318842	ENSP00000322219	566	G/E	gGg/gAg	rs1801688	0.36	0.998	NA	-0.591	low_impact	1.654	high_impact	NA	NA
7	Liver metastases	Common in primary and metastatic tumors	X:38147170	RPGR	retinitis pigmentosa GTPase regulator	ENST00000339363	ENSP00000343671	566	G/E	gGg/gAg	rs1801688	0	0.998	0.975	1.936	medium_impact	1.654	high_impact	-0.036	low_impact
7	Liver metastases	Private in metastatic tumor	19:1440375	RPS15	ribosomal protein S15	ENST00000233609	ENSP00000233609	118	E/K	Gag/Aag	-	0	0.969	4.395	2.159	high_impact	0.998	medium_impact	2.909	medium_impact
7	Liver metastases	Private in metastatic tumor	X:18660135	RS1	retinoschisin 1	ENST00000379984	ENSP00000369320	222	K/Q	Aag/Cag	-	0.01	0.998	1.43	1.637	medium_impact	1.613	high_impact	0.578	low_impact
7	Liver metas	Private in metastatic	6:116950734	RSPH4A	radial spoke head 4 homolog A (Chlamydomonas)	ENST000003	ENSP000003575	309	R/H	cGc/cAc	rs6927567	0.	0.999	NA	1.128	medium_imp	1.91	high_impact	NA	NA

	tases	tumor				68580	69					01				act				
7	Primary tumor	Private in primary tumor	22:20785191	SCARF2	scavenger receptor class F, member 2	ENST00000341328	ENSP00000343561	287	G/R	Ggc/Cgc	-	0.03	0.999	0.975	0.641	medium_impact	1.885	high_impact	0.064	low_impact
7	Liver metastases	Private in metastatic tumor	16:29897054	SEZ6L2	seizure related 6 homolog (mouse)-like 2	ENST00000350527	ENSP00000310206	339	G/R	Ggg/Cgg	-	0	0.975	NA	2.929	high_impact	0.976	medium_impact	NA	NA
7	Liver metastases	Private in metastatic tumor	8:70591619	SLCO5A1	solute carrier organic anion transporter family, member 5A1	ENST00000260126	ENSP00000260126	673	T/I	aCa/aTa	-	0.16	0.997	0.85	0.006	medium_impact	1.566	high_impact	-0.323	low_impact
7	Primary tumor	Common in primary and metastatic tumors	16:3633486	SLX4	SLX4 structure-specific endonuclease subunit homolog (S. cerevisiae)	ENST00000294008	ENSP00000294008	1589	R/C	Cgc/Tgc	-	0	1	2.255	2.578	high_impact	2.09	high_impact	1.608	medium_impact
7	Liver metastases	Common in primary and metastatic tumors	16:3633486	SLX4	SLX4 structure-specific endonuclease subunit homolog (S. cerevisiae)	ENST00000294008	ENSP00000294008	1589	R/C	Cgc/Tgc	-	0	1	2.255	2.578	high_impact	2.09	high_impact	1.608	medium_impact
7	Liver metastases	Private in metastatic tumor	17:18166111	SMCR7	Smith-Magenis syndrome chromosome region, candidate 7	ENST00000395706	ENSP00000379057	37	A/D	gCc/gAc	-	0.01	0.995	NA	1.137	medium_impact	1.5	high_impact	NA	NA
7	Primary tumor	Private in primary tumor	19:49132410	SPHK2	sphingosine kinase 2	ENST00000443164	ENSP00000413369	511	G/R	Ggg/Cgg	-	0	0.99	NA	2.232	high_impact	1.651	high_impact	NA	NA
7	Liver metas	Private in metastatic	11:130064064	ST14	suppression of tumorigenicity 14 (colon carcinoma)	ENST000002	ENSP000002787	299	P/L	cCc/cTc	-	0.	0.999	2.51	0.843	medium_imp	1.828	high_impact	1.443	medium_impact

	tases	tumor				78742	42					02				act				
7	Primary tumor	Common in primary and metastatic tumors	14:62567295	SYT16	synaptotagmin XVI	ENST0000430451	ENSP00000394700	603	R/H	cGt/cAt	-	0.13	1	2.4	0.131	medium_impact	2.029	high_impact	1.273	medium_impact
7	Liver metastases	Common in primary and metastatic tumors	14:62567295	SYT16	synaptotagmin XVI	ENST0000430451	ENSP00000394700	603	R/H	cGt/cAt	-	0.13	1	2.4	0.131	medium_impact	2.029	high_impact	1.273	medium_impact
7	Primary tumor	Common in primary and metastatic tumors	2:160035208	TANC1	tetratricopeptide repeat, ankyrin repeat and coiled-coil containing 1	ENST0000454300	ENSP00000396339	576	G/R	Ggc/Cgc	-	0	0.996	NA	2.117	high_impact	1.537	high_impact	NA	NA
7	Liver metastases	Common in primary and metastatic tumors	2:160035208	TANC1	tetratricopeptide repeat, ankyrin repeat and coiled-coil containing 1	ENST0000454300	ENSP00000396339	576	G/R	Ggc/Cgc	-	0	0.996	NA	2.117	high_impact	1.537	high_impact	NA	NA
7	Liver metastases	Private in metastatic tumor	8:30704392	TEX15	testis expressed 15	ENST0000256246	ENSP00000256246	714	K/N	aaG/aaC	-	0	0.988	1.1	2.929	high_impact	1.163	medium_impact	0.217	low_impact
7	Primary tumor	Common in primary and metastatic tumors	11:2188714	TH	tyrosine hydroxylase	ENST0000416223	ENSP00000403440	30	G/S	Ggc/Agc	-	0	0.023	NA	2.366	high_impact	-0.408	low_impact	NA	NA
7	Liver metastases	Common in primary and metastatic tumors	11:2188714	TH	tyrosine hydroxylase	ENST0000416223	ENSP00000403440	30	G/S	Ggc/Agc	-	0	0.023	NA	2.366	high_impact	-0.408	low_impact	NA	NA
7	Prima	Private in	12:6420	TM	transmembrane protein 5	ENST0	ENSP00	308	P/	Cct/T	-	0	0.9	2.08	0.238	mediu	1.613	high_impa	1.29	medium_imp

	ry tumor	primary tumor	2462	EM5		0000261234	000261234		S	ct		.11	98			m_impact		ct		act
7	Liver metastases	Private in metastatic tumor	12:98909833	TMPO	thymopoietin	ENST0000343315	ENSP0000340251	96	P/R	cCg/cGg	-	0.01	0.996	NA	0.994	medium_impact	1.537	high_impact	NA	NA
7	Primary tumor	Common in primary and metastatic tumors	21:10943003	TPTE	transmembrane phosphatase with tensin homology	ENST0000342420	ENSP0000344441	157	R/Q	cGa/cAa	rs1810856	0	1	NA	1.924	medium_impact	1.891	high_impact	NA	NA
7	Liver metastases	Common in primary and metastatic tumors	21:10943003	TPTE	transmembrane phosphatase with tensin homology	ENST0000342420	ENSP0000344441	157	R/Q	cGa/cAa	rs1810856	0	1	NA	1.924	medium_impact	1.891	high_impact	NA	NA
7	Primary tumor	Private in primary tumor	1:156551848	TTC24	tetratricopeptide repeat domain 24	ENST0000340086	ENSP0000339487	4	E/G	gAg/gGg	rs6682716	0	0.205	NA	2.02	high_impact	-0.086	low_impact	NA	NA
7	Liver metastases	Private in metastatic tumor	6:139583814	TXLNB	taxilin beta	ENST0000358430	ENSP0000351206	262	E/K	Gag/Aag	-	0.04	0.999	2.175	0.846	medium_impact	1.787	high_impact	1.394	medium_impact
7	Liver metastases	Private in metastatic tumor	X:16846329	TXLNG	taxilin gamma	ENST0000398155	ENSP0000381222	72	A/V	gCa/gTa	-	0.4	0.999	NA	-0.703	low_impact	1.787	high_impact	NA	NA
7	Liver metastases	Private in metastatic tumor	10:12077223	UPF2	UPF2 regulator of nonsense transcripts homolog (yeast)	ENST0000357604	ENSP0000350221	67	K/R	aAg/aGg	-	0	0	0	2.02	high_impact	-1.457	low_impact	-1.112	low_impact
7	Primary	Common in primary and	16:23102027	USP31	ubiquitin specific peptidase 31	ENST000002	ENSP000002196	445	D/Y	Gat/Tat	rs1978066	0	0.873	0.85	2.021	high_impact	0.673	medium_impact	-0.104	low_impact

	tumor	metastatic tumors				19689	89													
7	Liver metastases	Common in primary and metastatic tumors	16:23102027	USP31	ubiquitin specific peptidase 31	ENST00000219689	ENSP00000219689	445	D/Y	Gat/Tat	rs1978066	0	0.873	0.85	2.021	high_impact	0.673	medium_impact	-0.104	low_impact
7	Primary tumor	Private in primary tumor	9:140459604	WDR85	WD repeat domain 85	ENST00000277540	ENSP00000277540	215	G/C	Ggc/Tgc	-	0	1	3.22	2.062	high_impact	1.894	high_impact	2.311	medium_impact
7	Liver metastases	Private in metastatic tumor	4:4322377	ZBTB49	zinc finger and BTB domain containing 49	ENST00000538529	ENSP00000445653	27	F/L	ttT/ttG	-	0	0.993	NA	2.064	high_impact	1.511	high_impact	NA	NA
7	Liver metastases	Private in metastatic tumor	8:40683111	ZMAT4	zinc finger, matrin-type 4	ENST00000519406	ENSP00000428423	29	R/C	Cgt/Tgt	-	0	0.986	NA	2.084	high_impact	1.325	medium_impact	NA	NA
7	Liver metastases	Private in metastatic tumor	19:53303705	ZNF28	zinc finger protein 28	ENST00000391783	ENSP00000375661	412	K/Q	Aaa/Caa	rs10417163	0	0.073	NA	2.084	high_impact	-0.386	low_impact	NA	NA
7	Primary tumor	Private in primary tumor	19:57910599	ZNF548	zinc finger protein 548	ENST00000366197	ENSP00000379482	315	G/E	gGg/gAg	-	0.06	0.993	1.225	0.43	medium_impact	1.501	high_impact	0.15	low_impact
7	Primary tumor	Common in primary and metastatic tumors	9:116770636	ZNF618	zinc finger protein 618	ENST00000374124	ENSP00000363239	186	E/G	gAa/gGa	-	0	0.904	NA	2.071	high_impact	0.779	medium_impact	NA	NA
7	Liver metastases	Common in primary and metastatic tumors	9:116770636	ZNF618	zinc finger protein 618	ENST00000374124	ENSP00000363239	186	E/G	gAa/gGa	-	0	0.904	NA	2.071	high_impact	0.779	medium_impact	NA	NA

7	Primary tumor	Private in primary tumor	1:120168654	ZNF697	zinc finger protein 697	ENST00000421812	ENSP00000396857	24	F/V	Ttt/Gtt	rs74702216	0	0.004	0.695	2.064	high_impact	-1.171	low_impact	-0.311	low_impact
7	Liver metastases	Private in metastatic tumor	16:30545862	ZNF747	zinc finger protein 747	ENST00000252799	ENSP00000252799	47	S/P	Tcc/Ccc	-	0	0.992	3.585	2.084	high_impact	1.467	medium_impact	2.338	medium_impact
8	Primary tumor	Common in primary and metastatic tumors	2:217124369	41702	membrane-associated ring finger (C3HC4) 4	ENST00000273067	ENSP00000273067	300	R/H	cGc/cAc	-	0.03	0.997	2.095	0.691	medium_impact	1.772	high_impact	1.22	medium_impact
8	Liver metastases	Common in primary and metastatic tumors	2:217124369	41702	membrane-associated ring finger (C3HC4) 4	ENST00000273067	ENSP00000273067	300	R/H	cGc/cAc	-	0.03	0.997	2.095	0.691	medium_impact	1.772	high_impact	1.22	medium_impact
8	Liver metastases	Private in metastatic tumor	12:43821125	ADAMTS20	ADAM metallopeptidase with thrombospondin type 1 motif, 20	ENST00000395541	ENSP00000378911	483	G/R	Gga/Aga	-	0	0.062	NA	2.29	high_impact	-0.19	low_impact	NA	NA
8	Liver metastases	Private in metastatic tumor	10:51748684	AGAP6	ArfGAP with GTPase domain, ankyrin repeat and PH domain 6	ENST00000412531	ENSP00000400972	70	R/Q	cGg/cAg	rs77235195	0	0.51	1.34	2.067	high_impact	0.211	medium_impact	0.132	low_impact
8	Liver metastases	Private in metastatic tumor	7:100151813	AGFG2	ArfGAP with FG repeats 2	ENST00000300176	ENSP00000300176	228	D/V	gAc/gTc	-	0	0.996	2.215	2.067	high_impact	1.582	high_impact	1.027	medium_impact
8	Primary tumor	Private in primary tumor	2:97505297	ANKRD23	ankyrin repeat domain 23	ENST00000318357	ENSP00000321679	303	T/P	Acc/Ccc	rs74826568	0	0.698	0.695	2.062	high_impact	0.41	medium_impact	-0.338	low_impact
8	Liver metastases	Private in metastatic tumor	19:3754094	APBA3	amyloid beta (A4) precursor protein-binding, family A, member 3	ENST00000316757	ENSP00000315136	258	G/R	Ggg/Agg	-	0	0.997	2.54	2.062	high_impact	1.625	high_impact	1.597	medium_impact
8	Prima	Common in	2:25966	ASX	additional sex combs like 2 (Drosophila)	ENST0	ENSP00	976	E/	gAa/	-	0	0.9	2.255	2.049	high_im	0.982	medium_i	1.122	medium_imp

	ry tumor	primary and metastatic tumors	279	L2		0000435504	000391447		G	gGa			55			pact		mpact		act
8	Liver metastases	Common in primary and metastatic tumors	2:25966279	ASXL2	additional sex combs like 2 (Drosophila)	ENST00000336112	ENSP00000337250	948	E/G	gAa/gGa	-	0	0.955	NA	2.049	high_impact	0.982	medium_impact	NA	NA
8	Primary tumor	Private in primary tumor	2:220085471	ATG9A	ATG9 autophagy related 9 homolog A (S. cerevisiae)	ENST00000409618	ENSP00000386710	838	K/E	Aag/Gag	-	0	0.832	0.975	2.105	high_impact	0.777	medium_impact	-0.449	low_impact
8	Primary tumor	Common in primary and metastatic tumors	17:79419010	BAHCC1	BAH domain and coiled-coil containing 1	ENST00000307745	ENSP00000303486	1398	K/T	aAg/aCg	-	0	0.953	NA	2.084	high_impact	1.012	medium_impact	NA	NA
8	Liver metastases	Common in primary and metastatic tumors	17:79419010	BAHCC1	BAH domain and coiled-coil containing 1	ENST00000307745	ENSP00000303486	1398	K/T	aAg/aCg	-	0	0.953	NA	2.084	high_impact	1.012	medium_impact	NA	NA
8	Primary tumor	Private in primary tumor	19:15353743	BRD4	bromodomain containing 4	ENST00000263377	ENSP00000263377	1046	P/H	cCc/cAc	-	0	0	1.355	2.064	high_impact	-1.491	low_impact	0.343	low_impact
8	Primary tumor	Private in primary tumor	11:6232189	C11orf42	chromosome 11 open reading frame 42	ENST00000316375	ENSP00000321021	307	G/R	Ggg/Cgg	-	0	0.948	0.695	2.929	high_impact	0.786	medium_impact	-0.227	low_impact
8	Liver metastases	Private in metastatic tumor	12:112654894	C12orf51	chromosome 12 open reading frame 51	ENST00000430131	ENSP00000404379	1972	G/R	Ggg/Cgg	-	NA	0.998	0.345	NA	NA	2.019	high_impact	-0.859	low_impact
8	Primary tumor	Private in primary tumor	14:74196543	C14orf43	chromosome 14 open reading frame 43	ENST00000423556	ENSP00000407767	632	S/I	aGc/aTc	-	0	0.996	2.545	2.064	high_impact	1.653	high_impact	1.528	medium_impact

8	Primary tumor	Private in primary tumor	7:48081095	C7orf57	chromosome 7 open reading frame 57	ENST0000034890 4	ENSP0000335500	74	A/S	Gcg/Tcg	rs10951942	0	0.993	2.32	2.929	high_impact	1.299	medium_impact	1.552	medium_impact
8	Liver metastases	Private in metastatic tumor	22:37904603	CARD10	caspase recruitment domain family, member 10	ENST00000406271	ENSP00000385799	46	R/S	agG/agT	-	0.01	0.996	NA	1.136	medium_impact	1.779	high_impact	NA	NA
8	Primary tumor	Common in primary and metastatic tumors	14:91739666	CCDC88C	coiled-coil domain containing 88C	ENST00000389857	ENSP00000374507	1797	R/H	cGc/cAc	-	0.01	0.997	2.045	1.141	medium_impact	1.709	high_impact	1.279	medium_impact
8	Liver metastases	Common in primary and metastatic tumors	14:91739666	CCDC88C	coiled-coil domain containing 88C	ENST00000334448	ENSP00000335593	247	R/H	cGc/cAc	-	0.18	0.997	NA	0.002	medium_impact	1.709	high_impact	NA	NA
8	Primary tumor	Private in primary tumor	2:73477453	CCT7	chaperonin containing TCP1, subunit 7 (eta)	ENST00000539919	ENSP00000437824	320	C/S	Tgc/Agc	-	0	0.993	NA	2.124	high_impact	1.415	medium_impact	NA	NA
8	Primary tumor	Common in primary and metastatic tumors	22:42341308	CENPM	centromere protein M	ENST00000402420	ENSP00000384132	72	S/P	Tct/Cct	rs7293091	0	0.796	NA	2.929	high_impact	0.4	medium_impact	NA	NA
8	Liver metastases	Common in primary and metastatic tumors	22:42341308	CENPM	centromere protein M	ENST00000402420	ENSP00000384132	72	S/P	Tct/Cct	rs7293091	0	0.796	NA	2.929	high_impact	0.4	medium_impact	NA	NA
8	Primary tumor	Private in primary tumor	2:175618973	CHRNA1	cholinergic receptor, nicotinic, alpha 1 (muscle)	ENST00000261007	ENSP00000261007	197	D/N	Gac/Aac	-	0.16	0.999	1.695	-0.291	low_impact	1.686	high_impact	0.204	low_impact

8	Liver metastases	Private in metastatic tumor	11:66049743	CNIH2	cornichon homolog 2 (Drosophila)	ENST00000528063	ENSP00000435537	32	D/G	gAt/gGt	-	0	0.937	NA	2.064	high_impact	0.867	medium_impact	NA	NA
8	Primary tumor	Common in primary and metastatic tumors	17:13980350	COX10	COX10 homolog, cytochrome c oxidase assembly protein, heme A: farnesyltransferase (yeast)	ENST00000429152	ENSP00000397750	120	R/Q	cGa/cAa	rs8077302	0	0.989	NA	3.121	high_impact	2.302	high_impact	NA	NA
8	Liver metastases	Common in primary and metastatic tumors	17:13980350	COX10	COX10 homolog, cytochrome c oxidase assembly protein, heme A: farnesyltransferase (yeast)	ENST00000429152	ENSP00000397750	120	R/Q	cGa/cAa	rs8077302	0	0.989	NA	3.121	high_impact	2.302	high_impact	NA	NA
8	Liver metastases	Private in metastatic tumor	8:68421768	CPA6	carboxypeptidase A6	ENST00000518549	ENSP00000431112	173	S/C	tCt/tGt	rs17853192	0	0.95	NA	2.042	high_impact	1.034	medium_impact	NA	NA
8	Liver metastases	Private in metastatic tumor	X:117712522	DOCK11	dedicator of cytokinesis 11	ENST00000276202	ENSP00000276202	475	I/S	aTt/aGt	-	0	0.988	3.365	2.06	high_impact	1.123	medium_impact	1.853	medium_impact
8	Primary tumor	Common in primary and metastatic tumors	17:48457722	EME1	essential meiotic endonuclease 1 homolog 1 (S. pombe)	ENST00000338165	ENSP00000339897	466	A/P	Gct/Cct	rs77309724	0.14	0.998	2.645	-0.1	low_impact	1.722	high_impact	1.348	medium_impact
8	Liver metastases	Common in primary and metastatic tumors	17:48457722	EME1	essential meiotic endonuclease 1 homolog 1 (S. pombe)	ENST00000510246	ENSP00000425567	278	A/P	Gct/Cct	rs77309724	0.08	0.996	NA	0.153	medium_impact	1.557	high_impact	NA	NA
8	Liver metastases	Private in metastatic tumor	19:16535987	EPS15L1	epidermal growth factor receptor pathway substrate 15-like 1	ENST00000535753	ENSP00000440103	233	K/N	aaA/aaC	-	0.09	0.996	NA	0.103	medium_impact	1.762	high_impact	NA	NA
8	Prima	Private in	7:10239	FA	family with sequence similarity 185, member	ENST0	ENSP00	179	G/	Ggg/	-	0	1	NA	2.929	high_im	1.787	high_impa	NA	NA

	ry tumor	primary tumor	2222	M185A	A	0000418198	000410034		R	Cgg						pact		ct		
8	Liver metastases	Private in metastatic tumor	19:10426597	FDX1L	ferredoxin 1-like	ENST00000453681	ENSP00000404383	26	R/G	Aga/Gga	rs62640397	0	0.775	NA	2.038	high_impact	0.373	medium_impact	NA	NA
8	Liver metastases	Private in metastatic tumor	X:153760261	G6PD	glucose-6-phosphate dehydrogenase	ENST00000291567	ENSP00000291567	501	F/S	tTc/tCc	CM973154	0	1	2.13	2.055	high_impact	1.774	high_impact	0.403	low_impact
8	Liver metastases	Private in metastatic tumor	2:121726358	GLI2	GLI family zinc finger 2	ENST00000360874	ENSP00000441454	100	T/S	aCt/aGt	-	0	0	NA	2.063	high_impact	-1.461	low_impact	NA	NA
8	Primary tumor	Common in primary and metastatic tumors	16:20327359	GP2	glycoprotein 2 (zymogen granule membrane)	ENST00000381362	ENSP00000370767	477	C/G	Tgc/Ggc	-	0	0.994	3.32	2.929	high_impact	1.338	medium_impact	2.647	medium_impact
8	Liver metastases	Common in primary and metastatic tumors	16:20327359	GP2	glycoprotein 2 (zymogen granule membrane)	ENST00000341642	ENSP00000343861	327	C/G	Tgc/Ggc	-	0	0.984	NA	2.929	high_impact	1.09	medium_impact	NA	NA
8	Liver metastases	Private in metastatic tumor	16:10031919	GRIN2A	glutamate receptor, ionotropic, N-methyl D-aspartate 2A	ENST00000535259	ENSP00000441572	145	A/T	Gct/Act	-	0.01	0.999	NA	1.063	medium_impact	2.004	high_impact	NA	NA
8	Liver metastases	Private in metastatic tumor	15:65678268	IGDCC4	immunoglobulin superfamily, DCC subclass, member 4	ENST00000356152	ENSP00000348473	756	Q/H	caG/caC	-	0	0.84	NA	2.062	high_impact	0.611	medium_impact	NA	NA
8	Primary tumor	Private in primary tumor	11:133806080	IGSF9B	immunoglobulin superfamily, member 9B	ENST00000527648	ENSP00000436576	230	F/S	tTc/tCc	-	0	0.404	NA	2.062	high_impact	0.11	medium_impact	NA	NA
8	Prima	Private in	5:36000	IRX	iroquois homeobox 1	ENST0	ENSP00	320	A/	gCg/	-	0	0.9	2.34	2.056	high_im	1.758	high_impa	1.258	medium_imp

	ry tumor	primary tumor	21	1		0000302006	000305244		E	gAg			98			pact		ct		act
8	Liver metastases	Private in metastatic tumor	15:74468391	ISLR	immunoglobulin superfamily containing leucine-rich repeat	ENST00000395118	ENSP00000378550	398	A/T	Gct/Act	-	0	0	0.205	2.062	high_impact	-1.483	low_impact	-0.852	low_impact
8	Liver metastases	Private in metastatic tumor	12:52822517	KRT75	keratin 75	ENST00000252245	ENSP00000252245	349	L/Q	cTg/cAg	-	0	0.998	4.06	2.018	high_impact	1.515	high_impact	2.66	medium_impact
8	Liver metastases	Private in metastatic tumor	21:45999880	KRTAP10-5	keratin associated protein 10-5	ENST00000400372	ENSP00000383223	192	C/W	tgC/tgG	-	0	0.998	2.92	2.929	high_impact	1.613	high_impact	2.209	medium_impact
8	Primary tumor	Private in primary tumor	17:39203455	KRTAP2-1	keratin associated protein 2-1	ENST00000391419	ENSP00000375238	22	P/R	cCc/cGc	rs80149678	0	0.981	2.285	2.929	high_impact	1.046	medium_impact	1.514	medium_impact
8	Liver metastases	Private in metastatic tumor	20:42143381	L3MBTL1	l(3)mbt-like 1 (Drosophila)	ENST00000457824	ENSP00000415487	54	P/R	cCg/cGg	-	0	0.94	NA	2.037	high_impact	0.991	medium_impact	NA	NA
8	Liver metastases	Private in metastatic tumor	21:15524921	LIPI	lipase, member I	ENST00000344577	ENSP00000343331	385	G/E	gGa/gAa	rs74369337	0	0.979	NA	2.044	high_impact	1.148	medium_impact	NA	NA
8	Liver metastases	Private in metastatic tumor	X:140993690	MAGEC1	melanoma antigen family C, 1	ENST00000285879	ENSP00000285879	167	V/E	gTg/gAg	-	0	0	0	2.062	high_impact	-1.483	low_impact	-1.067	low_impact
8	Liver metastases	Private in metastatic tumor	3:47957956	MAP4	microtubule-associated protein 4	ENST00000426837	ENSP00000407602	471	P/R	cCg/cGg	-	0	0	NA	2.018	high_impact	-1.636	low_impact	NA	NA
8	Primary tumor	Private in primary tumor	6:30672257	MDC1	mediator of DNA-damage checkpoint 1	ENST00000376405	ENSP00000365587	1304	P/H	cCt/cAt	-	0	0.948	NA	2.082	high_impact	0.9	medium_impact	NA	NA
8	Prima	Private in	6:30672	MD	mediator of DNA-damage checkpoint 1	ENST0	ENSP00	216	L/	Ctg/	-	0	0	NA	2.082	high_im	-1.654	low_impa	NA	NA

	ry tumor	primary tumor	257	C1		0000420817	000411775		M	Atg						pact		ct		
8	Liver metastases	Private in metastatic tumor	19:42838217	MEGF8	multiple EGF-like-domains 8	ENST00000334370	ENSP00000334219	137	F/C	tTc/tGc	-	0	0.056	NA	2.018	high_impact	-0.656	low_impact	NA	NA
8	Liver metastases	Private in metastatic tumor	18:48703653	MEX3C	mex-3 homolog C (C. elegans)	ENST00000406189	ENSP00000385610	350	K/Q	Aaa/Caa	-	0.01	0.999	2.9	1.126	medium_impact	1.904	high_impact	1.872	medium_impact
8	Primary tumor	Private in primary tumor	11:12348790	MICALCL	MICAL C-terminal like	ENST00000256186	ENSP00000256186	569	E/A	gAg/gCg	-	0	1	3.515	1.89	medium_impact	2.063	high_impact	3.079	high_impact
8	Liver metastases	Private in metastatic tumor	16:55532269	MMP2	matrix metallopeptidase 2 (gelatinase A, 72kDa gelatinase, 72kDa type IV collagenase)	ENST00000543485	ENSP00000444143	484	G/R	Gga/Aga	-	0.03	0.998	NA	0.727	medium_impact	1.687	high_impact	NA	NA
8	Liver metastases	Private in metastatic tumor	2:95772187	MRPS5	mitochondrial ribosomal protein S5	ENST00000345084	ENSP00000341660	218	F/C	tTt/tGt	-	0	1	NA	2.159	high_impact	1.861	high_impact	NA	NA
8	Liver metastases	Private in metastatic tumor	3:195495916	MUC4	mucin 4, cell surface associated	ENST00000478156	ENSP00000419798	4502	N/K	aaC/aaG	rs2550240	NA	0.993	NA	NA	NA	1.571	high_impact	NA	NA
8	Primary tumor	Private in primary tumor	17:10362585	MYH4	myosin, heavy chain 4, skeletal muscle	ENST00000255381	ENSP00000255381	524	I/F	Atc/Ttc	-	0	0.999	3.5	1.972	medium_impact	1.657	high_impact	1.29	medium_impact
8	Primary tumor	Common in primary and metastatic tumors	19:8613137	MYO1F	myosin IF	ENST00000305795	ENSP00000304899	434	G/D	gGc/gAc	-	0	0.999	NA	1.879	medium_impact	1.685	high_impact	NA	NA
8	Liver	Common in	19:8613	MY	myosin IF	ENST0	ENSP00	389	G/	gGc/	-	0	1	4.87	1.879	mediu	1.685	high_impa	2.918	medium_imp

	metastases	primary and metastatic tumors	137	O1F		0000338257	000344871		D	gAc						m_impact		ct		act
8	Primary tumor	Common in primary and metastatic tumors	1:24406636	MYOM3	myomesin family, member 3	ENST0000330966	ENSP0000332670	820	V/G	gTg/gGg	-	0	0.793	NA	2.062	high_impact	0.534	medium_impact	NA	NA
8	Liver metastases	Common in primary and metastatic tumors	1:24406636	MYOM3	myomesin family, member 3	ENST0000374434	ENSP0000363557	819	V/G	gTg/gGg	-	0	0.24	1.35	2.062	high_impact	-0.077	low_impact	0.349	low_impact
8	Primary tumor	Private in primary tumor	20:62191616	NA	NA	ENST0000427522	ENSP0000393257	1953	T/M	aCg/aTg	-	0	1	NA	1.92	medium_impact	1.762	high_impact	NA	NA
8	Liver metastases	Private in metastatic tumor	20:62193020	NA	NA	ENST0000467148	ENSP0000417401	2257	P/Q	cCg/cAg	-	0	0.999	2.265	1.92	medium_impact	1.762	high_impact	1.531	medium_impact
8	Liver metastases	Private in metastatic tumor	7:45121999	NACAD	NAC alpha domain containing	ENST0000490531	ENSP0000420477	1260	E/D	gaA/gaC	-	0	0.217	1.04	2.929	high_impact	-0.261	low_impact	0.151	low_impact
8	Liver metastases	Private in metastatic tumor	2:152417135	NEB	nebulin	ENST0000172853	ENSP0000172853	4728	A/T	Gcc/Acc	-	0	0.984	2.705	2.154	high_impact	0.845	medium_impact	1.768	medium_impact
8	Primary tumor	Private in primary tumor	9:33466323	NOL6	nucleolar protein family 6 (RNA-associated)	ENST0000297990	ENSP0000297990	731	Y/C	tAc/tGc	-	0.01	0.996	0.97	1.126	medium_impact	1.563	high_impact	-0.126	low_impact
8	Primary tumor	Private in primary tumor	1:145281613	NOTCH2NL	notch 2 N-terminal like	ENST0000362074	ENSP0000354929	181	S/R	agC/agA	rs12120756	0.1	0.998	1.275	0.158	medium_impact	1.671	high_impact	0.236	low_impact

												1								
8	Liver metastases	Private in metastatic tumor	15:69331276	NOX5	NADPH oxidase, EF-hand calcium binding domain 5	ENST00000530406	ENSP00000432440	456	I/T	aTt/aCt	-	0	0.998	NA	2.031	high_impact	1.12	medium_impact	NA	NA
8	Liver metastases	Private in metastatic tumor	10:55955444	PCDH15	protocadherin-related 15	ENST00000395440	ENSP00000378827	435	D/A	gAt/gCt	rs4935502	0	0.889	NA	2.075	high_impact	0.675	medium_impact	NA	NA
8	Liver metastases	Private in metastatic tumor	2:68607972	PLEK	pleckstrin	ENST00000234313	ENSP00000234313	106	G/C	Ggc/Tgc	-	0.002	0.998	1.795	0.662	medium_impact	1.612	high_impact	0.458	low_impact
8	Liver metastases	Private in metastatic tumor	22:50726459	PLXNB2	plexin B2	ENST00000414275	ENSP00000408377	463	R/Q	cGg/cAg	-	0.002	0.998	NA	0.888	medium_impact	1.792	high_impact	NA	NA
8	Liver metastases	Private in metastatic tumor	6:31106499	PSORS1C1	psoriasis susceptibility 1 candidate 1	ENST00000259881	ENSP00000259881	37	R/H	cGt/cAt	rs9263726	0	0.302	0	2.929	high_impact	-0.149	low_impact	-0.987	low_impact
8	Primary tumor	Private in primary tumor	1:109823031	PSRC1	proline/serine-rich coiled-coil 1	ENST00000409267	ENSP00000386323	329	P/S	Cca/Tca	-	0	0.07	NA	2.07	high_impact	-0.406	low_impact	NA	NA
8	Primary tumor	Private in primary tumor	13:98086993	RAP2A	RAP2A, member of RAS oncogene family	ENST00000245304	ENSP00000245304	90	F/S	tTc/tCc	-	0	0.994	4.37	1.937	medium_impact	1.556	high_impact	2.575	medium_impact
8	Liver metastases	Private in metastatic tumor	3:38755495	SCN10A	sodium channel, voltage-gated, type X, alpha subunit	ENST00000449082	ENSP00000390600	1253	R/H	cGc/cAc	-	0	1	5.29	2.08	high_impact	1.723	high_impact	3.637	high_impact
8	Primary	Common in primary and	6:100868686	SIM1	single-minded homolog 1 (Drosophila)	ENST000002	ENSP00002629	383	R/G	Agg/Ggg	-	0	0.997	0.975	2.056	high_impact	1.656	high_impact	-0.091	low_impact

	tumor	metastatic tumors				62901	01													
8	Liver metastases	Common in primary and metastatic tumors	6:100868686	SIM1	single-minded homolog 1 (Drosophila)	ENST00000262901	ENSP00000262901	383	R/G	Agg/Ggg	-	0	0.997	0.975	2.056	high_impact	1.656	high_impact	-0.091	low_impact
8	Primary tumor	Private in primary tumor	1:153934772	SLC39A1	solute carrier family 39 (zinc transporter), member 1	ENST00000368623	ENSP00000357612	81	L/W	tTg/tGg	-	0	1	2.725	2.016	high_impact	2.084	high_impact	2.036	medium_impact
8	Liver metastases	Private in metastatic tumor	X:142717240	SLITRK4	SLIT and NTRK-like family, member 4	ENST00000356928	ENSP00000349400	562	T/M	aCg/aTg	-	0	0.175	1.545	2.062	high_impact	-0.174	low_impact	0.553	low_impact
8	Liver metastases	Private in metastatic tumor	2:231036796	SP110	SP110 nuclear body protein	ENST00000358662	ENSP00000351488	601	P/S	Cct/Tct	-	0	0.731	2.485	2.119	high_impact	0.424	medium_impact	1.276	medium_impact
8	Liver metastases	Private in metastatic tumor	19:56029820	SSC5D	scavenger receptor cysteine rich domain containing (5 domains)	ENST00000389623	ENSP00000374274	1393	P/S	Ccc/Tcc	-	0	0	0.205	2.091	high_impact	-1.824	low_impact	-0.7	low_impact
8	Liver metastases	Private in metastatic tumor	19:56005129	SSC5D	scavenger receptor cysteine rich domain containing (5 domains)	ENST00000541230	ENSP00000444330	355	G/R	Ggg/Agg	-	0.19	1	NA	-0.216	low_impact	1.885	high_impact	NA	NA
8	Liver metastases	Private in metastatic tumor	11:8747735	ST5	suppression of tumorigenicity 5	ENST00000447053	ENSP00000397400	64	D/E	gaT/gaG	-	0	0.993	NA	2.062	high_impact	1.417	medium_impact	NA	NA
8	Primary tumor	Common in primary and metastatic tumors	3:52558008	STAB1	stabilin 1	ENST00000469989	ENSP00000418426	113	M/T	aTg/aCg	rs13303	0.996	0.998	NA	-2.049	low_impact	1.55	high_impact	NA	NA
8	Liver	Common in	3:52558	STA	stabilin 1	ENST0	ENSP00	113	M/	aTg/	rs133	0	0.9	NA	-2.04	low_im	1.55	high_impa	NA	NA

	metastases	primary and metastatic tumors	008	B1		0000469989	000418426		T	aCg	03	.96	98		9	pact		ct		
8	Primary tumor	Private in primary tumor	2:191843641	STAT1	signal transducer and activator of transcription 1, 91kDa	ENST00000392322	ENSP00000376136	605	E/G	gAg/gGg	-	0	0.999	2.8	2.119	high_impact	1.856	high_impact	1.581	medium_impact
8	Primary tumor	Private in primary tumor	19:1611816	TCF3	transcription factor 3 (E2A immunoglobulin enhancer binding factors E12/E47)	ENST00000453954	ENSP00000396363	616	K/E	Aaa/Gaa	-	0	0.997	NA	3.104	high_impact	1.602	high_impact	NA	NA
8	Liver metastases	Private in metastatic tumor	1:218520046	TGFB2	transforming growth factor, beta 2	ENST00000366930	ENSP00000355897	1	M/I	atG/atA	-	0	0.366	NA	2.135	high_impact	0.029	medium_impact	NA	NA
8	Liver metastases	Private in metastatic tumor	15:39880359	THBS1	thrombospondin 1	ENST00000260356	ENSP00000260356	471	G/R	Ggg/Agg	-	0	1	3.465	1.712	medium_impact	1.973	high_impact	4.684	high_impact
8	Primary tumor	Common in primary and metastatic tumors	17:7578526	TP53	tumor protein p53	ENST00000514944	ENSP00000423862	42	C/F	tGc/tTc	COSM10647	0	0.998	NA	2.265	high_impact	1.98	high_impact	NA	NA
8	Liver metastases	Common in primary and metastatic tumors	17:7578526	TP53	tumor protein p53	ENST00000396473	ENSP00000379735	124	C/F	tGc/tTc	COSM10647	0	1	NA	2.265	high_impact	2.147	high_impact	NA	NA
8	Liver metastases	Private in metastatic tumor	16:1272080	TPSG1	tryptase gamma 1	ENST00000234798	ENSP00000234798	225	P/R	cCt/cGt	-	0	0.997	4.69	2.111	high_impact	1.559	high_impact	3.683	high_impact
8	Liver metastases	Private in metastatic tumor	1:114973428	TRIM33	tripartite motif containing 33	ENST00000448034	ENSP00000402333	120	Q/K	Cag/Aag	-	0.02	0.996	NA	1.262	medium_impact	2.195	high_impact	NA	NA

8	Liver metastases	Private in metastatic tumor	22:38121795	TRIOBP	TRIO and F-actin binding protein	ENST00000417174	ENSP00000415209	1078	R/C	Cgc/Tgc	-	0	1	NA	1.948	medium_impact	1.56	high_impact	NA	NA
8	Primary tumor	Common in primary and metastatic tumors	11:76507282	TSKU	tsukushi small leucine rich proteoglycan homolog (Xenopus laevis)	ENST00000333090	ENSP00000332668	208	R/C	Cgc/Tgc	rs3740772	0	1	2.185	2.062	high_impact	1.894	high_impact	1.225	medium_impact
8	Liver metastases	Common in primary and metastatic tumors	11:76507282	TSKU	tsukushi small leucine rich proteoglycan homolog (Xenopus laevis)	ENST00000439807	ENSP00000387819	176	R/C	Cgc/Tgc	rs3740772	0	0.979	NA	2.062	high_impact	1.145	medium_impact	NA	NA
8	Primary tumor	Private in primary tumor	1:215847574	USH2A	Usher syndrome 2A (autosomal recessive, mild)	ENST00000307340	ENSP00000305941	4560	G/D	gGt/gAt	-	0	1	3.395	2.197	high_impact	1.708	high_impact	3.646	high_impact
8	Primary tumor	Private in primary tumor	19:44056412	XRCC1	X-ray repair complementing defective repair in Chinese hamster cells 1	ENST00000543982	ENSP00000443671	249	R/H	cGt/cAt	rs25489	0.16	0.993	NA	-0.114	low_impact	1.515	high_impact	NA	NA
8	Primary tumor	Private in primary tumor	14:24002614	ZFHX2	zinc finger homeobox 2	ENST00000419474	ENSP00000413418	641	G/R	Ggg/Cgg	-	0	0	1.525	2.056	high_impact	-1.54	low_impact	0.453	low_impact
8	Liver metastases	Private in metastatic tumor	11:64852613	ZFPL1	zinc finger protein-like 1	ENST00000526945	ENSP00000432529	37	W/C	tgG/tgC	-	0	1	NA	2.064	high_impact	2.005	high_impact	NA	NA
8	Liver metastases	Private in metastatic tumor	19:58118393	ZNF530	zinc finger protein 530	ENST00000332854	ENSP00000332861	500	F/L	ttT/ttA	-	0	0.955	3.025	2.084	high_impact	1.023	medium_impact	1.819	medium_impact
8	Liver metastases	Private in metastatic tumor	20:57828953	ZNF831	zinc finger protein 831	ENST00000371030	ENSP00000360069	1397	D/Y	Gat/Tat	-	0	0.47	1.24	2.084	high_impact	0.224	medium_impact	0.164	low_impact

8	Liver metastases	Private in metastatic tumor	19:20215097	ZNF90	zinc finger protein 90	ENST00000418063	ENSP00000410466	18	W/S	tGg/tCg	-	0	0.999	4.72	2.066	high_impact	1.96	high_impact	3.994	high_impact
9	Liver metastases	Private in metastatic tumor	1:6445639	ACOT7	acyl-CoA thioesterase 7	ENST00000377855	ENSP00000367086	30	G/W	Ggg/Tgg	-	0	0.717	1.39	3.041	high_impact	1.335	medium_impact	0.619	low_impact
9	Primary tumor	Private in primary tumor	7:100246370	ACTL6B	actin-like 6B	ENST00000160382	ENSP00000160382	182	G/S	Ggc/Agc	-	0	0.795	3.21	2.111	high_impact	0.51	medium_impact	2.318	medium_impact
9	Primary tumor	Common in primary and metastatic tumors	1:236902815	ACTN2	actinin, alpha 2	ENST00000545611	ENSP00000443955	133	E/K	Gag/Aag	-	0	0.972	NA	2.661	high_impact	1.269	medium_impact	NA	NA
9	Liver metastases	Common in primary and metastatic tumors	1:236902815	ACTN2	actinin, alpha 2	ENST00000366578	ENSP00000355537	364	E/K	Gag/Aag	-	0	0.371	3.365	2.661	high_impact	0.352	medium_impact	1.65	medium_impact
9	Primary tumor	Private in primary tumor	5:156908936	ADAM19	ADAM metallopeptidase domain 19	ENST00000517374	ENSP00000431027	427	R/W	Cgg/Tgg	-	0.03	0.997	NA	0.727	medium_impact	1.592	high_impact	NA	NA
9	Primary tumor	Private in primary tumor	4:100052734	ADH4	alcohol dehydrogenase 4 (class II), pi polypeptide	ENST00000505590	ENSP00000425416	274	P/L	cCg/cTg	-	0	0.999	NA	1.55	medium_impact	2.679	high_impact	NA	NA
9	Primary tumor	Private in primary tumor	1:27878560	AHDC1	AT hook, DNA binding motif, containing 1	ENST00000247087	ENSP00000247087	23	R/G	Cgg/Ggg	-	0	0.861	0.345	2.064	high_impact	0.718	medium_impact	-0.662	low_impact
9	Primary tumor	Common in primary and metastatic	1:26663362	AIM1L	absent in melanoma 1-like	ENST00000429942	ENSP00000409834	187	S/N	aGc/aAc	rs10751735	0.3	0.999	NA	-0.419	low_impact	1.879	high_impact	NA	NA

		tumors										9								
9	Liver metastases	Common in primary and metastatic tumors	1:26663362	AIM1L	absent in melanoma 1-like	ENST0000429942	ENSP0000409834	187	S/N	aGc/aAc	rs10751735	0.39	0.999	NA	-0.419	low_impact	1.879	high_impact	NA	NA
9	Primary tumor	Common in primary and metastatic tumors	19:49965286	ALDH16A1	aldehyde dehydrogenase 16 family, member A1	ENST0000433981	ENSP0000398675	137	R/H	cGc/cAc	-	0	0.997	NA	1.974	medium_impact	1.568	high_impact	NA	NA
9	Liver metastases	Common in primary and metastatic tumors	19:49965286	ALDH16A1	aldehyde dehydrogenase 16 family, member A1	ENST0000433981	ENSP0000398675	137	R/H	cGc/cAc	-	0	0.997	NA	1.974	medium_impact	1.568	high_impact	NA	NA
9	Primary tumor	Private in primary tumor	17:6909292	ALOX12	arachidonate 12-lipoxygenase	ENST0000251535	ENSP0000251535	455	L/H	cTc/cAc	-	0	0.719	1.975	2.718	high_impact	0.451	medium_impact	0.418	low_impact
9	Liver metastases	Private in metastatic tumor	11:70034096	ANO1	anoctamin 1, calcium activated chloride channel	ENST0000398543	ENSP0000381551	837	G/R	Ggg/Agg	rs3740722	0	0.002	NA	2.124	high_impact	-1.349	low_impact	NA	NA
9	Primary tumor	Common in primary and metastatic tumors	5:112173473	APC	adenomatous polyposis coli	ENST0000457016	ENSP0000413133	728	N/Y	Aat/Tat	-	0	0.998	2.28	2.134	high_impact	1.737	high_impact	1.278	medium_impact
9	Liver metastases	Common in primary and metastatic tumors	5:112173473	APC	adenomatous polyposis coli	ENST0000257430	ENSP0000257430	728	N/Y	Aat/Tat	-	0	0.998	2.28	2.134	high_impact	1.737	high_impact	1.278	medium_impact
9	Primary tumor	Private in primary tumor	22:36123134	APOL5	apolipoprotein L, 5	ENST0000249044	ENSP0000249044	340	L/Q	cTg/cAg	-	0	1	2.32	2.157	high_impact	2.201	high_impact	1.835	medium_impact

9	Liver metastases	Private in metastatic tumor	2:220085471	ATG9A	ATG9 autophagy related 9 homolog A (S. cerevisiae)	ENST00000396761	ENSP00000379983	838	K/E	Aag/Gag	-	0	0.832	0.975	2.105	high_impact	0.777	medium_impact	-0.449	low_impact
9	Liver metastases	Private in metastatic tumor	X:76939771	ATRX	alpha thalassemia/mental retardation syndrome X-linked	ENST00000400862	ENSP00000383659	282	G/A	gGa/gCa	-	0.003	0.993	NA	0.708	medium_impact	2.172	high_impact	NA	NA
9	Primary tumor	Common in primary and metastatic tumors	17:47246064	B4GALNT2	beta-1,4-N-acetyl-galactosaminyl transferase 2	ENST00000300404	ENSP00000300404	433	N/Y	Aat/Tat	-	1	0.998	2.135	-3.114	low_impact	1.665	high_impact	1.106	medium_impact
9	Liver metastases	Common in primary and metastatic tumors	17:47246064	B4GALNT2	beta-1,4-N-acetyl-galactosaminyl transferase 2	ENST00000504681	ENSP00000425510	347	N/Y	Aat/Tat	-	1	0.998	NA	-3.114	low_impact	1.665	high_impact	NA	NA
9	Primary tumor	Private in primary tumor	1:169346168	BLZF1	basic leucine zipper nuclear factor 1	ENST00000367808	ENSP00000356782	140	L/S	tTa/tCa	-	0	0.999	2.36	2.066	high_impact	1.96	high_impact	1.46	medium_impact
9	Primary tumor	Private in primary tumor	10:124921806	BUB3	budding uninhibited by benzimidazoles 3 homolog (yeast)	ENST00000407911	ENSP00000383941	211	S/G	Agc/Ggc	-	0	0.067	2.66	2.062	high_impact	-0.439	low_impact	1.723	medium_impact
9	Primary tumor	Private in primary tumor	10:124921801	BUB3	budding uninhibited by benzimidazoles 3 homolog (yeast)	ENST00000538238	ENSP00000444354	129	D/G	gAc/gGc	-	0	0.291	NA	2.062	high_impact	-0.012	low_impact	NA	NA
9	Primary tumor	Private in primary tumor	17:56386455	BZRAP1	benzodiazapine receptor (peripheral) associated protein 1	ENST00000268893	ENSP00000268893	1333	L/Q	cTg/cAg	-	0	0.996	NA	2.23	high_impact	1.225	medium_impact	NA	NA
9	Liver metastases	Private in metastatic tumor	11:101937378	C11orf70	chromosome 11 open reading frame 70	ENST00000529204	ENSP00000432322	36	R/Q	cGa/cAa	-	0	0	NA	2.929	high_impact	-1.667	low_impact	NA	NA

9	Liver metastases	Private in metastatic tumor	19:2280885	C19orf35	chromosome 19 open reading frame 35	ENST0000342063	ENSP00000345102	16	P/S	Ccc/Tcc	rs55662626	0	0.794	1.79	2.929	high_impact	0.397	medium_impact	0.972	low_impact
9	Liver metastases	Private in metastatic tumor	3:143691196	C3orf58	chromosome 3 open reading frame 58	ENST0000315691	ENSP00000320081	8	K/Q	Aag/Cag	-	0	0	0.805	2.929	high_impact	-1.667	low_impact	-0.106	low_impact
9	Liver metastases	Private in metastatic tumor	6:54054686	C6orf142	chromosome 6 open reading frame 142	ENST0000370876	ENSP00000359913	164	S/T	Tct/Act	rs6934690	0	0.053	NA	2.929	high_impact	-0.661	low_impact	NA	NA
9	Primary tumor	Private in primary tumor	9:140173940	C9orf167	chromosome 9 open reading frame 167	ENST0000357503	ENSP00000350102	267	D/N	Gac/Aac	-	0	0.081	NA	2.011	high_impact	-0.401	low_impact	NA	NA
9	Primary tumor	Private in primary tumor	X:49067085	CACNA1F	calcium channel, voltage-dependent, L type, alpha 1F subunit	ENST0000376265	ENSP00000365441	1495	F/V	Ttt/Gtt	-	0	0.96	2.93	2.318	high_impact	0.926	medium_impact	2.581	medium_impact
9	Primary tumor	Private in primary tumor	9:69256833	CBWD6	COBW domain containing 6	ENST0000377441	ENSP00000366660	100	W/R	Tgg/Cgg	rs113407575	0	0.862	NA	2.015	high_impact	0.649	medium_impact	NA	NA
9	Primary tumor	Private in primary tumor	7:92979250	CCDC132	coiled-coil domain containing 132	ENST0000305866	ENSP00000307666	790	D/H	Gat/Cat	-	0	0.99	1.09	2.929	high_impact	1.209	medium_impact	0.206	low_impact
9	Primary tumor	Common in primary and metastatic tumors	10:61552692	CCDC6	coiled-coil domain containing 6	ENST0000263102	ENSP00000263102	470	P/T	Ccc/Acc	rs1053266	0	0	1.39	2.111	high_impact	-1.545	low_impact	0.331	low_impact
9	Liver metastases	Common in primary and metastatic tumors	10:61552692	CCDC6	coiled-coil domain containing 6	ENST0000263102	ENSP00000263102	470	P/T	Ccc/Acc	rs1053266	0	0	1.39	2.111	high_impact	-1.545	low_impact	0.331	low_impact
9	Prima	Common in	5:10254	CCT	chaperonin containing TCP1, subunit 5	ENST0	ENSP00	75	A/	Gcc/	-	0	0.9	4.57	2.124	high_im	1.9	high_impa	3.065	high_impact

	ry tumor	primary and metastatic tumors	842	5	(epsilon)	0000280326	000280326		T	Acc			99			pact		ct		
9	Liver metastases	Common in primary and metastatic tumors	5:10254842	CCT5	chaperonin containing TCP1, subunit 5 (epsilon)	ENST00000503026	ENSP0000423318	54	A/T	Gcc/Acc	-	0	0.985	NA	2.124	high_impact	1.223	medium_impact	NA	NA
9	Liver metastases	Private in metastatic tumor	19:8367709	CD320	CD320 molecule	ENST00000537716	ENSP0000437697	178	G/R	Gga/Aga	rs2336573	0	0.979	NA	2.1	high_impact	1.132	medium_impact	NA	NA
9	Primary tumor	Common in primary and metastatic tumors	10:73434888	CDH23	cadherin-related 23	ENST00000398860	ENSP0000381836	495	G/A	gGc/gCc	rs1227049	0.02	0.997	NA	0.875	medium_impact	1.573	high_impact	NA	NA
9	Liver metastases	Common in primary and metastatic tumors	10:73434888	CDH23	cadherin-related 23	ENST00000299366	ENSP0000299366	495	G/A	gGc/gCc	rs1227049	0	0.999	NA	2.075	high_impact	1.838	high_impact	NA	NA
9	Primary tumor	Private in primary tumor	16:55860205	CES1	carboxylesterase 1	ENST00000422046	ENSP0000390492	87	C/F	tGc/tTc	rs5023782	0	1	NA	1.953	medium_impact	1.574	high_impact	NA	NA
9	Primary tumor	Private in primary tumor	2:27324420	CGREF1	cell growth regulator with EF-hand domain 1	ENST00000312734	ENSP0000324025	227	G/S	Ggc/Agc	-	0	0	NA	2.075	high_impact	-1.491	low_impact	NA	NA
9	Liver metastases	Private in metastatic tumor	11:99786797	CNTN5	contactin 5	ENST00000418526	ENSP0000393229	123	F/I	Ttt/Att	-	0	0.968	NA	2.062	high_impact	1.039	medium_impact	NA	NA
9	Primary tumor	Common in primary and metastatic	8:139890409	COL22A1	collagen, type XXII, alpha 1	ENST00000303045	ENSP0000303153	81	R/H	cGc/cAc	-	0	1	1.52	2.018	high_impact	1.682	high_impact	0.302	low_impact

		tumors																		
9	Liver metastases	Common in primary and metastatic tumors	8:139890409	COL22A1	collagen, type XXII, alpha 1	ENST00000435777	ENSP00000387655	81	R/H	cGc/cAc	-	0	0.999	NA	2.018	high_impact	1.682	high_impact	NA	NA
9	Liver metastases	Private in metastatic tumor	5:177987740	COL23A1	collagen, type XXIII, alpha 1	ENST00000407622	ENSP00000385092	75	G/R	Gga/Aga	rs34594012	0	0	NA	2.062	high_impact	-1.483	low_impact	NA	NA
9	Primary tumor	Private in primary tumor	2:189859541	COL3A1	collagen, type III, alpha 1	ENST00000317840	ENSP00000315243	480	G/V	gGa/gTa	-	0	0	NA	2.263	high_impact	-2.068	low_impact	NA	NA
9	Liver metastases	Private in metastatic tumor	8:68076677	CSPP1	centrosome and spindle pole associated protein 1	ENST00000412460	ENSP00000415782	571	R/H	cGt/cAt	-	0.02	0.999	NA	1.244	medium_impact	1.787	high_impact	NA	NA
9	Liver metastases	Private in metastatic tumor	7:50547559	DDC	dopa decarboxylase (aromatic L-amino acid decarboxylase)	ENST00000357936	ENSP00000350616	316	V/E	gTg/gAg	-	0	1	3.63	1.828	medium_impact	1.697	high_impact	1.576	medium_impact
9	Primary tumor	Common in primary and metastatic tumors	17:76446377	DNAH17	dynein, axonemal, heavy chain 17	ENST00000300671	ENSP00000300671	3662	D/N	Gat/Aat	rs2289752	0.1	0.999	NA	0.13	medium_impact	1.81	high_impact	NA	NA
9	Liver metastases	Common in primary and metastatic tumors	17:76446377	DNAH17	dynein, axonemal, heavy chain 17	ENST00000442057	ENSP00000396166	868	D/N	Gat/Aat	rs2289752	0.1	0.999	NA	0.13	medium_impact	1.81	high_impact	NA	NA
9	Liver metastases	Private in metastatic tumor	11:103049880	DYNC2H1	dynein, cytoplasmic 2, heavy chain 1	ENST00000398093	ENSP00000381167	2089	N/Y	Aat/Tat	-	0	1	NA	1.879	medium_impact	1.685	high_impact	NA	NA
9	Liver	Private in	21:4019	ETS	v-ets erythroblastosis virus E26 oncogene	ENST0	ENSP00	381	F/	tTc/t	-	0	1	1.29	0.681	mediu	1.936	high_impa	0.307	low_impact

	metastases	metastatic tumor	3576	2	homolog 2 (avian)	0000360938	000354194		S	Cc		.03				m_impact		ct		
9	Liver metastases	Private in metastatic tumor	11:92088232	FAT3	FAT tumor suppressor homolog 3 (Drosophila)	ENST0000525166	ENSP0000432586	835	I/K	aTa/aAa	-	0	0.234	NA	2.075	high_impact	-0.112	low_impact	NA	NA
9	Primary tumor	Common in primary and metastatic tumors	4:153249390	FBXW7	F-box and WD repeat domain containing 7	ENST0000534231	ENSP0000435213	224	T/S	aCt/aGt	-	0.01	0.995	NA	1.138	medium_impact	1.556	high_impact	NA	NA
9	Liver metastases	Common in primary and metastatic tumors	4:153249390	FBXW7	F-box and WD repeat domain containing 7	ENST0000534231	ENSP0000435213	224	T/S	aCt/aGt	-	0.01	0.995	NA	1.138	medium_impact	1.556	high_impact	NA	NA
9	Primary tumor	Common in primary and metastatic tumors	19:40366322	FCGBP	Fc fragment of IgG binding protein	ENST0000221347	ENSP0000221347	4638	G/R	Ggg/Agg	-	0.44	1	2.19	-0.604	low_impact	1.894	high_impact	1.23	medium_impact
9	Liver metastases	Common in primary and metastatic tumors	19:40366322	FCGBP	Fc fragment of IgG binding protein	ENST0000221347	ENSP0000221347	4638	G/R	Ggg/Agg	-	0.44	1	2.19	-0.604	low_impact	1.894	high_impact	1.23	medium_impact
9	Primary tumor	Private in primary tumor	4:159754684	FNIP2	folliculin interacting protein 2	ENST0000504715	ENSP0000420841	22	Q/K	Caa/Aaa	-	0	0.722	NA	2.062	high_impact	0.439	medium_impact	NA	NA
9	Primary tumor	Common in primary and metastatic tumors	2:186671831	FSIP2	fibrous sheath interacting protein 2	ENST0000343098	ENSP0000344403	6022	S/F	tCt/tTt	-	0	0.996	0.975	2.929	high_impact	1.44	medium_impact	0.08	low_impact
9	Liver	Common in	2:18667	FSI	fibrous sheath interacting protein 2	ENST0	ENSP00	5933	S/	tCt/tT	-	0	0.9	1.04	2.929	high_im	1.44	medium_i	0.151	low_impact

	metastases	primary and metastatic tumors	1831	P2		0000424728	000401306		F	t			96			pact		mpact		
9	Primary tumor	Common in primary and metastatic tumors	X:19042064	GPR64	G protein-coupled receptor 64	ENST0000356606	ENSP0000349015	144	R/L	cGc/cTc	-	0	0.147	NA	2.048	high_impact	-0.186	low_impact	NA	NA
9	Liver metastases	Common in primary and metastatic tumors	X:19042064	GPR64	G protein-coupled receptor 64	ENST0000397917	ENSP0000381013	81	R/L	cGc/cTc	-	0	0.889	NA	2.048	high_impact	0.762	medium_impact	NA	NA
9	Primary tumor	Common in primary and metastatic tumors	2:144903170	GTDC1	glycosyltransferase-like domain containing 1	ENST0000392871	ENSP0000376610	106	F/I	Ttc/Atc	-	0.37	0.998	NA	-0.453	low_impact	1.724	high_impact	NA	NA
9	Liver metastases	Common in primary and metastatic tumors	2:144903170	GTDC1	glycosyltransferase-like domain containing 1	ENST0000392871	ENSP0000376610	106	F/I	Ttc/Atc	-	0.37	0.998	NA	-0.453	low_impact	1.724	high_impact	NA	NA
9	Liver metastases	Private in metastatic tumor	12:96384250	HAL	histidine ammonia-lyase	ENST0000261208	ENSP0000261208	259	P/R	cCa/cGa	rs121434329	0	0.999	4.3	1.88	medium_impact	1.742	high_impact	2.369	medium_impact
9	Primary tumor	Common in primary and metastatic tumors	22:17589794	IL17RA	interleukin 17 receptor A	ENST0000319363	ENSP0000320936	562	P/Q	cCg/cAg	rs12484684	0.06	0.998	2.36	0.393	medium_impact	1.689	high_impact	1.024	medium_impact
9	Liver metastases	Common in primary and metastatic tumors	22:17589794	IL17RA	interleukin 17 receptor A	ENST0000425985	ENSP0000400078	510	P/Q	cCg/cAg	rs12484684	0.08	0.999	NA	0.277	medium_impact	1.855	high_impact	NA	NA

9	Primary tumor	Private in primary tumor	2:237300679	IQCA1	IQ motif containing with AAA domain 1	ENST00000254653	ENSP00000254653	451	L/F	ttA/ttT	-	0.002	0.998	NA	0.83	medium_impact	1.666	high_impact	NA	NA
9	Primary tumor	Private in primary tumor	3:52830632	ITIH3	inter-alpha (globulin) inhibitor H3	ENST00000398670	ENSP00000381662	84	P/A	Ccc/Gcc	-	0	0.998	NA	2.194	high_impact	1.838	high_impact	NA	NA
9	Primary tumor	Private in primary tumor	7:150647067	KCNH2	potassium voltage-gated channel, subfamily H (eag-related), member 2	ENST00000430723	ENSP00000387657	863	A/P	Gct/Cct	-	0	0.265	NA	2.309	high_impact	0.294	medium_impact	NA	NA
9	Liver metastases	Private in metastatic tumor	1:196397323	KCNT2	potassium channel, subfamily T, member 2	ENST00000535608	ENSP00000445584	120	E/V	gAa/gTa	-	0	0.909	NA	2.293	high_impact	0.663	medium_impact	NA	NA
9	Primary tumor	Private in primary tumor	X:44966780	KDM6A	lysine (K)-specific demethylase 6A	ENST00000543216	ENSP00000443078	1256	E/G	gAa/gGa	-	0	0.033	NA	2.061	high_impact	-0.545	low_impact	NA	NA
9	Primary tumor	Private in primary tumor	X:44966776	KDM6A	lysine (K)-specific demethylase 6A	ENST00000433797	ENSP00000398929	977	C/G	Tgt/Ggt	-	0	0.993	NA	2.061	high_impact	1.496	medium_impact	NA	NA
9	Liver metastases	Private in metastatic tumor	6:33374193	KIFC1	kinesin family member C1	ENST00000428849	ENSP00000393963	586	L/P	cTt/cCt	-	0	0.997	3.62	2.056	high_impact	1.545	high_impact	2.499	medium_impact
9	Primary tumor	Private in primary tumor	12:53242440	KRT78	keratin 78	ENST00000304620	ENSP00000306261	92	L/P	cTg/cCg	rs2013335	0	0.996	3.45	2.018	high_impact	1.348	medium_impact	2.093	medium_impact
9	Primary tumor	Private in primary tumor	10:85981890	LRIT2	leucine-rich repeat, immunoglobulin-like and transmembrane domains 2	ENST00000538192	ENSP00000438264	490	G/V	gGc/gTc	-	0	1	NA	2.062	high_impact	1.894	high_impact	NA	NA
9	Liver metas	Private in metastatic	21:45877078	LRRC3	leucine rich repeat containing 3	ENST000002	ENSP000002915	184	P/L	cCt/cTt	rs78362277	0	0.992	2.045	2.062	high_impact	1.384	medium_impact	1.078	medium_impact

	tases	tumor				91592	92													
9	Primary tumor	Common in primary and metastatic tumors	11:76372237	LRRC32	leucine rich repeat containing 32	ENST00000404995	ENSP00000385766	134	N/Y	Aac/Tac	-	0	1	3.94	2.062	high_impact	1.894	high_impact	3.066	high_impact
9	Liver metastases	Common in primary and metastatic tumors	11:76372237	LRRC32	leucine rich repeat containing 32	ENST00000404995	ENSP00000385766	134	N/Y	Aac/Tac	-	0	1	3.94	2.062	high_impact	1.894	high_impact	3.066	high_impact
9	Liver metastases	Private in metastatic tumor	6:31683157	LY6G6D	lymphocyte antigen 6 complex, locus G6D	ENST00000375824	ENSP00000364984	9	L/V	Ttg/Gtg	rs3749952	0	0.782	NA	2.929	high_impact	0.379	medium_impact	NA	NA
9	Primary tumor	Private in primary tumor	X:51638222	MAGED1	melanoma antigen family D, 1	ENST00000430189	ENSP00000402032	40	P/L	cCa/cTa	-	0	0.975	NA	2.033	high_impact	1.005	medium_impact	NA	NA
9	Primary tumor	Private in primary tumor	6:161533750	MAP3K4	mitogen-activated protein kinase kinase kinase 4	ENST00000366919	ENSP00000355886	1474	R/C	Cgt/Tgt	-	0	1	NA	1.785	medium_impact	2.108	high_impact	NA	NA
9	Primary tumor	Private in primary tumor	5:126770455	MEGF10	multiple EGF-like-domains 10	ENST00000274473	ENSP00000274473	639	H/Q	caC/caG	-	0.13	0.998	1.265	0.083	medium_impact	1.671	high_impact	0.226	low_impact
9	Primary tumor	Private in primary tumor	19:46394071	MYPOP	Myb-related transcription factor, partner of profilin	ENST00000322217	ENSP00000325402	337	P/H	cCc/cAc	-	0	0.594	0.695	2.052	high_impact	0.307	medium_impact	-0.51	low_impact
9	Primary tumor	Private in primary tumor	2:233745908	NGEF	neuronal guanine nucleotide exchange factor	ENST00000373552	ENSP00000362653	538	E/D	gaG/gaC	-	0.02	0.997	NA	0.688	medium_impact	1.594	high_impact	NA	NA
9	Liver	Private in	3:13243	NP	nephronophthisis 3 (adolescent)	ENST0	ENSP00	207	V/I	Gta/	-	0	0.0	1.645	2.011	high_im	-0.706	low_impa	0.471	low_impact

	metastases	metastatic tumor	7889	HP3		0000337331	000338766			Ata			24			pact		ct		
9	Primary tumor	Private in primary tumor	5:176722013	NSD1	nuclear receptor binding SET domain protein 1	ENST0000361032	ENSP0000354310	2445	L/F	ttA/ttT	-	0	0.996	NA	1.43	medium_impact	1.601	high_impact	NA	NA
9	Liver metastases	Private in metastatic tumor	12:29649145	OVCH1	ovochymase 1	ENST0000318184	ENSP0000326708	84	T/A	Aca/Gca	-	0	0.814	3.985	2.061	high_impact	0.519	medium_impact	2.384	medium_impact
9	Primary tumor	Common in primary and metastatic tumors	3:122404034	PARP14	poly (ADP-ribose) polymerase family, member 14	ENST0000398162	ENSP0000381228	64	R/W	Cgg/Tgg	-	0.04	0.995	2.16	0.594	medium_impact	1.569	high_impact	0.962	low_impact
9	Liver metastases	Common in primary and metastatic tumors	3:122404034	PARP14	poly (ADP-ribose) polymerase family, member 14	ENST0000494811	ENSP0000418535	73	R/W	Cgg/Tgg	-	0	0.47	NA	2.061	high_impact	0.236	medium_impact	NA	NA
9	Liver metastases	Private in metastatic tumor	7:82581285	PCLO	piccolo (presynaptic cytomatrix protein)	ENST0000423517	ENSP0000388393	2995	S/F	tCt/tTt	-	NA	0.996	NA	NA	NA	1.832	high_impact	NA	NA
9	Primary tumor	Private in primary tumor	19:9958798	PIN1	peptidylprolyl cis/trans isomerase, NIMA-interacting 1	ENST0000247970	ENSP0000247970	122	L/V	Ctg/Gtg	-	0	0.976	3.67	2.954	high_impact	1.983	high_impact	4.176	high_impact
9	Primary tumor	Private in primary tumor	11:64023045	PLCB3	phospholipase C, beta 3 (phosphatidylinositol-specific)	ENST0000279230	ENSP0000279230	185	R/L	cGg/cTg	-	1	0.993	1.695	-3.329	low_impact	1.59	high_impact	0.709	low_impact
9	Liver metastases	Private in metastatic tumor	12:19406875	PLEKHA5	pleckstrin homology domain containing, family A member 5	ENST0000538714	ENSP0000439673	77	H/Y	Cat/Tat	-	0	0.993	NA	2.192	high_impact	1.534	high_impact	NA	NA
9	Primary	Private in primary tumor	22:22042368	PPIL2	peptidylprolyl isomerase (cyclophilin)-like 2	ENST000003	ENSP000003818	332	G/R	Ggg/Cgg	-	0	1	4.62	1.916	medium_imp	1.788	high_impact	2.315	medium_impact

	tumor					98831	12									act				
9	Liver metastases	Private in metastatic tumor	9:33796758	PRSS3	protease, serine, 3	ENST0000429677	ENSP00000401828	46	I/N	aTc/aAc	-	0	0.996	NA	2.111	high_impact	1.488	medium_impact	NA	NA
9	Liver metastases	Private in metastatic tumor	7:150069812	REPIN1	replication initiator 1	ENST0000425389	ENSP00000388287	494	K/N	aaA/aaC	-	0	0.931	3.38	2.056	high_impact	0.851	medium_impact	2.285	medium_impact
9	Liver metastases	Private in metastatic tumor	14:24808714	RIPK3	receptor-interacting serine-threonine kinase 3	ENST0000216274	ENSP00000216274	37	R/P	cGg/cCg	-	0.1	0.996	2.775	0.323	medium_impact	1.543	high_impact	2.605	medium_impact
9	Primary tumor	Private in primary tumor	16:67681851	RLTPR	RGD motif, leucine rich repeats, tropomodulin domain and proline-rich containing	ENST0000545661	ENSP00000441481	354	E/G	gAg/gGg	-	0	0.602	NA	2.929	high_impact	0.164	medium_impact	NA	NA
9	Primary tumor	Private in primary tumor	1:1223385	SCNN1D	sodium channel, nonvoltage-gated 1, delta	ENST0000379099	ENSP00000368393	197	E/Q	Gag/Cag	rs75809000	0.17	0.996	NA	-0.075	low_impact	1.517	high_impact	NA	NA
9	Primary tumor	Private in primary tumor	16:29897054	SEZ6L2	seizure related 6 homolog (mouse)-like 2	ENST0000537485	ENSP00000439412	365	G/R	Ggg/Cgg	-	0	0.94	NA	2.929	high_impact	0.748	medium_impact	NA	NA
9	Liver metastases	Private in metastatic tumor	X:9863840	SHROOM2	shroom family member 2	ENST0000380913	ENSP00000370299	631	R/L	cGg/cTg	-	0	0.995	2.125	2.068	high_impact	1.459	medium_impact	1.227	medium_impact
9	Primary tumor	Common in primary and metastatic tumors	17:18166122	SMCR7	Smith-Magenis syndrome chromosome region, candidate 7	ENST0000323019	ENSP00000323591	30	L/V	Ctg/Gtg	-	0	0.979	NA	2.062	high_impact	1.145	medium_impact	NA	NA
9	Liver metas	Common in primary and	17:18166122	SMCR7	Smith-Magenis syndrome chromosome region, candidate 7	ENST000003	ENSP000003790	41	L/V	Ctg/Gtg	-	0	0.979	NA	2.062	high_impact	1.145	medium_impact	NA	NA

	tases	metastatic tumors				95706	57													
9	Primary tumor	Private in primary tumor	17:18166114	SMCR7	Smith-Magenis syndrome chromosome region, candidate 7	ENST00000395704	ENSP00000379056	27	N/T	aAt/aCt	-	0	0.584	NA	2.062	high_impact	0.288	medium_impact	NA	NA
9	Liver metastases	Private in metastatic tumor	17:18166114	SMCR7	Smith-Magenis syndrome chromosome region, candidate 7	ENST00000395706	ENSP00000379057	38	N/S	aAt/aGt	-	0	0.979	NA	2.062	high_impact	1.145	medium_impact	NA	NA
9	Primary tumor	Private in primary tumor	17:18166111	SMCR7	Smith-Magenis syndrome chromosome region, candidate 7	ENST00000395706	ENSP00000379057	37	A/D	gCc/gAc	-	0.01	0.995	NA	1.137	medium_impact	1.5	high_impact	NA	NA
9	Primary tumor	Private in primary tumor	3:181430452	SOX2	SRY (sex determining region Y)-box 2	ENST00000431565	ENSP00000439111	102	M/V	Atg/Gtg	-	0	0.852	NA	2.063	high_impact	0.634	medium_impact	NA	NA
9	Primary tumor	Private in primary tumor	3:181430450	SOX2	SRY (sex determining region Y)-box 2	ENST00000325404	ENSP00000323588	101	H/P	cAc/cCc	-	0	0.999	4.145	2.063	high_impact	1.884	high_impact	3.297	high_impact
9	Primary tumor	Private in primary tumor	3:181430447	SOX2	SRY (sex determining region Y)-box 2	ENST00000431565	ENSP00000439111	100	L/R	cTg/cGg	-	0	0.879	NA	2.063	high_impact	0.691	medium_impact	NA	NA
9	Primary tumor	Common in primary and metastatic tumors	6:44310854	SPATS1	spermatogenesis associated, serine-rich 1	ENST00000288390	ENSP00000424400	8	G/R	Gga/Aga	rs10948132	0	0.995	1.79	2.929	high_impact	1.384	medium_impact	0.972	low_impact
9	Liver metastases	Common in primary and metastatic tumors	6:44310854	SPATS1	spermatogenesis associated, serine-rich 1	ENST00000323108	ENSP00000437552	8	G/R	Gga/Aga	rs10948132	0	0.995	NA	2.929	high_impact	1.384	medium_impact	NA	NA
9	Liver	Private in	7:99917	SPD	speedy homolog E3 (Xenopus laevis)	ENST0	ENSP00	94	Y/	Tac/	-	0	0.9	NA	2.929	high_im	1.44	medium_i	NA	NA

	metastases	metastatic tumor	252	YE3		0000437326	000401147		D	Gac			96			pact		mpact		
9	Liver metastases	Private in metastatic tumor	2:220349773	SPEG	SPEG complex locus	ENST00000265327	ENSP0000265327	2530	S/C	Agc/Tgc	-	0	0.887	NA	2.032	high_impact	0.721	medium_impact	NA	NA
9	Primary tumor	Private in primary tumor	1:32279629	SPOCD1	SPOC domain containing 1	ENST00000533231	ENSP0000435851	436	R/W	Cgg/Tgg	rs6669563	0	0.966	NA	2.069	high_impact	1.06	medium_impact	NA	NA
9	Liver metastases	Private in metastatic tumor	7:75890194	SRRM3	serine/arginine repetitive matrix 3	ENST00000326382	ENSP0000325298	183	K/I	aAa/aTa	-	0	0.927	1.7	2.929	high_impact	0.696	medium_impact	0.874	low_impact
9	Primary tumor	Private in primary tumor	X:48207000	SSX3	synovial sarcoma, X breakpoint 3	ENST00000376895	ENSP0000366092	81	R/H	cGt/cAt	-	0	0.034	NA	2.084	high_impact	-0.588	low_impact	NA	NA
9	Primary tumor	Common in primary and metastatic tumors	1:47717377	STIL	SCL/TAL1 interrupting locus	ENST00000337817	ENSP0000337367	1099	N/H	Aat/Cat	-	0.04	0.998	1.39	0.846	medium_impact	1.613	high_impact	0.534	low_impact
9	Liver metastases	Common in primary and metastatic tumors	1:47717377	STIL	SCL/TAL1 interrupting locus	ENST00000337817	ENSP0000337367	1099	N/H	Aat/Cat	-	0.04	0.998	1.39	0.846	medium_impact	1.613	high_impact	0.534	low_impact
9	Liver metastases	Private in metastatic tumor	11:124502114	TBRG1	transforming growth factor beta regulator 1	ENST00000375005	ENSP0000364144	255	P/S	Cca/Tca	rs76570671	0	0.993	NA	2.064	high_impact	1.511	high_impact	NA	NA
9	Primary tumor	Common in primary and metastatic tumors	17:7577139	TP53	tumor protein p53	ENST00000455263	ENSP0000398846	267	R/G	Cgg/Ggg	rs55832599	0	1	3.145	2.265	high_impact	2.147	high_impact	4.415	high_impact
9	Liver	Common in	17:7577	TP5	tumor protein p53	ENST0	ENSP00	256	R/	Cgg/	rs558	0	1	NA	2.265	high_im	2.147	high_impa	NA	NA

	metastases	primary and metastatic tumors	139	3		0000396473	000379735		G	Ggg	32599					pact		ct		
9	Primary tumor	Private in primary tumor	2:1481231	TPO	thyroid peroxidase	ENST00000345913	ENSP0000318820	398	S/T	aGc/aCc	rs2175977	0.02	0.994	NA	0.746	medium_impact	1.632	high_impact	NA	NA
9	Liver metastases	Private in metastatic tumor	6:123833491	TRDN	triadin	ENST00000361029	ENSP0000354307	28	K/N	aaA/aaC	-	0	0	NA	2.031	high_impact	-1.492	low_impact	NA	NA
9	Liver metastases	Private in metastatic tumor	1:117659352	TRIM45	tripartite motif containing 45	ENST00000369461	ENSP0000358473	356	R/Q	cGg/cAg	rs3738413	0.03	0.996	NA	0.675	medium_impact	1.6	high_impact	NA	NA
9	Primary tumor	Private in primary tumor	17:73874071	TRIM47	tripartite motif containing 47	ENST00000254816	ENSP0000254816	187	R/W	Cgg/Tgg	rs4600514	0	0.998	2.24	2.11	high_impact	1.876	high_impact	1.38	medium_impact
9	Liver metastases	Private in metastatic tumor	19:36168781	UPK1A	uroplakin 1A	ENST00000222275	ENSP0000222275	239	A/V	gCc/gTc	-	0.12	0.993	1.525	0.026	medium_impact	1.672	high_impact	0.026	low_impact
9	Liver metastases	Private in metastatic tumor	2:128477234	WDR33	WD repeat domain 33	ENST00000322313	ENSP0000325377	789	G/R	Ggc/Cgc	-	0	0.995	0.55	2.062	high_impact	1.5	high_impact	-0.49	low_impact
9	Liver metastases	Private in metastatic tumor	19:7685441	XAB2	XPA binding protein 2	ENST00000358368	ENSP0000351137	696	D/H	Gac/Cac	-	0.04	0.998	2.425	0.571	medium_impact	1.724	high_impact	1.477	medium_impact
9	Primary	Private in primary tumor	19:52468203	ZNF350	zinc finger protein 350	ENST000002	ENSP000002436	501	R/S	agA/agT	rs2278415	0	0.036	0.695	2.089	high_impact	-0.619	low_impact	-0.272	low_impact

	tumor					43644	44													
9	Primary tumor	Private in primary tumor	X:47307799	ZNF41	zinc finger protein 41	ENST00000397050	ENSP00000380243	467	H/R	cAt/cGt	-	0	0.999	NA	2.066	high_impact	1.96	high_impact	NA	NA
9	Primary tumor	Private in primary tumor	19:19905488	ZNF506	zinc finger protein 506	ENST00000443905	ENSP00000393835	403	G/V	gGc/gTc	-	0	0.999	3.08	2.084	high_impact	1.992	high_impact	1.87	medium_impact
9	Primary tumor	Private in primary tumor	19:58370895	ZNF587	zinc finger protein 587	ENST00000376209	ENSP00000365382	329	R/L	cGt/cTt	-	0	0.683	NA	2.084	high_impact	0.447	medium_impact	NA	NA
9	Primary tumor	Private in primary tumor	19:20807298	ZNF626	zinc finger protein 626	ENST00000453075	ENSP00000390883	386	F/S	tTt/tCt	-	0	0.999	NA	2.056	high_impact	1.933	high_impact	NA	NA
9	Primary tumor	Private in primary tumor	1:26688458	ZNF683	zinc finger protein 683	ENST00000403843	ENSP00000384782	420	H/L	cAc/cTc	-	0	0.998	2.76	2.064	high_impact	1.829	high_impact	1.742	medium_impact
9	Primary tumor	Common in primary and metastatic tumors	16:3139541	ZSCAN10	zinc finger and SCAN domain containing 10	ENST00000538082	ENSP00000440047	510	K/Q	Aag/Cag	-	0	0.915	NA	2.089	high_impact	0.821	medium_impact	NA	NA
9	Liver metastases	Common in primary and metastatic tumors	16:3139541	ZSCAN10	zinc finger and SCAN domain containing 10	ENST00000538082	ENSP00000440047	510	K/Q	Aag/Cag	-	0	0.915	NA	2.089	high_impact	0.821	medium_impact	NA	NA
10	Primary tumor	Private in primary tumor	1:94578549	ABCA4	ATP-binding cassette, sub-family A (ABC1), member 4	ENST00000535735	ENSP00000437682	47	P/R	cCa/cGa	-	0	0	NA	2.189	high_impact	-1.45	low_impact	NA	NA
10	Primary tumor	Common in primary and metastatic	12:6752776	ACRBP	acrosin binding protein	ENST00000414226	ENSP00000402725	303	T/A	Acc/Gcc	rs3741923	0	0.013	NA	2.064	high_impact	-0.877	low_impact	NA	NA

		tumors																		
10	Liver metastases	Common in primary and metastatic tumors	12:6752776	ACRBP	acrosin binding protein	ENST000000414226	ENSP00000402725	303	T/A	Acc/Gcc	rs3741923	0	0.013	NA	2.064	high_impact	-0.877	low_impact	NA	NA
10	Primary tumor	Common in primary and metastatic tumors	12:7457072	ACSM4	acyl-CoA synthetase medium-chain family member 4	ENST000000399422	ENSP00000382349	49	P/S	Cct/Tct	-	0	0.995	1.69	2.665	high_impact	0.678	medium_impact	-0.082	low_impact
10	Liver metastases	Common in primary and metastatic tumors	12:7457072	ACSM4	acyl-CoA synthetase medium-chain family member 4	ENST000000399422	ENSP00000382349	49	P/S	Cct/Tct	-	0	0.995	1.69	2.665	high_impact	0.678	medium_impact	-0.082	low_impact
10	Liver metastases	Private in metastatic tumor	15:79083120	ADAMTS7	ADAM metallopeptidase with thrombospondin type 1 motif, 7	ENST000000456326	ENSP00000396177	307	T/M	aCg/aTg	rs2127898	0	0.805	NA	2.174	high_impact	0.562	medium_impact	NA	NA
10	Liver metastases	Private in metastatic tumor	4:2906707	ADD1	adducin 1 (alpha)	ENST000000398123	ENSP00000381191	460	G/W	Ggg/Tgg	rs4961	0.07	1	NA	0.074	medium_impact	1.839	high_impact	NA	NA
10	Liver metastases	Private in metastatic tumor	6:106967028	AIM1	absent in melanoma 1	ENST000000369066	ENSP00000358062	241	E/K	Gaa/Aaa	-	0	0.979	1.955	2.056	high_impact	1.145	medium_impact	1.04	medium_impact
10	Liver metastases	Private in metastatic tumor	X:55035653	ALAS2	aminolevulinate, delta-, synthase 2	ENST000000335854	ENSP00000337131	538	F/S	tTc/tCc	-	0	0.996	NA	2.575	high_impact	1.643	high_impact	NA	NA
10	Primary tumor	Common in primary and metastatic tumors	3:125865766	ALDH1L1	aldehyde dehydrogenase 1 family, member L1	ENST000000472186	ENSP00000420293	330	V/F	Gtt/Ttt	rs2886059	0	0.083	NA	2.154	high_impact	-0.409	low_impact	NA	NA

10	Liver metastases	Common in primary and metastatic tumors	3:125865766	ALDH1L1	aldehyde dehydrogenase 1 family, member L1	ENST0000472186	ENSP00000420293	330	V/F	Gtt/Ttt	rs2886059	0	0.083	NA	2.154	high_impact	-0.409	low_impact	NA	NA
10	Liver metastases	Private in metastatic tumor	19:36500397	ALKBH6	alkB, alkylation repair homolog 6 (E. coli)	ENST0000252984	ENSP00000252984	171	V/G	gTg/gGg	-	0	0.992	2.325	2.061	high_impact	1.464	medium_impact	0.968	low_impact
10	Liver metastases	Private in metastatic tumor	8:143546253	BAI1	brain-specific angiogenesis inhibitor 1	ENST0000323289	ENSP00000313046	232	G/R	Ggg/Cgg	-	0	0.621	0	2.048	high_impact	0.37	medium_impact	-0.941	low_impact
10	Primary tumor	Private in primary tumor	X:50659488	BMP15	bone morphogenetic protein 15	ENST0000252677	ENSP00000252677	354	R/W	Cgg/Tgg	-	0	0.997	3.02	2.087	high_impact	1.569	high_impact	1.834	medium_impact
10	Liver metastases	Private in metastatic tumor	1:92442695	BRDT	bromodomain, testis-specific	ENST0000402388	ENSP00000384051	238	K/N	aaA/aaC	rs1156281	0.1	0.996	1.04	0.149	medium_impact	1.73	high_impact	0.012	low_impact
10	Liver metastases	Private in metastatic tumor	11:6232189	C11orf42	chromosome 11 open reading frame 42	ENST0000316375	ENSP00000321021	307	G/R	Ggg/Cgg	-	0	0.948	0.695	2.929	high_impact	0.786	medium_impact	-0.227	low_impact
10	Liver metastases	Private in metastatic tumor	18:30969588	C18orf34	chromosome 18 open reading frame 34	ENST0000402325	ENSP00000385234	42	A/T	Gcc/Acc	rs12606658	0	0.123	NA	2.929	high_impact	-0.431	low_impact	NA	NA
10	Liver metastases	Private in metastatic tumor	21:30464866	C21orf7	chromosome 21 open reading frame 7	ENST0000286791	ENSP00000286791	112	I/V	Att/Gtt	rs3746843	0	0	NA	2.062	high_impact	-1.483	low_impact	NA	NA
10	Primary tumor	Common in primary and metastatic tumors	9:98691137	C9orf102	chromosome 9 open reading frame 102	ENST0000437817	ENSP00000416286	403	V/A	gTc/gCc	rs2274654	0	0.967	NA	2.043	high_impact	1.095	medium_impact	NA	NA
10	Liver	Common in	9:98691	C9o	chromosome 9 open reading frame 102	ENST0	ENSP00	274	V/	gTc/	rs227	0	0.9	NA	2.043	high_im	1.095	medium_i	NA	NA

	metastases	primary and metastatic tumors	137	rf102		0000456993	000409751		A	gCc	4654		67			pact		mpact		
10	Liver metastases	Private in metastatic tumor	9:139734225	C9orf86	chromosome 9 open reading frame 86	ENST000000311502	ENSP00000311134	613	K/T	aAg/aCg	-	0	0.995	NA	1.969	medium_impact	1.548	high_impact	NA	NA
10	Primary tumor	Private in primary tumor	1:26524549	CATSPER4	cation channel, sperm associated 4	ENST000000518899	ENSP00000429464	220	L/H	cTc/cAc	-	0	0.997	NA	2.075	high_impact	1.63	high_impact	NA	NA
10	Primary tumor	Private in primary tumor	2:55555433	CCDC88A	coiled-coil domain containing 88A	ENST000000413716	ENSP00000404431	997	K/N	aaA/aaT	-	0.19	0.993	NA	-0.048	low_impact	1.683	high_impact	NA	NA
10	Primary tumor	Private in primary tumor	18:67563208	CD226	CD226 molecule	ENST000000280200	ENSP00000280200	152	C/W	tgT/tgG	-	0	0.999	2.175	2.311	high_impact	2.063	high_impact	1.257	medium_impact
10	Liver metastases	Private in metastatic tumor	17:45221286	CDC27	cell division cycle 27 homolog (S. cerevisiae)	ENST000000527547	ENSP00000437339	378	S/N	aGt/aAt	rs77440865	0	0.999	NA	2.062	high_impact	1.894	high_impact	NA	NA
10	Liver metastases	Private in metastatic tumor	17:45221273	CDC27	cell division cycle 27 homolog (S. cerevisiae)	ENST000000527547	ENSP00000437339	382	F/L	ttT/ttG	rs1141701	0	0.934	NA	2.062	high_impact	0.853	medium_impact	NA	NA
10	Liver metastases	Private in metastatic tumor	X:47086790	CDK16	cyclin-dependent kinase 16	ENST000000462827	ENSP00000430824	20	N/D	Aat/Gat	-	0.47	0.999	NA	-0.963	low_impact	1.522	high_impact	NA	NA
10	Primary tumor	Common in primary and metastatic tumors	12:122826087	CLIP1	CAP-GLY domain containing linker protein 1	ENST000000542885	ENSP00000438729	389	L/S	tTg/tCg	-	0	0.915	NA	2.129	high_impact	0.854	medium_impact	NA	NA

10	Liver metastases	Common in primary and metastatic tumors	12:122826087	CLIP1	CAP-GLY domain containing linker protein 1	ENST00000545889	ENSP00000438743	245	L/S	tTg/tCg	-	0	0.265	NA	2.129	high_impact	0.043	medium_impact	NA	NA
10	Primary tumor	Private in primary tumor	5:79031558	CMYA5	cardiomyopathy associated 5	ENST00000446378	ENSP00000394770	2324	G/S	Ggt/Agt	rs62621912	0	0.995	2.175	2.02	high_impact	1.555	high_impact	1.124	medium_impact
10	Primary tumor	Private in primary tumor	7:146829418	CNTNAP2	contactin associated protein-like 2	ENST00000361727	ENSP00000354778	389	R/W	Cgg/Tgg	-	0.05	0.998	1.79	0.43	medium_impact	1.638	high_impact	0.774	low_impact
10	Liver metastases	Private in metastatic tumor	2:189916180	COL5A2	collagen, type V, alpha 2	ENST00000452536	ENSP00000398603	573	G/R	Ggg/Cgg	-	NA	0.998	NA	NA	NA	1.611	high_impact	NA	NA
10	Primary tumor	Private in primary tumor	8:68421768	CPA6	carboxypeptidase A6	ENST00000297769	ENSP00000297769	25	S/C	tCt/tGt	rs17853192	0	0.976	NA	2.042	high_impact	1.214	medium_impact	NA	NA
10	Liver metastases	Private in metastatic tumor	19:49139083	DBP	D site of albumin promoter (albumin D-box) binding protein	ENST00000222122	ENSP00000222122	102	P/T	Cca/Aca	-	0	0.265	1.915	2.008	high_impact	-0.023	low_impact	0.996	low_impact
10	Liver metastases	Private in metastatic tumor	9:1056959	DMRT2	doublesex and mab-3 related transcription factor 2	ENST00000358146	ENSP00000350865	458	E/Q	Gaa/Caa	rs17641078	0	0.907	NA	2.066	high_impact	0.802	medium_impact	NA	NA
10	Liver metastases	Private in metastatic tumor	11:62375714	EML3	echinoderm microtubule associated protein like 3	ENST00000278845	ENSP00000278845	390	V/M	Gtg/Atg	-	0.04	0.996	NA	0.571	medium_impact	1.554	high_impact	NA	NA
10	Liver metastases	Private in metastatic tumor	11:62373644	EML3	echinoderm microtubule associated protein like 3	ENST00000278845	ENSP00000278845	517	S/C	tCt/tGt	-	0.0	0.999	NA	0.4	medium_impact	1.894	high_impact	NA	NA

												6								
10	Liver metastases	Private in metastatic tumor	13:114502357	FAM70B	family with sequence similarity 70, member B	ENST00000375348	ENSP00000364497	138	G/W	Ggg/Tgg	-	0	0.998	NA	2.929	high_impact	1.613	high_impact	NA	NA
10	Primary tumor	Common in primary and metastatic tumors	17:18653070	FBXW10	F-box and WD repeat domain containing 10	ENST00000308799	ENSP00000310382	236	E/K	Gaa/Aaa	rs9895749	0	0.96	NA	2.062	high_impact	0.982	medium_impact	NA	NA
10	Liver metastases	Common in primary and metastatic tumors	17:18653070	FBXW10	F-box and WD repeat domain containing 10	ENST00000301938	ENSP00000306937	236	E/K	Gaa/Aaa	rs9895749	0	0.889	NA	2.062	high_impact	0.714	medium_impact	NA	NA
10	Primary tumor	Private in primary tumor	17:80696368	FN3K	fructosamine 3 kinase	ENST00000300784	ENSP00000300784	49	R/W	Cgg/Tgg	-	0.01	0.999	2.475	1.095	medium_impact	1.978	high_impact	1.381	medium_impact
10	Liver metastases	Private in metastatic tumor	15:39910404	FSIP1	fibrous sheath interacting protein 1	ENST00000350221	ENSP00000280236	411	L/F	Ctt/Ttt	rs12908846	0	0.952	0.895	2.062	high_impact	0.936	medium_impact	-0.128	low_impact
10	Liver metastases	Private in metastatic tumor	3:180680840	FXR1	fragile X mental retardation, autosomal homolog 1	ENST00000468861	ENSP00000420515	302	G/D	gGc/gAc	-	0.62	0.997	NA	-1.3	low_impact	2.054	high_impact	NA	NA
10	Liver metastases	Private in metastatic tumor	1:193074705	GLRX2	glutaredoxin 2	ENST00000367440	ENSP00000356410	22	A/T	Gca/Aca	-	0	0.832	NA	2.195	high_impact	0.464	medium_impact	NA	NA
10	Primary tumor	Private in primary tumor	19:17691513	GLT25D1	glycosyltransferase 25 domain containing 1	ENST00000379714	ENSP00000369036	195	V/E	gTg/gAg	-	0	0.792	NA	2.04	high_impact	0.563	medium_impact	NA	NA
10	Prima	Private in	6:24450	GPL	glycosylphosphatidylinositol specific	ENST0	ENSP00	469	V/	Gtg/	-	0	0.9	4.185	1.169	mediu	1.573	high_impa	1.911	medium_imp

	ry tumor	primary tumor	058	D1	phospholipase D1	0000230036	000230036		M	Atg		.01	94			m_impact		ct		act
10	Liver metastases	Private in metastatic tumor	5:176025287	GPRIN1	G protein regulated inducer of neurite outgrowth 1	ENST0000335532	ENSP00000335279	517	G/R	Gga/Aga	-	0.04	0.995	NA	0.757	medium_impact	1.649	high_impact	NA	NA
10	Primary tumor	Private in primary tumor	3:51749692	GRM2	glutamate receptor, metabotropic 2	ENST0000395052	ENSP00000378492	635	R/W	Cgg/Tgg	-	0	1	3.065	2.538	high_impact	1.969	high_impact	1.676	medium_impact
10	Primary tumor	Private in primary tumor	10:93185087	HECTD2	HECT domain containing 2	ENST0000298068	ENSP00000298068	63	I/T	aTt/aCt	-	0	0.004	1.59	2.114	high_impact	-1.129	low_impact	0.43	low_impact
10	Primary tumor	Common in primary and metastatic tumors	X:130415208	IGSF1	immunoglobulin superfamily, member 1	ENST0000361420	ENSP00000355010	544	R/W	Cgg/Tgg	-	0	0.998	0.69	2.439	high_impact	1.599	high_impact	-0.472	low_impact
10	Liver metastases	Common in primary and metastatic tumors	X:130415208	IGSF1	immunoglobulin superfamily, member 1	ENST0000370910	ENSP00000359947	535	R/W	Cgg/Tgg	-	0	0.979	NA	2.439	high_impact	0.936	medium_impact	NA	NA
10	Primary tumor	Private in primary tumor	19:39734325	IL28B	interleukin 28B (interferon, lambda 3)	ENST0000413851	ENSP00000409000	180	R/C	Cgc/Tgc	rs77379751	0	1	2.785	2.087	high_impact	1.83	high_impact	1.612	medium_impact
10	Liver metastases	Private in metastatic tumor	14:105173878	INF2	inverted formin, FH2 and WH2 domain containing	ENST0000392634	ENSP00000376410	425	P/L	cCa/cTa	-	0	0	2.18	2.122	high_impact	-1.544	low_impact	0.83	low_impact
10	Liver metastases	Private in metastatic tumor	14:105173881	INF2	inverted formin, FH2 and WH2 domain containing	ENST0000392634	ENSP00000376410	426	P/L	cCc/cTc	-	0	0	1.365	2.122	high_impact	-1.544	low_impact	0.049	low_impact

10	Primary tumor	Private in primary tumor	16:31374535	ITGAX	integrin, alpha X (complement component 3 receptor 4 subunit)	ENST00000268296	ENSP00000268296	517	P/R	cCc/cGc	rs2230429	0	0.997	1.88	1.949	medium_impact	1.593	high_impact	0.622	low_impact
10	Primary tumor	Private in primary tumor	1:111216263	KCNA3	potassium voltage-gated channel, shaker-related subfamily, member 3	ENST00000369769	ENSP00000358784	390	T/M	aCg/aTg	-	0	1	4.075	2.235	high_impact	2.141	high_impact	4.617	high_impact
10	Primary tumor	Private in primary tumor	6:39285685	KCNK16	potassium channel, subfamily K, member 16	ENST00000373229	ENSP00000362326	124	F/L	ttC/ttG	-	0	0.999	1.57	2.511	high_impact	2.082	high_impact	0.695	low_impact
10	Liver metastases	Private in metastatic tumor	8:136594291	KHDRBS3	KH domain containing, RNA binding, signal transduction associated 3	ENST00000355849	ENSP00000348108	261	P/L	cCg/cTg	-	0	0.013	1.7	2.267	high_impact	-0.526	low_impact	0.481	low_impact
10	Primary tumor	Private in primary tumor	16:85667696	KIAA0182	KIAA0182	ENST00000253458	ENSP00000253458	62	A/T	Gcc/Acc	-	0	0	0.805	2.062	high_impact	-1.483	low_impact	-0.223	low_impact
10	Primary tumor	Common in primary and metastatic tumors	13:42385446	KIAA0564	KIAA0564	ENST00000379310	ENSP00000368612	660	R/G	Aga/Gga	rs9562353	0	1	2.885	2.101	high_impact	1.834	high_impact	1.483	medium_impact
10	Liver metastases	Common in primary and metastatic tumors	13:42385446	KIAA0564	KIAA0564	ENST00000379310	ENSP00000368612	660	R/G	Aga/Gga	rs9562353	0	1	2.885	2.101	high_impact	1.834	high_impact	1.483	medium_impact
10	Primary tumor	Common in primary and metastatic tumors	9:34256634	KIF24	kinesin family member 24	ENST00000443226	ENSP00000414628	37	H/Y	Cac/Tac	rs76974239	0	0	NA	2.056	high_impact	-1.51	low_impact	NA	NA
10	Liver metastases	Common in primary and metastatic	9:34256634	KIF24	kinesin family member 24	ENST00000443226	ENSP00000414628	37	H/Y	Cac/Tac	rs76974239	0	0	NA	2.056	high_impact	-1.51	low_impact	NA	NA

		tumors																		
10	Primary tumor	Private in primary tumor	X:24024195	KLHL15	kelch-like 15 (Drosophila)	ENST00000328046	ENSP0000332791	206	R/W	Cgg/Tgg	-	0	0.999	1.665	2.062	high_impact	1.894	high_impact	0.679	low_impact
10	Primary tumor	Private in primary tumor	21:15524921	LIPI	lipase, member I	ENST00000344577	ENSP0000343331	385	G/E	gGa/gAa	rs74369337	0	0.979	NA	2.044	high_impact	1.148	medium_impact	NA	NA
10	Primary tumor	Common in primary and metastatic tumors	19:39805868	LRFN1	leucine rich repeat and fibronectin type III domain containing 1	ENST00000248668	ENSP0000248668	37	R/C	Cgc/Tgc	-	0.14	1	2.015	0.026	medium_impact	1.894	high_impact	1.046	medium_impact
10	Liver metastases	Common in primary and metastatic tumors	19:39805868	LRFN1	leucine rich repeat and fibronectin type III domain containing 1	ENST00000248668	ENSP0000248668	37	R/C	Cgc/Tgc	-	0.14	1	2.015	0.026	medium_impact	1.894	high_impact	1.046	medium_impact
10	Primary tumor	Private in primary tumor	3:169514585	LRRC34	leucine rich repeat containing 34	ENST00000528597	ENSP0000436883	35	L/I	Tta/Ata	rs10936600	0	0.958	NA	2.064	high_impact	0.973	medium_impact	NA	NA
10	Primary tumor	Private in primary tumor	16:79633594	MAF	v-maf musculoaponeurotic fibrosarcoma oncogene homolog (avian)	ENST00000326043	ENSP0000327048	69	P/L	cCt/cTt	-	0	0.99	NA	2.077	high_impact	1.447	medium_impact	NA	NA
10	Primary tumor	Private in primary tumor	6:90424429	MDN1	MDN1, midasin homolog (yeast)	ENST00000369393	ENSP0000358400	2301	R/Q	cGa/cAa	-	0.02	1	2.705	0.857	medium_impact	1.9	high_impact	1.36	medium_impact
10	Liver metastases	Private in metastatic tumor	10:129902158	MKI67	antigen identified by monoclonal antibody Ki-67	ENST00000368654	ENSP0000357643	2649	R/H	cGt/cAt	rs12777740	0.29	0.996	0.69	-0.343	low_impact	1.566	high_impact	-0.356	low_impact
10	Prima	Private in	16:5553	MM	matrix metallopeptidase 2 (gelatinase A,	ENST0	ENSP00	560	G/	Gga/	-	0	0.9	3.4	0.727	mediu	1.687	high_impa	2.235	medium_imp

	ry tumor	primary tumor	2269	P2	72kDa gelatinase, 72kDa type IV collagenase)	0000219070	000219070		R	Aga		.03	98			m_impact		ct		act
10	Primary tumor	Private in primary tumor	6:36940552	MTCH1	mitochondrial carrier homolog 1 (C. elegans)	ENST00000373565	ENSP00000362666	91	L/P	cTg/cCg	-	0	0.995	NA	2.062	high_impact	1.5	high_impact	NA	NA
10	Liver metastases	Private in metastatic tumor	11:1017183	MUC6	mucin 6, oligomeric mucus/gel-forming	ENST00000421673	ENSP00000406861	1873	P/Q	cCa/cAa	rs34844844	0.001	0.996	NA	1.137	medium_impact	1.554	high_impact	NA	NA
10	Primary tumor	Private in primary tumor	11:65632281	MUS81	MUS81 endonuclease homolog (S. cerevisiae)	ENST00000308110	ENSP00000307853	423	Y/H	Tac/Cac	-	0	0.963	2.74	2.219	high_impact	0.802	medium_impact	2.037	medium_impact
10	Primary tumor	Common in primary and metastatic tumors	10:95083039	MYOF	myoferlin	ENST00000371501	ENSP00000360556	1783	R/Q	cGg/cAg	rs11594445	0	0.999	NA	2.161	high_impact	2.029	high_impact	NA	NA
10	Liver metastases	Common in primary and metastatic tumors	10:95083039	MYOF	myoferlin	ENST00000359263	ENSP00000352208	1783	R/Q	cGg/cAg	rs11594445	0	0.988	2.885	2.161	high_impact	1.407	medium_impact	1.75	medium_impact
10	Primary tumor	Common in primary and metastatic tumors	11:64855986	NA	NA	ENST00000524603	ENSP00000435002	98	F/L	Ttc/Ctc	rs2282498	0	0.053	NA	2.929	high_impact	-0.661	low_impact	NA	NA
10	Liver metastases	Common in primary and metastatic tumors	11:64855986	NA	NA	ENST00000530719	ENSP00000432459	98	F/L	Ttc/Ctc	rs2282498	0	0.053	NA	2.929	high_impact	-0.661	low_impact	NA	NA
10	Prima	Private in	3:17534	NAA	N-acetylated alpha-linked acidic	ENST0	ENSP00	622	P/	cCc/	rs986	0	0.9	1.04	2.12	high_im	1.868	high_impa	-0.028	low_impact

	ry tumor	primary tumor	5143	LADL2	dipeptidase-like 2	0000454872	000404705		R	cGc	6564		99			pact		ct		
10	Primary tumor	Private in primary tumor	12:78582428	NAV3	neuron navigator 3	ENST0000228327	ENSP0000228327	1954	L/V	Ctg/Gtg	-	0	0.859	NA	2.011	high_impact	0.612	medium_impact	NA	NA
10	Primary tumor	Private in primary tumor	1:148009349	NBPF14	neuroblastoma breakpoint family, member 14	ENST0000310701	ENSP0000309907	659	M/T	aTg/aCg	-	0	0	NA	2.929	high_impact	-1.667	low_impact	NA	NA
10	Primary tumor	Private in primary tumor	16:57060353	NLRC5	NLR family, CARD domain containing 5	ENST0000538110	ENSP0000441597	7	C/R	Tgc/Cgc	rs28438857	0	0	NA	2.011	high_impact	-1.472	low_impact	NA	NA
10	Primary tumor	Private in primary tumor	4:56502307	NMU	neuromedin U	ENST0000505262	ENSP0000424246	18	A/E	gCg/gAg	rs3828555	0	0.438	NA	2.026	high_impact	0.096	medium_impact	NA	NA
10	Primary tumor	Common in primary and metastatic tumors	11:5475506	OR51I2	olfactory receptor, family 51, subfamily I, member 2	ENST0000341449	ENSP0000341987	263	R/H	cGc/cAc	rs11037502	0.01	0.999	3	0.874	medium_impact	1.724	high_impact	0.969	low_impact
10	Liver metastases	Common in primary and metastatic tumors	11:5475506	OR51I2	olfactory receptor, family 51, subfamily I, member 2	ENST0000341449	ENSP0000341987	263	R/H	cGc/cAc	rs11037502	0.01	0.999	3	0.874	medium_impact	1.724	high_impact	0.969	low_impact
10	Primary tumor	Private in primary tumor	11:6129837	OR56B4	olfactory receptor, family 56, subfamily B, member 4	ENST0000316529	ENSP0000321196	277	P/S	Cct/Tct	rs1462983	0.65	0.999	1.155	-0.92	low_impact	1.724	high_impact	-0.247	low_impact
10	Liver metastases	Private in metastatic tumor	12:58111945	OS9	osteosarcoma amplified 9, endoplasmic reticulum lectin	ENST0000413095	ENSP0000413112	178	G/D	gGc/gAc	-	0.07	0.992	NA	0.328	medium_impact	1.537	high_impact	NA	NA

10	Primary tumor	Common in primary and metastatic tumors	12:80747242	OTOGL	otogelin-like	ENST00000458043	ENSP00000400895	1840	I/V	Att/Gtt	rs7297767	0.88	0.595	NA	260.269	high_impact	497.979	high_impact	NA	NA
10	Liver metastases	Common in primary and metastatic tumors	12:80747242	OTOGL	otogelin-like	ENST00000298820	ENSP00000298820	283	I/V	Att/Gtt	rs7297767	0.71	0.998	NA	369.974	high_impact	1080.773	high_impact	NA	NA
10	Liver metastases	Private in metastatic tumor	X:91873516	PCDH11X	protocadherin 11 X-linked	ENST00000356934	ENSP00000349408	1207	L/F	ttG/ttC	-	0	0.975	NA	2.075	high_impact	1.056	medium_impact	NA	NA
10	Primary tumor	Common in primary and metastatic tumors	5:140573986	PCDHB10	protocadherin beta 10	ENST00000239446	ENSP00000239446	621	N/D	Aat/Gat	-	0	0.988	1.95	2.075	high_impact	1.236	medium_impact	0.918	low_impact
10	Liver metastases	Common in primary and metastatic tumors	5:140573986	PCDHB10	protocadherin beta 10	ENST00000239446	ENSP00000239446	621	N/D	Aat/Gat	-	0	0.988	1.95	2.075	high_impact	1.236	medium_impact	0.918	low_impact
10	Primary tumor	Private in primary tumor	5:140595625	PCDHB13	protocadherin beta 13	ENST00000430318	ENSP00000416253	644	V/I	Gtc/Atc	rs2910005	0	0.927	NA	2.075	high_impact	0.786	medium_impact	NA	NA
10	Primary tumor	Common in primary and metastatic tumors	7:44104839	PGAM2	phosphoglycerate mutase 2 (muscle)	ENST00000297283	ENSP00000297283	97	G/D	gGc/gAc	rs77938727	0	0.999	3.455	4.098	high_impact	1.377	medium_impact	1.081	medium_impact
10	Liver metastases	Common in primary and metastatic tumors	7:44104839	PGAM2	phosphoglycerate mutase 2 (muscle)	ENST00000297283	ENSP00000297283	97	G/D	gGc/gAc	rs77938727	0	0.999	3.455	4.098	high_impact	1.377	medium_impact	1.081	medium_impact

10	Primary tumor	Common in primary and metastatic tumors	7:47968927	PKD1L1	polycystic kidney disease 1 like 1	ENST00000289672	ENSP00000289672	312	V/F	Gtt/Ttt	rs2686817	0	0.988	0.805	2.064	high_impact	1.291	medium_impact	-0.196	low_impact
10	Liver metastases	Common in primary and metastatic tumors	7:47968927	PKD1L1	polycystic kidney disease 1 like 1	ENST00000289672	ENSP00000289672	312	V/F	Gtt/Ttt	rs2686817	0	0.988	0.805	2.064	high_impact	1.291	medium_impact	-0.196	low_impact
10	Primary tumor	Common in primary and metastatic tumors	2:198950240	PLCL1	phospholipase C-like 1	ENST00000437704	ENSP00000414138	569	V/I	Gta/Ata	rs1064213	0	0.994	NA	1.95	medium_impact	1.629	high_impact	NA	NA
10	Liver metastases	Common in primary and metastatic tumors	2:198950240	PLCL1	phospholipase C-like 1	ENST00000437704	ENSP00000414138	569	V/I	Gta/Ata	rs1064213	0	0.994	NA	1.95	medium_impact	1.629	high_impact	NA	NA
10	Primary tumor	Common in primary and metastatic tumors	7:102212939	POLR2J3	polymerase (RNA) II (DNA directed) polypeptide J3	ENST00000508848	ENSP00000425877	10	F/L	ttC/ttG	-	0.01	0.999	NA	1.292	medium_impact	2.203	high_impact	NA	NA
10	Liver metastases	Common in primary and metastatic tumors	7:102212939	POLR2J3	polymerase (RNA) II (DNA directed) polypeptide J3	ENST00000379340	ENSP00000368645	10	F/L	ttC/ttG	-	0.01	0.999	2.39	1.292	medium_impact	2.203	high_impact	1.314	medium_impact
10	Primary tumor	Common in primary and metastatic tumors	6:106553435	PRDM1	PR domain containing 1, with ZNF domain	ENST00000369089	ENSP00000358085	333	P/L	cCg/cTg	rs77256382	0	0.992	NA	2.066	high_impact	1.438	medium_impact	NA	NA
10	Liver metas	Common in primary and	6:106553435	PRDM	PR domain containing 1, with ZNF domain	ENST000003	ENSP000003580	333	P/L	cCg/cTg	rs77256382	0	0.992	NA	2.066	high_impact	1.438	medium_impact	NA	NA

	tases	metastatic tumors		1		69089	85													
10	Primary tumor	Common in primary and metastatic tumors	4:152201053	PRSS48	protease, serine, 48	ENST00000530477	ENSP00000431197	36	C/Y	tGt/tAt	rs36097019	0	0.999	NA	2.061	high_impact	1.856	high_impact	NA	NA
10	Liver metastases	Common in primary and metastatic tumors	4:152201053	PRSS48	protease, serine, 48	ENST00000530477	ENSP00000431197	36	C/Y	tGt/tAt	rs36097019	0	0.999	NA	2.061	high_impact	1.856	high_impact	NA	NA
10	Primary tumor	Private in primary tumor	10:89692905	PTEN	phosphatase and tensin homolog	ENST00000371953	ENSP00000361021	130	R/Q	cGa/cAa	rs121909229	0	1	3.9	2.594	high_impact	1.867	high_impact	7.959	high_impact
10	Liver metastases	Private in metastatic tumor	10:23482635	PTF1A	pancreas specific transcription factor, 1a	ENST00000376504	ENSP00000365687	263	S/P	Tcc/Ccc	rs7918487	0	0	1.78	2.056	high_impact	-1.54	low_impact	0.705	low_impact
10	Primary tumor	Common in primary and metastatic tumors	11:36597313	RAG1	recombination activating gene 1	ENST00000534663	ENSP00000434610	820	K/R	aAg/aGg	rs2227973	0	0.301	NA	2.114	high_impact	0.126	medium_impact	NA	NA
10	Liver metastases	Common in primary and metastatic tumors	11:36597313	RAG1	recombination activating gene 1	ENST00000299440	ENSP00000299440	820	K/R	aAg/aGg	rs2227973	0	0.025	1.81	2.114	high_impact	-0.631	low_impact	0.657	low_impact
10	Primary tumor	Private in primary tumor	X:114426553	RBMXL3	RNA binding motif protein, X-linked-like 3	ENST00000424776	ENSP00000417451	850	D/G	gAc/gGc	-	0	0.632	0	2.056	high_impact	0.343	medium_impact	-1.129	low_impact
10	Primary tumor	Private in primary tumor	15:59347930	RNF111	ring finger protein 111	ENST00000434298	ENSP00000393641	353	S/C	Agt/Tgt	-	0	0.95	0.69	2.11	high_impact	1.044	medium_impact	-0.328	low_impact

10	Primary tumor	Common in primary and metastatic tumors	6:45390626	RUNX2	runt-related transcription factor 2	ENST00000371438	ENSP00000360493	119	P/S	Ccc/Tcc	-	0	0.999	3.165	2.076	high_impact	2.38	high_impact	2.772	medium_impact
10	Liver metastases	Common in primary and metastatic tumors	6:45390626	RUNX2	runt-related transcription factor 2	ENST00000371432	ENSP00000360486	105	P/S	Ccc/Tcc	-	0	0.998	NA	2.076	high_impact	2.188	high_impact	NA	NA
10	Primary tumor	Private in primary tumor	15:33954413	RYR3	ryanodine receptor 3	ENST00000415757	ENSP00000399610	1561	T/M	aCg/aTg	-	NA	1	NA	NA	NA	1.721	high_impact	NA	NA
10	Primary tumor	Private in primary tumor	8:19218745	SH2D4A	SH2 domain containing 4A	ENST00000523736	ENSP00000428048	168	E/G	gAa/gGa	rs35647122	0	0.664	NA	2.062	high_impact	0.372	medium_impact	NA	NA
10	Liver metastases	Private in metastatic tumor	15:48527152	SLC12A1	solute carrier family 12 (sodium/potassium/chloride transporters), member 1	ENST00000428362	ENSP00000410367	202	P/L	cCa/cTa	-	0	0.998	NA	2.219	high_impact	1.513	high_impact	NA	NA
10	Primary tumor	Private in primary tumor	17:78220006	SLC26A11	solute carrier family 26, member 11	ENST00000546047	ENSP00000440724	384	T/M	aCg/aTg	-	0	1	NA	1.828	medium_impact	1.702	high_impact	NA	NA
10	Primary tumor	Common in primary and metastatic tumors	7:150767637	SLC4A2	solute carrier family 4, anion exchanger, member 2 (erythrocyte membrane protein band 3-like 1)	ENST00000485713	ENSP00000419412	515	E/K	Gag/Aag	-	0	0.997	3.775	1.963	medium_impact	1.601	high_impact	2.434	medium_impact
10	Liver metastases	Common in primary and metastatic tumors	7:150767637	SLC4A2	solute carrier family 4, anion exchanger, member 2 (erythrocyte membrane protein band 3-like 1)	ENST00000413384	ENSP00000405600	515	E/K	Gag/Aag	-	0	0.997	3.775	1.963	medium_impact	1.601	high_impact	2.434	medium_impact
10	Primary	Common in primary and	12:20905250	SLCO1C	solute carrier organic anion transporter family, member 1C1	ENST00005	ENSP000004445	559	S/F	tCt/tTt	rs6487138	0	0.235	NA	2.306	high_impact	0.047	medium_impact	NA	NA

	tumor	metastatic tumors		1		45102	27													
10	Liver metastases	Common in primary and metastatic tumors	12:20905250	SLCO1C1	solute carrier organic anion transporter family, member 1C1	ENST0000545604	ENSP00000444149	677	S/F	tCt/tTt	rs6487138	0	0.235	NA	2.306	high_impact	0.047	medium_impact	NA	NA
10	Primary tumor	Private in primary tumor	2:46986987	SOCS5	suppressor of cytokine signaling 5	ENST0000306503	ENSP00000305133	440	D/N	Gac/Aac	-	0	0.996	2.56	2.057	high_impact	1.326	medium_impact	1.683	medium_impact
10	Primary tumor	Common in primary and metastatic tumors	12:23793778	SOX5	SRY (sex determining region Y)-box 5	ENST0000541536	ENSP00000441973	316	A/T	Gca/Aca	-	0.05	0.997	NA	0.434	medium_impact	1.621	high_impact	NA	NA
10	Liver metastases	Common in primary and metastatic tumors	12:23793778	SOX5	SRY (sex determining region Y)-box 5	ENST0000537393	ENSP00000439832	294	A/T	Gca/Aca	-	0.02	0.998	NA	0.823	medium_impact	1.72	high_impact	NA	NA
10	Liver metastases	Private in metastatic tumor	2:32288989	SPAST	spastin	ENST0000315285	ENSP00000320885	30	A/D	gCc/gAc	-	0	0	0	2.243	high_impact	-1.127	low_impact	-0.973	low_impact
10	Primary tumor	Common in primary and metastatic tumors	1:48764419	SPATA6	spermatogenesis associated 6	ENST0000396199	ENSP00000379502	406	C/Y	tGt/tAt	rs1056042	0	0.948	NA	2.929	high_impact	0.786	medium_impact	NA	NA
10	Liver metastases	Common in primary and metastatic tumors	1:48764419	SPATA6	spermatogenesis associated 6	ENST0000396199	ENSP00000379502	406	C/Y	tGt/tAt	rs1056042	0	0.948	NA	2.929	high_impact	0.786	medium_impact	NA	NA
10	Primary	Common in primary and	20:44352620	SPINT4	serine peptidase inhibitor, Kunitz type 4	ENST000002	ENSP000002790	73	G/S	Ggc/Agc	rs6017667	0	0.999	3.515	2.079	high_impact	1.922	high_impact	2.285	medium_impact

	tumor	metastatic tumors				79058	58													
10	Liver metastases	Common in primary and metastatic tumors	20:44352620	SPINT4	serine peptidase inhibitor, Kunitz type 4	ENST00000279058	ENSP00000279058	73	G/S	Ggc/Agc	rs6017667	0	0.999	3.515	2.079	high_impact	1.922	high_impact	2.285	medium_impact
10	Liver metastases	Private in metastatic tumor	X:48054740	SSX5	synovial sarcoma, X breakpoint 5	ENST00000311798	ENSP00000312415	19	E/Q	Gag/Cag	rs4824675	0	0.552	NA	2.084	high_impact	0.307	medium_impact	NA	NA
10	Primary tumor	Common in primary and metastatic tumors	19:19121086	SUGP2	SURP and G patch domain containing 2	ENST00000337018	ENSP00000337926	639	R/Q	cGg/cAg	rs116953419	0.02	0.999	0.975	0.843	medium_impact	1.904	high_impact	-0.12	low_impact
10	Liver metastases	Common in primary and metastatic tumors	19:19121086	SUGP2	SURP and G patch domain containing 2	ENST00000452918	ENSP00000389380	639	R/Q	cGg/cAg	rs116953419	0.02	0.999	0.975	0.843	medium_impact	1.904	high_impact	-0.12	low_impact
10	Primary tumor	Common in primary and metastatic tumors	11:1858262	SYT8	synaptotagmin VIII	ENST00000341958	ENSP00000343691	289	T/M	aCg/aTg	rs484955	0	1	NA	2.062	high_impact	1.894	high_impact	NA	NA
10	Liver metastases	Common in primary and metastatic tumors	11:1858262	SYT8	synaptotagmin VIII	ENST00000381978	ENSP00000371406	302	T/M	aCg/aTg	rs484955	0	0.658	NA	2.062	high_impact	0.365	medium_impact	NA	NA
10	Primary tumor	Private in primary tumor	6:159463270	TAGAP	T-cell activation RhoGTPase activating protein	ENST00000367066	ENSP00000356033	52	K/M	aAg/aTg	-	0.01	0.999	1.1	1.174	medium_impact	1.963	high_impact	0.096	low_impact
10	Prima	Common in	1:12687	TAS	taste receptor, type 1, member 3	ENST0	ENSP00	514	F/	ttC/tt	-	0	0.9	1.565	0.02	mediu	1.652	high_impa	0.308	low_impact

	ry tumor	primary and metastatic tumors	01	1R3		0000339381	000344411		L	G		.12	99			m_impact		ct		
10	Liver metastases	Common in primary and metastatic tumors	1:1268701	TAS1R3	taste receptor, type 1, member 3	ENST00000339381	ENSP00000344411	514	F/L	ttC/ttG	-	0.12	0.999	1.565	0.02	medium_impact	1.652	high_impact	0.308	low_impact
10	Primary tumor	Common in primary and metastatic tumors	1:36550658	TEKT2	tektin 2 (testicular)	ENST00000469024	ENSP00000434183	46	R/C	Cgc/Tgc	rs12043423	0	0.999	NA	2.929	high_impact	1.787	high_impact	NA	NA
10	Liver metastases	Common in primary and metastatic tumors	1:36550658	TEKT2	tektin 2 (testicular)	ENST00000469024	ENSP00000434183	46	R/C	Cgc/Tgc	rs12043423	0	0.999	NA	2.929	high_impact	1.787	high_impact	NA	NA
10	Liver metastases	Private in metastatic tumor	1:92161307	TGFBR3	transforming growth factor, beta receptor III	ENST00000465892	ENSP00000432638	786	V/M	Gtg/Atg	-	0	0.998	NA	2.318	high_impact	1.468	medium_impact	NA	NA
10	Liver metastases	Private in metastatic tumor	6:32064372	TNXB	tenascin XB	ENST00000375247	ENSP00000364396	420	V/M	Gtg/Atg	-	0	0.974	NA	2.031	high_impact	1.021	medium_impact	NA	NA
10	Primary tumor	Common in primary and metastatic tumors	14:21500121	TPPP2	tubulin polymerization-promoting protein family member 2	ENST00000530140	ENSP00000435356	133	R/L	cGc/cTc	rs9624	0	0.999	NA	2.929	high_impact	1.787	high_impact	NA	NA
10	Liver metastases	Common in primary and metastatic tumors	14:21500121	TPPP2	tubulin polymerization-promoting protein family member 2	ENST00000321760	ENSP00000317595	133	R/L	cGc/cTc	rs9624	0	0.999	3.455	2.929	high_impact	1.787	high_impact	2.795	medium_impact
10	Prima	Private in	3:36872	TRA	tetratricopeptide repeat and ankyrin repeat	ENST0	ENSP00	2716	K/	Aaa/	-	0	0.9	NA	2.175	high_im	1.282	medium_i	NA	NA

	ry tumor	primary tumor	796	NK1	containing 1	0000429976	000416168		Q	Caa			89			pact		mpact		
10	Primary tumor	Common in primary and metastatic tumors	8:141461267	TRAPPC9	trafficking protein particle complex 9	ENST00000389328	ENSP00000373979	167	G/A	gGt/gCt	-	0	0.999	NA	2.929	high_impact	1.787	high_impact	NA	NA
10	Liver metastases	Common in primary and metastatic tumors	8:141461267	TRAPPC9	trafficking protein particle complex 9	ENST00000389327	ENSP00000373978	69	G/A	gGt/gCt	-	0	0.998	NA	2.929	high_impact	1.613	high_impact	NA	NA
10	Liver metastases	Private in metastatic tumor	5:180661359	TRIM41	tripartite motif containing 41	ENST00000438174	ENSP00000394459	178	G/S	Ggc/Agc	-	0.02	0.996	NA	0.764	medium_impact	1.573	high_impact	NA	NA
10	Liver metastases	Private in metastatic tumor	3:32859625	TRIM71	tripartite motif containing 71	ENST00000383763	ENSP00000373272	18	M/R	aTg/aGg	-	0	0	0.55	2.069	high_impact	-1.491	low_impact	-0.477	low_impact
10	Primary tumor	Private in primary tumor	21:45846591	TRPM2	transient receptor potential cation channel, subfamily M, member 2	ENST00000540347	ENSP00000439161	60	D/N	Gac/Aac	-	0.38	0.955	NA	-0.231	low_impact	1.713	high_impact	NA	NA
10	Primary tumor	Private in primary tumor	15:50903364	TRPM7	transient receptor potential cation channel, subfamily M, member 7	ENST00000313478	ENSP00000320239	736	H/D	Cac/Gac	-	0	0.976	3.22	2.197	high_impact	0.982	medium_impact	3.408	high_impact
10	Primary tumor	Private in primary tumor	5:134223804	TXNDC15	thioredoxin domain containing 15	ENST00000508810	ENSP00000424428	158	V/L	Gtg/Ctg	rs79262456	0	0.688	NA	2.929	high_impact	0.258	medium_impact	NA	NA
10	Primary tumor	Private in primary tumor	15:62316035	VPS13C	vacuolar protein sorting 13 homolog C (S. cerevisiae)	ENST00000261517	ENSP00000261517	153	R/H	cGt/cAt	rs12595158	0	0.993	0.69	2.929	high_impact	1.299	medium_impact	-0.232	low_impact

10	Liver metastases	Private in metastatic tumor	7:73085699	VPS37D	vacuolar protein sorting 37 homolog D (S. cerevisiae)	ENST00000451519	ENSP00000413337	165	H/P	cAc/cCc	-	0	0.302	NA	2.929	high_impact	-0.149	low_impact	NA	NA
10	Liver metastases	Private in metastatic tumor	3:184542548	VPS8	vacuolar protein sorting 8 homolog (S. cerevisiae)	ENST00000424721	ENSP00000397553	43	K/R	aAg/aGg	rs117998598	0	0.254	NA	2.117	high_impact	-0.057	low_impact	NA	NA
10	Liver metastases	Private in metastatic tumor	12:118511726	VSIG10	V-set and immunoglobulin domain containing 10	ENST00000359236	ENSP00000352172	333	V/M	Gtg/Atg	rs9668527	0.1	0.996	2.81	0.178	medium_impact	1.554	high_impact	1.88	medium_impact
10	Primary tumor	Private in primary tumor	19:7685441	XAB2	XPA binding protein 2	ENST00000534844	ENSP00000438225	693	D/H	Gac/Cac	-	0.04	0.998	NA	0.571	medium_impact	1.724	high_impact	NA	NA
10	Primary tumor	Common in primary and metastatic tumors	14:23994804	ZFHX2	zinc finger homeobox 2	ENST00000419474	ENSP00000413418	1449	F/L	ttT/ttG	-	0	0	2.11	2.056	high_impact	-1.54	low_impact	1.031	medium_impact
10	Liver metastases	Common in primary and metastatic tumors	14:23994804	ZFHX2	zinc finger homeobox 2	ENST00000419474	ENSP00000413418	1449	F/L	ttT/ttG	-	0	0	2.11	2.056	high_impact	-1.54	low_impact	1.031	medium_impact
10	Primary tumor	Common in primary and metastatic tumors	19:53612720	ZNF415	zinc finger protein 415	ENST00000448501	ENSP00000396492	241	Y/C	tAt/tGt	rs1560099	1	0.995	-1.345	-3.414	low_impact	1.586	high_impact	-2.233	low_impact
10	Liver metastases	Common in primary and metastatic tumors	19:53612720	ZNF415	zinc finger protein 415	ENST00000440291	ENSP00000414601	180	Y/C	tAt/tGt	rs1560099	1	0.998	NA	-3.414	low_impact	1.818	high_impact	NA	NA
10	Prima	Private in	20:6259	ZNF	zinc finger protein 512B	ENST0	ENSP00	651	S/	tCg/t	-	0	0.8	1.7	2.064	high_im	0.783	medium_i	0.687	low_impact

	ry tumor	primary tumor	4464	512B		0000450537	000393795		L	Tg			89			pact		mpact		
10	Primary tumor	Private in primary tumor	3:75787620	ZNF717	zinc finger protein 717	ENST0000478296	ENSP0000419377	335	H/R	cAt/cGt	rs75737034	0	0.536	NA	2.084	high_impact	0.29	medium_impact	NA	NA
10	Primary tumor	Common in primary and metastatic tumors	9:99580834	ZNF782	zinc finger protein 782	ENST0000481138	ENSP0000419397	491	S/A	Tca/Gca	rs75989782	0	0.647	0.87	2.084	high_impact	0.407	medium_impact	-0.179	low_impact
10	Liver metastases	Common in primary and metastatic tumors	9:99580834	ZNF782	zinc finger protein 782	ENST0000481138	ENSP0000419397	491	S/A	Tca/Gca	rs75989782	0	0.647	0.87	2.084	high_impact	0.407	medium_impact	-0.179	low_impact
10	Primary tumor	Private in primary tumor	19:58549511	ZSCAN1	zinc finger and SCAN domain containing 1	ENST0000391700	ENSP0000375581	103	P/S	Ccc/Tcc	-	0	0.978	NA	2.066	high_impact	1.181	medium_impact	NA	NA

Table S3. Summary of searching results for metastases related genes in GeneRIF database

Gene symbol	Gene ID	Pubmed ID	GeneRIF text
ACTN4	81	15493875	Actinin alpha-4 is mainly present intracellularly and is involved in cellular motility via interaction with the actin cytoskeleton, thus probably affecting the metastatic potential of tumor cells.
ACTN4	81	18362906	Motility-related ACTN4 is associated with advanced and metastatic ovarian carcinoma.
ACTN4	81	18765526	Actinin-4 contributes to the invasive growth of pancreatic ductal carcinoma, and ACTN4 is one of the candidate oncogenes in this chromosome locus.
ACTN4	81	19913389	expression of alpha-actinin-4 was significantly associated with invasion potential defined by the Matrigel invasion assay. Cancer cell lines with higher alpha-actinin-4 expression had greater invasive potential.
ACTN4	81	23810014	Data suggest that ACTN4 plays roles in breast cancer tumorigenesis via promotion of cell proliferation, metastasis, and drug resistance.
ACTN4	81	23817592	Increased expression of ACTN4 is associated with invasiveness of bladder cancer.
ALOX12	239	14669797	our data suggest that an increase in 12-LOX expression enhances the metastatic potential of human prostate cancer cells.
ALOX12	239	22237009	12(S)-LOX expression in inflammatory areas of colorectal tumours has the capacity to induce an invasive phenotype in colorectal cancer cells and could be targeted for therapy.
APC	324	11921277	Three APC mutations have been identified in 22 tested samples, all of them in xenografts developed from metastatic prostate tumors.
APC	324	12800196	APC inactivation due to mutations, LOH and methylation is associated with invasive and in situ lobular breast cancer.
APC	324	15832580	The APC mutations associated wiht desmoid tumors and responsible for the behavior of the invasiveness of these neoplasms.
APC	324	17653897	A nonsense mutation in Arg1114X in the APC gene was found in 5 of 43 colorectal cancer patients. The patients also had higher rates of metastasis.
APC	324	18844223	adenomatous polyposis coli gene and nuclear b-catenin may have roles in wnt-related progression of invasive urothelial carcinomas
APC	324	21302450	Higher rates of the expressions of both APC and c-Myc proteins in non-small cell lung cancer primary foci were found compared with those in lymph node metastases.
APC	324	23302090	Membrane protrusions with APC/beta-catenin-containing puncta control the migratory potential and mesenchymal morphology of mammary tumor cells.
APC	324	23393221	CDX2 activated APC and AXIN2 promoter activities via intestinal cell-specific enhancer elements; results suggest that a low CDX2 level has influence on the Wnt signaling in invasive colon cancer cells possibly promoting cellular migration
APC	324	24197976	The methylated pattern of APC promoter 1A was detected in 43.8% of gastric adenocarcinoma cases and correlated with T-stage (P = 0.046) and distant metastasis
PRDM1	639	19433448	the induction of Blimp1 represents a novel mechanism whereby the RelB NF-kappaB subunit mediates repression, specifically of ERalpha, thereby promoting a more migratory phenotype in breast cancer cells
PRDM1	639	22438909	Blimp1 is a mediator of Ras/Raf/AP-1 signaling that promotes cell migration, and is repressed by LOX-PP in lung cancer.
BRCA1	672	15009718	BRCA1 downregulation in melanoma cells did not make them more aggressive and could lead to new therapeutic strategies for this tumor, which is so difficult to control once metastasized.

BRCA1	672	16011248	nm23H1 protein could effectively restrain gastric cancer metastasis and development; and BRCA1 protein could restain tumor from becoming lower differentiation.
BRCA1	672	16944270	Ninteen percent of the women who developed both invasive breast and ovarian tumors carried one of the analyzed BRCA1 gene mutations but none of the women were positive for the analyzed BRCA2 mutation.
BRCA1	672	17952592	The site of first distant metastasis is different between BRCA1- and BRCA2-associated and sporadic breast cancer patients.
BRCA1	672	19012002	Studied BRCA1 and BRCA2 exp'n in young breast cancer patients;BRCA1 & BRCA2 expression correlated in healthy, but not in tumor tissues. Neither BRCA1 nor BRCA2 exp'n was assoc'd with tumor histology, diff'n, nodal metastasis or p53 and HER-2 exp'n.
BRCA1	672	19098453	study demonstrates that BRCA1 controls cell motility and invasion through its regulation of several key genes which are crucial in the progression of breast cancer
BRCA1	672	19644562	BRCA1 methylation correlated with age at diagnosis (P = .015) and 5-years disease free survival (P = .016) while hMLH1 methylation was more frequent in larger tumors (P = .002) and in presence of distant metastasis (P = .004).
BRCA1	672	19885732	In this prospective study of women who were unaffected at the time of genetic testing and who were negative for the known familial mutation in BRCA1/2, no excess risk of invasive breast cancer was observed.
BRCA1	672	20038817	loss of BRCA1 function may contribute to the aggressiveness of Ras-MAPK driven breast cancer with associated increase in levels of cyclin D1 and c-myc, enhanced MAPK activity, angiogenic potential & invasiveness
BRCA1	672	20134318	The prophylactic procedure is highly effective in preventing invasive breast cancer in BRCA1 mutation carriers
BRCA1	672	20384635	natural mutant allele of BRCA1 (Mut.BRCA1) can elicit some of the changes involved in metastatic progression in human breast cancer via the overexpression of osteopontin.
BRCA1	672	20398395	Lympho-vascular invasion is frequent in BRCA1 germline mutation related breast cancers, but seems to occur as often in sporadic controls matched for age, grade and tumor type.
BRCA1	672	20406939	Data extend the ovarian BRCAness phenotype, imply BRCA1/2-deficient ovarian cancer is biologically distinct, and suggest that patients with visceral metastases should be considered for BRCA1/2 sequencing.
BRCA1	672	21080930	study found no difference in the prevalence of loss of wt BRCA1 between ER+ and ER- invasive BRCA1-associated breast cancers
BRCA1	672	21324516	BRCA1 and BRCA2 mutations are common in women with invasive ovarian cancer. All women diagnosed with invasive non-mucinous ovarian cancer should be considered to be candidates for genetic testing.
BRCA1	672	22274685	Among patients with invasive epithelial ovarian cancer, having a germline mutation in BRCA1 or BRCA2 was associated with improved 5-year overall survival. BRCA2 carriers had the best prognosis.
BRCA1	672	22431556	BRCA1/p220 loss of expression or function triggers BRCA1-IRIS overexpression through a post-transcriptional mechanism, which in turn promotes formation of aggressive and invasive breast tumors.
BRCA1	672	22710074	Invasive tubal carcinoma and TIC were limited to BRCA-mutation carriers, whereas hyperplasia and minor epithelial atypia were commonly found in both BRCA-mutation carriers and controls.
BRCA1	672	22931600	Decreased BRCA1 expression can enhance progesterone-stimulated tumor cell proliferation and migration in sporadic breast cancer.

BRCA1	672	23082475	Sixty-two patients (index cases) with invasive breast cancer belonging to sixty families and their asymptomatic female first-degree relatives (300 cases) were studied for germline mutations of BRCA1 and BRCA2 genes.
BRCA1	672	23257159	for women with invasive ovarian cancer, the short-term survival advantage of carrying a BRCA1 or BRCA2 mutation does not lead to a long-term survival benefit
BRCA1	672	23569316	BRCA1/2 germ-line mutations confer a more aggressive prostate cancer phenotype with a higher probability of nodal involvement, distant metastasis and poor survival.
BRCA1	672	23860775	Data indicate that patients negative for BRCA1 protein were more often with pathological tumor-node-metastasis stage III, positive for lymph node metastasis and MYC overexpression than BRCA1-positive tumors.
BRAF	673	12644542	results demonstrate that the mutational status of BRAF and KRAS is distinctly different among histologic types of ovarian serous carcinoma, occurring most frequently in invasive micropapillary serous carcinomas and its precursors, serous borderline tumors
BRAF	673	15998781	Role of BRAF mutation in facilitating metastasis and progression of papillary thyroid cancer in lymph nodes.
BRAF	673	16959844	BRAFV600E activates not only MAPK but also NF-kappaB signaling pathway in human thyroid cancer cells, leading to an acquisition of apoptotic resistance and promotion of invasion.
BRAF	673	17387744	BRAF(V600E) mutation is identified in a subset of cutaneous metastases from papillary thyroid carcinomas
BRAF	673	18628356	The frequencies of LOHs of 17q21, 17p13, 10q23, and 22q13 were higher in tumors with lymph node metastasis, suggesting that these LOHs may be important in increased lymph node metastasis.
BRAF	673	19001320	Wild-type BRAF is required for response to panitumumab or cetuximab in metastatic colorectal cancer.
BRAF	673	19014278	In Korean patients with papillary thyroid carcinoma, the BRAFV600E mutation is associated with a lower frequency of background Hashimoto thyroiditis and a high frequency of lymph node metastasis.
BRAF	673	19079609	Genetic extinction of BRAF(V600E) in established prostate tumors did not lead to tumor regression, indicating that while sufficient to initiate development of invasive prostate adenocarcinoma, BRAF(V600E) is not required for its maintenance.
BRAF	673	19282104	study of KRAS/BRAF mutation status in a large and well-documented cohort of primary and metastatic renal cell carcinoma
BRAF	673	19355825	BRAF(V600E) is associated some of the aggressive clinicopathological features of papillary thyroid carcinoma including younger age at diagnosis, larger tumor size, and classic histological type, as well as also extrathyroidal invasion.
BRAF	673	19571295	study concludes that a BRAF mutation is a negative prognostic marker in patients with metastatic colorectal cancer
BRAF	673	19584155	Results suggest that the mutations of EGFR, KRAS, BRAF between primary tumors and corresponding lymph node metastases should be considered whenever mutations are used for the selection of patients for EGFR-directed tyrosine kinase inhibitor therapy.
BRAF	673	19603018	Assessing KRAS and BRAF mutations might help optimising the selection of the candidate metastatic colorectal cancer patients to receive anti-EGFR moAbs.
BRAF	673	19603024	BRAF (P=0.01) mutations predicted reduced progress free survival in response to cetuximab salvage therapy in patients with metastatic colorectal cancer .
BRAF	673	19850689	BRAF(T1799A) can be detected in the blood of papillary thyroid carcinoma patients with residual or metastatic disease and may provide diagnostic information
BRAF	673	20049644	BRAF mutations only in metastases is not associated with resistance to anti-EGFR treatment in primary colorectal tumors.

BRAF	673	20498063	Knockdown of B-Raf(V600E) resulted in thrombospondin-1 down-regulation and a reduction of adhesion and migration/invasion of human thyroid cancer cells.
BRAF	673	20619739	if KRAS is not mutated, assessing BRAF, NRAS, and PIK3CA exon 20 mutations (in that order) gives additional information about the efficacy of cetuximab plus chemotherapy in metastatic colorectal cancer.
BRAF	673	20631031	No significant difference in BRAF alterations was found between pT1 tumors and thyroid capsule invasion and pT3 tumors.
BRAF	673	20645028	samples of metastatic colorectal cancer were tested for the presence of the seven most common mutations in the KRAS gene and the V600E mutation in the BRAF gene
BRAF	673	20857202	BRAF V600E mutation is associated with lack of response in wild-type KRAS metastatic colorectal cancer treated with anti-EGFR monoclonal antibodies.
BRAF	673	21289333	803 metastatic colorectal cancer samples studied for KRAS exon 2 and BRAF exon 15 mutations; BRAF mutated samples were characterized for mismatch repair function; 344 tumours were mutated -34 involving BRAF mutations (8 of microsatellite instability type)
BRAF	673	21456008	the impact of BRAF mutation and microsatellite instability on prognosis in metastatic colorectal cancer
BRAF	673	21693616	Eight of 16 primary tumor samples and 4 of 6 metastatic samples showed BRAF V600E gene mutations and no copy number changes were associated exclusively with metastatic cancer
BRAF	673	21750338	BRAF mutation of papillary thyroid carcinoma may have differential predictive values for LN metastasis, according to tumor size.
BRAF	673	21796448	Importance of infiltrative growth pattern and invasiveness over presence of BRAF mutation in classic and follicular variant papillary thyroid carcinoma for development of nodal metastases.
BRAF	673	21943101	BRAF mutation V600E significantly induces cell migration and invasion properties in vitro in colon cancer cells
BRAF	673	21948220	In malignant FNABs in papillary thyroid carcinoma, BRAF(V600E) mutation was significantly associated with presence of extra-thyroidal extension and metastases after surgery.
BRAF	673	22249628	None of the molecular marker mutations that were analyzed in this study, including the BRAF mutation, predicted lymph node metastasis in classic papillary thyroid carcinoma.
BRAF	673	22435913	analysis of a patient with pancreatic metastasis arising from a BRAF(V600E)-positive papillary thyroid cancer [case report]
BRAF	673	22681706	BRAFV600E mutation is associated with lymph node metastasis in multiple papillary thyroid carcinoma.
BRAF	673	22740704	These results clearly prove that the BRAFV600E mutation is not associated with the development of distant metastases or fatal outcome in papillary thyroid carcinoma
BRAF	673	22870241	High-throughput genotyping in metastatic esophageal squamous cell carcinoma identifies phosphoinositide-3-kinase and BRAF mutations.
BRAF	673	22941165	The BRAF V600E mutation is the only independent predictor of compartment lymph node metastasis in papillary thyroid carcinoma.
BRAF	673	23055546	BRAF mutation was associated with lymph node metastases (LNM), advanced stage, extrathyroidal extension, tumor size, male gender, multifocality, absence of capsule, classic PTC, and tall-cell variant papillary thyroid cancer.
BRAF	673	23062653	BRAF mutation was not found to be significantly associated with lymph node metastasis in patients with papillary thyroid cancer
BRAF	673	23098991	Studied differential miRNA expression in metastatic colorectal cancer by microarrays from primary tumors of 33 patients who had wild-type KRAS/BRAF and third- to

			sixth-line anti-EGFRmAb treatment, with/without irinotecan.
BRAF	673	23132792	BRAFV600E mutation is associated with cervical lymph node metastasis and recurrence in papillary thyroid cancer.
BRAF	673	23242808	Data indicate that BRAF and EGF receptor or SRC family kinase inhibition blocked proliferation and invasion of the resistant tumors.
BRAF	673	23401445	The role of BRAF V600E as targetable driver mutation in lung adenocarcinoma is strengthened by finding that in vivo expression of V600E in mice leads to development of invasive adenocarcinoma, a phenotype that is reversed when V600E expression is stopped.
BRAF	673	23435375	BRAF(V600E) increases migration and invasion of thyroid cancer cells via upregulation of Snail with a concomitant decrease of its target E-cadherin.
BRAF	673	23533235	genetic association studies in population in Italy: Data suggest that V600E mutation in BRAF in subjects with papillary thyroid carcinomas with/without lymph node metastases is not associated with disease progression.
BRAF	673	23569304	Antitumor effects of vemurafenib are mediated by inhibiting oncogenic MAPK signaling in BRAF(V600)-mutant metastatic melanoma. Data suggest that inhibition downstream of BRAF should help to overcome acquired resistance.
BRAF	673	23613396	K-RAS and B-RAF mutations do not seem to be predictive of treatment outcome as potential biomarkers for bevacizumab therapy in metastatic colorectal cancer.
BRAF	673	23615046	BRAF mutation is associated with poor response to anti-EGFR MoAbs and it is an adverse prognostic biomarker of the survival of patients with metastatic colorectal cancer.
BRAF	673	23636013	Braf mutations were not associated with the risk of lymph node metastasis in papillary thyroid carcinoma.
BRAF	673	23981603	BRAF(V600E) mutation is predictive for distant metastasis in papillary thyroid carcinoma but not positively.
BRAF	673	24030686	There was no significant association between BRAF positivity and tumor multicentricity, lymphovascular invasion, extranodal extension, central neck involvement, advanced stage (stage III or IV), and distant metastasis.
BRAF	673	24123003	HSP60 protein expression may inhibit lymph node metastasis in papillary thyroid carcinomas harboring the BRAF mutation.
BRAF	673	24335665	Provided there is adequate quantity of viable tumor cells, immunohistochemical testing any melanoma sample from a patient with metastatic disease will accurately determine BRAF status.
BRAF	673	24367680	BRAF V600E-mutation in metastatic colorectal cancer characterizes a subgroup of patients with distinct biologic, clinical and pathological features and is associated with very poor patients' prognosis.
BRAF	673	24559116	BRAF mutation was significantly associated with a larger tumor size, extrathyroidal invasion and lymph node metastasis in papillary thyroid cancer.
CA2	760	22416960	A significant correlation was found between positive carbonic anhydrase II staining and oral squamous cell carcinoma for more advanced clinical stage and larger tumor size, but not for positive lymph node metastasis, distal metastasis, and recurrence.
CA9	768	15069539	significantly higher rate of strong CA IX expression in non-invasive cancers influences survival data
CA9	768	17390110	study confirms that the expression level of CA9 gene in conventional renal cell carcinoma is related to metastasis
CA9	768	18695901	Low expression of CAIX and high expression of VEGF is associated with metastasis in clear cell renal cell carcinoma.
CA9	768	18976937	COX-2 and carbonic anhydrase IX seem to be important predictors of outcome in patients with metastatic renal cell carcinomas
CA9	768	19539328	CA9 single nucleotide polymorphisms are common in patients with metastatic clear cell renal cell carcinoma

CA9	768	19619339	Carbonic anhydrase 9 may have a role in survival with a lower dose of bevacizumab in patients with previously treated metastatic colorectal cancer
CA9	768	20358226	CAIX is overexpressed in the majority of vulvar carcinomas with relationships to advanced tumor stages and development of lymph node metastases.
CA9	768	21223596	Data show that CAIX is associated with advanced tumor stages and lymph node metastases in cervical cancer, potentially representing a new target in this disease.
CA9	768	21363891	CA9 modulates tumor-associated cell migration and invasion.
CA9	768	21745383	Elevated serum levels of the invasion markers TIMP-1 and CAIX in metastatic breast cancer are prognostic markers and are associated with the presence of circulating tumor cells
CA9	768	21870331	both MCT1 and CD147, but not MCT4, were associated with GLUT1 and CAIX expression in a large series of invasive breast carcinoma samples
CA9	768	22289741	Pharmacologic interference of CAIX catalytic activity using monoclonal antibodies or CAIX-specific small molecule inhibitors, consequently disrupting pH regulation by cancer cells, has been shown recently to impair primary tumor growth and metastasis.
CA9	768	23802595	Carbon anhydrase IX specific immune responses in patients with metastatic renal cell carcinoma potentially cured by interleukin-2 based immunotherapy.
CA9	768	24926090	CAIX can be a useful ancillary marker for identifying mesothelial cells. There is no difference in CAIX expression between benign and malignant mesothelium. Caution should be exercised while evaluating for metastasis from renal cell carcinoma.
CANX	821	22065046	The data suggest that patients with low or defective TAP1 or calnexin in primary breast cancers may be at higher risks for developing brain metastasis due to the defects in T cell-based immunosurveillance.
RUNX2	860	15665096	Fidelity of Runx2 intranuclear organization is necessary for expression of target genes that mediate the osteolytic activity of metastatic breast cancer cells.
RUNX2	860	16166639	Runx2-regulated MMP9 levels are functionally related to the invasion properties of cancer cells
RUNX2	860	18829534	A novel role of Runx2 in up-regulating the vicious cycle of metastatic bone disease, in addition to Runx2 regulation of genes related to progression of tumor metastasis.
RUNX2	860	19915614	Runx2 is a key regulator of events associated with prostate cancer metastatic bone disease.
RUNX2	860	21649908	LGD1069 may impair angiogenic and metastatic potential induced by tumor cells through suppressing expression of Runx2 directly on human endothelial cells.
RUNX2	860	21885439	novel evidence that inhibition of BMP-2 or BMP-2-mediated MAPK/Runx2/Snail signaling is an attractive therapeutic target for osteolytic bone metastases in lung cancer patients.
RUNX2	860	22032690	Runx2 and CBFbeta are required for the expression of genes that mediate the ability of metastatic breast cancer cells to directly modulate both osteoclast and osteoblast function.
RUNX2	860	22396198	RUNX2 is a potent prognostic factor in human colon carcinoma patients through the promotion of cell proliferation and invasion properties, and is at least partly upregulated by estrogen signals through ERbeta of carcinoma cells.
RUNX2	860	22641097	Studied the potential molecular mechanism of thyroid cancer invasion.Data suggest enhanced Runx2 is functionally linked to tumor invasion and metastasis of thyroid carcinoma by regulating EMT-related molecules, MMPs and angiogenic/lymphangiogenic factors.
RUNX2	860	22821892	We showed that Id1 controls the expression of the Runx2 isoform I and that this transcription factor plays a central role in mediating the Id1 proinvasive function in thyroid tumor cells.

RUNX2	860	23389849	Runx2 is a novel and important downstream mediator of the phosphatidylinositol 3-kinase (PI3K)/Akt pathway that is linked to metastatic properties of breast cancer cells.
RUNX2	860	23933319	TSSC1 inhibits breast cancer cell invasion. Subsequently, TSSC1 is confirmed as a target of Runx2 and is negatively regulated by Runx2.
RUNX2	860	24069422	CDH6 is under the control of the transcription factor RUNX2, which we previously described as a crucial mediator of the Id1 pro-invasive function in thyroid tumor cells.
CD1D	912	21695190	Breast cancer cells, through downregulation of CD1d and subsequent evasion of natural killer T-mediated antitumor immunity, gain increased potential for metastatic tumor progression.
CD34	947	16415795	CD34 immunostaining provides some insights into the histogenesis of invasive and noninvasive implants in serous borderline tumors of the ovary.
CD34	947	16557578	bone marrow stem cells contribute to the growing tumor vasculature in Ewing's sarcoma and that vascular endothelial growth factor-165 is critical for the migration of CD34+ cells from the bone marrow into the tumor
CD34	947	17656039	Loss of CD34+ fibrocytes is not a consistent feature of invasive lobular carcinomas of the breast. A preserved CD34+ stromal cell population does not exclude malignancy.
CD34	947	18025805	VEGF, HIF-1alpha and CD34 expressions were more common in gastric tumors without serosal invasion
CD34	947	23803010	Data suggest that circulating CD34/VEGFR3 are biomarkers for epithelial ovarian cancer (EOC); circulating bone marrow-derived lymphatic/vascular endothelial progenitor cells are significantly increased in EOC and correlate with lymph node metastasis.
COL1A1	1277	15805113	prostate carcinoma cell proliferation is enhanced by the down-regulation of BRCA2 expression when interacting with COL1, a major component of the ECM at osseous metastatic sites
COL1A1	1277	16835341	Increased C-telopeptide of collagen type I is associated with skeletal invasion of breast cancer
COL1A1	1277	17140440	MT1-MMP is a major mediator of tumor cell invasiveness and type I collagen degradation by VHL RCC cells that express either MT1-MMP or HIF-2alpha
COL1A1	1277	17889845	Combined use of osteocalcin and beta-CTX could be useful in early detection of bone metastatic breast cancer which might improve the outcome of the disease.
COL1A1	1277	20181574	Elevated C-terminal type I collagen telopeptide is associated with with bone metastases from castration-resistant prostate cancer.
COL1A1	1277	20359090	The expression of Col I, Col IV and Fn was closely related to tumor invasion, the regional lymph node metastasis and other pathological features in laryngeal squamous cell carcinoma.
COL1A1	1277	22694915	basal lamina type IV collagen alpha chains, matrix metalloproteinases-2 and -9 expressions are expressed in oral dysplasia and invasive carcinoma
COL1A1	1277	23013730	High Fibrillar type I collagen matrices enhance metastasis/invasion of ovarian epithelial cancer via beta1 integrin and PTEN signals
COL1A1	1277	23276824	High serum and urine levels of cross-linked N-telopeptide of type I collagen is associated with bone metastasis in lung cancer.
COL4A6	1288	15211113	Collagen chains alpha5(IV) and alpha6(IV) were frequently absent in basement membrane from pancreatic adenocarcinoma, and their absence might be related to the invasion of cancer cells.
COL4A6	1288	20359090	The expression of Col I, Col IV and Fn was closely related to tumor invasion, the regional lymph node metastasis and other pathological features in laryngeal squamous cell carcinoma.

COL17A1	1308	16487966	Together, these data demonstrate a novel interaction between collagen XVII and alphaIIb integrin and also suggest a possibility to use tirofiban to inhibit the invasion and progression of alphaIIb expressing SCC tumors.
CSPG4	1464	15210734	MCSP may facilitate primary melanoma progression by enhancing the activation of key signaling pathways important for tumor invasion and growth.
CSPG4	1464	21658254	Cell surface P-selectin binding depends on CHST11 gene expression. CSPG4 serves as a P-selectin ligand through its CS chain and participates in P-selectin binding to the highly metastatic breast cancer cells.
DMD	1756	24793134	dystrophin is a tumor suppressor and likely anti-metastatic factor in myogenic tumors that progress to high-grade, lethal sarcomas
DNM2	1785	20574164	results suggest dynamin 2 might be involved in preventing tumor invasion and lymph node metastasis, possibly in relation with extracellular matrix degradation; may be a prognostic marker for these risk factors in early cervical squamous cell carcinoma
DNM2	1785	21841817	tumor cells overexpressing Dyn2 protruded lamellipodia at twice the rate, migrated faster (180%) and farther (2.5-fold greater distance) on glass and through transwell chambers
DNM2	1785	21996738	this study identifies Dyn2 as an effector that mediates PDGFRalpha-SHP-2-induced glioma tumor growth and invasion
DNM2	1785	23537630	study provides evidence the large GTPase Dyn2 regulates the small GTPase Rac1 to potentiate invasive migration of pancreatic tumor cells; Dyn2 plays an essential role in regulating Rac1-mediated pancreatic tumor cell migration through modulation of the Rac1 activator Vav1 via a direct interaction
DNM2	1785	24706848	results demonstrate a regulatory relationship between DNM2, miR-199a, and HIF, with implications in cancer metastasis
ERBB2	2064	11839648	NH(2)-terminal truncated HER-2 protein but not full-length receptor is associated with nodal metastasis in human breast cancer
ERBB2	2064	12060497	HER-2/neu peptides can activate T cells in draining lymph nodes from women with invasive breast cancer.
ERBB2	2064	12065844	S-erbB-2 serum levels above 40 U/ml independently predicted unfavorable response to 2d-line hormone or chemotherapy in advanced metastatic breast cancer. 1st-line drugs may select for overexpression of erbB-2 genes and lesser response to 2d-line drugs.
ERBB2	2064	12209684	significant HER2 expression seen in high-grade, muscle-invasive urothelial carcinoma; but HER2 expression in context of paclitaxel-based chemotherapy associated with significantly reduced risk of death
ERBB2	2064	12607595	Expression of vascular endothelial growth factor in invasive ductal carcinoma of the breast and the relation to angiogenesis and p53 and HER-2/neu protein expression.
ERBB2	2064	12910292	expression of nm 23 and c-erbB-2 in primary tumor and metastases of colorectal adenocarcinoma showed that the incidence and expression of both protein markers in primary tumor tissue tended to increase after the appearance of liver metastases
ERBB2	2064	14520464	overexpression of HER2/neu is associated with the development of muscle-invasive transitional cell carcinoma of the bladder
ERBB2	2064	14555515	High levels of serum HER-2/neu reflect aggressiveness of metastatic breast cancer
ERBB2	2064	14566828	value of this tumor marker regarding relapse, metastasis and death in resectable non-small cell lung cancer
ERBB2	2064	14631375	HER-2 protein expression can be a useful tool in differentiating a primary cutaneous appendageal neoplasm from HER-2 expressing metastatic breast carcinoma.
ERBB2	2064	14664140	expression of c-erbB-2 protein may reflect biological behaviour of the tumour and may appear to be an important factor in development of colorectal cancer metastases

ERBB2	2064	14761474	C-erbB-2 gene may be an important regulating gene in the coal miners with pneumoconiosis complicated by pulmonary cancer, and as a reference index to determine lymph node metastasis and prognosis.
ERBB2	2064	15140287	Data provide the distribution frequency of HER2 protein expression and gene amplification in invasive ductal and lobular breast cancer.
ERBB2	2064	15211117	In pancreatic ductal adenocarcinoma, membrane HER-2 overexpression was more frequent in intraductal than in invasive components but the incidence of cytoplasmic HER-2 overexpression did not differ between intraductal & invasive components.
ERBB2	2064	15254682	EGFR, c-erbB-2, VEGF and MMP-2 and MMP-9 play an important role in tumor growth, invasion and metastasis in squamous cell carcinoma of the head and neck
ERBB2	2064	15335267	In patients with breast cancer, most CNS metastatic tumor deposits showed expression for either EGFR or HER-2/neu, and less often for both.
ERBB2	2064	15721309	a splice variant of decay-accelerating factor is expressed in c-erbB-2-positive mammary carcinoma cells showing increased transendothelial invasiveness
ERBB2	2064	16169726	HER-2/neu and p53 are likely to be involved in the regulation of COX-2 expression in invasive ductal carcinomas of the breast.
ERBB2	2064	16372244	IHC and FISH together showed HER-2 overexpression/gene amplification in 21% of breast invasive carcinomas.
ERBB2	2064	16596190	Overexpression of HER-2 was associated with sentinel lymph node micro-metastasis in breast cancer patients
ERBB2	2064	16681686	HER2 appears to lack a critical role in the progression from ductal carcinoma in situ and invasive ductal carcinoma and HER2 status is maintained in metastatic lesions.
ERBB2	2064	16685382	The activation of HER-2/CXCR4/ Akt signaling pathway in primary breast tumors may contribute to the formation of bone metastases in breast cancer.
ERBB2	2064	16739379	There was a significant relationship between the expression of HER-2/neu and clinical stages and lymph node metastasis in laryngeal squamous cell carcinoma.
ERBB2	2064	16782798	Observed consistent increases in persistence associated with HER2 overexpression indicate a prospective mechanism for invasiveness previously documented in HER2-overexpressing human breast tumors.
ERBB2	2064	16984552	Significantly higher serum HER2 level was associated with bone metastasis of prostate cancer patients
ERBB2	2064	17102066	study shows that newly diagnosed HER-2-overexpressing breast cancer patients are at increased risk for brain metastases
ERBB2	2064	17243161	Amplicon size is associated with response to trastuzumab in metastatic breast cancer.
ERBB2	2064	17322545	Her-2/neu protein overexpression by HER-2 gene amplification may occur in extrahepatic cholangiocarcinoma and constitute an independent prognostic factor in patients with lymph node metastases.
ERBB2	2064	17478762	Expression of c-erbB-2 and p53 has no prognostic value in patients with early-stage breast cancer in which axillary lymph node metastasis is absent.
ERBB2	2064	17511881	HER2 gene status remains highly conserved as breast cancers metastasise but discrepant results occur because of interpretational difficulties of HER2 amplification.
ERBB2	2064	17541441	Brain-metastasizing breast cancer belongs predominantly to the basal type or Her-2/neu type.
ERBB2	2064	17549338	Overexpression of Her-2 is associated with Lymph node metastasis in bladder cancer
ERBB2	2064	17690040	c-erbB-2 is highly correlated with differentiation grade and metastasis of the extrahepatic cholangiocarcinoma tumor
ERBB2	2064	17879632	Significant heterogeneity exists between Her-2/neu expression in the in situ component and invasive components of adenocarcinoma of the breast.
ERBB2	2064	17982484	cellular PTPN13 inhibits Her2 activity by dephosphorylating the signal domain of Her2 and plays a role in attenuating invasiveness and metastasis of Her2

			overactive tumors.
ERBB2	2064	18035692	A tight interaction between HER-2 and EPIL gene expression in invasive breast cancer cells is probable.
ERBB2	2064	18038879	Overexpresion of Her-2 is associated with micrometastatic disease in men with prostate cancer
ERBB2	2064	18097576	efficacy of molecular targeted therapy can be expected even for patients with metastatic lymph nodes as long as the primary tumors are positive for HER-2 expression.
ERBB2	2064	18172732	HER2 overexpression was found in only four cases (10.5%) of the studied primary tumors and in all cases the HER2 expression was retained in the paired metastases.
ERBB2	2064	18324648	The androgen receptor signaling pathways may contribute to development of metastatic disease in prostate cancer.
ERBB2	2064	18337451	Lipid raft-disrupting agents inhibited raft-associated CXCL12/CXCR4 transactivation of the HER2 and cellular invasion in prostate cancer cells.
ERBB2	2064	18372913	PAR1-stimulated EGFR and ErbB2 transactivation leads to prolonged extracellular signal-regulated kinase-1 and -2 signaling and promotes breast carcinoma cell invasion.
ERBB2	2064	18390199	Hpa may up-regulate the expression of C-erbB-2 in invasive ductal breast carcinoma.
ERBB2	2064	18415713	There was no strong association between HER-2/neu overexpression and gene amplification in invasive urothelial carcinomas, and polysomy 17 was higher in tumors showing HER-2/neu overexpression.
ERBB2	2064	18501058	Overexpression of HER2 is encountered in approximately 20% of invasive breast cancers.
ERBB2	2064	18571923	This study shows superior survival in HER2+ group following whole brain radiotherapy for cerebral metastases of breast cancer as compared to HER2- group.
ERBB2	2064	18591932	effects of HER2 amplification on carcinogenesis, tumorigenesis and invasion may be due to its effects on normal and malignant mammary stem/progenitor cells.
ERBB2	2064	18700025	analysis of EGFR and HER2 expression in primary cervical cancers and corresponding lymph node metastases
ERBB2	2064	18801081	Grade 1 invasive ductal carcinoma have a higher incidence of lymph node metastasis and may have Her-2-neu overexpression compared to tubular carcinoma.
ERBB2	2064	18832579	These data support the cooperative function of Pyk2 and FAK in breast cancer progression and suggest that dual inhibition of FAK and Pyk2 is an efficient therapeutic approach for targeting invasive breast cancer.
ERBB2	2064	18855148	findings indicate that serum HER2/neu levels are clinically valuable in monitoring metastatic breast cancer and non-small cell lung cancer patients.
ERBB2	2064	18948375	we have demonstrated an increase in ERBB2 receptor activation in incompletely resected preinvasive breast cancer
ERBB2	2064	19190626	ErbB2 signalling regulated focal adhesion turnover in invasive breast cancer cells.
ERBB2	2064	19192581	Patients with a HER2 positive tumor receiving trastuzumab more frequently develop brain metastases than patients with a HER2 negative tumor but have a more favorable prognosis.
ERBB2	2064	19208313	ER and PR showed inverse association with HER-2/neu while positive association was seen with lymph node metastases in breast carcinoma patients in Pakistan
ERBB2	2064	19225924	Paients with HER2-overexpressing breast cancer treated with trastuzumab had a high incidence of brain metastasis.
ERBB2	2064	19255335	we cannot recommend using serum HER2 extracellular domain levels to make trastuzumab or other treatment decisions for individual patients with advanced/metastatic breast cancer.

ERBB2	2064	19269014	The high level of HER-2 amplification in combination with the homogeneity of its expression in primary and metastatic tumors argues for a possible therapeutic utility of trastuzumab in HER-2-amplified gastric adenocarcinomas.
ERBB2	2064	19270645	Invasive micropapillary carcinoma more commonly showed immunoreactivity for MUC1, CA125, and Her2Neu compared to invasive urothelial carcinoma with retraction artifact
ERBB2	2064	19443884	although HER2/neu amplification, found in high grade and invasive tumors, is a rare event in transitional cell carcinoma of urinary bladder
ERBB2	2064	19447586	retrospectively examined a series of breast cancer patients with brain metastases for the presence of HER-2
ERBB2	2064	19622585	Non-small cell lung carcinoma brain metastases have some significant differences in HER family receptor-related abnormalities from primary lung tumors.
ERBB2	2064	19729798	no difference in metastatic breast cancer survival between HER2-nonamplified and HER2-amplified patients for subgroup as whole
ERBB2	2064	19732720	14-3-3zeta cooperates with ErbB2 to promote ductal carcinoma in situ progression to invasive breast cancer by inducing epithelial-mesenchymal transition.
ERBB2	2064	19889613	5.1% of invasive bladder carcinomas had a HER2 gene amplification.
ERBB2	2064	19923084	The high expressions of VEGF-C and C-erbB-2 are closely related to lymph node metastasis in breast cancer patients.
ERBB2	2064	19956951	Our study shows that HER2-overexpressing breast cancer displays characteristic patterns of relapse and metastatic spread depending on estrogen receptor status
ERBB2	2064	20023245	Metastatic cancer-related hyperprolactinemia and lack of surgery-induced hyperprolactinemia are statistically more frequent in HER2-positive patients.
ERBB2	2064	20027338	A relationship between high levels of EGFR and HER-2 in the tumors of patients with esophageal squamous-cell carcinoma and intravascular tumor invasion and poor outcome of the disease was detected.
ERBB2	2064	20043103	Interpretive guidelines for HER2 status assay in circulating cancer cells (CTCs) and raise great opportunities for using CTCs as non-invasive and 'real-time' biopsy to examine and monitor the status of tumor markers.
ERBB2	2064	20087651	Quantified/identified differences in protein levels between tumor and adjacent normal breast tissue from the same breast in 18 women with stage I/II ER positive/Her2/neu negative invasive breast cancer.
ERBB2	2064	20126619	Data are consistent with a preferential localization of KLF6 in the nuclear compartment of early stage and small HER2-ERBB2 overexpressing ductal breast tumor cells, also presenting lymph node metastatic events
ERBB2	2064	20159713	The expression of HER-2/neu may play a role in the invasion, metastasis and progression of hepatocellular carcinoma.
ERBB2	2064	20178580	HER2 may have a role in response to trastuzumab in patients with metastatic breast cancer
ERBB2	2064	20202990	is expressed in breast cancer and metastatic lymph nodes
ERBB2	2064	20419393	HER2 gene amplification activates lamellipodia formation in breast cancer cells, which initiates their metalloproteinase-independent migration
ERBB2	2064	20585851	Phase II study of nab-paclitaxel, bevacizumab, and gemcitabine as first-line therapy for patients with HER2-negative metastatic breast cancer.
ERBB2	2064	20670419	The HER2 positivity rate of breast tumors (DIN1C-3, invasive carcinoma) may be as low as 9%. Immunohistochemistry as firstline testing for HER2 may result in false positive and false negative results.
ERBB2	2064	20731018	Data indicate that high ErbB2 expression enhances cholangiocarcinoma invasion, motility and proliferation via the AKT/p70S6K pathway.
ERBB2	2064	20859679	Compare HER2 status of circulating tumor cells in patients with metastatic breast cancer using two different methods.

ERBB2	2064	20929083	HER2/neu is a marker of prognostic significance that is routinely assessed in invasive cancer.
ERBB2	2064	20961652	Analyses indicate that p130Cas expression in ErbB2 positive human breast cancers significantly correlates with higher risk to develop distant metastasis, thus underlying the value of the p130Cas/ErbB2 synergism in regulating breast cancer invasion.
ERBB2	2064	20971737	ErbB-2 signaling via the phosphatidylinositol 3-kinase pathway results in up-regulated matriptase zymogen activity, which contributes to prostate cancer cell invasion.
ERBB2	2064	21050467	in ER negative basal and HER2+ breast cancer, gene expression modules reflecting T-cell helper-1 (Th1) and T-cell helper-2 (Th2) mediated immune responses play antagonistic roles as major risk factors for distant metastasis
ERBB2	2064	21172893	Trastuzumab-DM1 has robust single-agent activity in patients with heavily pretreated, HER2-positive metastatic breast cancer and is well tolerated at the recommended phase II dose.
ERBB2	2064	21198263	HER-2 expression significantly correlates with Survivin at the protein level in primary invasive ductal breast cancer tissues
ERBB2	2064	21259244	Expression of ADAM 10 in gastric cancer is significantly associated with lymph node and distant metastasis, high C-erbB-2 expression, and poor prognosis.
ERBB2	2064	21371829	COX-2 overexpression correlates with HER2 overexpression in invasive breast carcinomas and may provide a clinically useful biomarker for estimating tumor aggressiveness.
ERBB2	2064	21383283	Bevacizumab combination therapy improves clinical benefit in terms of increased progression-free survival in first-line treatment of Her-2 negative metastatic breast cancer.
ERBB2	2064	21437249	A comprehensive immunohistochemical study was performed to investigate the protein expression of eight CT genes in 454 invasive ductal carcinomas, including 225 ER/PR/HER2-negative (triple-negative) carcinomas.
ERBB2	2064	21445634	in ileal neuroendocrine cancer, HER-2/neu overexpression plays a role in the carcinogenetic process and by triggering the altered expression of c-Met and MTA-1, may activate the molecular pathway(s) promoting tumor progression and metastasis development.
ERBB2	2064	21503112	In invasive breast cancer ER is negatively associated with HER-2 amplification, whereas tumor emboli and lymph node status are positively associated with HER-2.
ERBB2	2064	21609713	HER2 expression level is directly related to breast cancer invasion.
ERBB2	2064	21640482	Her2 amplification in metastasising bladder cancer is relatively frequent, is homogeneous in each tumour component, and predicts early death.
ERBB2	2064	21667123	Expression of ER, HER2, and TOP2A displayed discordance with a sufficient frequency to emphasize the role of confirmatory biopsies from metastatic lesions in future management of recurrent breast cancer.
ERBB2	2064	21708009	There was no association between HER2/neu and VEGF expression among 317 colon cancer patients with all tumor stages. HER-2/neu expression was significantly correlated with tumor size & distant metastases.
ERBB2	2064	21733550	Multifocal/multicentric invasive ductal carcinomas usually have a single phenotype in terms of hormone receptors, human epidermal growth factor receptor 2, and molecular subtypes.
ERBB2	2064	21840040	We confirmed evidence that discordance of ER/Her2 expression between primary and metastatic breast carcinoma does occur.
ERBB2	2064	21913743	results indicated that expression patterns of ER, NM23-H1, and HER2 in primary breast cancer lesions warn that cells might have metastatic potential

ERBB2	2064	21953571	Report population pharmacokinetics of trastuzumab emtansine, a HER2-targeted antibody-drug conjugate, in patients with HER2-positive metastatic breast cancer.
ERBB2	2064	21961357	Her2-Neu was expressed in 36.1% of 122 Mexican breast cancer patients. There was a close with relation the tumour size & the presence of lymph node metastases but not with age, differentiation or estrogen receptors.
ERBB2	2064	21966491	Cinical study indicates that co-overexpression of Her2 with GEP100 in primary lung adenocarcinomas of patients is correlated with the presence of their node-metastasis with a statistical significance.
ERBB2	2064	21986939	A positive feedback loop between HER2 and ADAM12 in human head and neck cancer cells increases migration and invasion.
ERBB2	2064	22081131	SLP-2 and HER2/neu can play a role in lymph node/distant metastases of breast cancers
ERBB2	2064	22092393	the discordant HER2 amplification observed in metastatic gastric carcinoma lesions is explained substantially by heterogeneity within primary tumours.
ERBB2	2064	22102915	ErbB2 overexpression is associated with chemoradiation resistance in muscle-invasive bladder cancer.
ERBB2	2064	22104159	This is the first report of a positive response to trastuzumab in a patient with HER2-overexpressing metastatic gastric cancer that was accompanied by bone marrow involvement and severe thrombocytopenia.
ERBB2	2064	22119825	Invasive ductal mammary carcinoma type was analyzed for comparative investigation of hormonal receptors (estrogen receptor ER and progesterone receptor PR) and Her2/neu oncoprotein expression.
ERBB2	2064	22253521	Data suggest that iIn gastric cancer, HER2 gene amplification is the main mechanism for HER2 protein overexpression and is preserved in lymph node metastases.
ERBB2	2064	22262199	Report the interplay of HER2/HER3/PI3K and EGFR/HER2/PLC-gamma1 signalling in breast cancer cell migration and dissemination.
ERBB2	2064	22335909	HER-2 is stably expressed in primary gastric cancer and metastatic lymph nodes. HER-2 and leptin play an important role in the progression and angiogenesis of gastric cancer
ERBB2	2064	22340175	AEG-1 might facilitate the proliferation and invasion of breast cancer cells by upregulating HER2/neu expression.
ERBB2	2064	22378040	ErbB-2 overexpression in human breast cancer cell lines leads to phosphorylation & activation of Plexin-B1. This was required for ErbB-2-dependent activation of RhoA & RhoC & promoted invasive behavior.
ERBB2	2064	22410801	HER2 expression was less frequent in resectable gastric cancer than in metastatic gastric cancer; the impact of HER2 expression on survival was limited
ERBB2	2064	22460089	Aberrantly expressed miR-125b contributes to HEC1B cells invasion partly through directly down-regulating ERBB2 protein expression in endometrioid endometrial cancer.
ERBB2	2064	22504780	Letter/Case Report: HER2-positive metastatic apocrine carcinoma of the skin showing complete remission following administration of lapatinib/capecitabine.
ERBB2	2064	22534547	Combinations of trastuzumab plus docetaxel-based regimens were well tolerated and effective in previously treated metastatic gastric cancer of Chinese patients with HER2 over-expression or gene amplification.
ERBB2	2064	22646266	human epidermal growth factor receptor 2 (HER2/neu) protein, a 185-kDa transmembrane tyrosine kinase receptor, is associated with tumor proliferation, migration, and differentiation
ERBB2	2064	22737248	analysis of multiorgan metastasis of human HER-2+ breast cancer in Rag2-/-;Il2rg-/- mice and treatment with PI3K inhibitor
ERBB2	2064	22848366	Correlation of HER2, p95HER2 and HER3 expression and treatment outcome of lapatinib plus capecitabine in her2-positive metastatic breast cancer.

ERBB2	2064	22883999	Data suggest that HER-2 and leptin (but not leptin receptor) are up-regulated in gastric cancer and are associated with tumor invasiveness, lymphatic metastasis, and angiogenesis.
ERBB2	2064	22899400	Propose that HER2 represents a reasonable target for molecular therapy in HER2- positive metastatic or recurrent mucinous ovarian carcinoma.
ERBB2	2064	22911549	c-erbB-2 overexpression may have a prognostic significance in patients with metastatic gastric cancer.
ERBB2	2064	22952421	clinical data analyses revealed a strong correlation between CD151 and ErbB2 expression and metastasis-free survival of breast cancer patients.
ERBB2	2064	22973057	Patients with HER2(+) metastatic breast cancers have epithelial-to-mesenchymal transition-circulating tumor cells.
ERBB2	2064	23087329	A subset of high-grade non-muscle-invasive bladder cancers contain Her2 amplification and are associated with markedly aggressive behaviour.
ERBB2	2064	23220854	EGFR and Her2 were significantly upregulated in lymph node metastasized colorectal cancer (LNM CRC) compared to non-LNM CRC.
ERBB2	2064	23225912	HER2 examination on fluorescence in situ hybridization using fine-needle aspiration cytology samples of tumors in recurrent/metastatic sites or disseminated tumor cells in effusion is beneficial
ERBB2	2064	23279650	The positive rate of vasculogenic mimicry in invasive breast carcinoma correlated with the increased HER2 expression.
ERBB2	2064	23307470	WNT10B/beta-catenin signalling induces HMGA2 and proliferation in metastatic breast cancer tumours devoid of ERalpha, PR and HER2 expression.
ERBB2	2064	23318431	these data show that ionizing radiation-mediated activation of ErbB2 and induction of 14-3-3zeta collaborate to regulate FoxM1 and promote invasion of breast cancer cells
ERBB2	2064	23348520	HER2 gene copy number status is asociated with clinical efficacy in metastatic colorectal cancer
ERBB2	2064	23348899	Heterogeneous HER2 expression in primary gastric tumors and the variable lymph node metastasis status suggest evaluating multiple samples.
ERBB2	2064	23392720	Our study suggested that ER/PR status and histological grade are risk factors for the development of bone metastasis in breast cancer patients.
ERBB2	2064	23447672	interaction between LPP and alpha-actinin, an actin cross-linking protein, is necessary for TGFbeta-induced migration and invasion of ErbB2-expressing breast cancer cells.
ERBB2	2064	23468259	The study aimed at examining the relationship between HER2 immunohistochemistry assessment scores in paired core needle biopsies and whole tissue sections of invasive ductal breast carcinoma.
ERBB2	2064	23483255	Data indicate that monitoring tissue HER-2-positive breast cancer patients with serum HER-2 has a sufficient sensitivity to detect metastatic recurrence, while its use in monitoring of tissue HER-2-negative patients is unsatisfactory.
ERBB2	2064	23523315	Her-2/neu over-expression and parameters assessing metastatic burden in the SLN, particularly total metastatic area (TMA), predicted the presence of NSLN involvement and extranodal disease in patients with breast carcinoma and SLN metastases.
ERBB2	2064	23536719	these results suggest that coupling HDAC and HER2 inhibitory activities to an EGFR inhibitor may potentially be effective in overcoming drug resistance and preventing cancer cell migration
ERBB2	2064	23542467	HER2 mediates the effects of epidermal growth factor (EGF) on Snail, Slug and E-cadherin expression as well as on ovarian cancer cell invasion.
ERBB2	2064	23548965	BMs were detected very rarely in asymptomatic, non-metastatic HER2-positive breast cancer patients compared with asymptomatic, metastatic patients.
ERBB2	2064	23555577	newly identified HER2 epitopes seem to be crucial for an effective immune response against HER2-overexpressing breast cancers and might help discriminating

			between metastatic breast cancer and early breast cancer
ERBB2	2064	23575477	Data indicate that genomic profiling of relapsed CDH1-mutated invasive lobular carcinoma (ILC) featured a high incidence of ERBB2 alterations.
ERBB2	2064	23592719	DOCK1-Rac signaling as an HER2 effector pathway essential for HER2-mediated breast cancer progression to metastasis.
ERBB2	2064	23637122	Analysis of plasma DNA with digital PCR has the potential to screen for the acquisition of HER2 amplification in metastatic breast cancer.
ERBB2	2064	23665199	Two independent high-risk primary breast cancer subgroups for developing brain metastases were identified, represented by genetic alterations in either HER2 or EGFR/PTEN-driven pathways.
ERBB2	2064	23691049	Serum HER 2 extracellular domain level is correlated with tissue HER 2 status in metastatic gastric or gastro-oesophageal junction adenocarcinoma
ERBB2	2064	23723333	This study confirms that discordance in ER and PR receptor expression between the primary breast tumor and the corresponding metastatic lesions is high, whereas HER2 status remains relatively constant.
ERBB2	2064	23818346	Immunohistochemical expression of HER-2 was not associated with characteristics of invasive breast cancers.
ERBB2	2064	23867476	Expression of Delta-HER2 induces tumor formation and metastasis.
ERBB2	2064	23988598	Pertuzumab, a novel HER2 dimerization inhibitor, has been shown to be effective in the treatment of metastatic HER2-positive breast cancer--{REVIEW}
ERBB2	2064	24023299	In contrast to the higher incidence reported at the population level, 20-30% HER2-positive cases for metastatic carcinomas, and only 11% of the screen-detected breast carcinomas displayed HER2/neu gene amplification
ERBB2	2064	24036211	MTK1 was identified in the HER2/HER3-HRG mediated extracellular acidification and cell migration pathway in breast cancer cells.
ERBB2	2064	24104881	Breast cancer with a HER2 positive subtype is associated with the longest survival after diagnosis of distant metastases.
ERBB2	2064	24158524	siRNA cocktail, and co-downregulation of VEGF and HER2 result in significant inhibition of gastric cancer growth and migration in vitro
ERBB2	2064	24261710	HER2 status in primary gastric cancer is a reliable basis for deciding whether to treat with anti-HER2 agents in patients with lymph node metastases.
ERBB2	2064	24286315	miR-221 may promote trastuzumab resistance and metastasis of HER2-positive breast cancers by targeting PTEN, suggesting its role as a potential biomarker for progression and poor prognosis
ERBB2	2064	24311197	High-level ERBB2 amplification is associated with shorter time-to-metastasis, but improved response to trastuzumab in metastatic breast cancer.
ERBB2	2064	24322014	calretinin immunoexpression and the hormonal status for estrogen receptors (ER), progesterone receptors (PR), and ERRB2, in 33 cases of cutaneous metastases of ductal breast carcinoma, as well as in seven primary cutaneous apocrine tumors, were studied
ERBB2	2064	24344005	Capecitabine/cisplatin doublet has an encouraging antitumor activity with acceptable and manageable toxicity in anthracycline- and taxane-pretreated HER-2 negative metastatic breast carcinoma patients.
ERBB2	2064	24390418	High HER2 expression is associated with brain metastases arising from breast cancer with stereotactic radiosurgery.
ERBB2	2064	24643685	HER2 was positive in nearly 52 % of the primary tumors, and these expressed HER2 in corresponding lymph node and liver metastases in 93 and 100 % of the cases, respectively
ERBB2	2064	24719937	The expression of her-2 is closely related to differentiation degree, infiltration depth, lymph nodes' metastasis and pTNM stage of the esophageal cancer and to prognosis.

ESRRA	2101	20870744	Data provide evidence for an autocrine regulatory loop involving transcriptional upregulation of WNT11 by ERRalpha and beta-cat that influences the migratory capacity of cancer cells.
ETS2	2114	20145133	Ets2 is a central driver of a transcriptional program in tumor-associated macrophages that acts to promote lung metastasis of breast tumors.
FN1	2335	17512904	Twist regulates cell motility and invasion in gastric cancer cell lines, probably through the N-cadherin and fibronectin production
FN1	2335	18006840	expression of the alternatively spliced extra-domain A (EDA) of fibronectin in the neovasculature of metastases and primary tumors of human cancer patients was shown
FN1	2335	20097172	these data suggest that hypoxia promotes oral squamous cell carcinoma cell invasion that is elicited by HIF-1alpha-dependent alpha5 integrin and fibronectin induction.
FN1	2335	20359090	The expression of Col I, Col IV and Fn was closely related to tumor invasion, the regional lymph node metastasis and other pathological features in laryngeal squamous cell carcinoma.
FN1	2335	20530259	MSF is a novel marker associated with the M2 stage of macrophage polarization expressed by tumor-conditioned macrophages; MSF may contribute to macrophage-mediated promotion of cancer cell invasion and metastasis.
FN1	2335	20939933	rhFNHN29 and rhFNHC36 inhibit adhesion and invasion of liver cancer cells
FN1	2335	21036738	study suggests that FN and tissue transglutaminase (TG2) facilitate the metastatic activity of A431 tumor cells, and this may be partly attributed to TG2 enhancement of the association of FN and beta integrin
FN1	2335	21691075	Metastasis suppressor KAI1/CD82 attenuates the matrix adhesion of human prostate cancer cells by suppressing fibronectin expression and beta1 integrin activation.
FN1	2335	21924268	miRNA-1 targets fibronectin1 and suppresses the migration and invasion of the HEp2 laryngeal squamous carcinoma cell line.
FN1	2335	23098431	Serum oncofetal fibronectin mRNA in differentiated thyroid carcinoma cannot identify those with metastatic disease.
FN1	2335	23653350	Data indicate that metastatic breast cancer (BC) cells that failed to activate STAT3 downstream of EGFR did display robust STAT3 activity upon adhesion to fibronectin (FN).
FN1	2335	23684510	Fibronectin expression in carcinoma cells correlates with tumor aggressiveness and poor clinical outcome in patients with invasive breast cancer.
FN1	2335	23791940	MSF expression was upregulated in NSCLC tumors and promoted migration and invasion in A549 cells.
FN1	2335	23895273	The SOX2-FN1 axis is a key pathway in mediating the migration and invasion of ovarian cancer cells
FN1	2335	24743511	Endothelial cell-derived fibronectin extra domain A promotes colorectal cancer metastasis via inducing epithelial-mesenchymal transition
GATA4	2626	23239811	Results thus identify a novel epigenetic mechanism by which MYC activates GATA4 leading to metastasis in lung adenocarcinoma.
GLI2	2736	20660365	In human melanoma cells, GLI2 expression was heterogeneous, associated with loss of E-cadherin and increased in the most aggressive tumors. In this preclinical study, GLI2 was directly involved in driving melanoma invasion and metastasis.
GLI2	2736	23356443	high expression level of Gli2 was significantly associated with tumor differentiation, encapsulation, vascular invasion, early recurrence, and intra-hepatic metastasis in hepatocellular carcinoma.

GLI2	2736	23371028	GLI2 was correlated with lymphovascular invasion and intestinal type gastric cancer.
GNAS	2778	17186357	GNAS1 T393C single nucleotide polymorphism is a novel genetic host factor for disease progression in patients with invasive breast carcinoma
HLA-C	3107	20857097	HLA-Cw group 1, which serves as ligand for certain KIR, was significantly overtransmitted in women with invasive cervical cancer, and particularly in the subgroup of women infected with high risk HPV16 or 18 subtypes.
HMGA1	3159	15247513	HMG-I(Y) expression may have a role in intrahepatic metastasis of hepatocellular carcinoma
HMGA1	3159	17178855	HMGA1 may be a novel molecular determinant of invasiveness and metastasis, as well as a potential therapeutic target, in pancreatic adenocarcinoma.
HMGA1	3159	21138859	miR-296 regulates HMGA1 expression and is associated with prostate cancer growth and invasion
HMGA1	3159	21300033	the expression of HMGA1 is related to malignant proliferation, invasion, and differentiation of glioblastoma and glioma from the prospective of tumor stem cells
HMGA1	3159	21573994	knockdown of the HMGA1 gene could inhibit growth and metastasis potentials in tumor cells
HMGA1	3159	21984063	These results indicated that expression of HMGA1 correlates significantly with malignancy, proliferation, invasion, and angiogenesis of gliomas. We conclude that HMGA1 may be a potential biomarker and rational therapeutic target for human tumors.
HMGA1	3159	22503056	HMGA1 knockdown induced inhibition of colony formation and apoptosis in renal cell carcinoma cells.HMGA1 has a role in the metastatic including the acquisition of invasion potential and anoikis resistance.
HMGA1	3159	23201162	miR-137, induced by its upstream transcription factor HMGA1, can suppress colorectal cancer cell invasion and metastasis by targeting FMNL2.
HMGA1	3159	23394223	Expression in laryngeal squamous cell carcinoma is associated with clinical stage, histological grade, and lymph node metastasis
HMGA1	3159	23545254	Interfering with HMGA1 expression reduces the tumorigenic and metastatic potential of breast cancer cells in vivo.
HMGA1	3159	23835740	High HMGA1 expression is associated with colorectal cancer metastasis.
HRG	3273	21215706	Host-produced HRG inhibits tumor growth and metastasis by skewing tumor-associated macrophages (TAM) polarization away from the M2- to a tumor-inhibiting M1-like phenotype. Skewing of TAM polarization by HRG relies substantially on downregulation of PlGF.
IL15	3600	14581351	IL-15 produced by metastatic colon carcinoma cells can induce hyperplasia in the mucosa adjacent to colon cancer, thus contributing to angiogenesis and progression of the disease
ITIH3	3699	11807786	Transfection into H460M Human large cell lung carcinoma line decreased tumor metastasis in nude mice
KCNH2	3757	14744775	herg1 gene and HERG1 protein were expressed in a high percentage of primary human colorectal cancers, with the highest incidence occurring in metastatic cancers
KCNH2	3757	19495974	hERG1 channel was proved to be a potential biomarker for gastric cancer invasion and survival.
KIFC1	3833	19190132	High KIFC1 expression is associated with metastatic spread to the brain in primary non-small cell lung cancer.
KRAS	3845	12644542	results demonstrate that the mutational status of BRAF and KRAS is distinctly different among histologic types of ovarian serous carcinoma, occurring most frequently in invasive micropapillary serous carcinomas and its precursors, serous borderline tumors
KRAS	3845	15221969	Sensitivity of PNA clamp PCR assay in identifying occult micrometastases in the sentinal lymph nodes of colorectal cancer patients by single-base mutation analysis.

KRAS	3845	16257181	The results suggest that oncogenic K-Ras enhances the malignant phenotype and identify the mitogen-activated protein kinase p38 as a target to inhibit oncogenic K-Ras-induced pancreatic tumor cell migration.
KRAS	3845	17465446	Genetically, the non-polypoid growth type of submucosal invasive colorectal carcinoma shows much less frequent K-ras mutation.
KRAS	3845	17534846	it is suggested that atypical adenomatous hyperplasia (AAH) could develop by either KRAS or EGFR gene mutation, but AAH harbouring a KRAS gene mutation might not progress further to an invasive cancer
KRAS	3845	17998284	KRAS WT status is associated to survival benefit in cetuximab treated metastatic colorectal cancer.
KRAS	3845	18202412	results confirm the high prognostic value of KRAS mutations on response to cetuximab and survival in metastatic colorectal cancer patients treated with cetuximab
KRAS	3845	19056857	KRAS mutations in either primary or metastatic tumors from patients with colorectal cancer is concordant.
KRAS	3845	19179548	Report the importance of KRAS mutations and EGF61A>G polymorphism to the effect of cetuximab and irinotecan in metastatic colorectal cancer.
KRAS	3845	19282104	study of KRAS/BRAF mutation status in a large and well-documented cohort of primary and metastatic renal cell carcinoma
KRAS	3845	19376842	EGFR/KRAS mutation testing of multiple lung adenocarcinomas can assist in differentiating multiple primary lung adenocarcinomas from metastatic lesions.
KRAS	3845	19378369	Several key studies have demonstrated that patients with mutant KRAS do not respond to treatment with epidermal growth factor inhibitors. This finding has several implications for clinicians who treat patients with metastatic colorectal cancer.
KRAS	3845	19549774	Detection of KRAS mutational status in CTCs, by gene expression array, has potential for clinical application in selecting metastatic colorectal cancer patients most likely to benefit from cetuximab therapy.
KRAS	3845	19584155	Results suggest that the mutations of EGFR, KRAS, BRAF between primary tumors and corresponding lymph node metastases should be considered whenever mutations are used for the selection of patients for EGFR-directed tyrosine kinase inhibitor therapy.
KRAS	3845	19603018	Assessing KRAS and BRAF mutations might help optimising the selection of the candidate metastatic colorectal cancer patients to receive anti-EGFR moAbs.
KRAS	3845	19603024	KRAS mutation was associated with lack of response (P=0.002) and shorter progress free survival (P=0.09) in patients with metastatic colorectal cancer.
KRAS	3845	19740513	EGFR and KRAS status of primary lung carcinomas may not predict the status in the corresponding metastases.
KRAS	3845	20010090	The study suggests that activating KRAS mutants is a particularly important independent predictive marker in metastatic colorectal cancer patients treated with cetuximab
KRAS	3845	20020061	These results suggest a role for KRAS mutations in the propensity of primary colorectal tumors to metastasize to the lung.
KRAS	3845	20049644	KRAS mutations only in metastases is not associated with resistance to anti-EGFR treatment in primary colorectal tumors.
KRAS	3845	20049979	18/19 (94.7%) metastatic colorectal cancer smears were perfectly adequate for codon 12 and 13 KRAS mutational analysis by direct gene sequencing.
KRAS	3845	20100685	KRAS mutation testing, preferably at diagnosis, must now be considered a new standard of care for patients with metastatic colorectal cancer being considered for EGFR-targeting
KRAS	3845	20591910	Mutations in KRAS and BRAF genes are not directly implicated in the development of a MELF (macrocystic, elongated, and fragmented) pattern of invasion in endometrial carcinoma.
KRAS	3845	20619739	if KRAS is not mutated, assessing BRAF, NRAS, and PIK3CA exon 20 mutations (in that order) gives additional information about the efficacy of cetuximab plus

			chemotherapy in metastatic colorectal cancer.
KRAS	3845	20645028	samples of metastatic colorectal cancer were tested for the presence of the seven most common mutations in the KRAS gene and the V600E mutation in the BRAF gene
KRAS	3845	20840818	Expression level of EGFR protein and mutation frequency of KRAS gene in primary tumors were higher than that in metastases.
KRAS	3845	21129603	This review focuses on current knowledge regarding the molecular landscape of metastatic colorectal cancer including and beyond KRAS, and will summarize novel rationally-developed combinatorial regimens that are being evaluated in early clinical trials.
KRAS	3845	21155011	Data show that KRAS mutation and PTEN protein expression are significantly correlated with the response rate and survival time of Chinese metastatic colorectal cancer patients treated with cetuximab.
KRAS	3845	21169348	Mutated KRAS status in tumors of Japanese patients with metastatic colorectal cancer is associated with lack of response to panitumumab therapy
KRAS	3845	21174225	Combination chemotherapy with biweekly cetuximab and irinotecan was effective for pretreated metastatic colorectal cancer with wild-type KRAS.
KRAS	3845	21178719	KRAS mutation analysis helps to differentiate between pulmonary metastasis from colon adenocarcinoma in situ and primary lung adenocarcinoma
KRAS	3845	21228335	KRAS mutation is associated with treatment response for metastatic colorectal cancer.
KRAS	3845	21239505	KRAS mutation seems to be associated with metastasis in specific sites, lung and brain, in colorectal cancer patients
KRAS	3845	21364589	Kras(G12D) allele promotes metastasis in pancreatic cancer cells partly through the downregulation of E-cadherin.
KRAS	3845	21414214	these results suggest that a considerable proportion of NSCLC in Chinese population showed discrepancy in KRAS and EGFR mutation status between primary tumors and corresponding metastases.
KRAS	3845	21424126	data indicated that colorectal cancers with KRAS and PIK3CA bi-mutations are more likely to develop into liver metastasis
KRAS	3845	21468700	Expression of chemokine receptor CCR5 correlates with the presence of hepatic molecular metastases in K-ras positive colorectal cancer.
KRAS	3845	21478103	A review of several studies revealed a high rate of compliance of the KRAS gene mutational status in primary and metastatic tumors.
KRAS	3845	21497370	KRAS mutation in metastatic tumors is more commonly associated with advanced stages of cancer and correlates with a heavy smoking history.
KRAS	3845	21508389	Combined KRAS and TP53 mutation status is not predictive in CAPOX-treated metastatic colorectal cancer.
KRAS	3845	21550229	KRAS mutation status is a treatment effect modifier for anti-EGFR antibodies in metastatic colorectal cancer
KRAS	3845	21555681	Case Report: KRAS-mutation positive, metastatic tonsil carcinoma with cancer stem-like cell features and long-term response to gefitinib.
KRAS	3845	21573767	Heterogeneous distribution of K-ras codon 12 and 13 mutations within primary tumor, or between primary tumor and lymph node metastases, was demonstrated for 15 (20%) of 74 colon cancer patients having K-ras mutations.
KRAS	3845	21795973	compared with KRAS codon 12 mutations, codon 13-mutated metastatic colorectal cancer presents as a more aggressive disease frequently associated with local and distant metastases at first diagnosis
KRAS	3845	21825899	Heterogeneous KRAS mutations within the primary colorectal tumor might play an important role for discordant KRAS status between primary and metastatic sites.
KRAS	3845	21855038	Mutant KRAS is associated with low drug response in metastatic colorectal cancer.
KRAS	3845	21894049	KRAS mutation is associated with metastatic pancreatic ductal adenocarcinoma.

KRAS	3845	21943101	KRAS mutation G12V enhances the ability of colon adenocarcinoma cells to migrate and invade through filopodia formation and PI3K-dependent Cdc42 activation
KRAS	3845	21962894	KRAS mutation is associated with metastatic colorectal cancer
KRAS	3845	21982684	KRAS gene may be instable during the metastatic process observed in non-small cell lung cancer, whereas the mutant KRAS genotype in the primary tumor could be more likely to change back to the wild-type after metastasis.
KRAS	3845	22000810	KRAS mutation is associated with metastatic colorectal cancer.
KRAS	3845	22113502	loss of heterozygosity at Kras can provide cancer cells functional growth advantages and promote metastasis.
KRAS	3845	22177492	A considerable proportion of NSCLC in Chinese patients showed discrepancy in KRAS and EGFR mutation status between primary tumors and corresponding metastases.
KRAS	3845	22259183	The KRAS status varied among different areas of the same metastatic focus in metastatic colorectal adenocarcinomas.
KRAS	3845	22446022	KRAS may have a role in response of metastatic colorectal cancer to cetuximab
KRAS	3845	22638623	KRAS mutation status is predictive of cetuximab response in the Japanese metastatic colorectal cancer patients.
KRAS	3845	22674181	KRAS mutation is not associated with response to therapy in metachronous and synchronous metastatic colorectal adenocarcinoma
KRAS	3845	22744738	data showed that there was no significant difference in the mutation frequency of the KRAS gene between primary and distant metastatic colorectal tumors or lymph node metastatic tumors
KRAS	3845	22791568	K-RAS mutations and amino acid substitutions are associated with metastatic colorectal cancer.
KRAS	3845	22805857	K-ras mutation is strongly associated with perineural invasion phenotypically. K-ras mutation is an independent prognostic factor of intrahepatic cholangiocarcinoma after hepatectomy.
KRAS	3845	22876814	Organs initially involved by distant metastasis were different according to the KRAS mutational status inmetastatic or recurrent colorectal cancer (MRCRC) patients.
KRAS	3845	22948721	Data suggests that KRAS codon 12 mutation may not represent a homogeneous entity in metastatic colorectal cancer when treated with cetuximab-based first-line therapy.
KRAS	3845	22971512	A high frequency of KRAS gene mutations exists in Chinese patients with colorectal cancer, and are associated with poor survival, tumor differentiation and liver metastasis.
KRAS	3845	22972628	metastatic colorectal cancer patients with the KRAS p.G13D mutation appeared to benefit more from cetuximab than patients who had tumors with KRAS codon 12 mutations
KRAS	3845	23027075	This study provided further evidence for the prognostic importance of KRAS status in terms of recurrence-free and overall survival in patients with colorectal cancer liver metastases.
KRAS	3845	23075074	A meta-analysis was performed to compare KRAS gene mutations in colorectal cancer tissue samples with primary and metastatic colorectal cancers
KRAS	3845	23090619	Metastatic colorectal cancer patients with KRAS codon 13 mutations demonstrate a greater clinical response to anti-EGFR treatment than patients with other KRAS mutations [review]
KRAS	3845	23098991	Studied differential miRNA expression in metastatic colorectal cancer by microarrays from primary tumors of 33 patients who had wild-type KRAS/BRAF and third- to

			sixth-line anti-EGFRmAb treatment, with/without irinotecan.
KRAS	3845	23099803	Ddata suggest that KRAS alterations are linked to clinical phenotypes in endometrial carcinomas with increase in copy-number and mRNA expression levels from primary to metastatic lesions.
KRAS	3845	23111797	By identifying the KRAS mutational status at the metastasized colorectal carcinoma, a statement about the benefit of an anti-EGFR-therapy can be given, which is nowadays the basis of diagnostic and therapy of this cancer.
KRAS	3845	23134218	High KRAS expression is associated with the proliferation and invasion of non-small cell lung cancer.
KRAS	3845	23157828	KRAS mutation has no significant correlation with colorectal carcinoma patients' age, tumor site, tumor gross appearance, degree of differentiation, depth of invasion, TNM stages, lymphatic invasion, abdominal or distant metastases and prognosis.
KRAS	3845	23161513	Intercellular transfer of mutant KRAS is a characteristic feature of invasive colonic cancer cells.
KRAS	3845	23280667	Findings indicate that K-RAS regulates both caveolin-1 expression and other factors affecting caveolin-1 functions in colon cancer-derived cell migration.
KRAS	3845	23337026	EGFR and KRAS mutational rates were comparable in primary and unpaired metastatic lung adenocarcinoma in pre-chemotherapy and postchemotherapy groups.
KRAS	3845	23473612	This study confirmed that KRAS testing is definitely part of the management of most of metastatic colorectal cancer patients, despite discrepancies observed in the rate of prescription and the time of results.
KRAS	3845	23495083	KRAS mutations are associated with metastatic lung adenocarcinoma.
KRAS	3845	23510802	Data show that KRAS codon 12/13 mutations have negative prognostic value in metastatic colorectal cancer patients treated with FOLFOX/XELOX, but is not predictive of treatment benefit with cediranib, using progression-free survival and overall survival.
KRAS	3845	23558076	DAB2IP expression was reduced in patients with pancreatic cancer compared with those with no cancer. DAB2IP expression was correlated with the KRAS gene, perineurial invasion and clinical stage of the disease.
KRAS	3845	23613396	K-RAS and B-RAF mutations do not seem to be predictive of treatment outcome as potential biomarkers for bevacizumab therapy in metastatic colorectal cancer.
KRAS	3845	23625655	findings demonstrate that the presence of a K-ras mutation correlated with high initial CEA and CA 19-9 levels in patients with metastatic colorectal cancer
KRAS	3845	23790176	Taiwanese patients with metastatic colorectal cancer respond better to a cetuximab plus chemotherapy regime if their tumors have the wild-type KRAS gene
KRAS	3845	23975858	There was a negative correlation between KRAS codon 12 mutations and the level of HLA class I antigens in primary and metastatic tumors.
KRAS	3845	23999427	Stem cells with Kras(G12D) activation and Smad4 depletion can produce tumors that are multipotent and susceptible to epithelial mesenchymal transformation and metastasis.
KRAS	3845	24024839	RAS mutations, in addition to KRAS exon 2 mutations, predict a lack of response to anti-EGFR therapy in patients with metastatic colorectal cancer.
KRAS	3845	24244261	The combination of lenalidomide and cetuximab appeared to be well tolerated but did not have clinically meaningful activity in KRAS-mutant metastatic colorectal cancer patients.
KRAS	3845	24407191	As second- or later-line treatment of patients with KRAS-mutated metastatic colorectal cancer.
KRAS	3845	24491301	K-Ras mutation activates CSCs, contributing to colorectal tumorigenesis and metastasis in CRC cells harboring APC mutations. Initial activation of beta-catenin by APC loss and further enhancement through K-Ras mutation induces CD44, CD133, CD166 expression.

LAMA4	3910	16146715	laminin isoform changes are associated with brain tumor invasion and angiogenesis [review]
LAMA4	3910	17533363	alpha4 chain Lms have a de-adhesive function and could thus play a role in detachment, migration and invasion of renal carcinoma cells in vivo.
LAMA4	3910	18496706	Concomitant changes take place in laminin- and collagen-binding receptors. Laminin-411 reduces adhesion to laminin-511 and fibronectin, suggesting that tumor cells could utilize laminin-411 in their invasive behavior
LAMA4	3910	19048114	LM alpha4 and beta2 have roles in in vitro migration and in vivo tumorigenicity of prostate cancer cells
MAF	4094	18058096	After 32-50 weekly administrations of 100 ng GcMAF, all colorectal cancer patients exhibited healthy control levels of the serum Nagalase activity, indicating eradication of metastatic tumor cells.
MAP3K4	4216	24036211	MTK1 was identified in the HER2/HER3-HRG mediated extracellular acidification and cell migration pathway in breast cancer cells.
MICB	4277	24173243	Poorly differentiated tumors showed high MICA/B expression, which was related to extended tumor lymph node metastases and less frequent long-term survival.
MKI67	4288	11744991	Not significantly correlated with lymph node metastasis of breast invasive ductal carcinoma.
MKI67	4288	14674120	compared the expression of Ki-67 between primary breast tumors and metastasis to regional lymph nodes; estimated the relationships between Ki-67 and the anatomoclinical features of the breast cancer
MKI67	4288	17065089	analysis of Ki67 and thymidylate synthase expression in primary tumour compared with metastatic nodes in breast cancer patients
MKI67	4288	17647192	findings suggest Ki67 expression as a prognostic marker for lymph node metastasis in penile squamous carcinoma
MKI67	4288	18561741	Expression of Ki-67 in cervical intraepithelial neoplasia and invasive squamous cell carcinoma of the uterine cervix
MKI67	4288	18798062	Ki-67 plays a role in breast carcinoma cell proliferation & invasion. Expression of Ki-67 is essential for cell growth & tumorigenicity.
MKI67	4288	19080002	Overexpression of Bmi-1 and Ki67 protein are significantly correlated with tumorigenesis, metastasis and prognosis of colorectal carcinoma.
MKI67	4288	19690767	The main objectives were to assess tumor proliferation and invasiveness biomarkers (Ki-67, E-cadherin) and to identify potential correlation between biomarkers and classic prognostic factors in cervical cancer.
MKI67	4288	19788614	Endothelial proliferation assessed on Ki-67 combined with the lack of CD105 expression is often associated with invasive cervical carcinoma.
MKI67	4288	20338617	Increased expression of Ki-67 was associated with the presence of lymph node metastasis, advanced stages of disease, tumors occurring in the floor of mouth, and moderately/well-differentiated oral squamous cell carcinoma.
MKI67	4288	20483025	A review of literature is carried out to correlate the role of Ki-67 labeling index and its correlation with clinical findings, tumor size, invasiveness, recurrence, adenoma subtype, adenoma doubling time, and pituitary carcinomas is addressed.
MKI67	4288	20848772	The positive intensity of Ki67 protein expression in primary ovarian cancers was significantly higher than in metastatic ovarian cancers.
MKI67	4288	21655651	The aim of this work is to study the distribution of CD105 positive vessels and the proliferation rate of endothelial cells from liver metastasis of colon carcinoma based on double immunostaining CD105/Ki67.
MKI67	4288	21655654	The prognostic value of the correlations between the expression of estrogen and progesterone receptors, Ki67 expression and the histological stage, tumor stage, the degree of myometrial and vascular invasion, was investigated.
MKI67	4288	21809665	In grade 3 samples of CIN (Cervixal Intreapithelial Neoplasia), expression of Ki-67, separately or combined with thymidine phosphorylase, seemed to be related to

			progression to invasive cancer. Ki-67 levels were shown to correlate directly with CIN grade.
MKI67	4288	22452996	HIF-2a was correlated with ABCG2 expression, histology-grade and Ki67 expression in breast invasive ductal carcinoma
MKI67	4288	22542077	The expression of MIB-1 was statistically elevated in higher-grade osteosarcoma patients 25 years or younger and in tumors located in extremities and metastatic lesions.
MKI67	4288	22890751	High Ki67 expression in the primary tumor is an independent adverse prognostic factor in metastatic breast cancer.
MKI67	4288	22995634	Tumor sizes and high Ki67 PI in the invasive front appear to be important prognostic tools for head and neck squamous cell carcinoma.
MKI67	4288	23235554	Ki67 overexpression is associated with central nervous system metastases in operable breast cancer.
MKI67	4288	23818346	Immunohistochemical expression of Ki67 was not associated with characteristics of invasive breast cancers.
MKI67	4288	24360660	Data indicate that Ki-67 positivity is prognostic for predicting non-muscle invasive bladder cancer (NMIBC) recurrence and progression.
MKI67	4288	25211928	an association between the vimentin and ki-67 expression levels and the degree of spread of metastatic renal cell carcinoma was identified: the high level of expression of these markers precludes the increase of life expectancy
MMP2	4313	11168762	involvement of MMPs in microinvasive carcinomas
MMP2	4313	11854622	in pancreatic cancer, invasion into large veins and destroyed type veins could be a risk factor for liver metastasis and that increased expression MMP-2 and MMP-9 were related to such invasion.
MMP2	4313	11956628	expression level of MMP-2 mRNA may regulate with invasion ability of cervical cancer
MMP2	4313	12393872	Autocrine/paracrine prostaglandin E2 production by non-small cell lung cancer cells regulates enzyme and cd44 in cox-2-dependent invasion
MMP2	4313	12451991	The in vitro invasive ability of ovarian tumor cells appeared to be positive correlated to high expression of VEGF and MMP-2.
MMP2	4313	12479097	The over-expression of MMP-2, MMP-9, TIMP-1, and TIMP-2 may play a key role in invasion and lymph-node metastasis of in squamous carcinoma of the cervix.
MMP2	4313	12602913	MMP-2 is associated with tumor size, invasiveness and survival in breast cancer.
MMP2	4313	12632066	MMP2 is regulated by IL-8 in invasive pancreatic cancer
MMP2	4313	12684625	Increased expression of MMP-2 in advanced stage and high grade renal cell carcinoma might be associated with tumor invasion and metastasis
MMP2	4313	12845641	Increased in situ mmp-2 activity in renal cell tumor tissues correlates with tumor size, grade and vessel invasion
MMP2	4313	12861074	plays a critical role in inducing tumor cell infiltration, and this invasive phenotype is caused by activation of MMP-2
MMP2	4313	14519487	results suggest that matrix metalloproteinase 2 plays an important role in tumor invasion and metastasis
MMP2	4313	14614057	MMP-2 immunostaining was positive in 4 of 8 cases of invasive carcinoma and stained stromal area around tumor nest. Matrix metalloproteinases are activated in carcinoma in situ and in cervical intraepithelial neoplasia of uterine cervix.
MMP2	4313	14644155	findings established the existence of a novel alternative/complementary pathway by which furin increases tumor cell invasion through an amplification/activation loop between MMP-2 and TGFbeta
MMP2	4313	14713104	one of the mechanisms by which S100A4 may exert its effect on metastasis of some tumors is by regulating the MMP-2 activity.
MMP2	4313	14766248	Stromal MMP-2 occurs early and may play a role early in ovarian adenocarcinoma invasion.

MMP2	4313	14981939	Various growth factors induce migration of human non-small cell lung cancer cells in the presence of extracellular matrix (ECM) components MMP-2 and MMP-9.
MMP2	4313	14990972	Matrix metalloproteinase-2 may play a role in the pathogenesis, invasion, and metastasis of cutaneous squamous cell carcinoma.
MMP2	4313	15067014	MMP-2 or MMP-9 mediated tumor cell invasion requires integrin cytoplasmic-tail motif EKQKVDLSTDC
MMP2	4313	15254682	EGFR, c-erbB-2, VEGF and MMP-2 and MMP-9 play an important role in tumor growth, invasion and metastasis in squamous cell carcinoma of the head and neck
MMP2	4313	15485653	MMP-2 -1306 C-->T polymorphism may be associated with colorectal cancer development and invasion
MMP2	4313	15567754	MMP-2 plays an essential role in tumor invasion and metastasis, while TIMP-2 is shown to strongly inhibit cancer invasion and metastasis.
MMP2	4313	15609323	alphav integrins and MMP2 have roles in migration of human ovarian adenocarcinoma cells through endothelial extracellular matrix
MMP2	4313	15756447	E1AF positively regulates transcription from MT1-MMP genes, which plays an important role in invasion and metastasis of squamous cell carcinoma of the tongue by converting pro-MMP-2 into active-MMP-2
MMP2	4313	15820246	Hypoxia induces an increased invasive capacity via gelatinase up-regulation without loss of cell viability in adenocarcinomas
MMP2	4313	15929171	MMP-2 may play an important role in the development of invasion and metastasis of gastric cancer.
MMP2	4313	16134528	There was a significant correlation between active MMP-2 and the invasion and metastasis of epithelial ovarian cancer. Active MMP-2 is a prognostic indicator in patients with epithelial ovarian cancer.
MMP2	4313	16142392	MMP-2 may play an important role in the invasion and metastasis of oral squamous cell carcinoma.
MMP2	4313	16158251	The expression of MMP2 is consistently significantly higher in neoplastic brain tissue compared to normal brain tissue, and may be involved in the process of metastasis of breast cancer to the brain.
MMP2	4313	16407830	Both MAPKAPK2 and HSP27 are necessary for TGFbeta-mediated increases in MMP-2 and cell invasion in human prostate cancer.
MMP2	4313	16718824	MMP-2 expression has significant correlation with tumor invasion, tumor differentiation and lymph node metastases; MMP-2 may participate in the development of lymph node micrometastasis of gastric carcinoma
MMP2	4313	17029196	Data show that MMP2 may be relevant with carcinogenesis, development and metastasis of adenoid cystic carcinoma, and different metastasis potential may result from different subtype of MMPs gene family.
MMP2	4313	17203213	beta3 integrin in MDA-MB-231 breast cancer cells may lead to increased MMP-2 expression and activity and might contribute to the enhanced invasive potential observed
MMP2	4313	17545513	stromal fibroblasts play a role in promoting pancreatic cancer metastasis via activation of the uPA-plasminogen-MMP-2 cascade
MMP2	4313	17546628	Our results suggest that the presence of -1575G allele in the MMP-2 promoter region may be of significance in the assessment of colorectal cancer risk and invasive potential.
MMP2	4313	17570740	results suggest that MMP2 is a more sensitive predictor than MMP9 of lung cancer progression, metastasis, and survival
MMP2	4313	17706116	P-gp substrate drugs can greatly upregulate the expression of CD147 and MMP2 and 9 in multidrug resistant breast cancer cells, therefore enhancing the tumor metastatic capability.
MMP2	4313	17876296	ROCK-II is a critical mediator of colon cancer cell invasion through its modulation of MMP-2 and -13 at the site of invadopodia but regulates proliferation in

			non-malignant intestinal cells.
MMP2	4313	18000234	MMP-2 upregulation confers on tumor cells the ability to degrade the subepithelial basement membrane and subsequently invade the cervix.
MMP2	4313	18042068	MMP-2, MMP-9, TIMP-1 and TIMP-2 play an important role in the pathogenesis of non-melanoma skin cancer. The immunoexpression of these proteins may be useful indicators of cutaneous cancer invasion and progression.
MMP2	4313	18172354	The expression level of Matrix Metalloproteinase 2 mRNA showed an increasing trend according to differentiation, lymphatic invasion and Dukes' stage in colon cancer tissues.
MMP2	4313	18214299	Data show that enhanced expression of matrix metalloproteinases 2 was detected in colorectal cancer patients with distant metastases.
MMP2	4313	18265895	Crucial participants in tumour invasion and metastases are matrix metalloproteinases, tissue inhibitor of metalloproteinase inhibitors and cellular adhesion molecules. They play roles in tumour invasion and metastasis in non-small-cell lung carcinomas.
MMP2	4313	18288638	Data suggest that Fra-1 enhances lung cancer epithelial cell motility and invasion by inducing the activity of matrix metalloproteinases, in particular MMP-2 and MMP-9, and EGFR-activated signaling.
MMP2	4313	18330957	Metastatic gastric cancer presents more aggressive histological morphology and higher MMP-2 immunoreactivity than primary gastric cancer.
MMP2	4313	18337444	Knockdown of MMP-2 did not significantly alter the metastatic potential of the liver cancer cells but lead to a marked inhibition of metastatic foci growth.
MMP2	4313	18340378	MMP-2 expressed by metastatic ovarian cancer cells functionally regulates their attachment to peritoneal surfaces
MMP2	4313	18431027	soluble EMMPRIN probably triggers the promotion of cancer invasion in vivo
MMP2	4313	18483282	Rap1GAP inhibits tumor growth but induces MMP2- and MMP9-mediated SCC invasion and tumor progression, suggesting a role for this protein as a biomarker for early N-stage, aggressive SCCs
MMP2	4313	18644390	Data suggest that KiSS-1 suppresses the motility and invasive ability of renal cell carcinoma cells which possess hOT7T175 with either a negative expression or very low expression level of KiSS-1 through, at least in part, the down-regulation of MMP-2.
MMP2	4313	18652765	Results describe the relationship between protein expression of VEGF-C, MMP-2 and lymph node metastasis in papillary thyroid cancer.
MMP2	4313	18665467	Pathologic findings demonstrated that the intensity of MMP2 staining in cancerous tissues was associated significantly with histological types, distant metastasis, and Nevin stages of gallbladder carcinomas.
MMP2	4313	18707587	Data suggest that alpha5beta1 intergrin controls invasion ability of MCF-7/ADR breast carcinoma cells by regulating expression of MMP-2.
MMP2	4313	18714566	This study corroborates that stromal cells play an important role in tumor invasion and progression, mediated by the progressive enhancement of MMP-2 expression from CIN3 to advanced invasive tumor.
MMP2	4313	18757369	a link between RhoA, JNK, c-Jun, and MMP2 activity that is functionally involved in the reduction in osteosarcoma cell invasion by the statin. This suggests a novel strategy targeting RhoA-JNK-c-Jun signaling to reduce osteosarcoma cell tumorigenesis.
MMP2	4313	18946196	Ganoderic acid Me inhibits tumor invasion through down-regulating matrix metalloproteinases 2/9 gene expression.
MMP2	4313	18950008	COX-2 and MMP-2 expression was increased in nasopharyngeal carcinoma, and was positively correlated with lymph node metastasis.
MMP2	4313	18974156	results suggest that MMP-2 activation by MT1-MMP potentially amplifies protease activity, and combination with direct cleavage of substrate causes tissue

			degradation and enhances tumor invasion and metastasis, which highlights the complex role of TIMP-2
MMP2	4313	19088827	The positive expression of MMP-2 can be used to estimate the metastatic potential of cervical squamous carcinoma.
MMP2	4313	19101987	Platelet-derived microparticles promote prostate tumor invasiveness, at least in part by stimulation of MMP-2 production
MMP2	4313	19105397	Matrix metalloproteinase 2 and 9 as the factor of head and neck tumor metastasis
MMP2	4313	19116140	The role of MMP-2 and the signaling pathway involved in EGF-promoted invasion by human pancreatic cancer cells PANC-1 was investigated.
MMP2	4313	19191857	Expression of MMP-2 in esophageal squamous cell carcinomas was higher than in eophageal epithelium, and higher in the advanced stage; MMP-2 plays an important role in tumor invasion and metastasis of ESCCs.
MMP2	4313	19221481	The proteolytically active matrix-metalloproteinase (MMP)-2 is upregulated and as an early regulator of ovarian cancer metastasis.
MMP2	4313	19241124	selective COX-2 inhibition suppresses the invasion activity of OSCC cells via down-regulation of an MMP-2-activating mechanism involving TIMP-2 and production of the MMP-2 protein by an interaction between cancer cells and stromal fibroblasts
MMP2	4313	19286653	Low Levels of MMP-2 due to epigenetic silencing is associated with invasiveness in cancer.
MMP2	4313	19477063	curcumin has anti-metastatic potential resulting in inhibition of MMP-2 and -9 in human lung cancer A549 cells.
MMP2	4313	19528914	MMP2 was found in epidermal and dermal ests of maligant cells and in atypical melanocytes along the hair follicles in lentigo maligna melanoma, indicating its involvement in tumor invasion.
MMP2	4313	19529860	High expression of matrix metalloproteinase-2 was less significant for the invasive potential and prognosis of colorectal tumors.
MMP2	4313	19577357	Van Gogh-Like 2 regulates tumor cell migration and matrix metalloproteinase-dependent invasion.
MMP2	4313	19610062	High expression of MMP-2 is associated with malignant and invasive skin squamous cell carcinoma growth.
MMP2	4313	19625121	Up-regulation of MMP-2 and MMP-9 is one of the mechanisms by which Skp2 promotes lung cancer cell invasion.
MMP2	4313	19901964	Data show that P-cadherin overexpression in breast cancer cells with wild-type E-cadherin promotes cell invasion, motility, and induces the secretion of MMP-1 and MMP-2, which then lead to P-cadherin ectodomain cleavage.
MMP2	4313	19930715	results suggest that PRL-3's roles in motility, invasion, and metastasis in colon cancer are critically controlled by the integrin beta1-ERK1/2-MMP2 signaling
MMP2	4313	19956847	ICAM-3 enhances the migratory and invasive potential of human non-small cell lung cancer cells by inducing MMP-2 and MMP-9 via Akt and CREB
MMP2	4313	20372784	emodin, aloe-emodin and rhein inhibit the protein levels and activities of matrix MMP-2 but not gene expression of MMP-2; they do inhibit gene expression of MMP-9 and migration and invasion of human tongue cancer SCC-4 cells
MMP2	4313	20514447	TGF-beta, leading the tissue invasion mediated by MMP-2, is a strong promoter of tumor progression.
MMP2	4313	20600798	MMPs are critical targets of diallyl disulfide-induced anti-invasiveness in human prostate cancer LNCaP cells.
MMP2	4313	20646497	High expression of MMP-2 and IGF-1 proteins is significantly correlated with the invasion and metastasis of gastric carcinoma.
MMP2	4313	20646600	CD147 and MMP-2 play a role in invasion and metastasis of laryngeal carcinoma. Increased levels of MMP-2 and MMP-9 are induced by CD147 in laryngeal tumor cells.
MMP2	4313	20654122	MMP-2 and MMP-9 in conjunction with VEGF-C, promote lymphangiogenesis and lymph node metastasis of breast cancer.

MMP2	4313	20843706	metalloproteinase MMP-2 and MMP-9 -to-E-cadherin ratio has an effect on lymphangiogenesis and lymph node metastasis in prostate cancer
MMP2	4313	20970160	results indicate concurrent mechanisms in expression of MMP-2 and -9, RUNX1/AML1 and ETV5/ERM, and several basement membrane components, which likely associate with the invasive stage of endometrioid endometrial and ovarian endometrioid carcinoma.
MMP2	4313	21045016	Nicotinamide N-methyltransferase has a crucial role in cellular invasion via activating PI3K/Akt/SP1/MMP-2 pathway in clear cell renal cell carcinoma (ccRCC).
MMP2	4313	21104439	OPN enhanced hepatocellular carcinoma cells invasion through interaction with its receptor CD44v6 and increased MMP-2 and uPA expressions.
MMP2	4313	21104440	These findings show that CK19 and MMP-2 expression may be beneficial in predicting lymph node metastasis in hepatocellular carcinoma and survival.
MMP2	4313	21193558	results suggest GnRH-II-induced laminin receptor precursor expression increases 67-kDa nonintegrin laminin receptor, which appears to interact with laminin in the extracellular matrix to promote MMP-2 expression and enhance ovarian cancer cell invasion
MMP2	4313	21248144	The effect of estradiol on prostate cancer cell invasiveness via upregulation of MMP2 via a paracrine mechanism is reported.
MMP2	4313	21316839	The incidence of BAG3 positivity was significantly higher at advanced clinical stages of ovarian cancer than at early stages. It is suggected BAG3 binds to MMP2 to positively regulate the process of cell invasion.
MMP2	4313	21393869	Overexpression of MMP-2 and MMP-9 are often associated with increased invasive metastatic potential of colorectal adenocarcinoma.
MMP2	4313	21461859	data demonstrate, for the first time, that DDR1 mediates MMP-2 and -9 secretions and invasion induced by native type IV collagen in MDA-MB-231 breast cancer cells
MMP2	4313	21503582	Gallic acid suppresses the migration and invasion of PC-3 human prostate cancer cells via inhibition of matrix metalloproteinase-2 and -9 signaling pathways
MMP2	4313	21575462	Expressions of LOX and MMP-2 in gastric cancer tissues are significantly higher than that in pericancerous tissues, and are also higher in gastric cancer with lymph node metastasis.
MMP2	4313	21612407	The allele frequency of MMP2 rs243865 was not associated with the development of gastric cancer or its lymph node metastases. Epistasis between PARP1 rs1136410 & MMP2 rs243865 increased the risk of LNM of GC.
MMP2	4313	21627988	HABP1 enhanced melanoma cell migration, leading to MMP-2 activation and tumor growth.
MMP2	4313	21637818	Fluid shear stress in the tumor microenvironment may explain reduced glioma invasion through modulation of cell motility and MMP levels.
MMP2	4313	21687932	Results show that SLPI promotes the metastasis of SNU638 gastric cancer cells by increasing MMP-2 and MMP-9 expression through Elk-1 signaling, indicating its role as a signaling molecule not a protease inhibitor.
MMP2	4313	21842128	overexpression of serum response factor in hepatocellular carcinoma may play an important role in tumor cell migration and invasion through upregulation of matrix metalloproteinase-2 and matrix metalloproteinase-9
MMP2	4313	21901248	fibulin-3 negatively modulates the invasiveness of lung cancer cells via regulation of MMP-7 and MMP-2 and its expression is regulated by hypermethylation of the promoter region
MMP2	4313	21924036	CD147 and MMP-2 expression is correlated with the invasion and metastasis of squamous cell carcinoma and adenocarcinoma of the lungs
MMP2	4313	21968416	Matrix metalloproteinase 2 promotes cell growth and invasion in colorectal cancer.(
MMP2	4313	21989104	Taken together, our results suggest that the mechanism of QSOX1-mediated tumor cell invasion is by activation of MMP-2 and MMP-9.

MMP2	4313	22030137	The role of MMP-2 expression in the prognosis of invasive ductal carcinoma of the breast is rather limited.
MMP2	4313	22200661	ADAM17 contributes to androgen-independent prostate cancer cell invasion by shedding of EGFR ligand TGF-alpha, which subsequently activates the EGFR-MEK-ERK signaling pathway, leading finally to overexpression of MMP-2 and MMP-9.
MMP2	4313	22289521	These findings suggest that corticotropin-releasing hormone might induce invasion and migration by upregulating MMP-2 and MMP-9 in endometrial cancer.
MMP2	4313	22396544	Leukotriene B4 receptor-2 promotes invasiveness and metastasis of ovarian cancer cells through signal transducer and activator of transcription 3 (STAT3)-dependent up-regulation of matrix metalloproteinase 2
MMP2	4313	22519916	These results suggested that the inhibition on proMMP-2 expression by Liquiritigenin may be through suppression on PI3K/Akt signaling pathway, which in turn led to the inhibition of lung adenocarcinoma A549 cells migration.
MMP2	4313	22522455	These data show that overexpression of Aurora-A contributes to the malignancy development of ESCC by enhancing tumor cell invasion as well as MMP-2 activity and expression
MMP2	4313	22534634	Enhanced stromal MMP-2 expression in in situ and invasive squamous cell carcinoma relative actinic keratosis suggests role in disease progression.
MMP2	4313	22652796	hypothesized that BMP2 promotes cancer metastasis by modulating MMP-2 secretion and activity through intracellular ROS regulation and ERK activation in pancreatic cancer
MMP2	4313	22694915	basal lamina type IV collagen alpha chains, matrix metalloproteinases-2 and -9 expressions are expressed in oral dysplasia and invasive carcinoma
MMP2	4313	22846578	This study shows that MZF-1 represses MMP-2 transcription and suggests that this repression may be linked to inhibition of human cervical cancer cell migration and metastasis.
MMP2	4313	22883193	LOX, MMP-2, and MMP-9 may have a synergistic role in promoting invasion and metastasis of breast cancer.
MMP2	4313	22883459	MMP-2, MMP-9 and TIMP-2, TIMP-1 may play an important role in the development, progression, invasion and metastasis of non-melanoma skin cancer.
MMP2	4313	22968857	This data suggests that ETV5 has a significant role in regulating MMP2 expression and therefore matrix resorption in human chondrosarcoma, and thus may be a targetable upstream effector of the metastatic cascade in this cancer.
MMP2	4313	23038275	SIRT1 is an important regulator of MMP2 expression, activity, and prostate cancer cell invasion.
MMP2	4313	23134156	Mere15 inhibits tumor growth via both pro-apoptotic and antimetastasis pathways by reducing MMP2/9 expression.
MMP2	4313	23280016	analysis revealed that MDR1 expression in lymph node was not correlated with MMP-2 expression in lymph node, number of metastasized lymph nodes and tumor size
MMP2	4313	23306155	Derlin-1 is overexpressed in non-small cell lung cancer and promotes invasion by EGFR-ERK-mediated up-regulation of MMP-2 and MMP-9.
MMP2	4313	23388341	The high expressions of Mig-7 and MMP-2 in gastric carcinoma tissues may have a synergistic promoting effect on VM formation. VM is closely associated with the invasion, metastasis and poor prognosis of gastric carcinoma.
MMP2	4313	23544610	the inhibitory effect of TSA on cancer invasion is mediated through the suppression of HDAC2 expression, and the reduction of MMP-2 and MMP-9 expression induced by HDAC2 may be involved in the anti-invasive effect of TSA
MMP2	4313	23566884	Tomatidine inhibits invasion of human lung adenocarcinoma cell A549 by reducing matrix metalloproteinases expression.

MMP2	4313	23568763	Daidzein, R- and S-equol inhibit the invasion of MDA-MB-231 human breast cancer cells in part via the down-regulation of MMP-2 expression, with equipotent effects observed for the parent isoflavone daidzein and the equol enantiomers.
MMP2	4313	23577575	Increased MMP2 expression was found in nasopharyngeal carcinoma. In lymph node metastatic NPC, NPC cells had a higher rate of MMP-2 expression in tumor metastases than in the primary tumor.
MMP2	4313	23685894	Up-regulation of both MMP-2 and CD44v6 is significantly correlated with the development of lateral cervical lymph node metastasis in patients with papillary thyroid carcinoma.
MMP2	4313	23696025	The PAK5-Egr1-MMP2 signaling pathway is a critical regulator of cell migration and invasion in lung cancer cells.
MMP2	4313	23705892	enzyme activity assays confirmed increased MMP-1, -2, and -3 activity in cancer myofibroblasts, and cancer cell migration assays indicated stimulation by MMP-1, -2, and -3 in cancer-associated myofibroblast media.
MMP2	4313	23725476	Suggest that miR-491 is involved in metastasis of hepatocellular carcinoma by blocking epithelial to mesenchymal transition and decreasing MMP2/9 levels.
MMP2	4313	23832737	S100A4 plays a crucial role in proliferation and migratory, invasive processes in human renal cell cancer by a mechanism involving activation of NF-kB-bcl-2 and NF-kB-MMP-2 pathway
MMP2	4313	23897811	HDAC10 suppresses expression of matrix metalloproteinase (MMP) 2 and 9 genes, which are known to be critical for cancer cell invasion and metastasis
MMP2	4313	23902685	Rab40b mediates trafficking of MMP2/9 during invadopodia formation and metastasis of breast cancer cells.
MMP2	4313	23915071	Our data indicate that parallel expression of MMP-2 epithelial/stromal cells and p53 may enhance cells invasion and metastasis in ovarian carcinoma.
MMP2	4313	23936390	miR-29c serves as a tumor metastasis suppressor, which suppresses lung cancer cell adhesion to ECM and metastasis by directly inhibiting integrin beta1 and MMP2 expression
MMP2	4313	24386189	POU5F1 enhances the invasiveness of cancer stem-like cells in lung adenocarcinoma by upregulation of MMP-2 expression.
MMP2	4313	24395652	MMP-2 and TIMP-2 might play a role in the process of colorectal cancer invasion and metastasis
MMP2	4313	24479681	the IL-1beta/p38/AP-1(c-fos)/MMP2 & MMP9 pathway play an important role in metastasis in gastric adenocarcinoma
MMP2	4313	24851607	Comparison of proMMP-2 and prognostic factors revealed a statistically significant correlation between proMMP-2 and age in patients with histologic grade I tumors for invasive ductal breast cancer.
MUC4	4585	14744777	Inhibition of MUC4 expression suppresses pancreatic tumor cell growth and metastasis
MUC4	4585	14744777	MUC4 protein is associated with the metastatic pancreatic cancer phenotype and plays a role in altered growth and behavioral properties of the tumor cell.
MUC4	4585	16475027	Overexpresssed in the progression and lymphatic metastasis of prostate cancer.
MUC4	4585	21889495	these data indicate that MUC4 plays a key role in proliferative and migrating properties of esophageal cancer cells as well as is a tumor growth promoter.
MUC4	4585	22614008	Cigarette smoke promotes metastasis of pancreatic cancer through alpha7nAChR-mediated MUC4 upregulation.
MUC4	4585	23152882	The expression of MUC4 as well as MUC1 in early gastric cancers is a useful marker to predict poor prognostic factors related with vessel invasion.
MUC4	4585	23860190	Our data suggest that MUC4 appears to be a sensitive and specific marker for differentiating between metastatic adenocarcinoma cells and reactive mesothelial cells in effusion fluids

MUC4	4585	24037917	our study reveals a novel role for MUC4 in suppressing EMT and suggests that the assessment of MUC4 may function as a prognostic biomarker and could be a potential therapeutic target for lung adenocarcinoma metastasis.
MUC4	4585	24047396	MUC4 was observed in both primary pancreatic ductal adenocarcinoma (PDAC) tumors and matched lymph node metastases with a high level of concordance, suggesting that MUC4 expression is retained following PDAC metastasis.
MUC4	4585	24374017	MUC4-induced nuclear translocation of beta-catenin is a novel mechanism for growth, metastasis and angiogenesis of pancreatic cancer.
MUC6	4588	16475027	Overexpresssed in the progression and lymphatic metastasis of prostate cancer.
MUC6	4588	16807756	Down-regulation of MUC6 may contribute to malignant transformation of gastric epithelial cells and underlie the molecular bases of growth, invasion, metastasis and differentiation of gastric carcinoma.
MUC6	4588	21851820	results suggest that MUC6 may inhibit invasion of tumor cells through the basement membrane of the pancreatic duct and slow the development of infiltrating carcinoma.
NCAM1	4684	16211277	NCAM is associated not only with a cell-to-cell adhesion mechanism, but also with tumorigenesis, including growth, development and perineural invasion in human salivary gland tumors
NCAM1	4684	17181871	Neuroblastoma cells resistant to anticancer drugs have increased invasive capacity caused by down-regulation of NCAM adhesion receptor.
NCAM1	4684	17982624	NCAM expression may be used as a predictor of perineural invasion in adenoid cystic carcinoma
NCAM1	4684	23015367	Bioinformatic analysis of NCAM-associated expression profiles predicted a highly interactive protein network, which further implies potential molecular mechanisms underlying the metastatic processes of thyroid cancer
NFYC	4802	22104449	NFY-C expression was elevated in colorectal adenocarcinomas; moreover, NFY-C mRNA levels correlated with time to disease progression, while NFY-C protein expression was significantly higher in metastatic disease
OPCML	4978	17109515	The results were confirmed at the level of mRNA and protein, and suggested that four genes (OPCML, RNASE1, YES1 and ACK1) could play a key role in the tumorigenesis and metastasis of gastric cancer.
PDGFRB	5159	16007172	alpha-PDGFR expression might identify prostate cancer cells with the highest propensity to metastasize to the skeleton
PDGFRB	5159	19636022	Phase I dose escalation study of telatinib, a tyrosine kinase inhibitor of vascular endothelial growth factor receptor 2 and 3, platelet-derived growth factor receptor beta, and c-Kit, in patients with advanced or metastatic solid tumors.
PDGFRB	5159	20624165	Data indicate that secretion of PDGF-B by gastric carcinoma cells and expression of PDGF-Rbeta by tumor-associated stromal cells are associated with lymphatic metastasis.
PDGFRB	5159	21118571	PDGF-B, which is involved in the maintenance of microvessels, plays an important role in angiogenesis in intestinal-type gastric carcinomas. Phosphorylation of PDGFR-beta was correlated with depth of cancer invasion.
PDGFRB	5159	24725405	Study combined several orthogonal approaches and models to explore the basis whereby mutant p53 promotes invasion and metastasis in pancreatic ductal adenocarcinoma (PDAC)and identified the PDGFRb as necessary and sufficient to mediate the effects of mutant p53 on invasion and metastasis in both a murine model and human PDAC cells.

PDGFRB	5159	24769756	Increased expression of platelet-derived growth factor receptor-beta in pancreatic cancer cells is mediated by DNA binding and structural mutants of p53 and that this contributes to metastasis
PIK3CA	5290	15950905	Amino acid substitutions in PIK3CA from human colorectal cancer cell lines constitutively activate the AKT pathway, and consequently, promote tumor cell growth and invasion.
PIK3CA	5290	16788758	Increased expression for PIK3CA mrna is associated with regional lymph node metastasis in esophageal squamous cell carcinoma
PIK3CA	5290	17062663	PIK3CA may serve as a marker of invasion in endometrial cancer.
PIK3CA	5290	17546593	Data suggest that PIK3CA mutations contribute to the invasion step from intramucosal carcinoma to invasive carcinoma in colorectal carcinogenesis.
PIK3CA	5290	18084252	PIK3CA mutations are associated with myometrial invasion and tumors with PIK3CA mutations in exon 20 are frequently high-grade, invasive endometrial carcinomas.
PIK3CA	5290	18183466	PIK3CA mutations are common in invasive ductal carcinomas of the breast and PIK3CA exon 20 mutation is an independent risk factor for poor prognosis in breast cancer patients.
PIK3CA	5290	18852163	strong genetic evidence supporting a role of the PI3K/AKT signaling pathway in the tumorigenesis of pituitary tumors, particularly the invasive types
PIK3CA	5290	18990689	ARF1 regulates epidermal growth factor-dependent breast cancer cell growth and invasion during cancer progression by controlling the activation of the phosphatidylinositol 3-kinase pathway
PIK3CA	5290	19366826	PIK3CA mutations are not a major determinant of resistance to the epidermal growth factor receptor inhibitor cetuximab in metastatic colorectal cancer.
PIK3CA	5290	19418217	complete concordance of PIK3CA and AKT1 mutations in matched samples of invasive and in situ tumor indicates that these mutations occur early in breast cancer development
PIK3CA	5290	19603024	PIK3CA (P=0.01) mutations predicted reduced progress free survival in response to cetuximab salvage therapy in patients with metastatic colorectal cancer .
PIK3CA	5290	20107891	PIK3CA mutations were detected in 13/46 (28%) plasma-derived and 10/46 (21%) serum-derived cfDNA samples from metastatic breast cancer patients.
PIK3CA	5290	20551053	Results suggest that mutation of PIK3CA is an early event in breast cancer that is more likely to play a role in breast tumor initiation than in invasive progression. A role for exon 9 mutations in the progression of a subset of DCIS cannot be excluded.
PIK3CA	5290	20619739	if KRAS is not mutated, assessing BRAF, NRAS, and PIK3CA exon 20 mutations (in that order) gives additional information about the efficacy of cetuximab plus chemotherapy in metastatic colorectal cancer.
PIK3CA	5290	20813970	PIK3CA mutation is associated with poor trastuzumab response in HER2-positive metastatic breast cancer.
PIK3CA	5290	20954287	Up-regulation of PIK3CA may promote the metastasis of gastric cancer through aberrant activation of PI3K/Akt.
PIK3CA	5290	21209852	the PI3K/PAK1/ERK signaling pathway has a role in LPA-stimulated breast cancer cell migration
PIK3CA	5290	21424126	data indicated that colorectal cancers with KRAS and PIK3CA bi-mutations are more likely to develop into liver metastasis
PIK3CA	5290	21490305	PIK3CA mutations were detected in 19 (40%) of primary tumors and 21 (42%) of metastases
PIK3CA	5290	21594665	PIK3CA activating mutations and and PTEN loss is associated with less efficacy of trastuzumab therapy in metastatic breast cancer
PIK3CA	5290	21617917	Demonstrate the presence of PIK3CA and AKT1(E17K) mutations from pre-invasive to invasive to metastatic breast cancer tissue.

PIK3CA	5290	21708979	PI3K signaling via p110alpha regulates invadopodia-mediated invasion of breast cancer cells.
PIK3CA	5290	22039088	PIK3CA exon 20 mutations may be a potential biomarker for resistance to anti-EGFR monoclonal antibodies in KRAS wild-type metastatic colorectal cancer. [Review and meta-analysis]
PIK3CA	5290	22285706	loss of PTEN expression (by immunohistochemistry) and PIK3CA mutation is likely to be predictive of a lack of benefit to anti-EGFR therapy in metastatic colorectal cancer
PIK3CA	5290	22330809	PIK3CA mutation was associated with significantly longer metastasis-free survival in breast cancer patients.
PIK3CA	5290	22460814	PIK3CA mutations were identified in 13/24 columnar cell lesions (54%) and 3/8 invasive carcinomas (37%).
PIK3CA	5290	22737248	analysis of multiorgan metastasis of human HER-2+ breast cancer in Rag2-/-;Il2rg-/- mice and treatment with PI3K inhibitor
PIK3CA	5290	22851869	PI3K expression and PIK3CA mutations are related to colorectal cancer metastases
PIK3CA	5290	22870241	High-throughput genotyping in metastatic esophageal squamous cell carcinoma identifies phosphoinositide-3-kinase and BRAF mutations.
PIK3CA	5290	22994622	Data indicate that copy number amplification of the PIK3CA gene is associated with poor prognosis in head and neck squamous cell carcinoma (HNSCC) patients without lymph node metastasis.
PIK3CA	5290	23352210	The rate of PIK3CA mutations in this series of micropapillary carcinomas is similar to invasive ductal carcinomas; however, there may be an enrichment of AKT1 mutations.
PIK3CA	5290	23615713	Results show that stromal cell-derived factor-1 (SDF-1) enhanced ovarian cancer cell invasion through alphavbeta6 integrin-mediated urokinase-type plasminogen activator (uPA) expression via the p38 MAPK and PI3 K/Akt pathway.
PIK3CA	5290	23884447	Among 30 breast tumors, 10(33%) had BCSCs with AKT1, HRAS, or PIK3CA mutations. 3 mutations were detected in PIK3CA.Tumors in which BCSCs have defects in PI3K/Akt signaling are significantly more likely to manifest nodal metastases.
PIK3CA	5290	24320611	The Pik3ca(H1047R) mutation promotes the development of highly aggressive and invasive adenocarcinomas in intestine.
PIK3CA	5290	24328409	Low expression of miR-1 and high expression of PIK3CA are closely related to the invasion, progression and metastasis of non-small-cell lung cancer.There is a negative correlation between the expression of miR-1 and PIK3CA in non-small-cell lung cancer.
PIN1	5300	19885590	overexpression of Pin-1 and VEGF-C may promote tumor progression and metastasis
PIN1	5300	19920136	the importance of Pin1 in TGF-beta-induced migration and invasion of cancer cells
PIN1	5300	20009523	High expression of Pin1 correlated with poor survivial in NSCLC patients. Overexpression of Pin1 in Glc82 cells increased cell growth and colony formation and tumorigenicity in nude mice including cell migration, invasion.
PKD1	5310	18283041	propose that PKD1 is frequently silenced by epigenetic regulation, which plays a role in cell migration and metastasis in gastric cancer
PLEC	5339	18084872	results reveal that plectin is up-regulated in colorectal adenocarcinoma as well as in bizarre glands and locally invasive tumor nests in tubular adenoma, compared with normal colorectal mucosa
PLEC	5339	21821021	plectin participates in actin assembly and invasiveness in carcinoma cells in an isoform-specific manner
PLEC	5339	22245045	findings suggest that plectin promotes the migration and invasion of head and neck squamous cell carcinoma (HNSCC) cells through activation of Erk 1/2 kinase

			and is a potential prognostic biomarker of HNSCC.
PRSS3	5646	20947888	PRSS3 plays an important role in the progression, metastasis and prognosis of human pancreatic cancer.
PTCH1	5727	22407314	Expressions of Shh, Ptch1 and Gli1 were significantly correlated with stage, lymph node metastasis, venous invasion, hepatic infiltration, survival rate, and lymphatic invasion in gallbladder carcinoma.
PTCH1	5727	24335643	Case Report: identical PTCH1 mutations in epithelial and sarcomatoid primary tumor components and sarcomatoid metastasis of basal cell carcinosarcoma.
PTCH1	5727	24961235	MiR-212 may facilitate pancreatic ductal adenocarcinoma progression and metastasis through targeting PTCH1.
PTEN	5728	11896207	Abnormalities of the PTEN gene are associated with tumor progression, metastasis, and survival.
PTEN	5728	12687015	overexpression of RRM1 in human and mouse lung cancer cell lines induced PTEN expression, reduced phosphorylation of focal adhesion kinase (FAK), suppressed migration, invasion, and metastasis formation, and increased survival in an animal model.
PTEN	5728	12767263	Data suggest that PTEN tumor suppressor gene malfunction seems to be involved in metastasing capacity of hepatocellular carcinoma.
PTEN	5728	14674989	Loss of PTEN expression is common and correlates with tumor progression and lymph node metastasis in breast carcinoma.
PTEN	5728	14976311	results show that PTEN can inhibit cell migration through its C2 domain, independent of its lipid phosphatase activity; ability of PTEN to control cell migration through its C2 domain is likely to be an important feature of its tumor suppressor activity
PTEN	5728	15129904	a role of these tumor suppressor and metastasis suppressor genes in the evolution and progression of NSCLC
PTEN	5728	15551732	reduced PTEN expression may be an independent prognostic indicator in patients with invasive ductal carcinoma.
PTEN	5728	15633233	PTEN mutations are restricted to advanced gastric cancer, loss of heterozygosity and mutation of PTEN gene are closely related to the infiltration and metastasis of gastric cancer.
PTEN	5728	16020969	Reduced expression of PTEN protein correlated with lymph node metastases in the patients with breast cancer
PTEN	5728	16739377	Hypermethylation of PTEN gene promoter is associated with loss of its transcription in laryngeal squamous cell carcinoma, and may be related to lymph node metastasis.
PTEN	5728	17347137	loss of PTEN function in human prostate cancer may specifically facilitate bone rather than other organ metastasis and suggest that Rac1, as a PTEN effector, may contribute to this metastatic tropism.
PTEN	5728	17441421	The protein expression of PTEN was correlated with clinical stage, tumor differentiation grade, and cervical lymph node metastasis in laryngeal squamous cell carcinoma
PTEN	5728	17452630	aberrant PI3K pathway signaling is strongly associated with metastasis and poor survival across carcinoma types
PTEN	5728	17593340	Metastasis of renal cell carcinoma is correlated with inactivation of PTEN.
PTEN	5728	17669465	Increased promoter methylation of PTEN was present in renal metastasis (of breast cancer), coinciding with the decrease in the level of normal PTEN transcript
PTEN	5728	17880912	PTEN plays a critical role in MAGI-2-induced inhibition of cell migration and proliferation in human hepatocarcinoma cells
PTEN	5728	18239055	In pancreatic tumor cells calcium-dependent PKC-alpha mediates TGF-beta-induced transcriptional downregulation of PTEN, and this pathway promotes cell migration in a SMAD4-null environment.

PTEN	5728	18383208	Increased PTEN expression was associated with invasive adenocarcinoma of the prostate
PTEN	5728	18720079	The expression of metastasis inhibitor genes PTEN and thrombospondin 2 was down-regulated in the supraglottic carcinoma tissue with lymph node metastasis.
PTEN	5728	19036165	Loss of PTEN expression is associated with colorectal cancer liver metastasis and poor patient survival.
PTEN	5728	19261747	combined deletion of p53 and Pten in bladder epithelium leads to invasive cancer in a novel mouse model. Inactivation of p53 and PTEN promotes tumorigenesis in human bladder cells and is correlated with poor survival in human tumors.
PTEN	5728	19560785	The IGF-1/PI3K/PTEN/Akt/NF-small ka, CyrillicB cascade may be a key pathway stimulating metastasis of pancreatic cancer cells.
PTEN	5728	19940030	The TGF-beta/NF-kappaB/PTEN cascade may be a critical pathway for pancreatic cancer cells to proliferate and metastasize
PTEN	5728	19962668	show that miR-221&222, by targeting PTEN and TIMP3 tumor suppressors, induce TRAIL resistance and enhance cellular migration through the activation of the AKT pathway and metallopeptidases
PTEN	5728	20032390	Study supports clinical studies identifying an association of PTEN loss with late stage cancer. Cellular factors secreted from the surrounding tumor milieu act in concert with genetic changes in the tumor cells and contribute to enhanced tumor invasion.
PTEN	5728	20226014	Data show that the coexpression of HSP90 with PI3K-p110alpha or expression of HSP90 along with PTEN loss has a potential as a molecular prognostic marker to predict early relapse in patients with invasive breast cancers.
PTEN	5728	20407866	PTEN gene was significantly linked with the progression and metastasis of gastrointestinal stromal tumors.
PTEN	5728	20813970	PTEN loss is associated with poor trastuzumab response in HER2-positive metastatic breast cancer.
PTEN	5728	21029699	The overexpression of bFGF and underexpression of PTEN are closely related to the invasion and growth of cervical carcinoma.
PTEN	5728	21104017	Data show that MiR-21 was overexpressed in vitro and esophageal squamous cell carcinoma, and promoted the cell proliferation, might target PTEN at post-transcriptional level, and regulated the cancer invasion in Kazakh's ESCC.
PTEN	5728	21151099	The combined deregulation of the pVHL/HIF axis and PTEN alone appear to be important for multilocular cystic renal cell carcinoma development but not sufficient to drive metastatic processes.
PTEN	5728	21155011	Data show that KRAS mutation and PTEN protein expression are significantly correlated with the response rate and survival time of Chinese metastatic colorectal cancer patients treated with cetuximab.
PTEN	5728	21203412	PTEN controls cellular polarity, establishment of cell-cell junctions, paracellular permeability, migration and tumorigenic/metastatic potential of human colorectal cancer cells
PTEN	5728	21206972	Migration of renal tumor cells depends on dephosphorylation of Shc by PTEN.
PTEN	5728	21333374	augments invasiveness and growth of lung cancer cells through the PI3K/AKT/NFkB pathway
PTEN	5728	21490305	PTEN was lost in 14 (30%) primary tumors and 13 (25%) metastases
PTEN	5728	21575512	PTEN may suppress the invasive and migration ability of the ovarian cancer cell line A2780, possibly by downregulating MMP9.
PTEN	5728	21685691	MAGI1 may inhibit the cancer cell migration and invasion of hepatocellular carcinoma via regulating PTEN.
PTEN	5728	21696297	Metastases in most subcellular compartments showed comparable and stronger expression for pAKT, pmTOR, and PTEN than primary renal cell carcinomas

			(PRCCs).
PTEN	5728	21881486	PTEN loss seems to be an early event during tumorigenesis in primary and metastatic clear cell renal cell carcinomas.
PTEN	5728	22036936	results suggest that a high level of PTEN expression is associated with low-grade liver metastasis and satisfactory patient survival in pancreatic cancer
PTEN	5728	22240798	Loss of PTEN expression was present in 59 out of 273 carcinomas and was significantly correlated to genomic PTEN deletion, high-grade malignancy, increased tumour size (P=0.036), lymph node metastases (P=0.007) and worse disease-specific survival
PTEN	5728	22267008	microRNA-21 promotes tumor proliferation and invasion in gastric cancer by targeting PTEN.
PTEN	5728	22279049	IGF2BP1 promotes the velocity and directionality of tumor-derived cell migration by determining the cytoplasmic fate of two novel target mRNAs: MAPK4 and PTEN
PTEN	5728	22285706	loss of PTEN expression (by immunohistochemistry) and PIK3CA mutation is likely to be predictive of a lack of benefit to anti-EGFR therapy in metastatic colorectal cancer
PTEN	5728	22350410	Pten loss and RAS/MAPK activation cooperate to promote EMT and metastasis initiated from prostate cancer stem/progenitor cells.
PTEN	5728	22375056	PTEN protein phosphatase activity correlates with control of gene expression and invasion, a tumor-suppressing phenotype, but not with AKT activity.
PTEN	5728	22429330	PTEN mRNA expression was significantly downregulated in brain metastases compared with primary breast tumors, and PTEN mutations were frequently found in brain metastases.
PTEN	5728	22552802	This study concludes that the abnormal methylation of PTEN gene promoter CpG islands exists in the colorectal tumors and metastases.
PTEN	5728	22639407	Genomic PTEN deletion is a rare event in gastric adenocarcinoma but correlates with metastatic disease. The homogeneous distribution pattern indicates that this alteration occurs early in tumour development.
PTEN	5728	22885155	Our study demonstrated that miR-26a enhanced lung cancer cell metastasis potential via modulation of metastasis-related gene expression, and activation of AKT pathway by PTEN suppression
PTEN	5728	23104175	these results suggest that Sp1 is involved in the development and invasiveness of cancer through inhibition of PTEN
PTEN	5728	23319441	analysis of PTEN status in 135 sporadic invasive ductal carcinomas reveals that relapsed tumors are associated with a specific, complex DNA profile at the PTEN locus with a strikingly similar highly rearranged pan-genomic profile
PTEN	5728	23574264	In HER2-positive recurrent or metastatic breast cancer patients PTEN loss might indicate resistance to trastuzumab-based salvage treatment.
PTEN	5728	23617834	MicroRNA-32 (miR-32) regulates phosphatase and tensin homologue (PTEN) expression and promotes growth, migration, and invasion in colorectal carcinoma cells.
PTEN	5728	23623571	Reactivation of PTEN tumor suppressor pathway leads to a 50% reduction in colorectal cancer metastasis without affecting primary tumor formation.
PTEN	5728	23670029	Patients with mtp53 tumors were older at diagnosis, had more incidence of liver metastasis, and more frequent PTEN loss.
PTEN	5728	24133589	Found progressive loss of PTEN and increase in EGFR, TGF-alpha, P-AKT expression from benign samples to non small cell lung cancer. Changes were correlated to differentiation extent of cancer tissue, metastasis of lymph nodes and histological classification.
PTEN	5728	24156022	PTEN expression is consistent in colorectal cancer primaries and metastases and associates with patient survival.
PTEN	5728	24286315	miR-221 may promote trastuzumab resistance and metastasis of HER2-positive breast cancers by targeting PTEN, suggesting its role as a potential biomarker for

			progression and poor prognosis
PTEN	5728	24532253	Lipopolysaccharide induced miR-181a promotes pancreatic cancer cell migration via targeting PTEN and MAP2K4.
PTEN	5728	24599953	conclude that cross-talk between PTEN and PHLPPs is silenced in normal prostate cells but activated in TGF-beta1 transformed prostate stem and cancer cells and facilitates invasive growth
PTEN	5728	24842611	cancer associated fibroblasts with down-regulated miR-106b could significantly inhibit gastric cancer cell migration and invasion by targeting PTEN
RET	5979	14586073	Point mutation in exon 14 at codon 804 of the RET proto-oncogene locus in a case of lymph node metastases of medullary and papillary thyroid carcinoma.
RET	5979	17270543	Children of families with RET cysteine mutations may develop early metastatic medullary carcinoma of the thyroid gland.
RET	5979	18316596	a beta-catenin-RET kinase pathway is a critical contributor to the development and metastasis of thyroid carcinoma
RET	5979	20801952	RET S836S variant is associated with early onset and increased risk for metastatic disease in patients with hereditary or sporadic hereditary or sporadic medullary thyroid carcinoma.
RET	5979	23868506	Authors show that elevated levels of Ret receptor are found in different sub-types of human breast cancers and that high Ret correlates with decreased metastasis-free survival.
RET	5979	24778213	These findings collectively demonstrate that GFRalpha1 released by nerves enhances perineural invasion through GDNF-RET signaling and that GFRalpha1 expression by cancer cells enhances but is not required for it.
ROBO1	6091	19706539	Data uncover a previously unknown function of USP33 and reveal a new player in Slit-Robo signaling in cancer cell migration.
ROBO1	6091	20300657	These results describe a Slit-miR-218-Robo1 regulatory circuit whose disruption may contribute to gastric cancer metastasis.
ROBO1	6091	20438712	these findings reveal that through interacting with Robo1, Slit2 is a novel and potent lymphangiogenic factor and contributes to tumor lymphatic metastasis.
ROBO1	6091	21465248	three major members (Slit2/3 and Robo1) of Slit/Robo family are widely expressed in the human normal and malignant ovarian tissues; but Slit/Robo signaling may not play an important role in regulating human ovarian cancer cell proliferation and migration
ROBO1	6091	21875486	Robo1 expression correlates negatively with invasive ductal carcinoma brain metastasis, and correlates positively with the age and prognosis of IDC patients.
ROBO1	6091	22898079	breast cancer cell migration and invasion was promoted when miRNA- 218 was significantly down regulated, in the way of up-regulation of Robo1.
ROBO1	6091	23953227	Low Robo1 expression was associated with cell proliferation and migration in ICC and was one of the adverse prognostic factors in patients with these tumors.
ROS1	6098	23549810	ROS1 expression is significantly lower in proportion to higher histologic grade, higher mitotic counts, lower estrogen receptor expression, and a higher Ki-67 proliferation index in invasive ductal carcinoma of the breast.
ROS1	6098	23810364	ROS1 gene rearrangements are associated with metastatic non--small-cell lung cancer.
RRAS	6237	12548599	the oncogene R-Ras promotes tumor growth of cervical epithelial cells and increases their migration potential over collagen through a pathway that involves PI 3-K.
SCN5A	6331	16061851	Up-regulation of neonatal Na(v)1.5 occurs as an integral part of the metastatic process in human breast cancer
SCN5A	6331	19224165	Nav1.5 functional expression potentiated the invasive behavior of human breast cancer cell line MDA-MB-231 by increasing the secretion of MMP-9.
SCN5A	6331	20372843	Abnormal expression of Nav1.5 could be an integral component of the metastatic process in human ovarian cancer.
SCN5A	6331	20651255	Data established a role of SCN5A as a regulator of a colon cancer invasion network, involving genes that encompass Wnt signaling, cell migration, ectoderm

			development, response to biotic stimulus, steroid metabolic process, and cell cycle control.
SCN5A	6331	21170089	Na(V)1.5 and NHE1 are functionally coupled and enhance the invasiveness of cancer cells by increasing H(+) efflux.
CCL24	6369	17908961	eotaxin-2 is a chemokine strongly associated with primary and metastatic tumors of colorectal origin
SMTN	6525	20636795	Smoothelin immunohistochemistry has diagnostic utility in the evaluation of muscularis propria invasion in urothelial carcinoma.
SOX2	6657	16596179	Increased SOX2 is associated with the pancreatobiliary phenotype of ampulla of vater carcinoma and involved in later events in carcinogenesis, such as invasion and metastasis
SOX2	6657	19554373	High SOX2 is correlated with lymph node metastasis in colorectal cancer.
SOX2	6657	22184289	Glioblastoma multiforme tissue grade IV specimens showed significant expression of Twist1 and Sox2, known mesenchymal and stemness related markers, respectively, indicating their association with glial tumor genesis and metastasis.
SOX2	6657	22236193	study reported that, in the univariate analysis, SOX2 expression was correlated with a poorer distant metastasis-free survival in nasopharyngeal carcinoma patients
SOX2	6657	23005595	The results indicate that high expression rates of SOX2 might be a prediction marker for oral squamous cell carcinoma lymph node metastasis.
SOX2	6657	23307254	Our data suggested for the first time that the high expression of Sox2 may contribute to the development of non-muscle-invasive bladder cancer
SOX2	6657	23414798	Data indicate that sex determining region Y (SRY)-box 2 (SOX2) up-regulation is frequent in early squamous cell carcinomas of the oral cavity and associated with decreased risk of lymphatic metastasis.
SOX2	6657	23430442	A high level of Sox2 expression correlates with metastasis and low survival rate of patients with hepatocellular carcinoma.
SOX2	6657	23815808	Sox2 regulates the invasiveness of breast cancer cells via a mechanism that is dependent on Twist1 and the transcriptional status of Sox2
SOX2	6657	23895273	The SOX2-FN1 axis is a key pathway in mediating the migration and invasion of ovarian cancer cells
SOX2	6657	24382260	SOX2 was detected in 19.0% (105 of 552) of invasive breast carcinomas and 12.3% (seven of 57) of DCIS. Expression correlated with larger tumour size (P = 0.005) and higher grade (P = 0.002).
ST14	6768	14584072	Cervical carcinoma cells expressed high levels of TADG-15, suggesting that this protease may play an important role in invasion and metastasis.
ST14	6768	14747469	matriptase is downregulated through suppression of activation of receptor-bound pro-urokinase, and leads to inhibition of tumor invasion
ST14	6768	16237759	SNC19/ST14 gene overexpression could enhance invasion of colorectal cancer cells in vitro significantly and influence early cell adherence to ECM, but could not change cell movement significantly.
ST14	6768	17456594	overexpression of MSP, MT-SP1, and MST1R was a strong independent indicator of both metastasis and death in human breast cancer
ST14	6768	18649735	Overexpression of bikunin reduced the gene expression of matriptase, which attenuated in vitro cell invasion. Different metastatic characteristics between PC-3 and PC-J cells suggest that matriptase plays a role in the metastasis of prostate cancer.
ST14	6768	20145119	MT-SP1 activity as a useful biomarker to visualize epithelial cancers using a noninvasive antibody-based method.
ST14	6768	20971737	ErbB-2 signaling via the phosphatidylinositol 3-kinase pathway results in up-regulated matriptase zymogen activity, which contributes to prostate cancer cell invasion.
ST14	6768	21097670	Epithin, which regulates endothelial Tie2 functions, plays a critical role in the fine tuning of transendothelial migration for normal and cancer cells.

ST14	6768	23248318	Emergent active matriptase is a functional biomarker of the transformed epithelium and that its proteolytic activity can be exploited to noninvasively evaluate tumorigenesis in vivo.
STAT1	6772	22178447	Stat1 and CD74 overexpression is co-dependent and linked to increased invasion and lymph node metastasis in triple-negative breast cancer.
STAT1	6772	22320867	expression of pSTAT-1 and its correlation with estrogen receptor, progesterone receptor, caspase-3, and pAkt and prognosis in premenopausal and postmenopausal patients with invasive breast cancer
STAT1	6772	23917355	our results suggest that CUG2 enhances metastasis and drug resistance through STAT1 activation, which eventually contributes to tumor progression
STAT6	6778	18294957	These findings, together with the observation of constitutive Stat6 activation in many human malignancies, suggest that Stat6 activities could be a biomarker for cancer cell's invasive/metastatic capability.
STAT6	6778	22108090	Stat6 is involved in a process that promotes and transforms cancerous cells to a more severe pro-growth, apoptosis-resistant and metastasis-capable phenotype.
TBXAS1	6916	15870920	Significantly higher expression of thromboxane synthase is associated with metastasis in non-small cell lung cancer
TBXAS1	6916	16357168	Overexpression of thromboxane synthase is associated with invasive bladder cancer
TGFB2	7042	15389580	PSA-mediated activation of latent TGFbeta2 may be an important mechanism for autocrine TGFbeta regulation in the prostate and may potentially contribute to the formation of osteoblastic lesions in bone metastatic prostate cancer.
TGFBR2	7048	12970754	We observed subsequent loss of type II TGFbeta receptor (TBR2) expression in metastatic renal cell carcinomas [RCC}. We propose that loss of TBR3 is necessary for RCC carcinogenesis, and loss of TBR2 leads to acquisition of a metastatic phenotype.
TGFBR2	7048	15976377	Repression of type II TGF-beta receptor may act as significant determinant of lung adenocarcinoma invasiveness, an early step in tumor progression toward metastasis.
TGFBR2	7048	19538865	Low expression of Smad4 and TbetaRII may promote metastasis of oral squamous cell carcinoma.
TGFBR2	7048	19624886	TGF-betaRII expression was related with depth of invasion, lymph node metastasis, tumor differentiation, and Lauren classification of gastric cancer.
TGFBR2	7048	20878063	The simultaneous expression of Endoglin (CD105), transforming growth factor (TGF)-beta1 and TGF-beta receptor (R) II were quantified in normal human colon, pre-malignant dysplastic tissue, and invasive colon cancer specimens
TGFBR2	7048	21454688	Restoration of transforming growth factor-beta receptor II expression in colon cancer cells with microsatellite instability increases metastatic potential in vivo
TGFBR2	7048	22278155	Data suggest that TGF-beta, TGF-betaR1, TGF-betaR2, Smad4, pSmad2/3, and E-cadherin are closely related to tumor-node-metastasis (TNM) stage of colorectal cancer (CRC).
TGFBR2	7048	22672900	Data indicate that abrogation of TGFbeta signaling through introduction of a dominant negative TGFbeta receptor II (TGFbetaRII) in non-metastatic FETalpha colon cancer cells permits metastasis to distal organs, but does not reduce invasive behavior.
TGFBR2	7048	22684895	miR-590-5p promotes proliferation and invasion in human hepatocellular carcinoma cells by directly targeting TGF-beta RII. [mir-590-5p]
TGFBR2	7048	22912877	Data indicate that MiR-106a inhibits the expression of transforming growth factor-beta receptor 2 (TGFBR2), leading to increased colorectal cancer (CRC) cell migration and invasion.
TGFBR2	7048	23220853	an association between TGF-betaRII-negative tumor and higher risk of metastasis to lungs and bones was verified.

TGFBR3	7049	17999987	type III transforming growth factor beta receptor has a role in preventing motility and invasiveness associated with epithelial to mesenchymal transition during pancreatic cancer progression
TGFBR3	7049	19416857	an unexpected TGF-beta signaling independent role for TbetaRIII in activating Cdc42, altering the actin cytoskeleton and reducing directional persistence to inhibit random migration of both cancer and normal epithelial cells
TGFBR3	7049	20153821	A review on the role of TbetaRIII as a suppressor of cancer progression and/or as a metastasis suppressor.
TGFBR3	7049	23835618	Low TGFBR3 expression is associated with bladder urothelial carcinoma and enhanced in the invasive bladder cancer.
THBS1	7057	11801541	Increased expression is associated with breast tumor metastasis
THBS1	7057	11882904	Stromally derived TSP-1 upregulates the production of MMP-9 and regulates matrix remodeling of tumor invasion in pancreatic adenocarcinoma
THBS1	7057	12927044	TSP1 expression is significantly higher in malignant epithelial sources over normal and benign epithelial sources, but no difference in expression levels is evident between primary tumors with or without metastases.
THBS1	7057	17106256	cyclin D1 may have a central role in mediating invasion and metastasis of cancer cells by controlling Rho/ROCK signaling and matrix deposition of TSP-1 [review]
THBS1	7057	18222489	The data document the importance of TSP-1 in promoting MMP-2-mediated cancer cell invasion.
THBS1	7057	18321763	TSP-1 promotes human follicular thyroid carcinoma cell invasion mainly through up-regulation of the urokinase-dependent activity.
THBS1	7057	18661355	recent results dealing with regulation of TSP-1 expression by epidermal growth factor & hepatocyte growth factor; results show TSP-1 can have opposite effects on cell invasion depending upon type of differentiated thyroid carcinoma studied [review]
THBS1	7057	19584238	Upregulation of thrombospondin-1 and angiogenesis is associated with pancreatic cancer metastasis.
THBS1	7057	19762163	Increased VEGF and MMP-9 expression as well as decreased TSP-1 expression may play considerable roles in the invasion and differentiation of urothelial carcinoma.
THBS1	7057	19959938	HGF is a potential downregulator of TSP-1, through MAPK signaling pathways, leading to the induction of MMP-9 expression and subsequent invasion of SKOV-3ovarian cancer cells.
THBS1	7057	20498063	Knockdown of B-Raf(V600E) resulted in TSP-1 down-regulation and a reduction of adhesion and migration/invasion of human thyroid cancer cells.
THBS1	7057	22037878	Data suggest that intratumor disruption of the hypoxic cycle through thrombospondin 1 (TSP1) silencing will limit tumor invasion.
THBS1	7057	22231149	Overexpression of THBS-1 in stromal myofibroblasts is associated with tumor growth and nodal metastasis in gastric carcinoma. THBS-1 may activate latent transforming growth factor-beta1 to stimulate fibroblasts to differentiate into myofibroblasts.
THBS1	7057	23380452	these data demonstrate that PRMT6 overexpression is associated with regulation of motility and invasion through up-regulation of TSP-1 and down-regulation of MMPs in human cancer cells.
THY1	7070	16007174	THY1 is a good candidate tumour suppressor gene in nasopharyngeal carcinoma, which is significantly associated with lymph node metastases.
THY1	7070	19615261	Down-regulated/loss expression of THY1 protein in epithelial ovarian cancer is significantly correlated with cancer cell proliferation and metastasis in the epithelial ovarian cancer.
THY1	7070	23382045	High CD90 tumor-initiating cell population is associated with an aggressive signature and metastatic capacity in esophageal cancer.

TNXB	7148	18091320	This study showed different patterns of expression of tenascin and fibronectin along the process of tumorigenesis and tumor progression in pleomorphic adenoma, a fact that might play a role in invasion properties of these tumors.
TP53	7157	11763417	A lower invasiveness and shorter survival was seen in tumors with a TP53 mutation
TP53	7157	11793443	TP53-mutated tumors need fewer additional genetic alterations to develop metastases in primary head and neck tumors compared with TP53 wild-type primary tumors.
TP53	7157	11920528	expression has an independent effect on prediction of survival, progression, and development of metastasis in transitional cell bladder carcinoma
TP53	7157	12190289	p53 was detected more frequently in CIN I compared with CIN II/III and invasive carcinoma
TP53	7157	12452062	abnormal expressions of COX-2, p53, PCNA, and nm23 associate with malignant potential, lymph node metastasis and clinical stage, and they might therefore play a role in development of gastric cancer
TP53	7157	12607595	VEGF expression plays a role in promoting angiogenesis in invasive ductal carcinoma of the breast, and p53 is likely to be involved in regulating VEGF expression.
TP53	7157	12905527	role of p53 gene in the biophysics and biology in murine erythroleukemia cell line with the goal of understanding the influence of this tumor suppressor gene on the deformability and metastasis of tumor cells
TP53	7157	12926152	p53 is involved in the progression of anal cancer and its expression increases from early in the development of pre-invasive anal lesions.
TP53	7157	14566828	value of this tumor marker regarding relapse, metastasis and death in resectable non-small cell lung cancer
TP53	7157	14648233	Bulgarian patients with invasive breast cancer screned for p53 gene mutations registered a 33.33% frequency of mutations.
TP53	7157	14749371	v-Fos-stimulated invasion is independent of the pRb/p16(INK4a) and p53 tumor suppressor pathways and telomerase
TP53	7157	15367885	p53 mutations were found in 70% of pancreatic adenocarcinoma cell lines and 33% of primary tumors. p53 missense mutations correlated with more frequenct metastases to all sites.
TP53	7157	16169726	HER-2/neu and p53 are likely to be involved in the regulation of COX-2 expression in invasive ductal carcinomas of the breast.
TP53	7157	16248462	Overexpression of p53 is related with the stage and cervical lymph node metastasis of nasopharyngeal carcinoma.
TP53	7157	16839413	The Bcl-2 protein expression has a close correlation with p27 and p53 protein expressions and the proliferation activity determined by MIB-1 counts in invasive ductal carcinoma of the breast.
TP53	7157	16951228	The p53-positive tumors had more lymph node metastasis, and p53-positive had the worst prognosis with gastric cancer.
TP53	7157	17130833	suggest a mechanism for elevated CXCR4 expression and metastasis of breast cancers with p53 mutations or isoform expression
TP53	7157	17259658	TP53 mutational status seems to be an important prognostic factor in patients undergoing surgical resection of colorectal cancer with hepatic metastases.
TP53	7157	17374967	xeroderma pigmentosum group C protein polymorphism might affect p53 alteration and the molecular pathway defined by the p53 alteration in the development of muscle-invasive bladder cancer
TP53	7157	17417775	we have identified PROX1 as a novel target gene that is hypermethylated and transcriptionally silenced in primary and metastatic breast cancer.
TP53	7157	17447881	Mutations of p53 were associated with lymph node metastases and III/IV stage of tumors that are signs of unfavorable prognosis in colorectal cancer.
TP53	7157	17449902	Common variation in the TP53 gene could modify the risk of invasive breast cancer.

TP53	7157	17478762	Expression of c-erbB-2 and p53 has no prognostic value in patients with early-stage breast cancer in which axillary lymph node metastasis is absent.
TP53	7157	17504383	Positive staining of p53 was significantly correlated with increased stage, lymph node metastases, and a nonendometrioid histology in endometrial carcinoma.
TP53	7157	17557566	the presence of p53 mutations in the surgical margins of squamous cell carcinoma of the head and neck may not increase the risk of local-regional recurrence, but probably increases the risk of developing distant metastases or second primary tumors
TP53	7157	17641416	N33, STK11 (19p13) and TP53 might play a role in the development of metastasis in larynx and pharynx squamous cell carcinomas.
TP53	7157	17709727	Patients with primary colorectal cancer with low TSP-1 expression, with or without detection of mp53 gene product, are more likely to harbor lymph node metastasis than patients with higher expression.
TP53	7157	17974918	The functional interplay between EGFR overexpression, hTERT activation, and p53 mutation in esophageal epithelial cells with activation of stromal fibroblasts induces tumor development, invasion, and differentiation.
TP53	7157	18094375	Stroma-specific loss of heterozygosity or allelic imbalance is associated with somatic TP53 mutations and regional lymph-node metastases in sporadic breast cancer but not in hereditary breast cancer.
TP53	7157	18230179	Findings suggest TP53 PIN3 Ins16bp polymorphism as a real risk modifier in breast cancer disease, either in sporadic and familial breast cancer. Furthermore, both TP53 polymorphisms are associated with higher incidence of lymph node metastases.
TP53	7157	18264135	p53 has a role in preventing centrosome amplification, ERalpha phenotypic heterogeneity and metastasis in breast cancer
TP53	7157	18330257	The expression of p53 was significantly correlated with lymph node metastasis in laryngeal carcinoma.
TP53	7157	18383208	Increased TP53 expression was associated with invasive adenocarcinoma of the prostate
TP53	7157	18386458	in endometrial carcinoma p53 overexpression was directly associated with unfavorable clinicopathologic factors: advanced stage, histologic subtype, advanced patient age and nodal metastasis
TP53	7157	18393238	The R allele of the TP53 R72P polymorphism may contribute to the etiology of liver metastases, particularly among those with positive P53 expression tumors. Both TP53 C-8343G and C-1863T may be not associated with colorectal liver metastases risk.
TP53	7157	18452596	might be markers of depth of invasion or lymph node involvement in patients with gastric cancers
TP53	7157	18561741	Expression of p53, in cervical intraepithelial neoplasia and invasive squamous cell carcinoma of the uterine cervix
TP53	7157	18670315	TP53 mutation is associated with metastatic pulmonary adenocarcinomas.
TP53	7157	18988302	Results demonstrate for the first time that the 72R allele of the p53 polymorphism has an increased risk for liver metastases in colorectal cancers positive for p53 overexpression.
TP53	7157	19141643	Data are the first demonstration that wild-type p53 protein binds to a response element within the EpCAM gene and negatively regulates EpCAM expression, and transcriptional repression of EpCAM contributes to p53 control of breast cancer invasion.
TP53	7157	19155088	p53 may play a role in influencing tumor metastasis through Lasp1 in hepatocellular caarcinoma.
TP53	7157	19261747	combined deletion of p53 and Pten in bladder epithelium leads to invasive cancer in a novel mouse model. Inactivation of p53 and PTEN promotes tumorigenesis in human bladder cells and is correlated with poor survival in human tumors.

TP53	7157	19367287	TP53 genotyping may be of clinical interest in selecting patients who may benefit from cetuximab-based chemotherapy in metastatic colorectal cancer.
TP53	7157	19403344	Full sequencing of TP53 identifies identical mutations within in situ and invasive components in breast cancer suggesting clonal evolution.
TP53	7157	19437562	Data show that FAT10 and mutant p53 levels in gastric cancer tissue were significantly correlated with lymph node metastasis and tumor, nodes, metastasis staging.
TP53	7157	19448627	Results indicate that wtp53 and p53 mutants may differentially control cancer invasion and metastasis through the p53-MDM2-Slug pathway.
TP53	7157	19513503	Enhanced S100A4 protein expression is clinicopathologically significant to metastatic potential and p53 dysfunction in colorectal cancer.
TP53	7157	19597352	Data show that downregulation of EMMPRIN by p53 leads to a decrease in the activity of MMP-9 and an inhibition of tumor cell invasion.
TP53	7157	19628749	Among human cancers, sebaceous carcinoma of the eyelid may be one of those showing most frequent mutation of the p53 gene, which may not be caused by exposure to UV. p21(WAF1/CIP1) downregulation may be associated with lymph node metastasis.
TP53	7157	19657752	TP53 increased in high-grade invasive ductal breast carcinoma compared to DCIS.
TP53	7157	19787241	Intronic polymorphisms in TP53 indicate lymph node metastasis in breast cancer.
TP53	7157	19836055	results indicated a close association between p53 protein expression in tumor-stromal fibroblasts, and both the presence of nodal metastasis and the outcome of invasive ductal carcinoma patients who received neoadjuvant therapy.
TP53	7157	19909015	Combined analysis of smoking, TP53, and FGFR3 mutations in Tunisian patients with invasive and superficial high-grade bladder tumors.
TP53	7157	19917135	Elevated expression of p53-R175H mutant may exert gain-of-function activity to activate the EGFR/PI3K/AKT pathway and thus may contribute to the invasive phenotype in endometrial cancer.
TP53	7157	20018721	Results identify two critical acquired functions of a stably expressed mutant form of p53 that drive pancreatic ductal adenocarcinoma; first, an escape from Kras(G12D)-induced senescence/growth arrest and second, the promotion of metastasis.
TP53	7157	20129645	A combination of low beta-catenin and high p53 expression in primary colorectal cancers may be a prognostic factor in predicting the progression of the disease, the occurrence of metastasis, and a more severe outcome.
TP53	7157	20299546	aryl hydrocarbon receptor ligands might contribute to tumor progression by inhibiting p53 regulation via the increased expression of the metastasis marker AGR2
TP53	7157	20386703	variants in TP53 were confirmed to be associated with risk of invasive serous ovarian cancer
TP53	7157	20514505	Tp53 gene mutation is associated with the differential diagnosis of primary and metastatic ovarian cancer.
TP53	7157	20811949	Tp53 and EGFR mutations usually precede lymph node metastasis and that these gene statuses in matched primary non-small cell lung cancers and lymph node metastasis show predominantly a concordant pattern
TP53	7157	20878064	we identified distinct novel non-canonical transcriptome networks involved in breast cancer cell proliferation, epithelial to mesenchymal transition , chemoresistance and invasion that arise following abrogation of p53 function
TP53	7157	21046311	As diffuse and multiple features on imaging modalities represent invasive characteristics of the tumors, p53 abnormalities may affect the invasive and aggressive nature of malignant astrocytomas
TP53	7157	21388952	Loss of p53 and acquisition of angiogenic microRNA profile are insufficient to facilitate progression of bladder urothelial carcinoma in situ to invasive carcinoma.

TP53	7157	21478913	These findings show an important role for p53 in the progression of serous borderline ovarian tumors to an invasive carcinoma, and suggest that downregulation of E-cadherin by DNMT1-mediated promoter methylation contributes to this process.
TP53	7157	21501231	A high p53-LI (p53+) was observed in 17/59 cases (28.8%). many p53+/PCNA+ cases could be confirmed as highly invasive cancer. the p53+/PCNA+ cases showed high risk of tumor recurrence; patients with p53+/PCNA+ had worse prognosis.
TP53	7157	21519792	lupulone-triggered enhanced expression of p38 plays a major role in the activation of p53 and of the TRAIL-death receptor apoptotic pathway in SW620 human colon cancer-derived metastatic cells
TP53	7157	21831840	These results also show that the extent of advanced cancer traits, such as invasion, may be determined by alterations in individual components of p53/MET regulatory network.
TP53	7157	21868761	KLF5 transactivates NOTCH1 in the context of p53 mutation or loss. KLF5 loss limited NOTCH1 activity and was sufficient on its own to transform primary human keratinocytes harboring mutant p53, leading to the formation of invasive tumors.
TP53	7157	21986947	Mutant p53 drives multinucleation and breast cancer invasion through a process that is suppressed by ANKRD11.
TP53	7157	22165746	direct correlation between p53 expression and gastric cancer and it indicates the ability of carcinoma cells to invade blood vessels
TP53	7157	22187033	Mutation in TP53 is the most common genetic alteration reported during metastasis to the brain in breast cancer
TP53	7157	22354696	TP53 mutations occur more frequently in pleomorphic variants of invasive lobular carcinoma (ILC) than ILC.
TP53	7157	22510504	The expression of TIMP-3 in the non-small cell lung cancer and metastasis groups was upregulated.
TP53	7157	22707142	among Asians, the p53 codon 72 Arg/Arg genotype is associated with a modestly decreased risk of gastric cancer, and that this difference in genotype distribution may be associated with cancer stage, location, differentiation and metastasis.
TP53	7157	22714709	PUMA, c-Myb and p53 protein expression closely relates to the carcinogenesis, fast-progression, easy-metastasis, high-invasion, and poor-prognosis in gallbladder adenocarcinoma.
TP53	7157	22847613	These data provide a novel understanding of the roles of p53 gain-of-function mutations in accelerating tumor progression and metastasis through modulation of the miR-130b-ZEB1 axis.
TP53	7157	23106581	Overexpression of TERT was comparable to that for p53 in invasive keratinizing squamous cell carcinoma; significant differences were calculated for differentiated vulvar intraepithelial neoplasia and high-grade classic vulvar intraepithelial neoplasia.
TP53	7157	23232572	High-grade bladder in situ/muscle-invasive carcinoma on is characterized by alteration of p53 and pRB.
TP53	7157	23404342	Loss of wild type-p53 may promote the bone metastasis of prostate cancer at least partially through repressing miR-145.
TP53	7157	23670029	Patients with mtp53 tumors were older at diagnosis, had more incidence of liver metastasis, and more frequent PTEN loss.
TP53	7157	23687381	this data suggest that plakoglobin regulates gene expression in conjunction with p53 and that plakoglobin may regulate p53 transcriptional activity, which may account, in part, for the tumor/metastasis suppressor activity of plakoglobin.
TP53	7157	23873310	our data indicate p53 showing predicting role in diagnosis to death (OS1) for visceral metastasis breast cancer (VMBC) patients. but not for metastases to death (OS2).

TP53	7157	23881403	The study demonstrates coordinated regulation of Numb, MDM2 and p53 on cell invasion and migration in pancreatic cancer.
TP53	7157	23915071	Our data indicate that parallel expression of MMP-2 epithelial/stromal cells and p53 may enhance cells invasion and metastasis in ovarian carcinoma.
TP53	7157	23959174	Furthermore, our study reveals that high expression of RPN2 and concomitant accumulation of mtp53 were associated with cancer tissues in a small cohort of metastatic breast cancer patients.
TP53	7157	23982184	Loss of P53 promotes the invasion and metastasis ability of prostate cancer cells and the mechanism is correlated with FAK-Src signaling pathway.
TP53	7157	24047084	Studies indicate that tumor suppressor gene p53 and other genes associated with DNA repair, cell proliferation, migration and angiogenesis are critical for the development of pterygium.
TP53	7157	24146864	p53 and p16 expressions are important for the development of triple-negative breast cancers in ductal carcinoma in situ and invasive ductal carcinoma.
TP53	7157	24285725	there is a p53-induced double-negative feedback loop involving miR-15a/16-1 and AP4 that stabilizes epithelial and mesenchymal states, respectively, which may determine metastatic prowess in colorectal cancer
TP53	7157	24487586	Myo10 upregulation in mutant p53-driven cancers is necessary for invasion and that plasma-membrane protrusions.
TP53	7157	24725405	Study combined several orthogonal approaches and models to explore the basis whereby mutant p53 promotes invasion and metastasis in pancreatic ductal adenocarcinoma (PDAC)and identified the PDGFRb as necessary and sufficient to mediate the effects of mutant p53 on invasion and metastasis in both a murine model and human PDAC cells.
TP53	7157	24924261	TP53 mutations may possibly be associated with a more indolent course of colorectal cancer after the diagnosis of metastatic disease.
TP53	7157	25119169	Ductal adenocarcinoma of the pancreas metastasing to the liver retained the primary tumour's SMAD4/TP53 protein status.
TPM1	7168	15897890	Epigenetic suppression of TPM1 may alter TGF-beta tumor suppressor function and contribute to metastatic properties of tumor cells.
TPM1	7168	22740512	Expression of low molecular weight isoforms from TPM1 and TPM3 genes is regulated very differently, which has a critical role in processes such as cancer metastasis.
TPO	7173	12459031	Positivity in TPO-RT-PCR correlates significantly with metastatic disease in cancer patients and with the presence of thyroid disease in general.
TPO	7173	22435912	analysis of how minimally invasive follicular thyroid carcinoma developed in dyshormonogenetic multinodular goiter due to thyroid peroxidase gene mutation [case report]
TRPM2	7226	23910495	TRPM7 plays a critical role in breast cancer cell migration through its kinase domain.
TYK2	7297	17920038	These results suggest that Tyk2 signaling in prostate cancer cells facilitate invasion of these cells, and interference with this signaling may be a potential therapeutic pathway.
TYK2	7297	22116632	data highlight the role of TYK2 downregulation in breast cancer cell de-differentitation and initiation of regional metastasis
UBE2I	7329	20023705	Molecular link exists between Ubc9 and the metastasis genes such as CDC42 and CXCR4 in breast cancer invasiveness and metastasis
UBE2I	7329	23381475	we demonstrated that upregulation of Ubc9 expression promotes migration and invasion. Ubc9 likely plays an important role in cancer progression by promoting invasion and metastasis in lung cancer.
XRCC1	7515	17116943	XRCC1-01, XRCC3-01, and CCND1-01 may be predictive of survival outcome in patients with metastatic breast cancer treated with DNA-damaging chemotherapy

XRCC1	7515	19127428	These results suggest that XRCC1Arg399Gln is a strong predictor of survival outcome for patients with metastatic breast cancer following high dose chemotherapy and autologous stem cell transplantation
XRCC1	7515	20863780	XRCC1 194Trp allele significantly increased the risk of gastric cancer and also associated with risk of gastric cardia carcinoma and promoted distant metastasis of gastric cancer.
XRCC1	7515	23435956	individuals whose expression of XRCC1, ERCC4, ERCC2, and ERCC1 are reduced may be at a higher risk of developing squamous intraepithelial lesion which eventually leads to invasive cervical carcinoma.
CXCR4	7852	11912162	Use of the stromal cell-derived factor-1/CXCR4 pathway in prostate cancer metastasis to bone.
CXCR4	7852	12499259	Vascular endothelial growth factor promotes breast carcinoma invasion in an autocrine manner by regulating the chemokine receptor CXCR4.
CXCR4	7852	12519884	findings suggest that a subset of anaplastic thyroid carcinoma cells expresses functional CXCR4, which may be important in tumor cell migration and local tumor invasion
CXCR4	7852	12690099	CXCR4 is induced by NF-kappa B and has a role in breast cancer cell migration and metastasis
CXCR4	7852	12927045	Tumors with high CXCR4 expression showed more extensive lymphatic spread than those with low CXCR4 expression, because the range and number of metastatic nodes were significantly larger in cases of high CXCR4 expression than in those with low expression.
CXCR4	7852	14567988	stromal-cell-derived factor-1alpha /CXCR4 signaling may be involved in the establishment of lymph node metastasis in oral squamous cell carcinoma via activation of both Extracellular Signal-Regulated Kinases and Akt induced by src-Family Kinases
CXCR4	7852	15026622	CXCR4 is expressed in human ductal carcinoma in situ as well as in atypical ductal hyperplasia; expression at this early step of tumor development suggests CXCR4 role in providing selective advantage to such cells on their way to metastasizing carcinomas
CXCR4	7852	15201990	CXCR4 signaling in oral SCC cells might be involved in the diverse action of oral squamous cell carcinoma, including invasion or micrometastasis at the primary site and lymph node metastasis.
CXCR4	7852	15235108	CXCR4 and its ligand stromal cell-derived factor 1alpha (SDF-1alpha) induced transendothelial breast cancer cell migration through activation of the PI-3K/AKT pathway and Ca(2+)-mediated signaling.
CXCR4	7852	15363550	CXCR4 may be a novel regulator of head and neck squamous cell carcinoma metastatic processes
CXCR4	7852	15467730	Mechanisms of CXCR4/CXCL12-mediated prostate cancer cell migration and invasion.
CXCR4	7852	15486895	CXCR4 expression in neuroblastoma primary tumors is significantly correlated with the pattern of metastatic spread
CXCR4	7852	15492752	CXCR4 signaling mediates the establishment of lymph node metastasis in oral squamous cell carcinoma.
CXCR4	7852	15540205	CXCR4 is a potential target for the attenuation of bladder cancer metastases.
CXCR4	7852	15542430	These results provide a plausible mechanism for HER2-mediated breast tumor metastasis and establish a functional link between HER2 and CXCR4 signaling pathways.
CXCR4	7852	15548713	CXCR4 receptor is frequently expressed in metastatic pancreatic tumor cells. CXCR4 not only stimulates cell motility and invasion but also promotes survival and proliferation

CXCR4	7852	15805285	CHK down-regulates CXCR4 through the YY1 transcription factor, leading to decreased CXCR4-mediated breast cancer cell motility and migration.
CXCR4	7852	15843590	In an organotypic melanoma culture, cytotoxic T cells (CTL) mediated by chemokine receptor CXCR4 expressed by the CTL migrate from the top layer through a collagen/fibroblast separating layer toward the tumor cells, resulting in tumor cell apoptosis.
CXCR4	7852	15844659	prostate cancers may be influenced by the CXCL12:CXCR4 pathway during metastasis
CXCR4	7852	16114056	CXCL12/CXCR4 axis is expressed in prostate cancer bone metastasis and exogenous CXCL12 induced MMP-9 expression.
CXCR4	7852	16230077	CXCR4 expression mediates organ-specific metastasis of pancreatic cancer cells.
CXCR4	7852	16322285	Overexpression of CXCR4 is associated with the metastatic potential of human non-small cell lung cancer
CXCR4	7852	16327980	findings suggest that CXCR4 might be involved in the lymph-node metastasis of oral squamous cell carcinoma
CXCR4	7852	16494043	CXCR4 and CCR7 are highly expressed in laryngeal carcinoma. Expression was associated with tumor grade, clinical stage and neck lymph node metastasis.
CXCR4	7852	16618732	CXCR4 function is subject to complex and potentially tightly controlled regulation in breast cancer cells via differential G protein-receptor complex formation, and this regulation may play a role in the transition from nonmetastatic to malignant tumors.
CXCR4	7852	16823836	Adenosine increases cell-surface CXCR4 protein, which enables the carcinoma cells to migrate toward CXCL12.
CXCR4	7852	16969502	expression of AR down-regulates the migratory responses of human prostate cancer cells via CXCR4 and CCR1
CXCR4	7852	17032700	Significantly higher CXCR4 expression is associated with lymph node metastasis in human cervical cancer
CXCR4	7852	17075975	CXCR4 expression in colorectal liver metastases suggests it is a predictive factor. CCL20 and receptor CCR6 expression in hepatocellular carcinomas indicates a role of a CCL20/CCR6 ligand-receptor pair in liver carcinogenesis and progression.
CXCR4	7852	17130833	suggest a mechanism for elevated CXCR4 expression and metastasis of breast cancers with p53 mutations or isoform expression
CXCR4	7852	17355795	CXCR4 overexpression indicates a higher lymph node metastasis potential of cervical adenocarcinoma.
CXCR4	7852	17461449	These results indicated that the activation of CXCR4 and its signaling pathways (MEK1/2 and Akt) are essential for CXCL12-induced cholangiocarcinoma cell invasion.
CXCR4	7852	17504381	Primary cervical adenocarcinoma cells expressing CXCR4 are significantly more likely to metastasize to pelvic lymph nodes.
CXCR4	7852	17510563	The CXCL12 ligand with its exclusive receptor CXCR4 has a pivotal role in the directional migration of cancer cells during the metastatic process. [REVIEW}
CXCR4	7852	17634424	These results suggested that, in cases of oral squamous cell carcinoma, the paracrine SDF-1/CXCR4 system potentiates lymph node metastasis, but distant metastasis might require the autocrine SDF-1/CXCR4 system.
CXCR4	7852	17641542	CXCR4 contributed to lymphatic involvement and nodal metastasis in colorectal cancer.
CXCR4	7852	17687643	prolonged Akt activation is an important signaling pathway for breast cancer cells expressing CXCR4 and is necessary for CXCL12-dependent cell migration
CXCR4	7852	17763975	High expression of nuclear CXCR4 was significantly correlated with lymph node metastasis in breast cancer.
CXCR4	7852	17889832	Data support the conclusion that miRNA against CXCR4 can serve as an alterative means of therapy to lower CXCR4 expression and to block the invasion and metastasis of breast cancer cells.
CXCR4	7852	17893878	Overexpression of SDF-1alpha/CXCR4 is associated with enhanced peritoneal metastasis of epithelial ovarian carcinoma

CXCR4	7852	17916907	bone marrow-derived-SDF-1alpha enhances the invasiveness of lung cancer cells by increasing MMP-9 expression through the CXCR4/ERK/NF-kappaB signal transduction pathway
CXCR4	7852	18201276	the pattern of chemokine receptor CXCR4 expression in patients with metastatic prostate cancer.
CXCR4	7852	18337451	Lipid raft-disrupting agents inhibited raft-associated CXCL12/CXCR4 transactivation of the HER2 and cellular invasion in prostate cancer cells.
CXCR4	7852	18373506	In node-negative breast cancers CXCR4 membrane expression is prognostic and probably plays a key role in vascular invasion by cancer cells and a role in lymphatic invasion.
CXCR4	7852	18416455	Inhibition of CXCR4 activity with AMD3100 decreases invasion of human colorectal cancer cells in vitro.
CXCR4	7852	18443596	Expression of nuclear CXCR4 predicts lymph node (LN) metastasis in colorectal cancer (CRC).
CXCR4	7852	18487224	CXCL12 enhances Laryngeal and hypopharyngeal squamous cell carcinoma cell invasiont through paracrine-activated CXCR4, triggering MMP-13 upregulation.
CXCR4	7852	18502034	Breast cancer metastasis suppressor 1 inhibits SDF-1alpha-induced migration of non-small cell lung cancer by decreasing CXCR4 expression.
CXCR4	7852	18599607	Neuronal and endothelial cells exposed to VEGF up-regulated the expression of SDF-1alpha. CXCR4-positive tumor cells migrated toward a SDF-1alpha gradient in vitro, whereas inhibition of CXCR4 expression decreased their migration
CXCR4	7852	18624931	downregulation induces anti-proliferative and anti-invasive effects in oral squamous cell carcinoma
CXCR4	7852	18696160	High CXCR4 expression is associated with extrathyroidal extension, angiolymphatic invasion, and lymph node metastasis in papillary thyroid carcinoma.
CXCR4	7852	18701133	The association between aberrant expression of CXCR4 in the nucleus of non-small cell lung cancer and metastasis to lymph nodes points toward a potential tumor metastasis promoting function of nuclear CXCR4.
CXCR4	7852	18803056	CXCR4 is associated with the progression of colorectal carcinoma. High CXCR4 expression is associated with metastasis.
CXCR4	7852	18836306	CXCR4/SDF-1 interaction blockade in prostate cancer cells by hTERT promoter induces CXCR4 knockdown and prevents metastasis
CXCR4	7852	18851783	CXCR4/SDF-1 participates in tumor invasiveness and metastasis in cervical cancer through regulating the adhesion ability by activating the MAPK signaling transduction pathway and promoting secretion of MMP-2.
CXCR4	7852	18949410	In gastric cancer, CXCL12/ CXCR4 axis seems to be more strongly associated with lymphatic or hematogenous metastasis than the establishment of peritoneal deposits.
CXCR4	7852	18954561	CXCR4 and CXCL12 may play an important role in the metastasis of HCC by promoting the migration of tumor cells.
CXCR4	7852	19020745	Results suggest that CXCR4 expression, histological patterns, and metastatic potential are closely related in adenoid cystic carcinoma.
CXCR4	7852	19148483	Strong expression of CXCR4 by gastric cancer cells is associated with lymphatic metastasis in patients with gastric cancer, and CXCR4 may play an important role during gastric cancer progression.
CXCR4	7852	19199057	CXCR4-CXCL12 axis can play a role in the progression of endometrial carcinoma and that specific therapies with antagonists of chemokines receptors could be of help in the treatment of metastatic patients.
CXCR4	7852	19302975	CXCR4/SDF-1 pathway was critical for TLR9 agonist to enhance the metastasis of human lung cancer cells.
CXCR4	7852	19373784	At least one mutated allele of CXCR4 gene is associated with the development of stage III or IV and the induction of lymph-node metastasis of oral cancer disease in

			Taiwanese.
CXCR4	7852	19438749	This study suggests that up-regulation of cytoplasmic expression of CXCR4 might be one of the molecular mechanisms facilitating lymph node metastasis of invasive micropapillary carcinoma
CXCR4	7852	19513547	CCR7 and CXCR4 expression predicts lymph node status including micrometastasis in gastric cancer.
CXCR4	7852	19513623	The SDF-1/CXCR4 axis played a critical role in the metastasis of human ovarian cancer by increasing the adhesion capability of cancer cells and the activity of MMP-2 and MMP-9 via ERK1/2 signaling pathway.
CXCR4	7852	19563077	Arrest of CXCR4 can inhibit liver metastasis of colon cancer through blocking cell proliferation and migration induced by SDF-1.
CXCR4	7852	19568410	the CXCR4 ligand CXCL12 is expressed by endothelial cells and likely Kupffer cells lining the liver sinusoids of liver metastatic human HEP-G2 hepatoma and HT-29LMM colon cancer
CXCR4	7852	19570110	significant association between expression of CXCR4 and lymph node metastasis, tumor size, UICC stage, tumor histology grade. SDF-1 stimulated proliferation of oral SCC cell and CXCR4 neutralization by monoclonal antibodies decreased proliferation.
CXCR4	7852	19580679	Neuropilin-2 in breast cancer has a role in lymph node metastasis, poor prognosis, and regulation of CXCR4 expression
CXCR4	7852	19588204	High CXCR4 is associated with liver metastasis of colorectal cancer.
CXCR4	7852	19615218	The expression of CXCR4 was significantly associated with lymphatic metastasis of pancreatic adenocarcinoma.
CXCR4	7852	19615279	CXCR4 expression is closely correlated to metastasis of nasopharyngeal carcinoma.
CXCR4	7852	19784070	Findings indicate that PAUF enhances the metastatic potential of pancreatic cancer cells, at least in part, by upregulating CXCR4 expression.
CXCR4	7852	19825996	Our results suggest that the phenotypic knockout strategy of CXCR4 using a novel recombinant protein TAT/54R/KDEL might be a possible approach for inhibiting relative tumor metastasis mediated by CXCR4/CXCL12 interaction.
CXCR4	7852	19859817	These findings implicate the CXCR4-CXCL12 axis in the metastasis of renal cell carcinoma.
CXCR4	7852	19885584	CXCR4 nuclear localization follows binding of its ligand SDF-1 and occurs in metastatic but not primary renal cell carcinoma
CXCR4	7852	19921580	Shikonin may also play a role in anti-metastasis via down-regulating the expression of CXCR4 and reducing the CXCL12-induced migratory response in colorectal carcinoma cells.
CXCR4	7852	20439195	We studied a unique cohort of 21 primary lung cancers with matched adrenal metastases for the expression of CX3CR1, CXCR4, CCR6, and CCR7
CXCR4	7852	20460402	The CXCR4-SDF1 axis has an important role in epithelial ovarian carcinoma (EOC) metastasis by a mechanism that targets EOC metastasis.
CXCR4	7852	20484957	Pancreatic stellate cells can secrete SDF-1 and increase the invasion of pancreatic cancer cells through the SDF-1/CXCR4 axis.
CXCR4	7852	20492653	CXCR4 is expressed on breast cancer cells and exposure to hypoxia upregulated this expression, increasing migratory and invasive potential.
CXCR4	7852	20563655	stromal derived factor-1 (SDF-1) and chemokine receptor (CXCR4) have roles in bone metastasis of renal carcinoma
CXCR4	7852	20578990	In patients with renal cell carcinoma, level of CXCR4 and CXCR7 expression in tumor tissue correlated with disease free survival and lymphatic metastasis; higher CXCR4 and CXCR7 expression predicts earlier relapse.
CXCR4	7852	20603605	Data suggest that that ITF2 is one of the CXCR4 targets, which is involved in CXCR4-dependent tumor growth and invasion of breast cancer cells.

CXCR4	7852	20723431	Increased protein expressions of nuclear CXCR4, VEGF-C, and CK-19 are independent risk factors for developing lymph node metastasis in hepatocellular carcinoma.
CXCR4	7852	20953377	HIF-1, CXCR4, and VEGF high expression levels in colon cancer were correlated positively with TNM stage, lymph node involvement, and distant metastasis.
CXCR4	7852	21059341	NF-kappaB-regulated CXCL12/CXCL4 autocrine system is essentially involved in the invasiveness of ovarian cancer.
CXCR4	7852	21080209	Enoxaparin (LMWH) inhibits the formation of hepatic metastasis of colon cancer by disrupting the interaction of CXCR4 and CXCL12.
CXCR4	7852	21087342	CXC chemokine receptor 4 (CXCR4) was found to be expressed in many tumors and significantly correlated with invasion, angiogenesis, metastasis, and prognosis.
CXCR4	7852	21454012	suppression of CXCR4 inhibits EGFRvIII-mediated breast cancer cell invasion and proliferation
CXCR4	7852	21468602	The high in vitro metastatic potential of human bladder carcinoma cell sublines is closely associated with increased CXCR4 expression.
CXCR4	7852	21527066	The expressions of CXCL12/CXCR4 and CXCL16/CXCR6 were significantly higher in epithelial ovarian carcinomas than in normal epithelial ovarian tissues or benign epithelial ovarian tumors. Expression of CXCR4 was related to lymph node metastasis.
CXCR4	7852	21630055	CXCL12 and CXCR4 are related to formation of gastric tumors and lymph node metastasis
CXCR4	7852	21633638	CXCR4/SDF-1 axis is involved in lymph node metastasis of gastric carcinoma.
CXCR4	7852	21738044	CXC chemokine receptor 4 plays a critical role in the metastasis of human ovarian cancer possibly through modulating the Wnt/beta-catenin pathway.
CXCR4	7852	21831961	These results indicated that PEA3 could activate CXCR4 promoter transcription and promote breast cancer metastasis.
CXCR4	7852	21934106	signaling-selective inhibition of the CXCR4-Galpha(13)-Rho axis prevents the metastatic spread of basal-like breast cancer cells.
CXCR4	7852	22075627	CXCR4 expression in metastatic liver tumors together with the upregulation of CXCL12 in hepatocytes may help to predict the clinical outcomes of patients with CLM after hepatectom
CXCR4	7852	22152016	CXCR4 and CXCR7 play different roles in metastasis, with CXCR4 mediating breast cancer invasion and CXCR7 impairing invasion but enhancing primary tumor growth through angiogenesis.
CXCR4	7852	22200669	Loss of breast cancer metastasis suppressor 1 promotes ovarian cancer cell metastasis by increasing chemokine receptor 4 expression.
CXCR4	7852	22266857	CXCR7+ cells promote growth and metastasis of CXCR4+ breast cancer cells.
CXCR4	7852	22299827	ED is a novel blocker of CXCR4 expression and, thus, has enormous potential as a powerful therapeutic agent for metastatic cancer.
CXCR4	7852	22377565	CXCR4 is a strong independent prognostic biomarker associated with distant metastatic recurrence in pancreatic adenocarcinoma.
CXCR4	7852	22430135	These findings suggest a possible role for the CXCL12/CXCR4 axis in the metastatic evolution of non-small-cell lung cancer
CXCR4	7852	22463589	High CXCR4 expression correlates with a poor response to sunitinib in metastatic renal cancer.
CXCR4	7852	22490156	Elevated levels of CXCR4 and MMP-9 are found in small cell lung cancer tissues. CXCR4 expression might be correlated with bone metastasis. CXCR4 is an independent prognostic factor for disease-free survival in SCLC.
CXCR4	7852	22689289	Both CXCR3 and CXCR4 are involved in metastasis of colorectal cancer to lymph nodes, lungs and liver.
CXCR4	7852	22871210	Flow cytometry analysis was performed to detect distinct subsets with CD133 and CXCR4 markers in human primary and metastatic CRC tissues. In human specimens, CD133(+)CXCR4(+) cells were higher in liver metastases than in primary colorectal tumors.

CXCR4	7852	22902648	CXCR4 is essential for ovarian tumor cell proliferation and invasion.
CXCR4	7852	23023480	hypoxia upregulates CXCR4 in gastric cancer cells in a HIF-1alpha-dependent manner, and upregulation of CXCR4 plays a role in cancer cell migration and invasion
CXCR4	7852	23259294	SDF-1 and CXCR4 protein are highly expressed in laryngocarcinoma and in metastatic lymph node tissue. Expression is correlated with lymph node metastasis, clinical stage and pathological grading.
CXCR4	7852	23468933	nuclear CXCR4 may be a mechanism underlying prostate cancer recurrence, increased metastatic ability and poorer prognosis after tumors have been treated with therapy that targets plasma membrane CXCR4
CXCR4	7852	23472069	up-regulation of TPST-1 and tyrosine sulfation of CXCR4 by LMP1 might be a potential mechanism contributing to nasopharyngeal carcinoma metastasis
CXCR4	7852	23591873	findings indicate that a chemokine-controlled pathway, consisting of Galphai2, ELMO1/Dock180, Rac1 and Rac2, regulates the actin cytoskeleton during breast cancer metastasis
CXCR4	7852	23647548	TRAIL-induced miR-146a expression suppresses CXCR4-mediated human breast cancer migration.
CXCR4	7852	23744532	Expression of miR-126 inhibited colon cancer cell viability and reduced tumor cell migration and invasion capacity by its negative regulation of CXCR4 expression.
CXCR4	7852	23765204	High CXCR4 expression is associated with invasive squamous cell vulvar cancers.
CXCR4	7852	23777983	Our results show CD133(+) and CD133(+)CXCR4(+) cancer cells correlate with the presence of lymph node metastasis in colorectal cancer.
CXCR4	7852	23800042	CXCR4 silencing by siRNA could suppress the proliferation, invasion and metastasis of esophageal carcinoma cell lines.
CXCR4	7852	23827153	The expression of chemokine receptor CXCR4 was up-regulated in the tissue with breast cancer bone metastasis.
CXCR4	7852	23845465	Early genetic events such as the loss of RANKL and the gain of CXCR4 expressions probably facilitate the metastatic progression concomitant with the primary tumor establishment.
CXCR4	7852	23868972	Immunohistochemical analysis of human breast tumor tissues shows a significant increase of PRG expression in the invasive areas of the tumors, suggesting that this RhoGEF is associated with breast tumor invasion in vivo
CXCR4	7852	23921147	integrin-mediated cell-ECM interactions can modulate tumor cell morphology, and regulate the expression of chemokine receptors CXCR7 and CXCR4 which are associated with the invasive phenotype and progression of prostate cancer.
CXCR4	7852	23924922	SDF-1alpha acts through CXCR4 to induce ovarian cancer cell migration, which could be facilitated by CD40 activation.
CXCR4	7852	24025971	CXCL12 and CXCR4 have a single ligand-single receptor relationship in mediating non-small cell lung cancer migration and metastasis, and in context of NSCLC that expresses CXCR4, the CXCR4-CXCL12 axis is not involved in angiogenesis of the primary tumor
CXCR4	7852	24101191	This retrospective study shows that a high expression of CXCR4 in breast cancer is associated with earlier distant metastasis and bone metastasis in breast cancer
CXCR4	7852	24175834	High CXCR4 expression promotes proliferation and invasion of pancreatic cancer.
CXCR4	7852	24238971	In colorectal cancer with liver metastasis, CXCR4 expression demonstrated associations with local progression, liver metastasis, and poor overall survival.
CXCR4	7852	24256053	Aberrant expression of CXCR4 significantly contributes to metastasis and predicts poor clinical outcome in breast cancer.
CXCR4	7852	24288553	Overexpression of HIF-2alpha, TWIST, and CXCR4 is associated with lymph node metastasis in papillary thyroid carcinoma.

CXCR4	7852	24318902	miR-139 targets CXCR4 and inhibits proliferation and metastasis of laryngeal squamous carcinoma cells
CXCR4	7852	24378831	S100A4, FOS and CXCR4, playing a major role in tumor progression and metastasis, are downregulated by sorafenib.
CXCR4	7852	24390633	these results demonstrate considerable potential of high-affinity CXCR4-blocking agents for OS tumor cell homing suppressive treatment in metastasizing OS complementary to current (neo)-adjuvant chemotherapy.
CXCR4	7852	24472670	These data suggest that CXCR4 inhibition by CTCE-9908 decreases the invasion potential in vitro, which then translated to a reduction of tumor spread with associated reduction in angiogenesis.
CXCR4	7852	24475985	Overexpression of CXCR4 was significantly associated with lymph node status and distant metastasis and indicated poor overall and disease free survival in breast cancer patients.
CXCR4	7852	24590865	Breast cancer nodal metastasis correlates with tumour and lymph node methylation profiles of Caveolin-1 and CXCR4.
FXR1	8087	23881279	FXR1 and UPF1 may have a functional role in prostate cancer progression and metastasis.
IRS2	8660	17030631	Transgenic mice overexpressing IRS2 in the mammary gland show progressive mammary hyperplasia, tumorigenesis and metastasis.
IRS2	8660	17361103	This review points out that IRS-2, which is implicated in mediating signals to promote tumor cell survival, growth and motility, is a positive regulator of breast cancer metastasis.
KRT75	9119	21427063	we observed a possible association between CK5/6 expression in the primary tumor and multiple versus solitary breast carcinoma brain metastasis
RABEP1	9135	21271679	Breast cancer cell line studies showed that microRNA, miR-373, was capable of promoting breast cancer invasion and metastasis via translational inhibition of TXNIP and RABEP1 that were the direct target genes of miR-373.
NUMBL	9253	23440423	Numbl-Klf4 signaling is critical to maintain multiple nodes of metastatic progression, including persistence of cancer-initiating cells.
NUMBL	9253	23681800	Numbl might be involved in the inhibition of growth, proliferation, and invasion of 95-D lung cancer cells.
MAGED1	9500	17140727	studies indicate for the first time that NRAGE could suppress metastasis of melanoma and pancreatic cancer probably through downregulation of MMP-2
MAGED1	9500	21109781	we establish the roles for Dlxin-1, one as an anti-tumorigenic and anti-invasive protein in high-grade gliomas and the other as an inducer of differentiation of glioma stem cells.
IQSEC1	9922	18084281	Results indicate that GEP100 links EGFR signalling to Arf6 activation to induce invasive activities of some breast cancer cells, and hence may contribute to their metastasis and malignancy.
IQSEC1	9922	21858086	Data suggest that GEP100-Arf6-AMAP1-cortactin pathway, activated by VEGFR2, appears to be common in angiogenesis and cancer invasion and metastasis, and provides their new therapeutic targets.
IQSEC1	9922	21966491	Cinical study indicates that co-overexpression of Her2 with GEP100 in primary lung adenocarcinomas of patients is correlated with the presence of their node-metastasis with a statistical significance.
IQSEC1	9922	22662237	GEP100 plays a significant role in pancreatic cancer invasion through regulating the expression of E-cadherin and the process of mesenchymal to epithelial transition (MET).
IQSEC1	9922	23747719	GEP100 regulates an Arf6/ERK/uPAR signaling cascade in EGF-induced breast cancer cell invasion.

MAGEC1	9947	12115486	Overexpression of MAGE-C1 is associated with mammary gland and ovary, non small cell lung carcinoma and metastatic melanoma
MAGEC1	9947	20696919	Peripheral blood mononuclear cells from 26 metastatic melanoma patients expressing CT7 in their tumor lesions were analyzed for CT7-specific T-cell responses using overlapping peptides.
IKZF1	10320	22859015	Ikaros is expressed at high levels in human ovarian cancer.Ikaros inhibits proliferation and, through upregulation of Slug, increases metastatic ability of ovarian serous adenocarcinoma cells.
MYBBP1A	10514	22339894	We provide experimental evidence that MYBBP1A is an important molecular switch in the regulation of tumor cell proliferation versus migration in head and neck squamous cell carcinoma cells.
NMU	10874	16878152	Overexpression of neuromedin U is associated with bladder tumor formation, lung metastasis and cancer cachexia
NMU	10874	19118941	NmU may be involved in the HGF-c-Met paracrine loop regulating cell migration, invasiveness and dissemination of pancreatic ductal adenocarcinoma.
NMU	10874	24876102	our results defined NmU as a candidate drug response biomarker for HER2-overexpressing cancers and as a candidate therapeutic target to limit metastatic progression and improve the efficacy of HER-targeted drugs.
AKAP13	11214	22161024	Amplification of AKAP-13 is associated with metastatic and aggressive papillary thyroid carcinomas.
STAB1	23166	21368224	CLEVER-1/stabilin-1 can support lymphocyte transendothelial migration; it also may provide an organ-specific signal for regulatory T cell recruitment to the inflamed liver and to hepatocellular carcinoma.
TPSG1	25823	20126998	tryptase promotes breast cancer migration and invasion.
PTPN23	25930	21724833	loss of PTPN23 may increase the activity of SRC and the phosphorylation status of the E-cadherin/beta-catenin signaling complex to promote tumor growth and invasive behavior in breast cancer
SERBP1	26135	17698176	In ovarian cancer, PAI-RBP1 is significantly overexpressed in tumor epithelial cells, suggesting a biological role in tumor invasion and metastasis. Its expression is higher in advanced disease.
MYOF	26509	22135466	MYOF plays a previously unrecognized role in cancer cell invasion.
MYOF	26509	22761893	suggest a novel role of MYOF in breast tumor cell invasion and a potential reversion to an epithelial phenotype upon loss of MYOF
DKK4	27121	18408752	DICKKOPF-4 is induced by TCF/beta-catenin and upregulated in human colon cancer, promotes tumour cell invasion and angiogenesis.
DKK4	27121	21994129	The TR/DKK4/Wnt/beta-catenin cascade influences the proliferation and migration of hepatoma cells during the metastasis process and support a tumor suppressor role of the thyroid hormone receptor.
BRD7	29117	22008115	Data indicate that BRD7 may be related to the occurrence, development, and metastasis of lung cancers.
CARD10	29775	17724468	Protein kinase C-CARMA3 signaling axis plays an essential role in lysophosphatidic acid-induced ovarian cancer cell in vitro invasion.
CARD10	29775	24633921	CARMA3 is overexpressed in pancreatic cancer and regulates malignant cell growth, invasion, and NF-kappaB signaling, which was dependent on its association with Bcl10.
REPIN1	29803	24285725	there is a p53-induced double-negative feedback loop involving miR-15a/16-1 and AP4 that stabilizes epithelial and mesenchymal states, respectively, which may determine metastatic prowess in colorectal cancer

ASAP1	50807	18400762	ASAP3 functions nonredundantly with ASAP1 to control cell movement and may have a role in cancer cell invasion.
ASAP1	50807	18519696	study suggests that the ASAP1 gene plays a role in prostate cancer metastasis and may represent a therapeutic target and/or biomarker for metastatic disease
ASAP1	50807	21858086	Data suggest that GEP100-Arf6-AMAP1-cortactin pathway, activated by VEGFR2, appears to be common in angiogenesis and cancer invasion and metastasis, and provides their new therapeutic targets.
ASAP1	50807	24427349	results indicated that elevated expression of ASAP1 plays an important role in the progression and metastasis of ovarian cancer
TMEM8B	51754	12918109	down-regulation may be closely associated with tumorigenesis and metastasis of colorectal carcinoma; may not contribute to development and progression of gastric carcinoma.
TMEM8B	51754	17641538	We demonstrated the subcellular localization and the effect of NGX6 and its mutants on the growth, proliferation, migration, or adhesion of nasopharyngeal carcinoma 5-8F cells.
TMEM8B	51754	19755717	NGX6a was significantly downregulated in nasopharyngeal caaarcinoma and is associated with tumor metastasis.
TMEM8B	51754	20543461	The short transcript of NGX6 play an important role in colorectal cancer, and reduced expression may contribute to metastasis.
TMEM8B	51754	22647848	Data suggest that nasopharyngeal carcinoma-associated gene 6 (NGX6) may inhibit colon cancer through the regulation of proteins involved in cell proliferation, metastasis, apoptosis, cytoskeletal structure, metabolism, and signal transduction.
TLR9	54106	17373717	TLR9-mediated invasion may represent a novel mechanism through which infections promote prostate cancer.
TLR9	54106	17986857	Functional expression of TLR9 is associated to the metastatic potential of human lung cancer cell.
TLR9	54106	18922969	TLR9 expression is increased in breast cancer and CpG oligonucleotide-induced cellular invasion is mediated via TLR9 and TRAF6, independent of MyD88.
TLR9	54106	19618291	TLR9 up-regulates COX-2 expression in prostate cancer cells, at least partially through the activation of NF-kappaB, which may be implicated in tumor invasion and metastasis.
TLR9	54106	21391743	Lymph node metastasis more likely occur in breast cancer patients with a positive TLR9 status and its expression might serve as an indicator of poor prognosis in patients with breast cancer.
TLR9	54106	21448350	The gene of TLR9 is correlated with the invasive and metastatic potential of human pancreatic carcinoma, and CPG ODN2216 induces the inhibition of migration and invasion of Panc-1 cells.
TLR9	54106	21607583	Expression of TLR9 isoforms A and B can be detected in clinical breast cancer specimens. The ERalpha and sex steroid hormones regulate TLR9 expression and invasive effects in the breast cancer cells.
KRT20	54474	12515621	The detection of cancer metastasis in the lymph nodes in colon carcinoma is almost doubled (21.9% vs 11.1%) by CK-20 mRNA
KRT20	54474	17240035	Dysregulation of CK20 expression is an early event in the carcinogenesis of papillary noninvasive bladder cancer.
KRT20	54474	18069772	Positive CK20 RT-PCR, depth of tumor invasion, lymph node status, metastasis and microvessel density are significantly correlated with vascular invasion.
KRT20	54474	18705345	Micrometastases of gastric cancer can be detected in circulating peripheral blood using quantitative real-time RT-PCR. CK19 is a better marker than CK18, CK20 and CEA.
KRT20	54474	19098678	It can be helpful in cases with metastatic rectal carcinoma, especially those with CK7+/CK20+ or CK20-/CK7- immunophenotype.

KRT20	54474	20395351	CK20 and VEGF expressions in peripheral blood of colorectal carcinoma patients are promising molecular markers for disease progression and metastasis.
KRT20	54474	20574624	Case Report: CK7+/CK20- Merkel cell carcinoma presenting as inguinal subcutaneous nodules with subsequent epidermotropic metastasis.
KRT20	54474	21938557	positivity rates of CK19 and CK20 in transverse mesocolon were 48.6 and 61.2%, respectively and increased with the depth of tumor invasion
KRT20	54474	22752373	High cytokeratin 20 mrna expression is associated with lymphatic metastasis in colon cancer.
KRT20	54474	24403457	High cytokeratin 20 expression is associated with invasive histological phenotype in poorly differentiated colorectal adenocarcinoma.
RNF111	54778	23467611	findings indicate that Arkadia is not critical for regulating tumor growth per se, but is required for the early stages of cancer cell colonization at the sites of metastasis
TRPM7	54822	22871386	Findings suggest that TRPM7 is part of a mechanosensory complex adopted by cancer cells to drive metastasis formation in breast cancer.
TRPM7	54822	23353055	TRPM7 regulates migration and invasion of metastatic breast cancer cells via MAPK pathway.
ANO1	55107	22912841	Enhanced expression of ANO1 in head and neck squamous cell carcinoma causes cell migration and correlates with poor prognosis.
ANO1	55107	23866066	As the different TMEM16A isoforms do not affect proliferation or migration and do not associate with tumors, our results suggest that the resulting channel activities are not directly involved in cell growth and motility.
ANO1	55107	24825187	investigated whether DOG1 plays a role in cell migration, apoptosis, proliferation and viability in gastrointestinal stromal tumors
FBXW7	55294	22270966	miR-223 targets FBXW7/hCdc4 expression at the post-transcriptional level and appears to regulate cellular apoptosis, proliferation, and invasion in gastric cancer.
CCDC88A	55704	19211784	provides the structural and biochemical basis for the prometastatic features of GIV, making the functional disruption of this unique G alpha i-GIV interface a promising target for therapy against cancer metastasis
CCDC88A	55704	20462955	Data show that inclusion or exclusion of GIV's GEF motif, which activates Galphai, modulates EGFR signaling, generates migration-proliferation dichotomy, and most likely influences cancer progression.
CCDC88A	55704	20974669	It was concluded that GIV-fl is a novel metastasis-related protein and an independent adverse prognosticator that may serve as a useful adjunct to traditional staging strategies in colorectal carcinoma.
CCDC88A	55704	22116776	Girdin protein may be a potential new distant metastasis biomarker of breast cancer
CCDC88A	55704	22714912	The Girdin protein may be a potential new early liver metastasis biomarker of colorectal cancer.
CCDC88A	55704	24326843	Up-regulated autophagy was negatively associated with Girdin level. There was a significant correlation between Girdin expression and lymph nodes metastasis in invasive ductal breast carcinoma.
EXOC2	55770	22761837	We identified interactions between RalA and its effectors sec5 and exo84 in the Exocyst complex as directly necessary for migration and invasion of prostate cancer tumor cells.
ADAMTS9	56999	18449890	ADAMTS9 expression was downregulated or lost in 17 of 23 (73.9%) lymph node metastatic nasopharyngeal carcinoma (NPC) specimens. After transfection of the ADAMTS9 gene into 7 NPC cell lines, a dramatic reduction of colony forming ability was observed.
MKL1	57591	24084383	results provided novel evidence supporting the metastasis-promoting functions of MRTF-A, and implied that MRTF-A might be a switch for the estrogen pathway to change its proliferation-promoting roles into migration-stimulating roles in breast cancer
MKL1	57591	24189459	Overexpression of SMYD3 promotes MRTF-A-mediated upregulation of MYL9 and migration of MCF-7 breast cancer cells

ZNF350	59348	19996286	Our findings suggest that ZBRK1 acts to inhibit metastasis of cervical carcinoma, perhaps by modulating MMP9 expression.
CDH23	64072	22413011	cadherin-23 is up-regulated in breast cancer tissue versus normal tissue and we propose that cadherin-23-mediated heterotypic adhesion between invading tumor cells and stromal fibroblasts may play a role in the metastatic cascade.
CSMD1	64478	19669408	Reduction of CSMD1 expression significantly associated with high tumour grade and decreased overall survival in invasive ductal breast carcinoma
MMP28	79148	21615884	MMP28 is frequently overexpressed during progression of gastric carcinoma, and contributes to tumor cell invasion and metastasis.
MMP28	79148	23803888	Inhibition of BCMO1 expression is associated with increased invasiveness of colon cancer cells and increased expression of MMP7 and MMP28. beta-Carotene can upregulate BCMO1 and reverse these effects.
MUS81	80198	21187482	Mus81 down-regulation correlated significantly to invasion depth (p = 0.015) and poorly-differentiated type (p = 0.016) of gastric cancer.
ADAM33	80332	19265133	ADAM33 has a key role in gastric cancer pathogenesis by up-regulating the interleukin (IL)-18 secretion process, which results in increased cell migration and proliferation.
ADAM33	80332	19267929	We suggest that ADAM33 promoter methylation may be a useful molecular marker for differentiating invasive lobular carcinoma and invasive ductal carcinoma
ANKRD30A	91074	17536312	testing demonstrates NY-BR-1 in a mostly focal fashion in the epithelia of ducts and acini of the mammary gland. In tumors, homogenous staining can be seen in almost all ductal carcinomas in situ and/or the intraductal component of invasive carcinomas.
MUC16	94025	14566828	value of this tumor marker regarding relapse, metastasis and death in resectable non-small cell lung cancer
MUC16	94025	14676194	might contribute to the metastasis of ovarian cancer to the peritoneum by initiating cell attachment to the mesothelial epithelium via binding to mesothelin
MUC16	94025	17067392	MUC16-Mesothelin binding is a high affinity, N-glycan dependent interaction that facilitates peritoneal metastasis of ovarian tumors.
MUC16	94025	18042071	The frequency of expression of Ca125...in invasive micropapillary carcinomas...was similar to the results in unselected mammary carcinoma.
MUC16	94025	18317225	In ovarian metastases from undiagnosed colorectal adenocarcinomas, elevated CA-125 levels and frequent coexpression of cytokeratin 7 are features that can contribute to misclassification of these metastases as primary ovarian neoplasms.
MUC16	94025	19270645	Invasive micropapillary carcinoma more commonly showed immunoreactivity for MUC1, CA125, and Her2Neu compared to invasive urothelial carcinoma with retraction artifact
MUC16	94025	20090516	adenocarcinomas with MUC16 expression may have an increased risk for metastases to pleura/peritoneum but not the leptomeninges or brain
MUC16	94025	21421261	Ectopic expression of the MUC16CTD enhances SKOV3 tumor cell growth, colony formation in soft agar and enhances tumor growth and metastases in SCID mice.
MUC16	94025	21836488	Describe CA125 immunoreactivity in 8 of 10 metastatic mucinous carcinomas arising after a diagnosis of primary ovarian mucinous carcinoma or mucinous borderline tumor of the intestinal type in which the primary neoplasms were mostly negative.
MUC16	94025	22727919	Data suggest that preoperative serum CA-125 can be used as a significant independent predictor of advanced stage of endometrial carcinoma as well as lymph node metastases.
MUC16	94025	23915849	STIP1 histoscores may be useful in detecting invasive human ovarian cancer in patients with low serum CA125 levels.
JDP2	122953	20677166	JDP2 expression was downregulated in pancreatic carcinoma & this correlated with metastasis & decreased post-surgery survival.
CCDC67	159989	22610074	suggest that CCDC67 is a putative tumor suppressor gene that is silenced in gastric cancers by promoter CpG methylation and that it may play an important role in

			cell signaling and migration related to tumorigenesis
VWCE	220001	19413886	Data suggest that URG11 contributes to gastric cancer growth and metastasis at least partially through activation of beta-catenin signalling pathway.
EPHA10	284656	24403271	EphA10 expression at both the gene and protein level in clinical breast cancer tissues is significantly associated with lymph node metastasis as well as stage progression.
MACC1	346389	21093878	Metastasis associated in colon cancer 1 gene overexpression may be a useful marker for predicting postoperative recurrence in patients with lung adenocarcinoma after surgery.
MACC1	346389	21447729	Expression analysis of MET, MACC1, and HGF in metastatic colorectal cancer.
MACC1	346389	21498695	MACC1 is more frequently expressed in vascular invasive hepatocellular carcinoma.
MACC1	346389	22179665	Concomitant downregulation of miR-1 and increase of MACC1 can thus contribute to MET overexpression and to the metastatic behavior of colon cancer cells.
MACC1	346389	22838389	the identification of coding MACC1 SNPs in primary colorectal tumors does not improve the prediction for metastasis formation or for patients' survival compared to MACC1 expression analysis alone.
MACC1	346389	23095620	In stage II colon cancer, MACC1 expression predicts development of metastases, outperforming microsatellite stability status, as well as KRAS/BRAF mutation status.
MACC1	346389	23166620	Circulating MACC1 transcripts in colorectal cancer patient plasma predict metastasis and prognosis.
MACC1	346389	23457029	MACC1 also promoted the proliferation, migration and invasion of both gastric cancer cell lines.
MACC1	346389	23665971	MACC1, a new easily detectable biomarker in cancer, is an independent prognostic factor of recurrence after liver resection of colorectal cancer metastasis.
MACC1	346389	24124150	MACC1 is related to colorectal cancer initiation and early-stage invasive growth.
MACC1	346389	24310811	MACC1 protein and mRNA overexpression in both NSCLC tissues and cell lines is related to tumor recurrence, metastasis, and prognosis

SUPPLEMENTARY REFERENCES

1. Kumar P, Henikoff S, Ng PC. Predicting the effects of coding non-synonymous variants on protein function using the SIFT algorithm. Nat Protoc 2009; 4: 1073-1081.
2. Adzhubei I, Jordan DM, Sunyaev SR. Predicting functional effect of human missense mutations using PolyPhen-2. Curr Protoc Hum Genet 2013; Chapter 7: Unit7.20.
3. Reva B, Antipin Y, Sander C. Predicting the functional impact of protein mutations: application to cancer genomics. Nucleic Acids Res 2011; 39: e118.
4. Gonzalez-Perez A, Lopez-Bigas N. Improving the assessment of the outcome of nonsynonymous SNVs with a consensus deleteriousness score, Condel. Am J Hum Genet 2011; 88: 440-449.
5. Shihab HA, Gough J, Cooper DN et al. Predicting the functional, molecular, and phenotypic consequences of amino acid substitutions using hidden Markov models. Hum Mutat 2013; 34: 57-65.
6. Gonzalez-Perez A, Deu-Pons J, Lopez-Bigas N. Improving the prediction of the functional impact of cancer mutations by baseline tolerance transformation. Genome Med 2012; 4: 89.
7. Kim TM, Jung SH, An CH et al. Subclonal Genomic Architectures of Primary and Metastatic Colorectal Cancer Based on Intratumoral Genetic Heterogeneity. Clin Cancer Res 2015; 21: 4461-4472.
8. Comprehensive molecular characterization of human colon and rectal cancer. Nature 2012; 487: 330-337.
9. Price TJ, Peeters M, Kim TW et al. Panitumumab versus cetuximab in patients with chemotherapy-refractory wild-type KRAS exon 2 metastatic colorectal cancer (ASPECCT): a randomised, multicentre, open-label, non-inferiority phase 3 study. Lancet Oncol 2014; 15: 569-579.
